# Mitochondria and Neuromast Tagging With Fluorescent Gallium‐Triapine Analogues: In Cellulo MP FLIM and Zebrafish Live Imaging

**DOI:** 10.1002/advs.202519815

**Published:** 2026-04-16

**Authors:** Megan J. Green, Michael W. Jones, Merissa Saleem, Haobo Ge, Melita Tvardauskaite, Julia Dudzic, Fernando Cortezon‐Tamarit, Rory L. Arrowsmith, Charareh Pourzand, Gabriele Kociock‐Kohn, Nicholas H. Rees, Jonathan R. Dilworth, Stephen Faulkner, Stanley W. Botchway, David Gurevich, Sofia I. Pascu

**Affiliations:** ^1^ Department of Chemistry University of Bath Claverton Down Bath UK; ^2^ Chemistry Research Laboratory University of Oxford Oxford UK; ^3^ Department of Life Sciences University of Bath Claverton Down Bath UK; ^4^ Central Laser Facility Rutherford Appleton Laboratory Research Complex at Harwell STFC Didcot UK; ^5^ Centre For Bioengineering & Biomedical Technologies (CBio) University of Bath Bath UK

**Keywords:** BODIPY conjugates, cellular uptake, correlated confocal microscopy, metal‐thiosemicarbazones, MP FLIM, zebrafish imaging

## Abstract

We report on the synthesis and imaging‐based investigations of fluorescent thiosemicarbazone–BODIPY conjugates and their gallium(III), indium(III), and iron(III) complexes, and their cellular and in vivo distribution using multiphoton fluorescence lifetime imaging microscopy (MP‐FLIM) in living cancer cells and live zebrafish. A panel of novel tridentate thiosemicarbazone ligands and their corresponding lipophilic and monocationic complexes with 1:2 metal‐to‐ligand stoichiometry was synthesized. These compounds are structurally related to clinically relevant thiosemicarbazone‐based systems with imaging and diagnostic applications. Comprehensive analytical characterization confirmed these probes’ identity, while their complex solution behavior was evaluated under biologically relevant conditions. Cellular uptake and subcellular distribution were investigated in HeLa and PC‐3 cells using correlated confocal microscopy and MP‐FLIM, revealing preferential mitochondrial localization for selected gallium complexes. In vivo imaging in zebrafish further unraveled the biodistribution of these fluorescent metal complexes, including selective labelling of neuromast structures. The combined in vitro and in vivo imaging data provide direct insight into the speciation, stability, and spatial distribution of thiosemicarbazone metal complexes in complex biological systems. This work establishes new fluorescent thiosemicarbazones as versatile platforms for studying metal complex behavior and biodistribution by advanced optical imaging techniques, opening new opportunities for advanced development of new theranostic applications.

## Graphical abstract

Fluorescent BODIPY‐conjugated thiosemicarbazone ligands and their Ga(III), In(III), and Fe(III) complexes, inspired by Triapine, are developed as theranostic agents. Multiphoton FLIM and confocal microscopy in cancer cells and zebrafish reveal real‐time uptake, mitochondrial localisation, and whilst spectroscopic assays indicated preserved complex integrity, the resease of gallium(III) ions in vivo is possible.

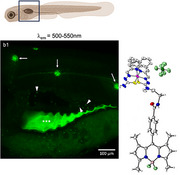

## 1 Introduction

Triapine, a heterocyclic mono(thiosemicarbazone)‐based drug reached clinical trials for ovarian cancers [1], as well as advanced head and neck cancers [2], with promising results seen in phase I and II clinical trials when administered intravenously [3, 4], Thiosemicarbazones such as Triapine are reported to be cytotoxic via inhibition of either ribonucleotide reductase (RR) or topoisomerase‐II (TI), essential to cell growth and proliferation. It has been proposed that the mechanism involves the free ligand sequestering iron, thereby disrupting the tyrosyl radical within the catalytic pocket of the R2 subunit of ribonucleotide reductase (RR). This renders the enzyme inactive and halts the cell cycle at the G1/S phase [5]. A deeper understanding of the interactions between such compounds and cellular systems is still required. While radiolabeled analogues, such as copper‐64 complexes, have shown high tumor uptake in murine models, the subcellular fate and stability of these complexes in biological environments and its precise mode of action at the cellular level remain poorly understood [6]. Moreover, the therapeutic potential of Triapine and its advancement in clinical trials and practical use have been reduced due to high toxicity leading to side effects, demonstrated ineffectiveness against solid tumors and poor water solubility [7]. Despite extensive testing in multiple clinical trials, for example, as a monotherapy on pancreatic and renal cell carcinomas [8, 9], or advanced leukemia [10], Triapine reached dose‐limiting concentrations in patients and was no longer deemed effective. Triapine has shown enhanced efficacy when used in combination with other therapeutics, such as the platinum‐based drug cisplatin and radiation, against cervical and vaginal cancers [11]. Whilst the Triapine‐Cisplatin‐radiation combination prevents further tumor growth, major side effects that come with the use of Triapine have been documented, such as methemoglobinemia and hypoxia [12].

There is considerable interest in modifying the pharmaceutical profile of thiosemicarbazones (TSCs) through structural changes that may enable repurposing and/or improved drug‐like properties [13]. Dp44mT, a functional TSC bearing similar heterocyclic groups, has shown activity against a wide range of cancers [14, 15]. When chelated to transition metals such as iron or copper, Dp44mT complexes undergo redox cycling to generate reactive oxygen species (ROS) [16, 17] thereby exacerbating the already elevated ROS levels in tumors and inducing apoptotic cell death. Additional mechanisms of action for Dp44mT and Triapine have also been reported. For Dp44mT, inhibition of topoisomerase IIα, leading to reduced cell growth and apoptosis, has been demonstrated in breast cancer cells. However, this activity showed poor correlation with DNA topoisomerase IIα mRNA levels in other cell lines, where both Dp44mT and Triapine were shown to lack inhibitory effects as catalytic inhibitors or poisons of DNA topoisomerase IIα in CHO or NCI 60‐cell line models [19]. Dp44mT has also been evaluated as a glioblastoma therapeutic; despite its lipophilicity, limited blood–brain barrier penetration reduces its efficacy [20].

**FIGURE 1:**
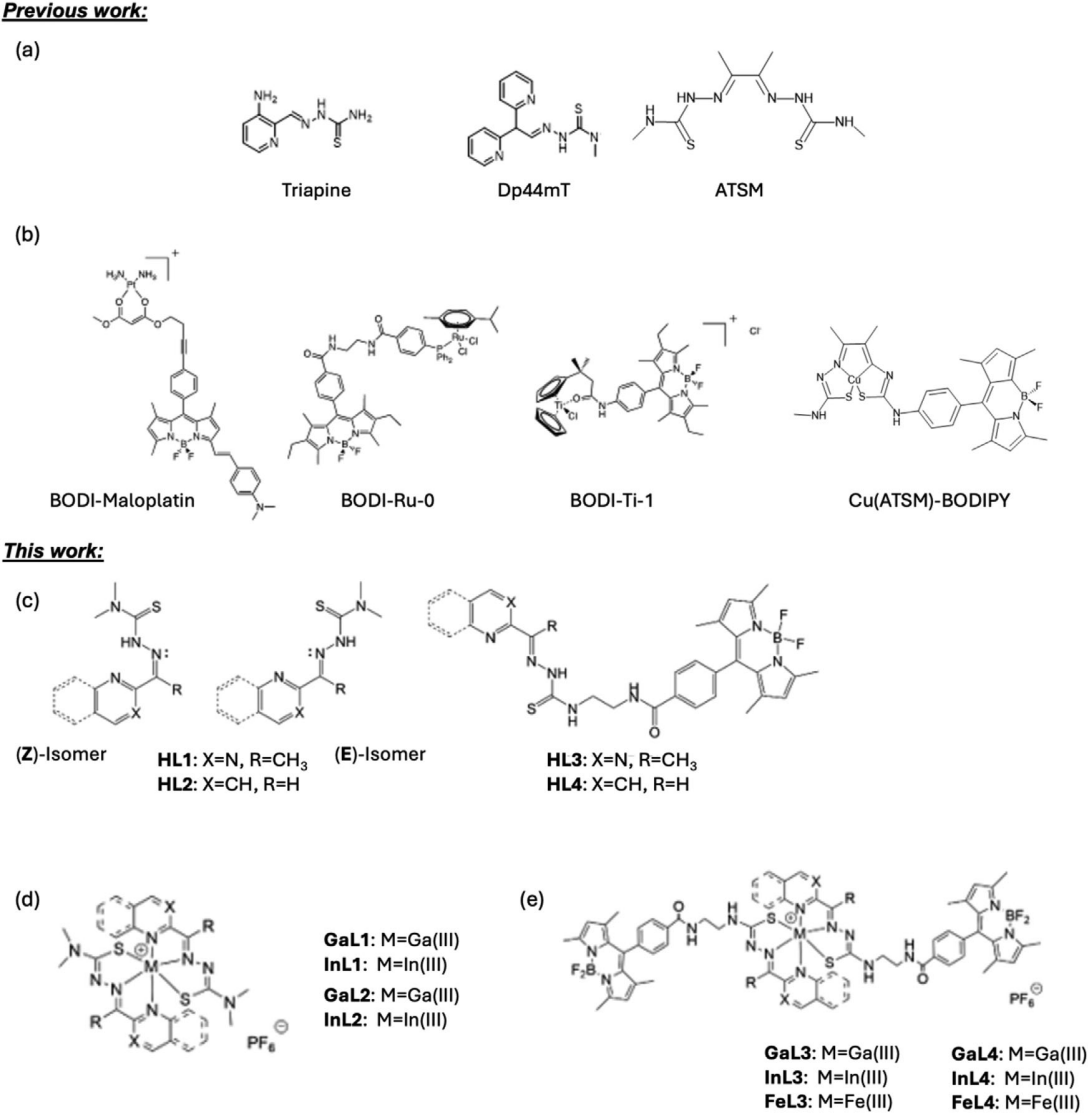
Previous work: (a) Structural representations of heterocyclic mono(thiosemicarbazones) reaching clinical trials; (b) BODIPY dyes as molecular tags and BODIPY‐tagged metal complexes of known cancer drugs [42]; This work: (c) The general framework of the small molecule compounds in our new family of tridentate pyrimidyl/quinolyl thiosemicarbazones of relevance to Triapine‐based drugs, including the two possible isomers of thiosemicarbazones studied hereby: (**E**)‐ and (***Z***)‐ isomers of pyrimidyl thiosemicarbazone (**HL1**) and quinolyl thiosemicarbazone (**HL2**) and corresponding Bodipy conjugates of pyrimidyl (**HL3**) and quinolyl (HL4) thiosemicarbazones. (d) General structural representation of the M(III) labelled TSC complexes of monoanionic pyrimidyl and quinolyl thiosemicarbazonato ligands (**L1** ^−^ and **L2** ^−^, respectively), as PF_6_^−^ salts. (e) General structural representation of the M(III) labelled TSC‐Bodipy conjugates of monoanionic pyrimidyl and quinolyl thiosemicarbazonato ligands denoted **L^3^** ^−^ and **L^4^** ^−^, respectively, as PF_6_^−^ salts, reported hereby.

Triapine and Dp44mT are structurally distinct yet share heterocyclic units and the thiosemicarbazone motif (Figure 1a), accounting for differences in efficacy, side effects, and in vivo uptake. A key structural similarity is the presence of a heterocyclic ring on the TSC backbone, which increases lipophilicity and facilitates diffusion across the cellular lipid bilayer [21, 22]. Other heterocycle‐containing TSCs have also shown anticancer activity, and the topoisomerase IIα inhibition and antiproliferative effects of α‐heterocyclic thiosemicarbazones related to Triapine and their corresponding copper(II) complexes have been extensively studied [23, 24, 25, 26, 27, 28, 29]. The Cu(II)‐Cl thiosemicarbazonato‐based complexes were found to catalytically inhibit topoisomerase IIα at concentrations (0.3–7.2 µm) more than an order of magnitude lower than the corresponding free ligands, and to preferentially inhibit proliferation in breast cancer cells with high topoisomerase IIα expression (SK‐BR‐3) compared with cells expressing lower levels (MCF‐7) [23, 24, 25, 26]. More broadly, formation of metal complexes has been widely reported to substantially enhance the antineoplastic activity of TSCs [30].

Overall, standard cytotoxicity assays of Triapine and related thiosemicarbazones confirm efficient cellular uptake but provide only very limited information on intracellular localization. Directly tracing these analogues in cells is challenging, as Triapine is not intrinsically fluorescent, necessitating fluorophore conjugation to enable cellular imaging. Metal‐coordinated TSC complexes have been reported to show greater therapeutic activity than the corresponding free ligand alone and have shown promising activity in multiple cancer cell lines [31, 32, 33, 34, 35, 36]. The formation of metal complexes also provides opportunities to increase the therapeutic potential of these compounds through radiolabeling of the metal center for applications in positron emission tomography (PET) and single photon emission computed tomography (SPECT) imaging [37]. For example, ^64^Cu‐labelled (diacetyl*‐bis* (N4‐methylthiosemicarbazone) copper (II)), [^64^Cu]Cu(ATSM), is a well‐documented hypoxia‐selective TSC, has been studied preclinically as a theranostic having both the ability to diagnose and treat tumors.

In vivo nuclear imaging modalities lack the spatial resolution needed to observe the nanometer‐scale distribution and intracellular speciation of metal complexes within cells, both of which are critical to understanding pharmacological mechanisms. While many PET and SPECT agents are designed to target extracellular receptors, intracellular uptake is common, and the fate of these agents’ post‐internalisation is often governed by the physicochemical properties and kinetic stability of the metal complex acting as the molecular probe. Tracking these dynamics at cellular resolution, however, remains a significant challenge. While one‐ and two‐photon confocal fluorescence microscopy (CFM) are widely used to visualize molecular interactions of organic and biological species in live cells, they cannot report on the chemical state of fluorophores or metal complexes. In contrast, fluorescence lifetime imaging microscopy (FLIM) is sensitive to the local chemical environment and can therefore reveal changes associated with complexation, polarity, and biomolecular binding.

Understanding the cellular behavior of cytotoxic metallo‐complexes is crucial for elucidating their mechanism of action in living systems. For gallium complexes in particular, broad speciation in aqueous environments has been noted in previous TSC‐related work, and here multiphoton lifetime imaging was employed to monitor this speciation directly in cells. One‐ and two‐photon CFM has been widely used to visualize molecular interactions of organic and biological species within living cells, but it lacks the ability to report on the chemical state of fluorophores or metal complexes. By contrast, multiphoton FLIM (MP FLIM) provides sensitivity to the local chemical environment and can be used to infer changes in complexation, polarity, or binding. Despite this, no single analytical method provides simultaneous real‐time information on both spatial distribution and chemical stability information about metal complexes in living cells [31, 32, 33, 34, 35, 36].

Fluorescence labelling of compounds with robust and kinetically stable tags such as boron‐dipyrromethene (BODIPY) enables direct tracking of localization, stability, and accumulation in cells. Excellent thermal and photophysical stability, negligible triplet formation, good solubility, high quantum yields and their ability to respond to far red and near‐infrared (NIR) excitation are crucial characteristics for imaging probes and make BODIPY attractive for biological imaging. These dyes exhibit strong two‐photon absorption and fluorescence emission across excitation 600–1350 nm, allowing for deeper tissue penetration and imaging with minimal phototoxicity, reduced light scattering, and low tissue autofluorescence.

A range of BODIPY‐tagged metal–complex based fluorophores have been applied to investigate the mechanism of metallodrugs, a selection shown in Figure 1b. For example, titanocene dichloride tagged with a BODIPY fluorophore (BODI‐Ti‐1) showed minimal nuclear accumulation at concentrations up to 50 mm and cellular interactions assays indicated mitochondrial, rather than DNA, targeting. BODI–Ru‐0, a BODIPY‐tagged analogue of RAPTA‐C and related complexes [37] demonstrated quick uptake into cancer cells with non‐specific cytoplasmic spreading, implying the BODIPY tag drives accumulation. BODIPY complexes have also been developed for photodynamic therapy, such as BODI‐maloplatin, based on the anticancer drug maloplatin‐B, which upon deep‐red light irradiation at the 660 nm wavelength produced singlet oxygen and induced apoptotic cell death, localizing primarily to mitochondria. Despite these significant advantages over cellular imaging using one‐photon excitation only, their integration into metal complex systems, especially thiosemicarbazones, remains scarce.

Previous work [38, 39] focused on Cu(ATSM)‐BODIPY labelled complex hybrids allowed unprecedented insight into the behavior of this class of metal complex within cells. Co‐localization studies with an ER‐tracker in HeLa cells showed strong ER correlation and general cytoplasmic dispersion, with the Cu(ATSM)‐BODIPY complex remaining virtually intact after within an hour of cellular incubation. Moving toward molecular imaging in the NIR region by using multiphoton excitation at 810, 910 nm and beyond should improve tissue penetration and reduced autofluorescence, leading to minimal photodamage, and more accurate, non‐invasive imaging.

An important aim of this work was to evaluate whether the introduction of a fluorophore altered the cell‐penetrating properties of Triapine‐inspired, heterocyclic, thiosemicarbazones (TSCs). MTT assays confirmed that these derivatives are widely taken up by cells, but such assays provide no insight into their intracellular localization. To address this unmet need, we have synthesised a series of novel, BODIPY‐tagged metal complexes of thiosemicarbazones, structurally related to Triapine (Figure 1c,d) and investigated their behavior utilizing complementary CFM and FLIM imaging approaches.

Octahedral gallium(III) and iron(III) complexes of tridentate monothiosemicarbazones were originally reported by Kowol and Keppler et al., who demonstrated that the in vitro cytotoxicity of such complexes exceeded that of the corresponding free ligands. [24, 25] Although the full mechanism of action remains unclear, gallium(III) was hypothesized to coordinate strongly to alpha‐N‐thiosemicarbazones and yield cytotoxic complexes. With this synthetic platform in hand, we examined whether coordination to gallium perturbs the distribution of the BODIPY‐tagged thiosemicarbazone (TSC) analogues. Subtle redistribution was observed additionally the localization of the metal complexes broadly like that of the free BODIPY‐labelled ligand. Because no direct evidence currently exists regarding the intracellular stability of these main group metal‐labelled constructs, we ascertain that it would be of interest to examine their behavior using FLIM, given that the free ligand is known to fluoresce with lifetimes distinct from those of its metal complexes.

An important aim of our work was the evaluation of cellular localization and speciation of gallium(III) and indium(III) complexes of pseudo‐octahedral complexes of pyrimidine and quinoline thiosemicarbazone ligands, utilizing HeLa and PC‐3 cells. The sensitivity of metal‐luminescence lifetimes to the local cellular environment and environmental changes render MP FLIM a unique diagnostic tool for tracking and reporting on localization, subcellular compartmentalization, metal–ligand integrity, and uptake dynamics. Specifically, our investigations of the temporal response of novel metal‐ion‐coordinated chromophores demonstrated their capability of exhibiting changes over picosecond to nanosecond timescales. These chromophores can therefore embed within intricate biomaterial environments and respond to changes in their lifetime rather than emission parameters alone.

We further complemented our in vitro design and testing of new BODIPY‐conjugates by exploring the real‐time behavior and uptake of fluorescently tagged tridentate thiosemicarbazone–metal complexes in live zebrafish embryos. Combining optical transparency and cellular resolution ideal for live imaging, toxicity profiling and tissue‐specific mapping, zebrafish also provide a physiologically relevant milieu for assessing biodistribution, complex stability, and off‐target effects, offering unique insight into the pharmacokinetics and intracellular pharmacology of these agents [40, 41]. Our results revealed the uptake of all fluorescent compounds into zebrafish, specifically organs of the digestive system. Moreover, our new gallium(III) quinoline‐thiosemicarbazone complex showed the unexpected labelling of mitochondria in a manner similar to that of its Fe(III) analogue, a quality conserved between zebrafish and tissue culture experiments. Specifically, gallium‐ and iron(III) quinoline‐based and BODIPY tagged compounds **GaL4** and **FeL4** both marked neuromasts in zebrafish, a mitochondria‐rich organ structure, where all other species investigated hereby indicated nonspecific uptake. Taken together, our results, discussed below, establish these BODIPY‐tagged thiosemicarbazone–metal complexes as powerful tools for correlating real‐time imaging of intracellular localization and highlighting their promise as targeted therapeutic candidates, paving the way for their rational development as future theranostic agents.

## 2 Results and Discussions

### 2.1 Synthesis and Characterization

**SCHEME 1:**
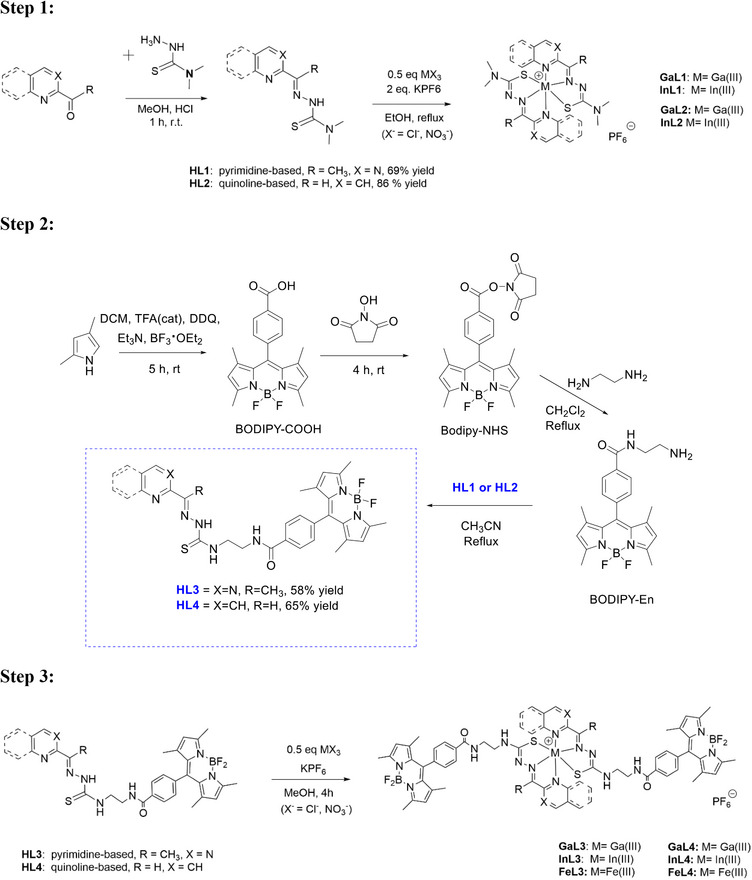
Stepwise synthetic protocols for the synthesis of the ligands and their respective gallium and indium complexes. Metalation of **HL1‐HL2** in the presence of an excess of KPF_6_ gave rise to the corresponding [M(L)_2_)]PF_6_ species in moderate yields denoted **GaL1, InL1, GaL2** and **InL2** (see Supporting Information). The corresponding BODIPY substituted complexes **GaL3, InL3, GaL4** and **InL4** were also synthesized by analogous methods, as detailed in the Experimental Section.

**FIGURE 2:**
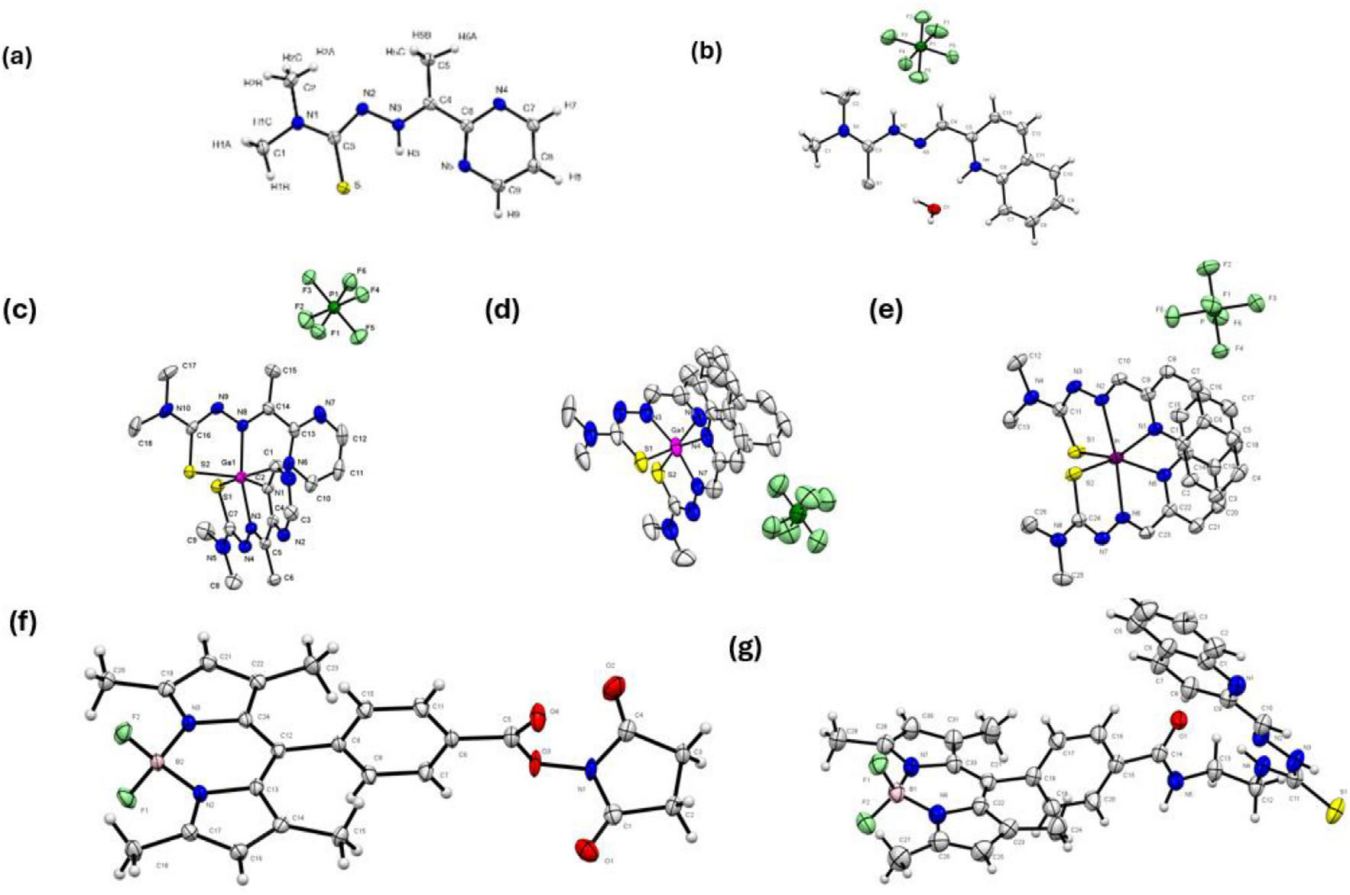
Single crystals X‐ray diffraction analysis showing displacement ellipsoid plots (30% probability) for the molecular structures of Compounds: **HL1** (a), **HL2** (HPF_6_ and H_2_O adduct, b), **GaL1** (c), **GaL2** (d) **InL2** (e) **BODIPY‐NHS** (f), and **HL4** (g). For **GaL1**, **GaL2** and **InL2**, the H atoms were not included, for clarity.

A panel of Ga(III) and In(III) complexes of pyrimidyl‐aldehydethiosemicarbazone (**HL1**), and quinolyl‐aldehydethiosemicarbazone (**HL2**), both bearing a 4,4‐dimethyl‐thiosemicarbazone functionalities have been synthesised and characterized (see Experimental Section). A general method for synthesizing the Ga(III) and In(III) complexes of **HL1–HL4** involved treating 2 equiv. of the ligand with the corresponding metal nitrate salt (1 equiv.). The components were dissolved in a minimal volume of MeOH (*ca*. 10 mL) and stirred at room temperature for 3 h. An excess of KPF_6_ (up to 3 equiv.) was then added, and the reaction was stirred for an additional hour. The precipitated product was collected by vacuum filtration and washed with cold MeOH, followed by recrystallisation from CH_2_Cl_2_ and Et_2_O. The as‐prepared TSC pro‐ligands such as **HL1,** which is new, and the previously reported **HL2**, were conjugated with the BODIPY‐ethylenediamine, in a stepwise protocol involving NHS‐activated BODIPY‐COOH, denoted BODIPY‐NHS. This precursor was obtained by a modification of a previously published method [43, 44] and fully characterized structurally hereby. Therefore, simple demethylated monothiosemicarbazonato ligand frameworks were derivatized with a functional BODIPY moiety through the introduction of an ethylene diamine linker, resulting in the formation of pyrimidyl‐ and quinolyl‐BODIPY‐labelled thiosemicarbazone ligands **HL3 (**pyrimidine‐based, *X *= N, *R* = CH_3_) and **HL4** (quinoline‐based, *X* = CH, *R* = H), respectively. Additionally, in the case of **HL1**, the precursor BODIPY‐NHS and **HL4**, the single crystals X‐ray diffraction confirmed the corresponding molecular structure, including, in the case of **HL1** and **HL4**, the preferred E isomer in the solid state. Compound **HL2** has been previously structurally characterized [26], yet a new, protonated variant is also reported hereby. The new compounds **GaL1**, **GaL2 and InL2** were also characterized by single‐crystal X‐ray diffraction which confirmed the pseudo‐octahedral geometry of the Ga(III) ion, bound in the environment of two N/N/S donors through the coordination of two mono‐anionic **L^2^** ^−^ units and the presence of the PF6^−^ counterion. For the corresponding Ga(III), In(III) and Fe(III)‐BODIPY conjugates of **HL3** and **HL4**), an analogous synthetic method as described above was followed and further purification by flash chromatography (CH_2_Cl_2_:MeOH, 9:1) was additionally performed to obtain material of advanced purity for in vitro and in vivo imaging. Scheme 1 summarizes the synthetic routes applied for the ligands and complexes used in this study, and Figure 2 depicts the molecular structures of the compounds analyzed by single‐crystal X‐ray diffraction hereby. Full details of the synthesis and characterization of the ligands and metal complexes can be found in the Experimental Section, and the crystallographic details are given in the Supporting Information.

All new ligands and Group 13 complexes were characterized by ^1^H NMR, (Figures S1–S17), ES‐MS^+^ (Figures S20–S29), gradient reverse phase HPLC in H_2_O: CH_3_CN (95:5% v/v, using 0.1% TFA in the mobile phase) (Figures S30–S39), and solid‐state IR spectroscopy (Figures S18, S19), and, where possible, by DOSY, ^1^ ^3^C{^1^H} NMR and HMQC. Extensive ^1^ ^1^B and ^1^ ^9^F NMR spectroscopy was performed for the free ligands such as, for example, **HL4** (Figure 2) as well as for the gallium and indium BODIPY conjugates; for **GaL3‐GaL4** and **InL3‐InL4**. The ^1^ ^9^F spectra, although broad, confirmed the presence of the PF_6_^−^ counterion in the expected 3:2 ratio relative to the two BF_2_ units, demonstrating complete metathesis from the nitrate salts. DOSY NMR experiments of **HL3**, **HL4, GaL4** and **InL4** indicated the formation of single, well‐defined species and confirmed that the BODIPY conjugates remain stable for over days in DMSO solutions under air. Consistent with previous reports [23, 24, 25, 26], thiosemicarbazones in this family can act as tridentate ligands toward metal ions, coordinating through a sulfur atom, a heterocyclic nitrogen, and an imine nitrogen. This coordination mode involves deprotonation of the hydrazinic N–H and tautomerization from the thione to the thiol form of the ligand, however complex equilibria involving protonation and isomers formation were observed in solution in line with well‐established dynamic exchange behavior of thiosemicarbazones [23, 24, 25, 26]. Interestingly, broadening of peaks and protonation do occur in DMSO solutions coupled with the emergence of resonances in the ^1^H NMR spectra at ca. 11‐12 ppm (vide infra).

The loss of ligand‐specific hydrazinic proton resonances was observed in the ^1^H NMR spectra of all complexes derived from **HL1‐HL2**. The NMR spectroscopy of **HL2** and of its **InL2** complex (Figure S5) showed the complete disappearance of the resonance at ca. 11.8 ppm upon metal coordination. ^1^H NMR (Figures S1–S13) and ESI‐MS data (including HRMS‐ESI) supported the formation of deprotonated ligands and the corresponding monocationic metal complexes in the series (Figures S20–S29). Upon standing of all metal complexes in aqueous DMSO, the appearance of proton resonances at ca. *δ* = 11–12.5 ppm and broadening of all peaks (a entioned above) suggested partial speciation and protonation behavior over days, which is in line with the well‐documented behavior of Ga(III) complexes in aqueous media. Extensive ^1^H NMR experiments with solvent suppression confirmed complex protonation behavior in line with expected Ga(OH)x and In(OH)x clusters formation, as well as the possible cyclisation of thiosemicarbazone ligands with elimination of NH_4_OH, which occurred over several weeks in DMSO solutions. It has been possible to acquire the ^71^Ga NMR spectrum for **GaL1** and **GaL4**, which showed broad resonances at ca −1.81 ppm (**GaL2**) and −1.93 ppm (**GaL4**) that were significantly changed from the free Ga(III) resonance in DMSO‐d_6_ which in our hands occurred as a broad resonance at ca. 253 ppm. For proof of concept, labelling of **HL1** with a generator‐produced ^68^Ga (a positron‐emitting radionuclide with short physical half‐life *t*_1/2_ = 68 min, i.e., is clinically relevant,) was also demonstrated via our previously established protocols [45] leading >90% radiochemical incorporation at room temperature.

Iron(III)–BODIPY complexes **FeL3** and **FeL4** were synthesised following a similar synthetic procedure with their gallium analogues, in a one‐pot reaction. Filtration and washing with cold ethanol afforded **FeL3** as a red solid (85% yield) and **FeL4** as a red solid (82% yield). ESI‐MS^+^ data showed a peak at m/z 1230.407 for **FeL3** corresponding to the [M+H]^+^ ion of the pyrimidine complex, and a peak at m/z 1300.417 for **FeL4** corresponding to the [M+H]^+^ ion of the quinoline complex.

In all cases, the IR spectroscopy (Figure S18) aided the solid‐state structural investigations, whereby the NH stretching region in thiosemicarbazone (TSC) ligands is a useful indicator of metal coordination and protonation state. Consistent with literature reports in our hands the free thiosemicarbazone (TSC) ligands showed the characteristic N─H stretching vibrations arising from the hydrazinic and exocyclic/terminal ─NH groups which typically appeared in the 3200–3400 cm^−^^1^ region of the corresponding IR spectra of **HL1‐HL4**. These broad bands appeared split, reflecting hydrogen‐bonding interactions and the presence of more than one non‐equivalent N*H* environment in free ligands. Hydrogen bonding and conjugation across the C═N─N─C(S) framework, combined with E/Z isomerism present in the free ligands, which are likely causing the appearance of these bands as broad or split.

Upon coordination to Ga(III), the positions and intensities of the N*H* stretching IR bands appeared to change, because metal binding alters the electron density of the thiosemicarbazone framework and can disrupt intra‐ or intermolecular hydrogen bonding. In these metal‐TSC complexes, the ν(NH) bands also shifted slightly to lower wavenumber, typically to around 3150—3300 cm^−^^1^, and in previously reported work, it was apparent that these become less intense upon metal coordination, consistent with increased delocalization within the ligand backbone and reduced hydrogen‐bonding and deprotonation‐accompanied coordination [23, 24, 25, 26, 27, 28, 29]. The solid‐state Raman spectroscopy (783 nm excitation, Figure S19) of **GaL2** and **InL2** was also performed. Despite fluorescence interference in the BODIPY‐containing systems, the Raman‐active metal–ligand modes attributable to Ga–S and Ga–N coordination were still detectable at very similar frequencies (see Supporting Information). This indicated that the local gallium coordination environment in the solid state is essentially identical for the parent **GaL1** and **GaL2**​ complex as unequivocally determined by single crystals analysis (Figure 2c–e) and that the BODIPY‐functionalized analogue **GaL4** (albeit very broad and significantly affected by fluorescence) likely retains the pseudo octahedral geometry around Ga(III) in the solid state. Specifically, for these pseudo‐octahedral Ga(III) thiosemicarbazone complexes, Ga‐S stretching modes are typically observed in the 250–330 cm^−1^ region, while Ga–N stretches appear at higher frequencies, in the 350–450 cm^−1^ range. These modes occur at comparable positions in both systems **GaL1** and **GaL2** and can be seen with a very weak intensity for **GaL3** and **GaL4** suggesting that these systems contain equivalent Ga(S/N/N)_2_]^+​^ coordination environments in the solid state.

This solid‐state equivalence supports our interpretation of the broadened and non‐ideal NMR spectra observed in aqueous DMSO solution where gallium is well known to undergo hydrolysis, ligand exchange, and formation of multiple interconverting hydroxo and solvated species, whereby metal‐F interactions may occur, for example, due to the BODIPY presence or PF_6_^−^ counterions. Complex dynamic equilibria in aqueous media are intrinsic to Ga(III) and In(III) chemistry as well as to thiosemicarbazone ligands, and these are amplified in large, flexible, fluorophore‐conjugated systems such as [**L4**]^−^ that exhibit fluoride groups, inevitably leading to line broadening and loss of small‐molecule‐like spectral resolution in ^1^H NMR. Analogous behavior was observed in the indium series, and a proposed decomplexation pathway with rapid release of Ga(OH)x species in solution is proposed in the Supporting Information. Overall, Raman data indicated that the metal coordination environment is conserved across the series in the solid state, while the observed NMR behavior of **GaL4** and **InL4** arises from solution‐phase dynamic speciation, consistent with established Lewis acidity that dominates the coordination chemistry of Ga(III) and In(III) [23, 24, 25, 26, 27, 28, 29].

### 2.2 Investigations in Solution by UV–Vis and Fluorescence Spectroscopies

**FIGURE 3:**
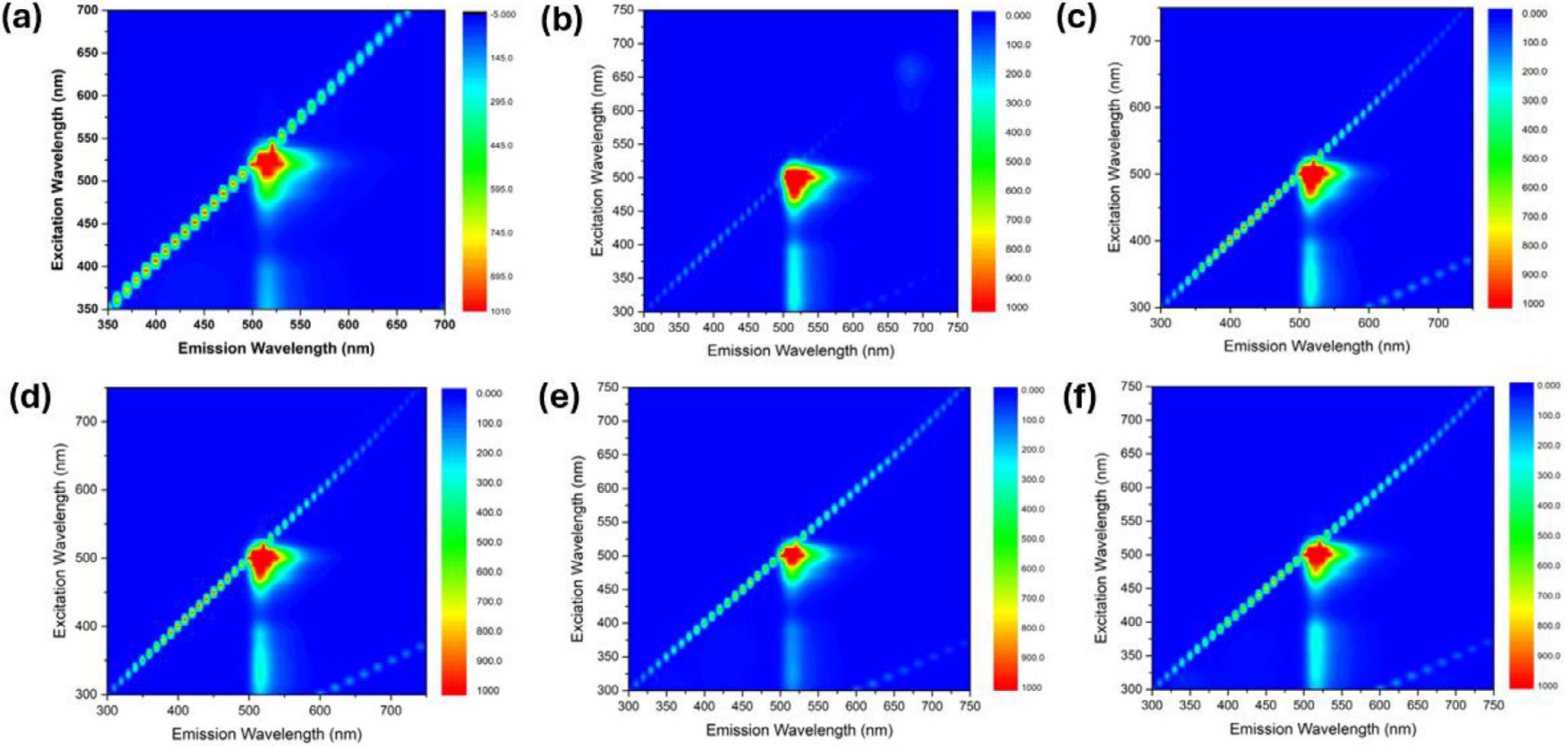
Excitation‐emission maps for establishing the single photon fluorescence profiles of Compounds **HL3** (a), **HL4** (b), **GaL3** (c) and **GaL4** (d), **FeL3** (e) and **FeL4** (f). Measurements were performed in DMSO at 750 nm concentrations.

Photophysical characterization of the BODIPY‐tagged ligands and complexes revealed broadly similar behavior in organic solution, dominated by the BODIPY chromophore. Excitation–emission spectra were collected in DMSO at 750 nm over the 300–750 nm range. Across the series, excitation between 450 and 540 nm produced emission from 480–600 nm, with the most intense excitation observed at 490–510 nm and maximal emission at 510–530 nm. Excitation at 300–400 nm resulted in much weaker emission (500–530 nm), attributable to minor contributions from the pyrimidine and quinoline groups present in the ligand frameworks (Figure 3).

The paramagnetic Fe(III) complexes (**FeL3**, **FeL4**) exhibited pronounced fluorescence quenching relative to the diamagnetic Ga(III) and In(III) analogues, while indium complexes generally showed enhanced emission across the series. Variations in the linker or heterocycle had negligible impact on excitation–emission profiles, indicating that confocal imaging using a 488 nm laser would yield strong emission in the 500–550 nm window. Representative excitation‐emission maps (Figure 3e–j) confirm that the photophysical response is dominated by the BODIPY tag. The fluorescence spectra of **HL3** and **HL4**, as well as their Ga(III) and In(III) complexes, closely resemble those of BODIPY‐COOH and BODIPY‐NHS, displaying strong emission at 511 nm upon excitation at 488 nm.

Photophysical data (Table 1) in DMSO, a solvent widely used in life‐sciences assays reveal consistent trends. BODIPY precursors (BODIPY‐COOH, BODIPY‐NHS, BODIPY‐En) showed typical excitation at 450–540 nm and emission at 480–600 nm, with quantum yields (*ΦF*) ranging from 0.28 to 0.45. Within the TSC–BODIPY series, quinoline‐based ligands were more emissive than pyrimidine analogues. Metal coordination generally reduced ΦF, particularly for Fe(III) complexes, while Ga(III) and In(III) derivatives retained moderate emission. Notably, **InL3** exhibited a slightly higher *ΦF* (0.34) than **GaL3** (0.27), consistent with enhanced fluorescence for the heavier indium complexes. Overall, complexation resulted in modest changes in intensity and emission wavelength (≤15%), while absorption maxima remained largely unchanged (503–505 nm), and the fluorescence lifetime of all BODIPY ligands and complexes is within the ranges expected for this unit, at ca. 3.5 to 3.7 ns in DMSO (under 2‐photon excitation 910 nm, see Figures S40–S45).

**TABLE 1:** Photophysical data for BODIPY‐based compounds discussed hereby. Concentration: 500 nm in DMSO. Relevant spectra in DSO and DMSO: PBS 1: 1 v/v mixtures are given in Supporting Information.

Compound	*λ*_max‐abs_ (nm)	*λ*_max‐em_ (nm)	Δ*λ* (nm)	*Φ*_F_ DMSO (DMSO: PBS)	*ε* DMSO (L mol^−1^ cm^−1^)
BODIPY‐COOH	503	510	7	0.64 (0.16)	88 460
BODIPY‐NHS	505	512	7	0.31 (0.19)	82 320
BODIPY‐En	503	512	9	0.34 (0.02)	89 650
HL3	503	515	12	0.43 (0.07)	81 000
HL4	503	514	11	0.48 (0.39)	80 090
GaL3	503	514	11	0.27	120 560
GaL4	503	514	11	0.38	121 000
InL3	503	516	13	0.34	118 230
InL4	503	514	11	0.26	119 000
FeL3	503	513	10	0.12	100 210
FeL4	503	515	12	0.20	110 880

*N.B*. Referenced against fluorescein in 0.1 m NaOH, *Φ *= 0.95. All *Φ*‐values are corrected for changes in refractive index. Estimated error based on three independent measurements = ±15%. *ΦF*‐values estimated in MeOH at 500 nm are given in Table S1.

Quantum yields were initially evaluated in DMSO relative to a 0.1 m fluorescein standard in aqueous NaOH. BODIPY‐COOH displayed a *ΦF* of 0.64, decreasing to 0.34 for ethylenediamine‐substituted BODIPY and remaining at ca. 0.43 for **HL3** and 0.48 for **HL4**. Upon Ga(III) and In(III) coordination, *ΦF*‐values decreased slightly, remaining between 0.26 and 0.38 across the **GaL3‐GaL4** and **InL3‐InL4** series, and decrease significantly for the paramagnetic Fe(III) analogues. Metal complexes derived from **HL1‐HL2** all exhibited *ΦF*‐values below 0.05%, although **GaL2** and **InL2** showed weak fluorescence in DMSO at concentrations of ca. 50–100 µm in agreement with previous reports on metallo‐thiosemicarbazone systems relevant to theranostics [45].

Structural effects are evident: while BODIPY‐COOH retained a *ΦF* of 0.45 in MeOH and 0.64 in DMSO, conversion to BODIPY‐NHS reduced *ΦF t*o 0.31 in DMSO and 0.28 in MeOH. These values significantly diminished in DMSO: PBS 1:1 mixtures. The ligand frameworks of **HL3** and **HL4** incorporate polar functionalities (e.g., NH groups) while metal(III) coordination was employed to generate lipophilic cationic species capable of cellular uptake. Although the presence of the ethylenediamine linker improved solubility in aqueous media, it reduced *ΦF* due to increased conformational flexibility and hydrogen‐bonding interactions. The combined presence of aromatic TSC units (π–π stacking, hydrogen bonding) across the series and additionally the presence of Lewis acidic metal ions prone to hydrolysis, for example, through Ga─OH formation [23, 24, 25, 26, 27, 28, 29, 30, 31, 32, 33, 34, 35, 36], complicates the estimation of *ΦF* determination in aqueous media. Measurements at 500 nm, in DMSO: PBS (1:1, pH 7.4) showed a significant reduction in *ΦF* for all BODIPY‐based metal complexes investigated (with estimated values ranging from 5%–8% only) due to absorbance broadening and fluorescence quenching: the absorption in aqueous‐containing solvents (e.g., DMSO: PBS 1:1 and 1:3 v/v ratios) increased slightly and was red‐shifted by ca. 2 nm, while fluorescence emission was quenched by up to 50%. Whilst the maximum emission intensities of the free BODIPY‐based ligands **HL3** and **HL4** remained largely unaffected in DMSO: PBS relative to pure DMSO their overall quantum yield was diminished. These effects are attributed to aggregation‐caused quenching with J‐aggregate formation supported by crystallographic data of this class of compounds. Importantly, the emission maxima changes were modest within 30 min (<15% relative to DMSO) and are unlikely to impact performance in biological media on the timescale investigated, however they introduce inaccuracies in the determination of *ΦF* in DMSO: PBS for **HL3‐HL4** and corresponding metal complexes. Therefore in all cellular assays, PBS was excluded, as even low levels (e.g., 1%–5% PBS in RPMI) produced detectable autofluorescence in control FLIM experiments (Figure S98).

Despite challenges associated with limited water solubility, which in turn cause the necessity to add of 0.1%–1% DMSO to solutions of metal complexes intended for cellular delivery assays, the metal–BODIPY conjugates, including the paramagnetic Fe(III) complexes, maintained sufficient fluorescence in aqueous environments to render these traceable by confocal fluorescence and FLIM (Figures S40–S44), to render these traceable in cells, supporting their suitability for MP‐FLIM and correlated confocal imaging. The kinetic stabilities of all compounds were evaluated in a range of solutions relevant for cell testing: stability studies in 10% RPMI serum media (SM) with 5% DMSO over 24 h revealed minimal spectral changes. Overall, most compounds showed less than 15% change in these spectra over 24 h monitoring in this media, suggesting that the complexes are kinetically stable under conditions relevant to cellular investigations.

### 2.3 Cellular Viability and Confocal Fluorescence Microscopy Imaging in Living Cells

To evaluate the biological activity in living cells, at first, MTT assays of free ligands **HL1–HL4** and their Ga(III), In(III), and Fe(III) complexes were performed and results showed reduced proliferation of PC‐3 and HeLa cells across the series. These cell lines were chosen because they overexpress Type II topoisomerases which are essential enzymes for DNA replication, transcription and chromosome segregation. Results for the extensive MTT assays carried out in HeLa, PC‐3 and a typical healthy cell line (AG09429, healthy controls, human gingival fibroblast) are given in Figures S46–S71 and Tables S2–S6).

Overall, MTT assays over 48 h observations and in selected cases, over 72 h observations, showed BODIPY‐labelled ligands and complexes were less active in PC‐3 cells than their non‐BODIPY counterparts and relevant controls (Figures S63–S65), though several still displayed measurable potencies over 24–48 h and therefore exhibit theranostic potential. The linker attaching BODIPY had minimal influence, and heteroaromatic variation within the TSC scaffold produced only modest differences. Metal coordination restored or enhanced activity across the series: **FeL3** and **FeL4** were weakly active at 24 h but more potent at 48 h, with **FeL4** nearly fivefold stronger than **FeL3** (7.10 ± 1.72 µm vs. 36.64 ± 2.62 µm).

Although some complexes remained weakly cytotoxic, several exhibited promising activities (Table 2, Figures S46–S71, and Tables S2–S6) and would warrant further evaluation, including selectivity studies, in future research. As necessary controls, IC_50_ values for free Ga(NO_3_)_3_, Triapine, free ATSM ligand, and commercially available clinically established metallodrugs (cisplatin, nedaplatin, oxaliplatin) were measured under previously established conditions [23, 24, 25, 26, 27, 28, 29, 30, 31, 32, 33, 34, 35, 36]. As expected, complexes derived from **HL1** and **HL2** (which do not incorporate BODIPY units) displayed low‐micromolar cytotoxicity against PC‐3 cells over 24–72 h, enhanced upon metal coordination (Table 2). Across the series, Ga(III) and In(III) coordination markedly increased activity relative to the free ligands, with **GaL2** and **InL1–InL2** being the most potent (sub‐micromolar level). **HL3** and **HL4** were essentially inactive, yet their Ga(III) and Fe(III) complexes displayed micromolar potency, demonstrating that metal coordination is the principal driver of biological activity.

**TABLE 2:** IC_50_ values estimated in PC‐3 (human prostate cancer cells) following incubation with compound for 48 h under standard MTT assays. *N.B*. Data presented as mean ± SD, *n* = 3; SD: Standard deviation; ns: No significant cytotoxicity compared with the control group (*p* > 0.05). Data of some treatment groups could not be fitted with a good dose‐response curve and the exact IC50 values could not be calculated; their IC50 values were expressed as concentration ranges and labelled with “#”. Extensive controls with commercial Triapine, cisplatin, oxaliplatin and nedaplatin as well as with free Ga(III) as Ga(NO_3_)_3_ and additional MTT‐estimated IC50 over 24 and 72 h assays are given in Figures S63–S65.

Compound	48 h IC_50_ (µm) PC‐3
Triapine	0.16 ± 044
Cisplatin	30.65 ± 3.25
HL1	3.36 ± 0.31
HL2	25.6 ± 7.89
HL3	>250^#^
HL4	>100^#^
GaL1	9.34 ± 0.35
GaL2	0.68 ± 0.02
GaL3	3.32 ± 0.84
GaL4	3.44 ± 0.53
InL1	0.82 ± 0.14
InL2	1.06 ± 0.28
InL3	9.42 ± 0.05
InL4	1.01 ± 0.19
FeL3	36.6 ± 2.62
FeL4	7.10 ± 1.72

MTT assays under a range of conditions over 24 and 48 h assays showed that after functionalization of the thiosemicarbazone with BODIPY tags, their activity toward both PC3 and HeLa cells decreases. This suggested that the inclusion of the BODIPY influences the cellular behavior of the compounds TSC backbone. This trend is also seen upon complexation to In(III) and Ga(III), with all BODIPY‐labelled complexes showing less inhibitory mitochondrial activity through MTT assays than their unlabeled analogues. The mechanistic understanding of the cytotoxic activity of gallium(III)‐based complexes against cancer cells that respond to platinum‐based chemotherapeutics is a matter of extended investigations [23, 24, 25, 26, 27, 28, 29, 30, 31, 32, 33, 34, 35, 36].

To directly trace these Triapine‐inspired and BODIPY‐conjugated metal complexes in living cells, laser confocal microscopy was employed to study cellular uptake and localization of their BODIPY fluorophores. PC‐3 and HeLa cells were incubated with ligands and complexes for 15–30 min prior to imaging assays, using 405, 488, and 561 nm excitation. IC_50_ values from 1 h MTT assays guided the choice of non‐toxic working concentrations, with cells treated with BODIPY‐tagged compounds at concentrations between 100 nm and 10 µm (1% DMSO). Free pyrimidine derivatives **HL1** and their Ga or In complexes were too weakly fluorescent to be visualized at these concentrations, whereas **HL2** and corresponding metal complexes required >50 µm for detection, which in turn compromised cell viability. Attaching BODIPY therefore enabled visualization of thiosemicarbazones in vitro. Multiwavelength excitation was used to stain with Hoechst or DAPI (*λ*_ex_ 405 nm), Mitotracker Red CMXRos (*λ*_ex_ = 543 nm) and Lysotracker Red DND‐99, (*λ*_ex_ = 543 nm) and ER tracker Red (ex 543 nm). Selected microscopy imaging micrographs including for colocalization assays are given in Figures S72–S95.

**FIGURE 4:**
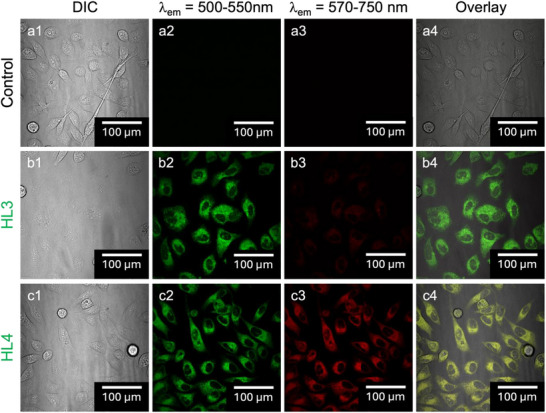
Confocal laser‐scanning microscopy images of PC‐3 cells (human prostate cancer) incubated with compounds at 500 nm concentration for 20 min, where (a1–a4) control group with 1% DMSO, (b1–b4) **HL3**, 1% DMSO, (c1–c4) **HL4**, 1% DMSO. a1–c1 is the DIC channel; a2–c2 is the emission detected at *λ* = 500–550 nm; a3–c3 is the emission detected at *λ* = 570–750 nm, excitation laser at *λ*_ex_ = 488 nm; a4–c4 is the merged image. Scale bar: 100 µm.

**FIGURE 5:**
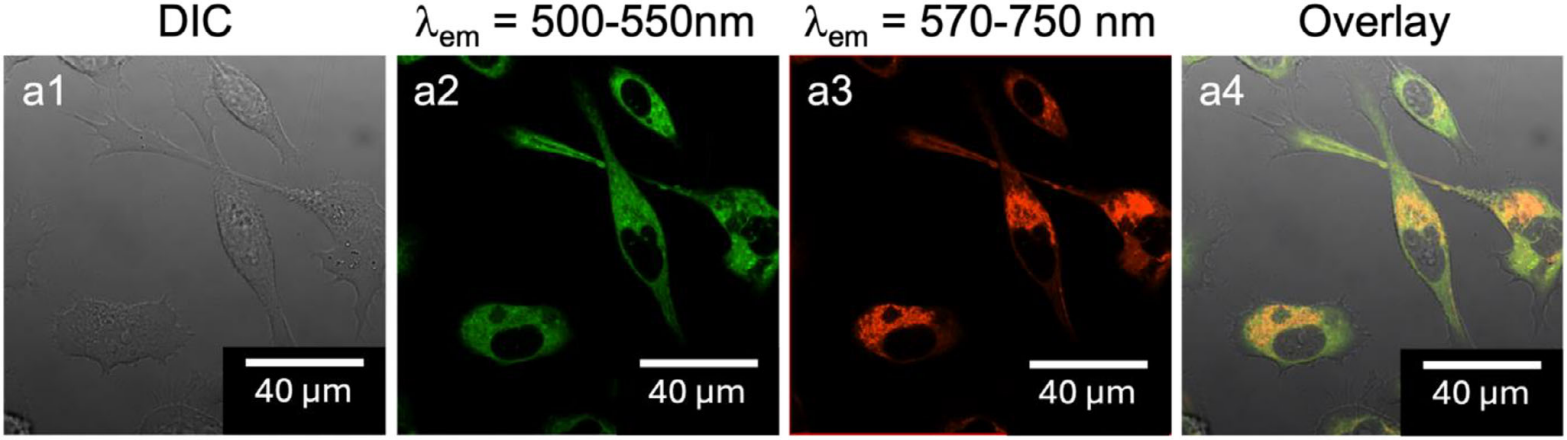
Confocal laser‐scanning microscopy of PC‐3 cells incubated with compound **GaL4** at 500 nm, 1% DMSO, and co‐incubated with Mitotracker Red CMXRos (500 nm) at 37°C for 20 min incubation, excitation laser at *λ*_ex_ = 488 nm. Scale bar: 40 µm. (Pearson coefficient: 0.815 ± 0.133) Full colocalization Manders parameters are given in Table S7 in the Supporting Information.

**FIGURE 6:**
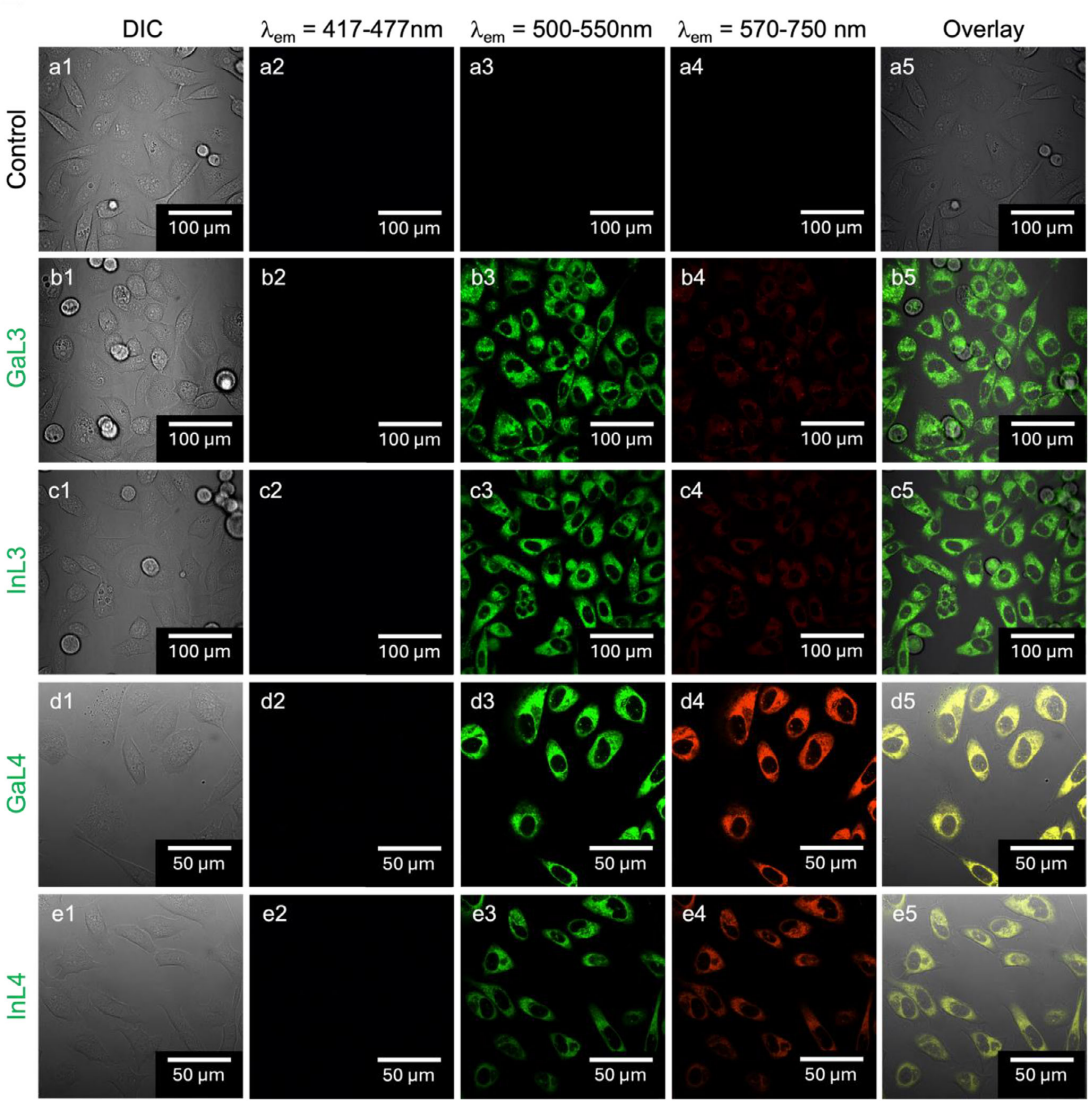
Confocal laser‐scanning microscopy of PC‐3 cells incubated with compounds at 500 nm concentration at 37°C for 20 min, where (a1–a5) control group 1% DMSO, (b1–b5) **GaL3**, 1% DMSO, (c1–c5) **InL3**, 1% DMSO, (d1–d5) **GaL4**, 1% DMSO, (e1–e5) **InL4**, 1% DMSO. (a1–e1) is the DIC channel; (a2–e2) is the emission detected at *λ* = 417–477 nm; (a3–e3) is the emission detected at *λ* = 500–550 nm; (a4–e4) is the emission detected at *λ* = 570–750 nm, excitation laser at *λ*_ex_ = 488 nm; (a5–e5) is the merged image. Scale bar: 100 µm for (a–c) series; 50 µm for (d,e) series.

**FIGURE 7:**
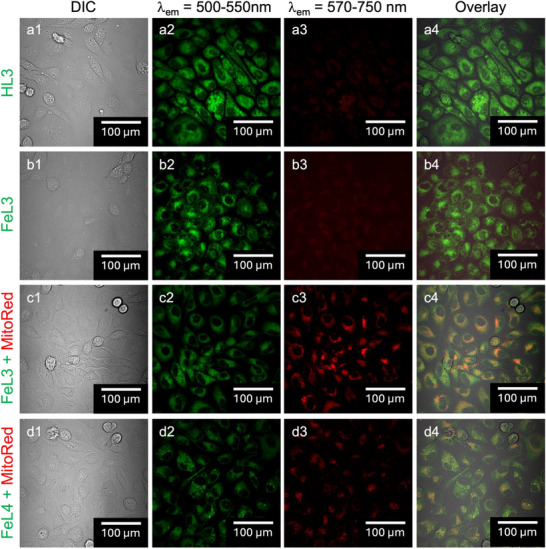
Confocal laser‐scanning microscopy of PC‐3 cells incubated in 1%: 99% DMSO: serum‐free media with compounds at 100 nm concentration at 37°C for 20 min, where (a1–a4) **HL3**, (b1–b4) **FeL3**, (c1–c4) **FeL3**, co‐incubated with Mitotracker Red CMXRos (500 nm), (d1–d4) **FeL4**, co‐incubated with Mitotracker Red CMXRos (500 nm). (a1–d1) is the DIC channel; (a2–d2) is the emission detected at *λ* = 500–550 nm; (a3–d3) is the emission detected at *λ* = 570–750 nm, excitation laser at *λ*_ex_ = 488 nm; (a4–d4) is the merged image. Scale bar: 100 µm.

Whilst the quinoline‐based monothiosemicarbazone **HL2** is only weakly fluorescent, the corresponding Ga(III) and In(III) complexes were still traceable by confocal microscopy, albeit at concentrations above 50 micromolar (Figures S74–S77). For example, complexes of type [M(L2)_2_]PF_6_ **GaL2** and **InL2**, did not colocalize with nuclear Hoechst dye, but showed co‐localization with ER and Mitotracker Red over 20 min. Across all BODIPY‐featuring compounds, confocal microscopy in PC‐3 and HeLa cells demonstrated uptake within 20 min, predominantly localizing to the endoplasmic reticulum. The free ligands **HL3** and **HL4** exhibited rapid uptake with distinct localization patterns throughout the cytoplasm, and emitting strongly in green and red channels (500–550 nm and 570–750 nm) upon 488 nm excitation. Additionally, cytoplasmic localization was observed for Ga‐substituted complexes (**GaL4**), with minimal nuclear uptake, confirmed by co‐localization with ER Red tracker, Mitotracker Red, and Hoechst dyes, and supported by MP‐FLIM. Uptake assays at 4°C suggested active transport consistent with prior BODIPY‐tagged compound studies [46]. Some representative images for the uptake of M(III) complexes of **HL3** and **HL4** in living PC‐3 cells are shown in Figures 4, 5, 6, 7. The Manders colocalization parameters are given in Table S7. Overall, the distribution of **HL3** and **HL4** within the cell is in line with previously reported compounds featuring BODIPY tags, with behavior in cells generally being characterized by ER colocalization and distribution dominated by this moiety [46]. However, the compounds **GaL4** and **FeL4** showed additional and distinct localization in cellular mitochondria (Figure 5), unlike corresponding free ligands and the other metal complexes analyzed hereby (Figure 4, 5, 6, 7)

Interestingly, Fe(III)–BODIPY conjugates **FeL3** and **FeL4** showed similar, consistent but weaker staining relative to their respective Ga analogues **GaL3** and **GaL4**, with ligand‐dependent behavior. Gallium(III) closely mimics iron(III) in charge, ionic radius and hard/soft ligand‐binding preferences, particularly toward N/S donors. Its strong similarity underlies its high‐affinity binding to endogenous iron‐transport and storage proteins such as transferrin and siderophores. However, unlike Fe^3^^+^, Ga^3^^+^ is redox‐inactive and cannot participate in the Fe^3^^+^/Fe^2^^+^ cycling required for mitochondrial electron transfer and broader iron metabolism. Quinoline‐substituted TSCs [23, 24, 25, 26] can support ligand‐centered redox chemistry, and previous reports showed that Ga–TSC conjugates undergo such processes within biologically relevant windows. Free Ga^3^^+^ itself disrupts iron metabolism by displacing Fe^3^^+^ from high‐affinity sites including FeS‐cluster precursors, mitochondrial siderophore‐like molecules and other Fe‐dependent cofactors [23, 24, 25, 26, 27, 28, 29, 30, 31, 32, 33, 34, 35, 36]. We postulate that when these gallium thiosemicarbazones (Ga‐TSC) localize to mitochondria, an organelle enriched in iron (featuring ∼15% of the 29–32 µm cellular pool), redox‐active proteins, and dense metal‐trafficking machinery, encounter conditions favoring Ga^3^^+^/Fe^3^^+^ exchange. This transmetallation may produce a localized functional iron deficiency, for example, total Fe remains unchanged, but bioavailable Fe decreases. Considering that mitochondria supply iron for FeS‐cluster and haem biosynthesis, while also exporting Fe needed by cytosolic enzymes such as ribonucleotide reductase (RNR), disruption of mitochondrial Fe handling can impair DNA synthesis. RNR relies on a dinuclear Fe center to generate the tyrosyl radical required for deoxyribonucleotide formation; interference with mitochondrial Fe trafficking therefore limits assembly of the RNR metallocentre.

These mitochondria‐localizing Ga‐TSC may be therefore well‐positioned to suppress cell proliferation by displacing Fe^3^^+^, perturbing mitochondrial iron homeostasis and restricting cytosolic Fe delivery to RNR. These features align with the broader behavior as a functional iron antagonist well established for Ga(III). The Pearson's coefficient was also estimated indicating mitochondria colocalization for **GaL4** 0.815 ± 0.132, **InL4** 0.82 ± 0.029 and **FeL4** 0.68 ± 0.05; ER colocalization for **GaL4** 0.92 ± 0.008, **InL4** 0.92 ± 0.014 and **FeL4** 0.94 ± 0.02. Full Manders’ colocalization parameters are given in Table 3, Table S7, and Figure S95 in Supporting Information.

**TABLE 3:** Overview of the Manders’ correlation parameters of selected BODIPY conjugates extrapolated by scatterplot analysis using the software Nikon Elements‐AR Analysis 4.30.02.

Compound	Endoplasmic reticulum (ER)	Mitochondria	Lysosome
HL3	0.489 ± 0.035	0.497 ± 0.047	0.629 ± 0.047
HL4	0.934 ± 0.021	0.852 ± 0.030	0.497 ± 0.050
GaL3	0.676 ± 0.045	0.589 ± 0.058	0.470 ± 0.057
InL3	0.539 ± 0.050	0.530 ± 0.043	0.596 ± 0.048
GaL4	0.985 ± 0.010	0.970 ± 0.016	Ns
InL4	0.990 ± 0.010	0.990 ± 0.010	Ns
FeL3	0.923 ± 0.024	0.773 ± 0.038	0.399 ± 0.050
FeL4	0.922 ± 0.018	0.678 ± 0.051	0.556 ± 0.049

### 2.4 Multiphoton Fluorescence Lifetimes (MP FLIM) Measurements in Solution and in Living Cells

Cellular uptake and intracellular distribution were assessed using laser scanning CFM under both single‐ (488 nm) and two‐photon excitation (910 and 1010 nm), taking advantage of the low‐energy, near‐infrared light for deeper tissue penetration and reduced photodamage. Time‐correlated single‐photon counting (TCSPC) and two‐photon FLIM techniques enabled investigation of the probes’ microenvironments, their interactions with mitochondria and ER, and the influence of surrounding cellular components giving rise to distinct lifetime distributions across the series **HL4**, **GaL4** and **InL4**, whilst showcasing the clear dominance of the BODIPY moiety and distinct behavior with respect to control groups (Figures 8, 9 and Table 4, and additional micrographs are given in Figures S96–S114).

**FIGURE 8:**
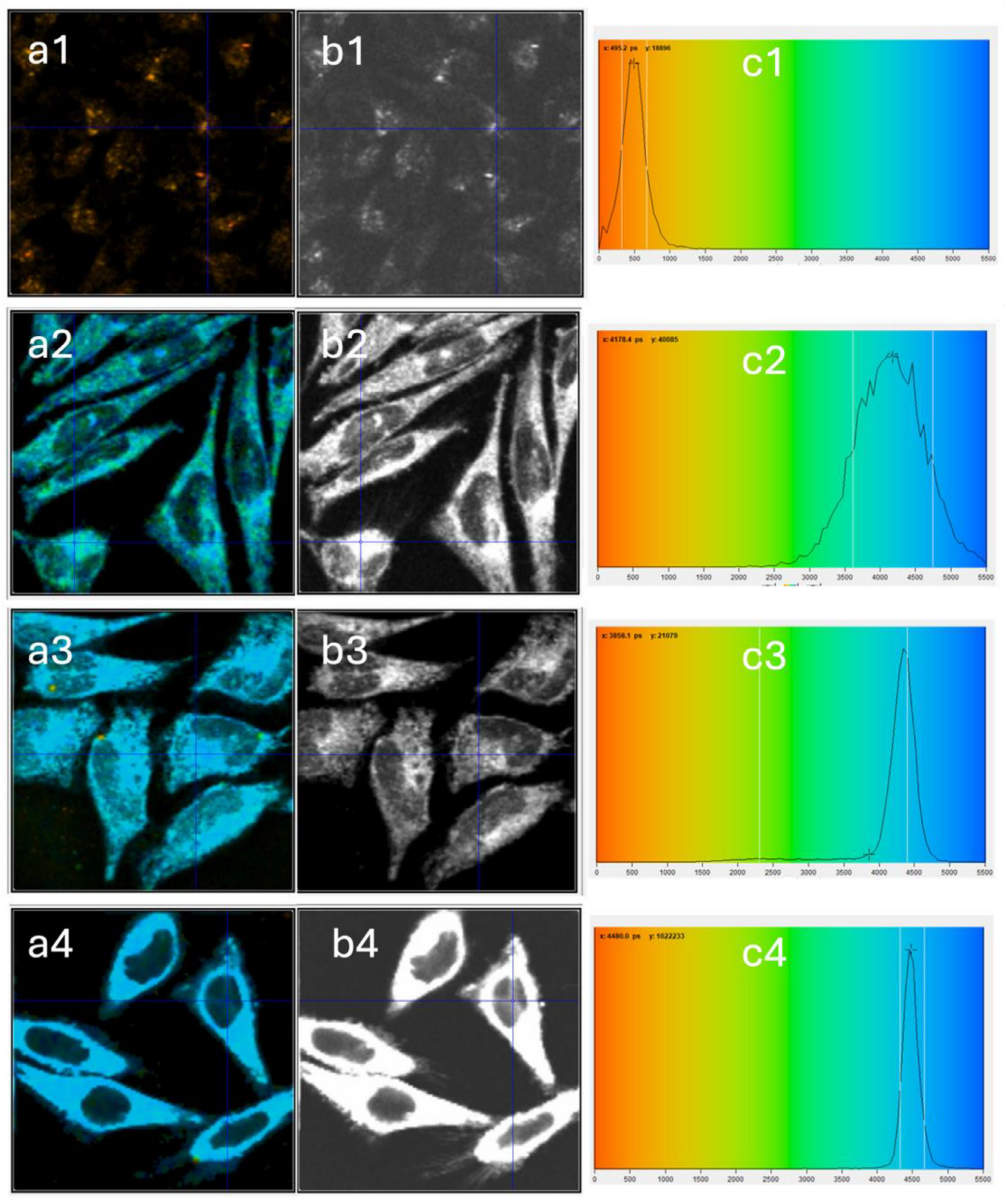
2‐photon FLIM maps, intensity images and in vitro fluorescence lifetime distribution histograms (left to right) of HeLa cells treated with 1 µm of **HL4** (a2–c2) and corresponding **GaL4** (a3–c3) and **InL4** (a4–c4) complexes, where (a1–c1) represents the micrographs for the control group (1% DMSO). Incubation was 20 min, 37°C followed by with 2‐photon excitation laser at *λ*_ex _= 910 nm. Field of view = 100 µm. Alternative micrographics for PC‐3 as well as HeLa cells are given in Figures S96–S114.

**FIGURE 9:**
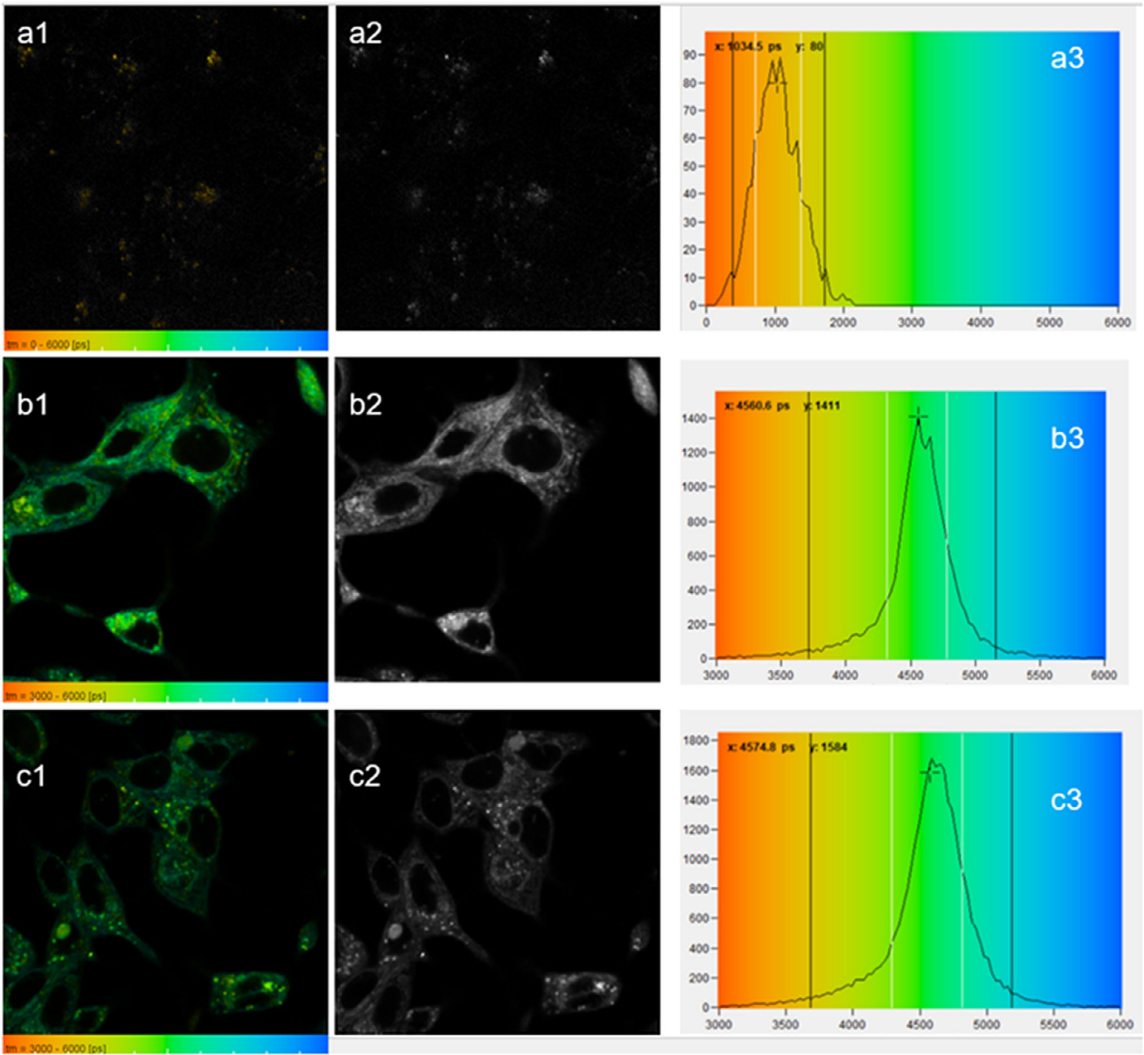
Single‐photon FLIM maps, intensity images and in vitro fluorescence lifetime distribution histograms (left to right) of PC‐3 cells treated with 1 µm of **HL4** (b2–b3) and corresponding **FeL4** (c1–c3) complex, where (a1–a3) represent the micrographs for the control group (1% DMSO). Incubation was 20 min, 37°C followed by excitation at *λ*_ex_ = 488 nm. Field of view = 100 µm. Alternative micrographics and corresponding 2‐photon FLIM imaging with excitation at 1010 nm are given in S102–S103).

**TABLE 4:** Calculated 2P FLIM data from the in vitro fluorescence lifetime distribution histograms of PC‐3 and HeLa cells treated with 1 µm of **HL3, HL4** and corresponding metal complexes, incubation for 15–20 min and control group (1% DMSO), at 37°C with multiphoton excitation at 910 nm (corresponding to Figure 8) or 1010 nm (corresponding to Figure 9). Data presented as peak ± error (ns). The full width at half maximum (FWHM), calculated from the lifetime distribution curve within the focal area was used to assess the fluorescence lifetime distribution error. The weakly fluorescent compound **InL2** was measured in Hela cells under similar conditions, 2P average lifetime distribution had a maximum of 0.34 ns ± 0.17, versus TCSPC (in 100 µm conc. and 1% DMSO) with parameters: T1 0.21 ns (87.35%) and t2 0.95 ns (12.65%), *χ* ^2 ^= 1.3. Additional 2P and 1P FLIM data, solution TCSPC in DMSO and micrographs are given in Figures S96–S114.

Treatment group	2P‐fluorescence lifetime (ns)
Control PC‐3 (1% DMSO)	1.08 ± 0.39
Control HeLa (1% DMSO)	0.49 ± 0.18
**HL3** (PC‐3)	4.56 ± 0.16
**HL3** (HeLa)	4 ± 0.57
**HL4** (HeLa)	4.18 ± 0.57
**HL4** (PC‐3)	4.56 ± 0.16
**GaL4** (HeLa)	3.86 ± 0.11
**GaL4** (PC‐3)	4.40 ± 0.17
**InL4** (HeLa)	4.48 ± 0.17
**InL4** (PC‐3)	4.41 ± 0.18
**FeL4** (PC‐3)	4.59 ± 0.20

For the DMSO solution spectra (determined by TCSPC spectroscopy of **InL2** as well as BODIPY conjugates (Figure S45a–h,S106) and corresponding, correlated, cellular FLIM studies, 2‐photon excitation at 910 or 1010 nm was used as it consistently gave better signal to background than single‐photon excitation, however a comparison with 1P FLIM imaging at 488 nm excitation was also carried out (Figure 8, 9, Table 4, and Figures S96–S114). Details of the experimental 2P FLIM set‐up for confocal‐FLIM correlative imaging used are as previously reported [49] and are given in the Experimental Section and Supporting Information. Cell uptake studies using FLIM were performed on adherent HeLa or PC‐3 cells covered with a glass cover slip and the background lifetimes measured. The ligands or complexes were dissolved in a small amount of DMSO and added to the cell culture medium to give concentrations of 1 to 10 µm and a maximum 1% DMSO. Decay lifetimes were measured for the complete field of view over five‐minute intervals. Uptake reached a maximum within 30 min, and the images and data presented below were obtained after 20 min. Exponential decay and weighted least squares analysis of the intensity data to extract rate constants were carried out using SPCImage analysis (SPCM64, version 9.0, Becker and Hickl, Germany) or F900 Edinburgh Instruments TCSPC analysis software. Throughout the panel of probes investigated, the lifetime parameters are different for the intact complex and free ligand, enabling these to be distinguished in living cells (Table 3). Significantly, the results obtained by MP FLIM for the M(III) derivatives have given a remarkable insight into their intracellular localization, which appears to be primarily mitochondrial in nature for **GaL4** and **FeL4**, whereas the localization of all other BODIPY‐tagged derivatives rests firmly within the ER.

The lifetime distribution maps were obtained for the ligand based on the single decay, whereas for the metal complexes maps based on both lifetimes’ decays. The partial intracellular dissociation of the indium (bis) tridentate species into a mixture of free ligand and bis complexes was also indicated by FLIM, based upon the relative lifetimes of each of the species, which indicates the decomposition of the complexes and where it occurs in cells. Lifetime point decays were measured in various locations within the cell and corresponding lifetimes calculated. In the situation where both ligand and metal complex were present, there could in principle be at least two different decays; the calculation of three or more closely spaced lifetimes is notoriously difficult and requires both very high‐quality data sets and additional data obtained using phase modulation [50].

Overall, the data analysis was sufficient to determine the intracellular speciation of both the ligands and their metal complexes as evidenced from Figures 8 and 9, allowing the study to progress toward in vivo validation. Although some variation in fluorescence lifetimes and subtle changes in biodistribution were observed for the gallium and iron complexes **GaL4** and **FeL4**, suggesting that partial dissociation may occur, the differences between the free ligand and the complex were not sufficiently large to quantify the extent of dissociation unambiguously.

Organelle targeting with lipophilic cations and small molecule conjugates has been of utmost interest, especially from the perspective of mitochondria targeting technology. Recent reports have highlighted that highly lipophilic small‐molecule probes may lose organelle specificity and distribute broadly throughout the cell, including the nucleus, with minimal selective accumulation [49]. While this paradigm is important to consider, its applicability to the BODIPY‐labelled metal–thiosemicarbazone (TSC) constructs studied here is challenging. Unlike simple organic fluorophores, these systems are large, charged, and undergo dynamic speciation in aqueous and intracellular environments, making a single log *p*‐value an incomplete descriptor of their behavior. Metal‐TSC complexes particularly those of Lewis acids such as Ga(III) can interconvert between neutral and ionic forms and may partially dissociate, generating multiple species with distinct permeabilities and binding profiles [27, 28, 29]. Moreover, their subcellular localization is influenced not only by lipophilicity but also by metal–ion coordination preferences, pH‐dependent ligand exchange, redox properties, and interactions with biomacromolecules. Although log *p*‐values which were estimated here from the SwissADME programme can provide useful additional information (see Table 4 and Experimental Section), they cannot act as predictors of intracellular distribution for these lipophilic cations which are also dynamically equilibrating metal complexes. Consequently, techniques sensitive to the chemical state of the fluorophore such as multiphoton FLIM (vide infra) can become essential tools for probing the speciation and localization of these constructs in living cells.

### 2.5 In Vivo Tests in Zebrafish

To determine whether these compounds can be internalized and tracked in vivo, we performed uptake and imaging experiments on zebrafish, selected for its strengths as a biomedical model for compound screening and uniquely suited for the required high‐resolution in vivo imaging due to its optical transparency. Initially, we focused on identifying the optimal concentration and timing for compound uptake and imaging, as well as assessing zebrafish larvae's tolerance to the compounds. Utilizing fluorescent stereomicroscopy revealed that the **HL1‐HL2** compounds and complexes thereof all demonstrate minimal fluorescence in vivo even at 200 mm; moreover, these concentrations were poorly tolerated, therefore ruling these simple thiosemicarbazone compounds out from further direct in vivo optical imaging investigations by this method (Figure S115). For the **HL4** ligand, the optimal conditions for uptake of the compounds were denoted by larval viability as well as fluorescence detection primarily in digestive organs, for example, the gut bulb and intestines. The latter was assessed by acquiring images using a stereoscope and identified as being 10 µm for 120 mins at 28.5°C.

**FIGURE 10:**
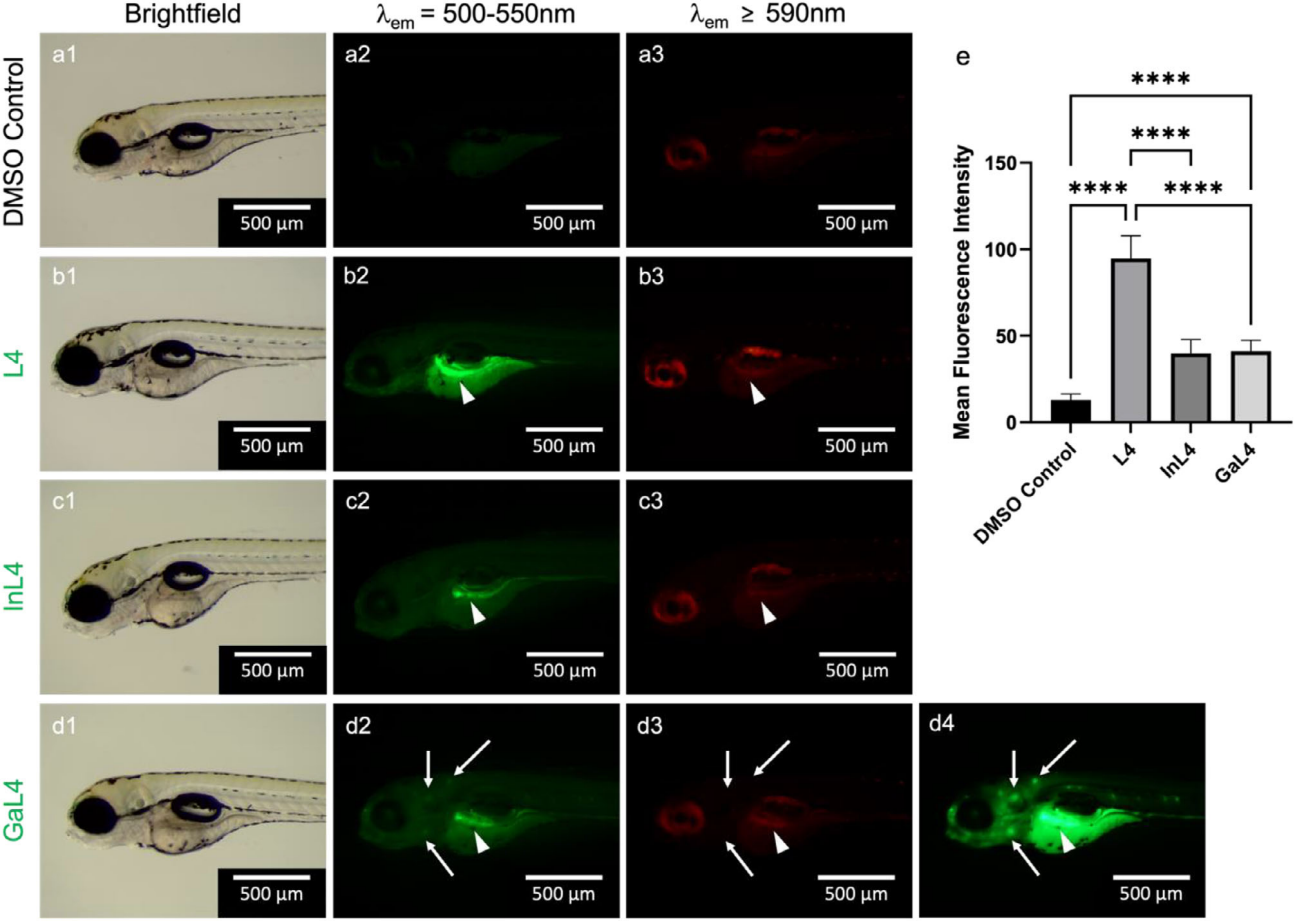
Representative brightfield and fluorescence stereomicroscope images of 4 dpf zebrafish larvae incubated with compounds at 10 µm for 120 min at 28.5°C, arrowheads indicate labelled digestive system, arrows indicate labelled neuromasts, where (a) control group in embryo media and 1% DMSO, (b) **HL4**, 1% DMSO, (c) **InL4**, 1% DMSO, (d) **GaL4**, 1% DMSO. (e) mean fluorescence intensity for the digestive organs of a2–d2, calculated from eight treated larval fish per condition, error bar stands for the standard deviation. a1–d1 is the brightfield channel; a2–d2 is the emission detected at *λ* = 500–550 nm, excitation at *λ*_ex_ = 450–490 nm; a3–d3 is the emission detected at *λ* ≥ 590 nm, excitation at *λ*_ex_ = 540–580 nm; d4 is an enhanced image of d2, taken at exposure (100 ms^−1^) and gain (2.40), whereas all other fluorescence images taken at exposure (100 ms^−1^) and gain (1). Scale bar: 500 µm. Significant difference determined via Brown–Forsythe and Welch ANOVA testing and confirmed through post hoc Dunnett's multiple comparison. (*) <0.05, (**) <0.010, (***) <0.001 and (****) < 0.0001.

**FIGURE 11:**
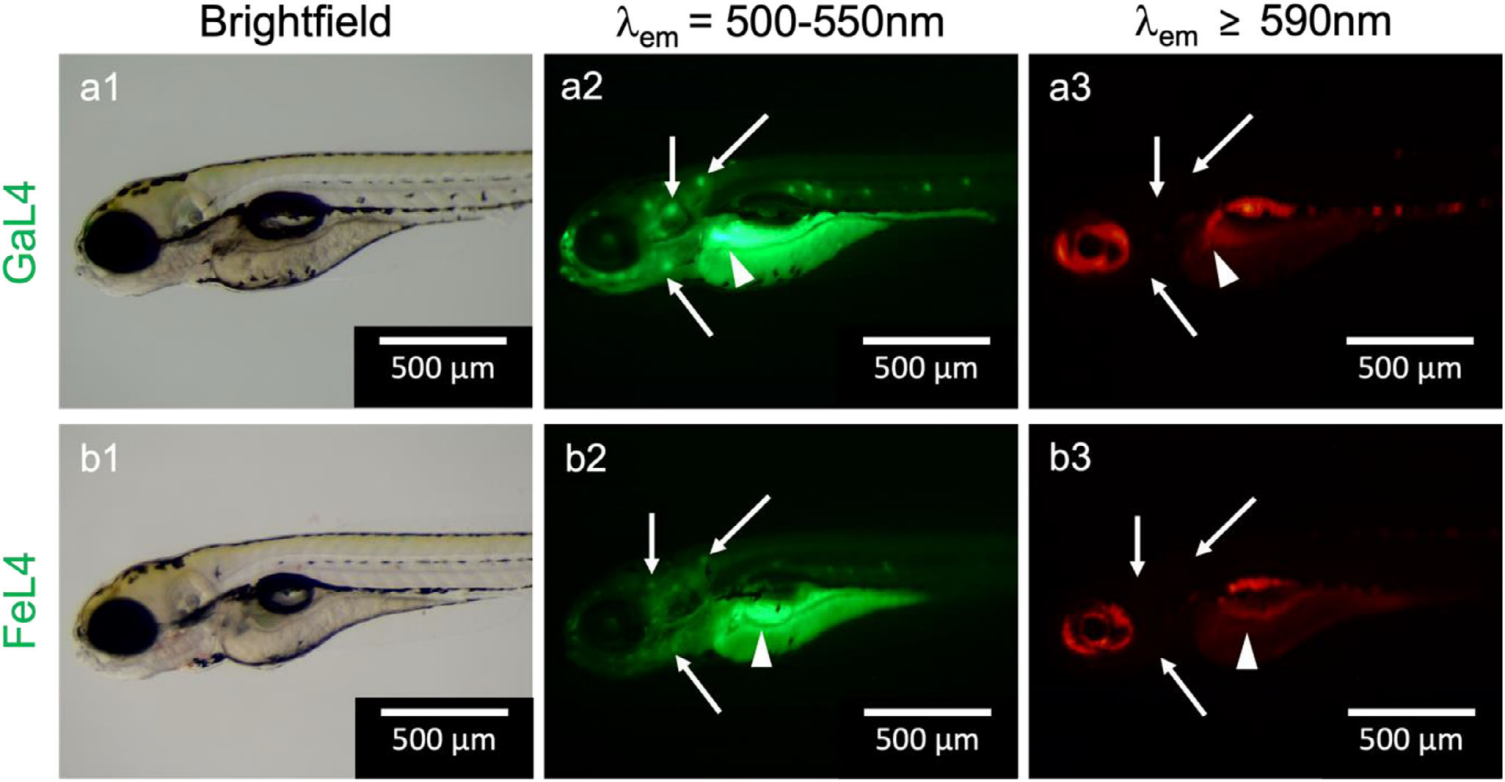
Representative brightfield and fluorescence stereomicroscope images of 4 dpf zebrafish larvae incubated with compounds at 10 µm for 120 min at 28.5°C, arrowheads indicate labelled digestive system, arrows indicate labelled neuromasts, where (a) **GaL4**, 1% DMSO, (b) **FeL4**, 1% DMSO. a1–b1 is the brightfield channel; a2–b2 is the emission detected at *λ* = 500–550 nm, excitation at *λ*_ex_ = 450–490 nm; a3–b3 is the emission detected at *λ* ≥€590 nm, excitation at *λ*_ex _= 540–580 nm. a2 and b2 are taken at exposure (100 ms^−1^) and gain (2.40), whereas all other fluorescence images taken at exposure (100 ms^−1^) and gain (1). Scale bar: 500 µm.

**FIGURE 12:**
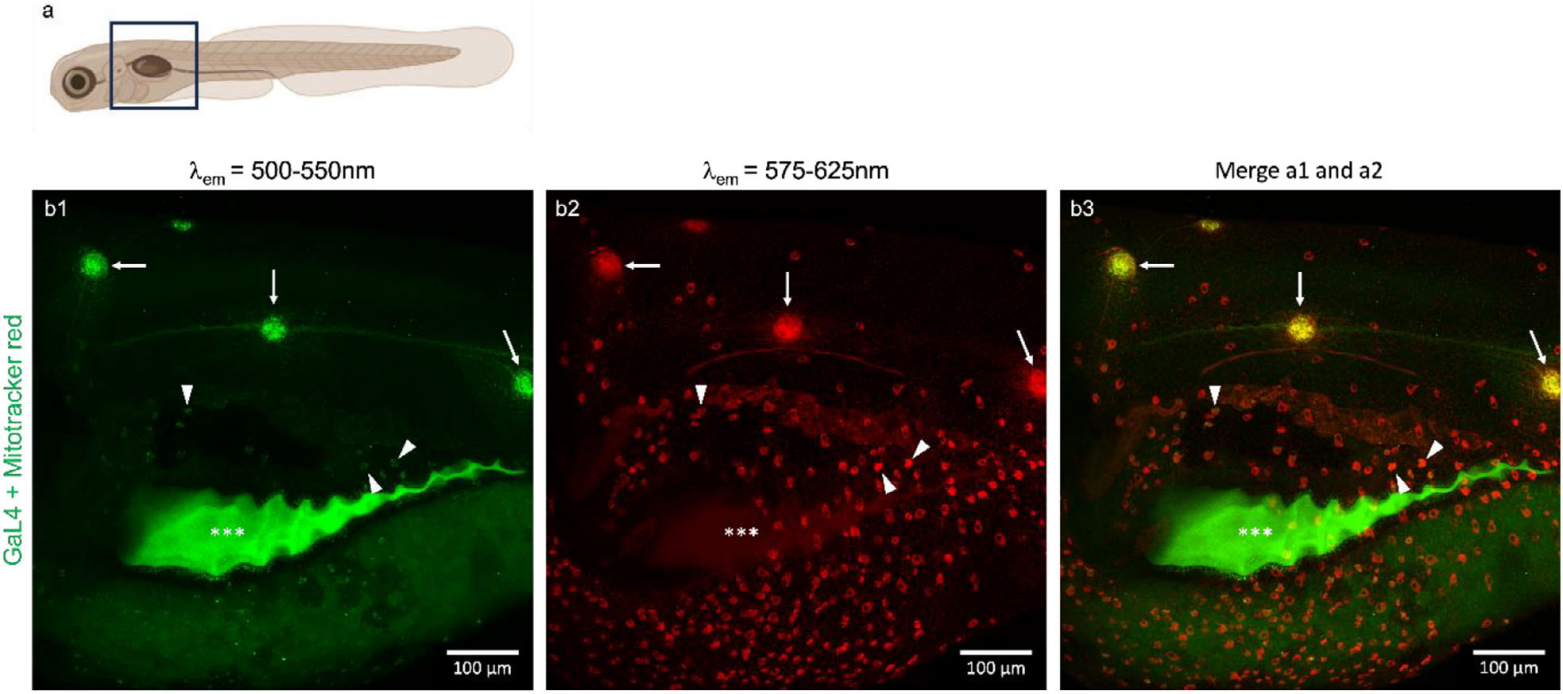
(a) Schematic demonstrating the area of imaging. (b) Representative confocal laser‐scanning microscope images of 4 dpf zebrafish larvae co‐incubated with **GaL4** at 10 µm and Mitotracker Red CMXRos at 500 nm, 1% DMSO, for 120 min at 28.5°C, arrows indicate co‐labelled neuromasts, arrow heads indicate co‐labelled mitochondria, asterisk indicates the labelled digestive system. b1 is the emission detected at *λ* = 500–550 nm, compounds were excited at *λ*_ex_ = 488 nm; b2 is emission detected at *λ* = 575–625 nm, compounds were excited at *λ*_ex_ = 561 nm; b3 is the merged image of b1 and b2. Scale bar: 100 µm.

Once optimized, zebrafish larvae at four days post fertilization (4 dpf) were further incubated with compounds **HL4**, and corresponding complexes **InL4, GaL4**, and **FeL4** in DMSO (Figures 10 and 11). Comparatively, **HL4** was significantly brighter than either of the metal‐containing **HL4** compounds and demonstrated non‐specific staining of the yolk as well as staining of digestive organs. **GaL4** trended as having slightly brighter intensity in vivo by comparison to **InL4** and demonstrated moderate staining of mitochondria, especially in neuromasts, that was largely absent in **HL4** or **InL4**‐stained fish (Figure 10; Figure S116). Showing conservation with our earlier cell culture results, **GaL4** treatment revealed a very similar staining pattern to **FeL4** treatment, with the primary difference the comparatively much brighter staining for the **GaL4** compound (Figure 11). Focusing on the uptake of **GaL4**, we co‐incubated with Mitotracker Red CMXRos at 500 nm and observed strong co‐labelling of neuromasts together with co‐labelling of some mitochondria throughout the trunk of the fish (Figure 12), in line with our earlier observations of this compound in vitro co‐labelling experiments (Figure 5).

To assess whether the selected ligand would impact on tissue uptake or in vivo fluorescence and localization, we compared **GaL3** to **GaL4** and observed that **GaL4** labels all structures less efficiently than **GaL3**, as well as being poorly tolerated over a 120‐minute treatment window (Figure S117). Consistent with this result, we demonstrated that **FeL3** was also less specific in labelling and very poorly tolerated by comparison to **FeL4** (Figures S118–S119). Moreover, compound speciation and differential labelling of structures were observed following treatments with **BODIPY‐COOH** (general non‐specific labelling), **BODIPY‐En** (dim neuromast staining), and co‐treatments of Ga(NO_3_)_3_ with **HL4** (dim gut and neuronal staining, Figure S118). Together, these observations highlight that all aspects of these compounds, presence, availability and selection of metals, BODIPY and ligand framework including the TSC motifs are crucial in controlling penetrance and targeting in an in vivo context.

The BODIPY‐tagged thiosemicarbazone–metal complexes synthesized and characterized in this study, most notably the gallium–quinoline derivative, exemplify the convergence of bioinorganic chemistry, functional imaging, toward chemotherapeutics development. As stated above, gallium(III), due to its close chemical similarity to ferric iron, is known for its capacity to bind transferrin and hijack iron uptake pathways to selectively target proliferating cancer cells, where it inhibits key iron‐dependent enzymes such as ribonucleotide reductase (RR), impairing DNA synthesis and inducing cell death [51, 52, 53]. In contrast, indium(III), though chemically related, does not effectively mimic iron and likely displays minimal interaction with transferrin, thus leading to limited cellular uptake and negligible inhibition of iron‐dependent processes [54, 55]. Ligand structure can also significantly influence compound behavior: quinoline‐ligated gallium complexes generally exhibit increased lipophilicity, stability, and mitochondrial targeting, favoring anticancer and organelle‐specific applications [56, 57, 58]. Conversely, pyrimidine‐ligated complexes generally confer different cellular uptake characteristics, which may result in altered biological effects and reduced organelle‐specific targeting [58, 59]. While Ga, Fe and In analogues used in this study localize to digestive tissues in vivo, only gallium strongly penetrates mitochondria, as verified by MitoTracker co‑labelling and in vitro experimentation. By contrast, ferric iron largely recapitulates the staining pattern of gallium but with far lower efficacy, while indium accumulates passively with limited bioactivity, suggesting potential as a non‐toxic imaging agent. Mitochondrial targeting by both the ferric‐quinoline and gallium–quinoline compounds was recapitulated in HeLa cells, validating the function in a clinically relevant human model [60].

The gallium–thiosemicarbazone–BODIPY complex behaves as a theranostic agent, combining cytotoxicity via iron mimicry and mitochondrial disruption with real‐time fluorescence imaging enabled by the BODIPY fluorophore, whose stability and photophysical properties are retained upon metal coordination [61, 62]. Notably, no prior studies have reported BODIPY‐tagged Triapine or Triapine‐derived thiosemicarbazonato‐metal complexes capable of tracking speciation or metal‐ligand integrity in cells, nor have MP FLIM‐based analyses been applied to such Ga(III), Fe(III) or In(III) TSCs in living cells. Moreover, organism‐level real‐time imaging of these complexes remains entirely unexplored, further highlighting the novelty of our approach and its promise in integrating metal‐based therapeutics with advanced fluorescence imaging.

Building on these imaging properties, the gallium–quinoline–thiosemicarbazone–BODIPY complexes presented here show strong translational potential across oncology and infectious disease applications. Complexation with quinoline–thiosemicarbazone ligands enhances bioactivity by improving membrane permeability, intracellular metal chelation dynamics, and organelle‐specific accumulation, as previously demonstrated in colon (HCT‐116) and hepatocellular (HepG2) models, where mitochondrial localization correlates with potent anticancer activity and ferroptosis‐like cell death [63]. Additionally, gallium–quinoline complexes have demonstrated efficacy against *Plasmodium falciparum*, including drug‐resistant strains, suggesting dual antitumor and antiparasitic applications [64]. Critically, the lifetime‐sensitive BODIPY tag enables FLIM to resolve real‐time intracellular behaviors, such as metal–ligand speciation, subcellular retention, and environmental sensitivity, with a level of resolution rarely achievable using conventional fluorescent agents [65, 66]. These combined features position gallium–quinoline‐TSC as a unique theranostic platform with the potential to target malignancies with high metabolic demands or mitochondrial vulnerabilities in aggressive cancers where survival rates remain low and imaging tools are insufficient [67, 68, 69].

This study shows the selective and relatively abundant accumulation of the gallium–quinoline–thiosemicarbazone complex in zebrafish neuromasts, which are highly mitochondria‐rich mechanosensory organs homologous to mammalian cochlear hair cells [70, 71]. Unlike traditional inner ear imaging agents, which are typically limited to intensity‐based emission profiles and lack anatomical specificity, the **GaL4** complex presented here demonstrates two rare and valuable properties: (i) strong, reproducible accumulation in neuromasts, and (ii) sensitivity of fluorescence lifetime to the cellular microenvironment. To our knowledge, no existing inner ear imaging agents simultaneously exhibit dynamic lifetime sensing and neuromast‐specific localization in vivo, a set of capabilities that uniquely positions these compounds for studying mitochondrial health, mechano‐transduction, and drug‐induced ototoxicity in real time [72, 73, 74]. Zebrafish lateral line neuromasts are widely used to model sensory hair cell degeneration and regeneration, especially in the context of aminoglycoside‐ or cisplatin‐induced ototoxicity, where mitochondrial depolarization and ROS generation precede cell death [75, 76, 77]. The mitochondrial localization of our gallium–quinoline probe has the potential of monitoring the dynamic, sub‐organellar resolution of such early pathological events. When combined with MP FLIM, this platform may facilitate tracking and assessment of mitochondrial dysfunction without relying on concentration‐dependent intensity readouts, offering a major advantage over traditional fluorophores used in neurosensory systems [78, 79].

The neuromast‐targeting behavior and mitochondrial selectivity of these complexes also suggest promising applications in the diagnosis and treatment of neurodegenerative diseases linked to mitochondrial dysfunction [80, 81, 82, 83]. Recent studies have shown that mitochondrial redox shifts and calcium dysregulation in hair cells may serve as early biomarkers of broader neurodegenerative processes, making neuromasts a powerful surrogate model for mitochondrial health in the nervous system [84, 85]. The use of gallium–quinoline‐BODIPY complexes of type **GaL4** in such contexts could enable non‐invasive, real‐time monitoring of mitochondrial dysfunction in live vertebrate models, offering insight into disease onset and progression. Moreover, these metal‐based chromophores can be engineered for targeted therapeutic delivery, such as conjugation to antioxidants, neuroprotective peptides, or mitochondrial uncouplers, transforming them into multimodal tools for both detection and intervention [86, 87, 88].

## 3 Conclusions

We have designed and synthesized a series of BODIPY‐tagged thiosemicarbazone metal complexes to interrogate metal speciation, intracellular localization, and dynamic behavior in living systems. These Ga(III), In(III) and Fe(III) complexes of pyrimidine and quinoline thiosemicarbazone ligands retained their structural integrity and photophysical responsiveness in both cell‐based and whole‐organism models. The developed compounds enabled complementary CFM and FLIM imaging, offering high‐resolution insight into subcellular localization, lifetime‐sensitive environmental sensing, and uptake kinetics. In vitro HeLa and PC‐3 imaging assays complemented by in vivo imaging in zebrafish embryos revealed selective biodistribution and organ‐specific uptake, with both ferric‐quinoline and in particular gallium–quinoline complexes showing conserved mitochondrial tracking and targeting of zebrafish neuromasts. Consequently, these lifetime‐responsive chromophores represent a versatile toolkit for real‐time visualization of metal–ligand behavior in complex biological environments. Using this as a platform, we are advancing their development as precision theranostic agents for targeted imaging and therapeutic applications, especially in the contexts of oncology, mitochondrial and neurosensory diseases.

In conclusion, we performed fluorescence imaging studies, including lifetime measurements of metal(III) complexes versus free ligands and biodistribution analyses in cells and zebrafish, to assess the stability of gallium in biological systems. In addition, co‐localization experiments with standard dyes [Hoechst or DAPI (*λ*_ex_ 405 nm), Mitotracker Red CMXRos (*λ*_ex_ 543 nm), Lysotracker Red DND‐99, (*λ*_ex_ 543 nm) and ER tracker Red (ex 543 nm)] were also conducted to explore the potential of these probes for organelle‐specific targeting and discovered that compounds **GaL4** and **FeL4** both localize in cellular mitochondria. We investigated how substituents on the TSC scaffold influence cellular localization, providing insights into structure–distribution relationships for these triapine‐derived probes. To evaluate the effect of the fluorescent label on antiproliferative activity, the corresponding non‐fluorescent thiosemicarbazones and their Ga(III), In(III) and Fe(III) complexes were also prepared and tested. The data suggested the metal–ligand integrity in the complexes both in vitro and in vivo over the timescale of the experiments, with real‐time high‐resolution visualization revealing subcellular distribution and offering new theranostic opportunities. By providing lifetime‐based contrast independent of local probe concentration, these constructs overcome limitations of conventional neuroimaging and open avenues for future theranostic development in neurodegenerative diseases.

## 4 Experimental Section

### 4.1 General Information

All reagents were purchased from Sigma‐Aldrich, Invitrogen or Alfa‐Aesar and were used as supplied without prior purification unless otherwise stated. Flash chromatography was performed using silica gel 60 (0.043–0.063 mm, VWR) using head pressure by means of head bellows. ^1^H NMR spectra were recorded on a Varian Mercury VX300 (300 MHz) spectrometer or a Varian Unity (500 MHz) spectrometer or a Bruker AVC 500 (500 MHz) spectrometer at 298 K and referenced to residual non‐deuterated solvent peaks. Chemical shifts were quoted in ppm with signal splittings recorded as singlet (s), doublet (d), triplet (t), quartet (q), quintet (qt) and multiplet (m). Coupling constants, *J*, were measured to the nearest 0.1 Hz and were presented as observed. ^13^C{^1^H} NMR spectra were recorded on a Varian Mercury VX300 (300 MHz) spectrometer or a Varian Unity (500 MHz) spectrometer or a Bruker AVC 500 (500 MHz) spectrometer at 298 K and were referenced to the solvent peak. ^19^F NMR spectra were recorded on a Varian Mercury VX300 (300 MHz) at 298 K and were referenced to a fluorobenzene spike (δ −113.15). NMR spectra were processed using MestreNova software, v. 14.

Mass spectrometry was performed using a Bruker Micromass LCT instrument. Mass spectra were recorded on a Micromass LCT time‐of‐flight mass spectrometer under conditions of electrospray ionization (ESI‐MS). Accurate masses were reported to four decimal places using tetraoctylammonium bromide (466.5352 Da) as an internal reference. Values were reported as a ratio of mass to charge in Daltons. Mass spectra were processed using MestreNova v. 14 and MassHunter software.

Electronic absorption spectroscopy (UV/Vis) was performed using a Perkin–Elmer Lambda 19 spectrometer, running UV Winlab software. Spectra were measured using 1 cm quartz cuvettes.

Fluorescence spectra were recorded in 1 cm quartz cuvettes using a Hitachi F‐4500 fluorescence spectrometer, running FL Solutions software. Relative quantum yields were determined by comparison to either Fluorescein in 0.1 m NaOH (*ΦR* = 0.95 at 496 nm) or [Ru(bipy)_3_](PF_6_)_2_ in water (*ΦR* = 0.042 in water at 420 nm).

Solid‐state Raman spectra were recorded using a Renishaw InVia confocal Raman microscope. Spectra were recorded in the range 200–4000 cm^−1^ using the extended scan option. Excitation was either using a 785 or 532 nm laser depending on the amount of photoluminescence. Additionally, various laser powers were used in a tunable mode as required depending on the amount of photoluminescence.

High Performance Liquid Chromatography (HPLC) was performed using a Gilson HPLC instrument equipped with a Dionex C18 Acclaim column (5 µm, 4.6 × 150 mm).

Method A: A 15‐minute gradient method was applied using H_2_O/ MeCN each containing 0.1% TFA as mobile phases with the following conditions: flow rate 1 mL/min, 0 min 20% MeCN; 1 min 20% MeCN; 4 min 95% MeCN; 11.5 min 95% MeCN; 13.5 min 15% MeCN, 15 min 15% MeCN.

Method B: A 20‐minute gradient method was applied using H_2_O/ MeCN containing 0.1% TFA as mobile phases with the following conditions: flow rate 1 mL/min, 0 min 20% MeCN; 1 min 20% MeCN; 4 min 95% MeCN; 11.5 min 95% MeCN; 17.5 min 15% MeCN, 20 min 15% MeCN.

Method C: A 35‐minute gradient method was applied using H_2_O/MeCN each containing 0.1% TFA as mobile phases with the following conditions: flow rate 1 mL/min, 0 min 5% MeCN; 12 min 95% MeCN; 26 min 95% MeCN; 30 min 5% MeCN, 35 min 5% MeCN.

### 4.2 X‐Ray Crystallography

Intensity data for **HL1** and **BODIPY‐NHS** were collected at 150(2) K on a Rigaku Xcalibur, EosS2 single crystal diffractometer using graphite monochromated Mo‐Kα radiation (*λ* = 0.71073 Å) and for **HL4, GaL1, GaL2** and **InL2** on a Rigaku SuperNova Dual EosS2 single crystal diffractometer using monochromated Cu‐Kα radiation (*λ* = 1.54184 Å). Unit cell determination, data collection data reduction and absorption correction were performed using the CrysAlisPro software [91]. The structures were solved with SHELXT [92, 93] and refined by a full‐matrix least‐squares procedure based on F^2^ (SHELXL‐2018/3) [92, 93]. All non‐hydrogen atoms were refined anisotropically. Hydrogen atoms were placed onto calculated positions and refined using a riding model. Where possible hetero atom hydrogen atoms were located in the difference Fourier map and were refined freely or with bond length restraints. The CCDC deposition data were: **HL1** (C_9_H_13_N_5_S) CCDC 2493568, **HL2** (as a HPF_6_ and H_2_O adduct C_13_H_15_N_4_SPF_6_.H_2_O CCDC 2516861, **GaL1** (C_18_H_24_GaN_10_S_2_, PF_6_) CCDC 2540008, **GaL2** (C_26_H_26_GaN_8_S_2_, PF_6_) CCDC 2516141, **InL2** (C_26_H_26_InN_8_S_2_, PF_6_) CCDC 2540007, **BODIPY‐NHS** (C_22_H_22_BF_2_N_3_O_4_) CCDC 2493570 and **HL4** (C_33_H_32_BF_2_N_7_OS) CCDC 2493569, and selected crystal data and structure refinement and molecular structures are given in Supporting Information including Figures S120–S126.

### 4.3 Cell Culture and Preparation

Cells were cultured at 37°C, 5% CO_2_ and high humidity. Once confluence was greater than 70% cells were collected. PC‐3 [94] and HeLa [95] were purchased from ATCC. PC‐3 cells were cultured in Roswell Park Memorial Institute (RMPI) 1640 serum and HeLa in Eagle's Minimum Essential Medium (EMEM) using standard protocols. The medium contained; 10% foetal calf serum (FCS), 0.5% penicillin/streptomycin (10 000 IU mL^−1^1/10 000 mg mL^−1^), and 1% 200 mm *L*‐glutamine. Phenol red was absent in all preparation steps. Supernatant containing dead cells and excess protein was aspirated. Live adherent cells were washed with 10 mL of phosphate buffer saline (PBS), removing any remaining media containing FCS. Cells were incubated with 6 mL trypsin solution (0.25% trypsin in PBS) for 8–10 min at 37°C. After this time, 6 mL of 10% RMPI medium was added to inactivate the trypsin, with the resultant solution centrifuged for 7 min (1000 rpm, room temperature), to remove any remaining dead cell matter. The supernatant liquid was aspirated, and 4 mL of 10% RMPI medium was added to the remaining cells. Cells were counted using a haemocytometer and seeded as appropriate. For AG09429 (human gingival fibroblasts) were purchased from the Coriell Institute for Medical Research (Camden, New Jersey), obtained from a 25‐year‐old apparently healthy female donor [96]. AGO9429 were cultured using the same procedure as Eagle's Minimum Essential Medium (EMEM). The media contained FCS (15%), 0.5% v/v penicillin/streptomycin (10 000 IU mL^−1^/10 000 mg mL^−1^) and 1% v/v *L*‐Glutamine (200 mm). All cells were tested for validity using standard mycoplasma screening protocols and these were contaminants free: upon defrosting, the cell stocks were cultured and maintained in accordance with the standard guidelines [97]: they were routinely checked for mycoplasma contamination and their culture was limited to 10 passages maximum from the frozen and certified stocks received.

### 4.4 MTT Assay Procedures

Cells cultured as described above were plated (7 × 10^3^ cells mL^−1^), in a 96‐well plate and were left to adhere for 48 h at 37°C and 5% CO_2_. Compounds were extensively lipophylised prior to assays and kept under vacuum drying overnight. Purity was deemed to be >90% by a combination of HPLC, NMR and TGA evaluations. The corresponding solutions were made by dissolving solids in fresh DMSO which were subsequently loaded at different concentrations into wells and were cultured for either 30 min, 24 h or 48 h, and in soe cases assyas were extended to 72 h observations. Final concentrations were: 250 µm, 100 µm, 50 µm, 10 µm, 1 µm, 0.5 µm, 0.1 µm, and 1 nm (1% compound in DMSO, 99% of 10% FCS media). After incubation for the specified time point, supernatant was aspirated from each well, which were then washed twice with PBS. 3‐(4,5‐dimethylthiazol‐2‐yl)‐2,5‐diphenyltetrazolium bromide (MTT) was added to each well (0.5 mg/mL, 10% serum free media (SFM)), followed by a three‐hour incubation. After aspiration of the MTT, 100 µL of DMSO was added to the wells. Plates were then read using a BMG LABTECH FLUOstar Optima microplate reader. Data were obtained from 3 plates per time point and was analysed using origin 9.1 to calculate IC50 values. Error was reported as the standard error.

### 4.5 Cell Culture for Imaging Procedures

Cells were seeded as monolayers in T75 tissue culture flasks (Falcon) and maintained in Roswell Park Memorial Institute (RPMI) 1640 medium supplemented with 10% fetal bovine serum (FBS), *L*‐glutamine, penicillin, and streptomycin. Cultures were incubated at 37°C in a humidified atmosphere containing 5% CO_2_ and passaged using trypsin upon reaching 70%–80% confluence.

For fluorescence microscopy, cell monolayers were plated in glass‐bottom Petri dishes three days before imaging to ensure proper adhesion, with approximately 7.5 × 10^4^ cells per dish. Prior to microscopy experiments, cells were seeded onto sterile glass dishes and incubated for 48 h to allow adherence. Stock solutions of the fluorescent compounds (1 mm in DMSO) were prepared and diluted in RPMI to achieve a final concentration of 10 µm (10 µL of a 10 mm solution in 990 µL medium). After aspiration of the growth medium, cells were washed five times with Hank's balanced salt solution (HBSS), incubated with the compound‐containing medium for 20 h at 37°C, and subsequently washed three times with HBSS to remove non‐internalized compounds. Cells were then recovered in 1 mL of serum‐free RPMI for imaging.

Confocal fluorescence microscopy was performed using a Nikon Eclipse Ti2‐E inverted confocal microscope equipped with an LU‐N3 laser unit (405, 488, and 561 nm) or a Nikon A1Rsi laser scanning confocal system fitted with a 60× oil‐immersion objective lens. The microscope included a motorized piezo Z‐stage, halogen lamp, and mercury lamp for widefield fluorescence imaging. All images were processed using the NIS‐Elements software package (Nikon Instruments).

Cell uptake studies using correlated CFM and two‐photon FLIM were carried out on live PC‐3 or HeLa cells adhered to glass‐bottom Petri dishes and incubated with the compounds for 15, 20, or 30 min at 37°C. Control images were recorded from cells exposed to Dulbecco's Modified Eagle Medium (DMEM) containing 1% DMSO to account for cellular autofluorescence. The compounds were dissolved in 1% DMSO and added to the cell culture medium to give final concentrations ranging from 10 to 100 µm. Fluorescence lifetime decays were collected for the entire field of view at 5 min intervals, with uptake reaching a maximum within 30 min. The data and images presented were obtained after 20 min of incubation.

Epifluorescence microscopy images were captured using a Nikon Eclipse TE2000 epifluorescence microscope. Cells were cultured as described above and plated in a glass‐bottom petri dish (35 mm diameter and 1.5 mm thickness) at 1.5 × 10^5^–2.5 × 10^5^ cells per dish and were left 48 h for cells to adhere. Prior to microscopy observation, cultured cells were washed with PBS three times and refilled with 990 µL of serum‐free media. Subsequently, compounds (10 µL in DMSO) were loaded to make the final volume 1 mL, at the appropriate concentration. Final concentrations were incubated with 1% DMSO. After the appropriate incubation time at either 37°C or 4°C, cells were washed with PBS three times, refilled with fresh SFM (1 mL) with confocal images captured immediately afterward.

Images were processed using Nikon NIS elements‐AR Analysis 4.30.02 software.

Co‐localisation Studies: To glass‐bottom petri dishes loaded with compound and washed as described above, 10 µL of desired tracking dye in 990 µL SFM was added, to give final, working concentrations as stated by the manufacturer. Dyes used were: ER‐Tracker Red (BODIPY TR Glibenclamide), MitoTracker Red CMXRos, Invitrogen LysoTracker Red DND‐99, ER‐Tracker Green (BODIPY FL Glibenclamide), and Invitrogen MitoTracker Green FM. Microscope dishes were then incubated for 20 min at 37°C. Plates were then washed with PBS twice, refilled with 1 mL fresh SFM and immediately imaged. Images were processed using Nikon NIS elements‐AR Analysis 4.30.02 software. Pearson values were calculated from 3 random fields of view over 3 independent experiments. Errors were reported as the standard deviation.

**Fluorescence Lifetime imaging** microscopy experiments were carried out at the Rutherford Appleton Laboratory using the Central Laser Facility OCTOPUS cluster. The instruments used for the 1P confocal‐FLIM and 2P‐FLIM have been previously described [89].

2‐Photon FLIM: Correlated images were acquired by raster scanning focused near‐infrared (NIR, 910 nm) femtosecond pulsed laser light (200 fs, 76 MHz; Coherent Mira F900, pumped by Verdi V15 lasers) through a 60× water‐immersion objective (NA 1.2). Emission was filtered using a BG39 filter following multiphoton excitation and detected with a Hamamatsu R3809U photomultiplier tube. Point fluorescence decays were recorded for each pixel (minimum 128 × 128 pixels), and lifetimes were calculated using Becker & Hickl SPCImage software (v. 4) as previously described [43, 44, 45, 46, 47, 48, 49, 50].

For the 1‐Photon excitation, 488 nm light was obtained using a Super K Extreme NKT‐SC 390–2000 nm super continuum laser (NKT, Photonic Solutions, UK) (variable repetition rate with 40–60 ps pulse width). The laser repetition rate was reduced to 39 MHz. SuperK SELECT (NKT) was used to obtain the 488 nm. Imaging was carried out on a Nikon Ti2‐E microscope equipped with a 60×, NA 1.27 water immersion objective. A Nikon EC2‐Si confocal scan head was used to raster‐scan the laser, and fluorescence was collected through the same objective. Detection was performed using a hybrid photomultiplier tube (HPM100‐40, Becker & Hickl), with a 525/39 bandpass filter and an FL515 long‐ pass filter (Thorlabs) to eliminate laser scatter.

For the 2‐Photon excitation, a mode‐locked tunable laser, 660–1320 nm (Chameleon Discovery NX, Coherent Lasers), was used to provide a laser light at a wavelength of 910 nm with 100 fs pulse width at 80 MHz. this is a custom‐built system constructed around the same 1PE microscope coupled to a Nikon EC1 modified confocal scanhead.

FLIM acquisition was carried out using the same confocal setup, integrated with a Becker & Hickl SPC830 or SPC‐QC 104 time‐correlated single‐photon counting (TCSPC) module, controlled via SPCM software (version 9.0, 64‐bit). Images were acquired at a resolution of 256 × 256 or 512 × 512 pixels using FiFo mode, which records individual photon arrival times and spatial coordinates, storing the data on the TCSPC PC card. Before FLIM data acquisition, the instrument response function (IRF) was determined to correct for electronic noise and laser pulse fluctuations. Calibration was performed using fluorophores with well‐characterized lifetimes, including 1 µm fluorescein, rhodamine B, and 7‐hydroxycoumarin carboxylic acid in water. Imaging proceeded only when measured lifetimes were within 5% of published values [90].

### 4.6 Zebrafish Husbandry

All experiments were conducted with approval from the local ethical review committee at the University of Bath and in accordance with the UK Home Office regulations (Guidance on the Operation of Animals, Scientific Procedures Act, 1986). All work was performed on zebrafish (*Danio rerio*) using wildtype AB strains, which were reared at 28.5°C on a 14 h light/10 h dark cycle. Staging and husbandry were performed as previously described [98]. Embryos were raised in E3 media (5 mm NaCl, 0.17 mm KCl, 0.33 mm CaCl_2_, 0.33 mm MgSO_4_) with 0.1% methylene blue up to the point of experimentation, according to standard protocols.

### 4.7 Zebrafish Compound Treatment and Imaging Experiments

Compound treatment was performed at the indicated concentrations (10 or 200 µm) in 1% DMSO for 120 min, using E3 media without additional methylene blue. The Ga(NO_3_)_3_ was at a range of 100 µm to 10 mm, with 100 µm the one selected for pre‐incubation. Following treatment, larvae were washed 3 × 5 min in E3 media, followed by immediate imaging. Prior to imaging, zebrafish larvae (4 dpf) were anaesthetized using Tricaine (Sigma‐Aldrich). For confocal imaging, fish were subsequently mounted in 1% low‐melting point agarose in 60 mm petri dishes and submerged in a layer of E3 media approximately 6 mm in depth.  Fluorescent stereomicroscope images were generated on a Leica MZFLiii system (Leica Microsystems) and captured using an Olympus EP50 camera (Olympus/Evident Corporation). Confocal images were generated on an Olympus Fluoview FV3000 system (Evident).

### 4.8 Zebrafish Image Analysis and Statistics

Fiji (ver. 2.16.0) was used for all image analysis. Fluorescent regions of interest (ROIs) were selected by utilizing the “wand” tool (Legacy mode), and these were used to quantify fluorescence intensity for the digestive system. Mean fluorescence intensity values were extracted for each ROI. Mean values were calculated and plotted using GraphPad Prism (ver. 10.6.1). Statistical analysis was carried out using a one‐way ANOVA test. Results are represented as mean ± SEM. The criterion for statistical significance was set at *p* < 0.05.

### 4.9 Synthesis of (E)‐*N,N*‐2‐(1‐(pyrimindin‐2‐yl)ethylidene)Hydrazine‐1‐Carbothiomaide (HL1)


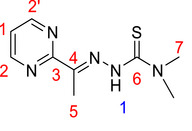


2‐Acetylpyrimidine (100 mg, 0.89 mmol) and 4,4‐dimethyl‐3‐thiosemicarbazide (300 mg, 2.5 mmol) were suspended in EtOH (20 mL). The resultant solution was stirred at room temperature and after 5 min a drop of HCl (75 µL) was added, and the reaction was left to stir for 1 h. The resultant precipitate was vacuum filtered and washed with cold EtOH to afford a yellow solid (138 mg, 69%).

Anal. Calcd for C_9_H_13_N_5_S: C, 48.39; H, 5.87; N, 31.37. Found: C, 48.76; H, 5.68; N, 32.17.

**^1^H NMR** (400 MHz, (CD_3_)_2_SO, 298 K): δ 11.08 (s, 1H, NH), 8.96 (d, *J* = 4.9 Hz, 2H, H‐2), 7.56 (t, *J* = 4.9 Hz, 1H, H‐1), 2.62 (s, 6H, H‐7), 2.42 (s, 3H, H‐5).

**^13^C{^1^H} NMR** (101 MHz, (CD_3_)_2_SO, 298 K): δ 179.3 (C‐6), 159.1 (C‐3), 157.8 (C‐2), 140.7 (C‐4), 120.7 (C‐1), 40.6 (C‐7).

**ESI‐MS**: [M+H]^+^ C_9_H_13_N_5_S calc. 224.096, found: 224.097.

**HPLC** (Method A): Rt (min) 8.41.

**IR** (solid): *ν* (cm^−1^) 3316 (NH), 3115 (NH), 3066, 2929, 2896, 1990, 1902, 1616, 1598, 1558, 1522, 1503, 1458, 1425, 1363, 1302, 816, 79, 450.

### 4.10 Synthesis of (*E*)‐*N*,*N*‐Dimethyl‐2‐(quinolin‐2‐ylmethylene)Hydrazine‐1‐Carbothioamide (HL2)


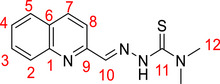


2‐Quinolinecarboxaldehyde (200 mg, 1.27 mmol) and 4,4‐dimethyl‐3‐thiosemicarbazide (150 mg, 1.25 mmol) were suspended in EtOH (20 mL) and stirred at room temperature. After 5 min of stirring, HCl (75 µL) was added, the solution was then left stirring for a further hour. The orange product was collected by vacuum filtration (278 mg, 86%).

Anal. Calcd for C_26_H_26_N_8_S_2_: C, 60.44; H, 5.58; N, 21.69. Found: C, 60.97; H, 5.01; N, 21.26.

**^1^H NMR** (400 MHz, (CD_3_)_2_SO, 298 K): δ 11.11 (s, 1H, NH), 8.61 (d, *J *= 8.7 Hz, 1H, H‐8), 8.55 (s, 1H, H‐10), 8.17 (d, *J* = 8.7 Hz, 2H, H‐5, H‐7), 8.11 (d, *J* = 8.2 Hz, 1H, H‐2), 7.90 (t, *J *= 8.7 Hz, 1H, H‐4), 7.72 (t, *J* = 7.7 Hz, 1H, H‐3), 3.36 (s, 6H, H‐12).

**^13^C{^1^H} NMR** (101 MHz, (CD_3_)_2_SO, 298 K): δ 178 (C‐11), 152.8 (C‐9), 147.3 (C‐1), 143.7 (C‐10), 139 (C‐7), 138.7 (C‐3), 131.3 (C‐2), 128.2 (C‐5), 127.8 (C‐6), 126.5 (C‐4), 118.3 (C‐8), 40.1 (C‐12).

**ESI‐MS**: [M+H]^+^ C_13_H_14_N_4_S calc. 259.1017, found: 259.1019.

**HPLC** (Method A): Rt (min) 8.23.

**IR** (solid): *ν* (cm^−1^) 3063 (NH), 3009 (NH), 2921, 1622, 1576, 1540, 1497, 1449, 1424, 1363, 1300, 831, 819, 787, 746, 706.

### 4.11 Synthesis of Complex GaL1


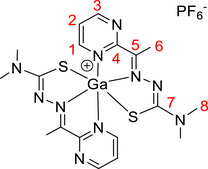


Compound **HL1** (50 mg, 0.22 mmol) and Ga(NO_3_)_3_ (28 mg 0.11 mmol) were dissolved in EtOH (15 mL) and stirred for 1 h at room temperature. KPF_6_ (18 mg, 0.11 mmol) was added to the solution which was left to stir for an hour. Precipitate was collected by vacuum filtration and washed with cold EtOH, to afford a yellow solid (42.4 mg, 74%). Elemental Anal. Calcd for C_18_H_24_GaN_10_PF_6_S_2_·1.5H_2_O: C, 31.50; H, 3.97; N, 20.41. Found: C, 31.53; H, 3.57; N, 20.91.

**^1^H NMR** (400 MHz, CD_3_CN, 298 K): δ 9.09–8.30 (broad m, 2H, H‐1 H‐3), 7.63 (t, *J* = 5.2 Hz, 1H, H‐2), 3.38 (s, 12H, H‐8), 2.79 (s, 6H, H‐6).

**^19^F NMR** (376 MHz, DMSO) δ −70.15 (P*F6*, d*, J *= 711 Hz).

**^31^P NMR** (162 MHz, DMSO) δ −144.19 (sept, *J* = 711 Hz)

**ESI‐MS**: [M]^+^ C_18_H_24_GaN_10_S_2_ calc. 513.0879, found: 513.0880.

**HPLC** (Method A): Rt (min) 9.56.

**IR** (solid): *ν* (cm^−1^) 3078 (weak), 2933, 2859, 2778, 1583, 1555, 1510, 1455, 1385, 1361, 1304, 825, 744, 653, 555.

### 4.12 Synthesis of Complex GaL2


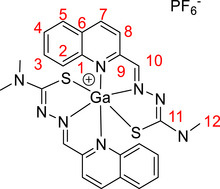


Compound **HL2** (60 mg, 0.24 mmol) and Ga(NO_3_)_3_ (31 mg 0.12 mmol) were dissolved in EtOH (15 mL) and stirred for 1 h at room temperature. KPF_6_ (23 mg, 0.12 mmol) was then added to the solution which was left to stir for an hour. Precipitate was collected by vacuum filtration and washed with cold EtOH, to afford a bright orange solid (56 mg, 76%).

**^1^H NMR** (400 MHz, (CD_3_)_2_SO, 25°C, 298 K): δ 9.28 (s, 2H, H‐10), 8.85 (d, *J* = 8.5 Hz, 2H, H‐7), 8.19 (d, *J* = 8.5 Hz, 2H, H‐8), 8.07 (dd, *J *= 8.2, 1.5 Hz, 2H, H‐5), 7.69 (ddd, *J* = 8.6, 6.9, 1.6 Hz, 2H, H‐3), 7.58 (ddd, *J* = 8.1, 6.9, 1 Hz, 2H, H‐4), 7.44 (d, *J* = 8.7 Hz, 2H, H‐2), 3.32 (s, 6H, H‐12), 3.18 (s, 6H, H‐12).

**^19^F NMR** (376 MHz, DMSO) δ −70.13 (P*F6*, d*, J = *711 Hz).

**^31^P NMR** (162 MHz, DMSO) δ −144.19 (sept, *J* = 711 Hz)

**^71^Ga NMR** (122 MHz, DMSO) δ −1.81

**ESI‐MS**: [M]^+^ C_26_H_26_GaN_8_S_2_ calc. 583.0978, found: 583.0979.

**HPLC** (Method A): Rt (min) 7.95.

**IR** (solid): *ν* (cm^−1^) 3091 (weak), 2978.5, 2933, 1601, 1589 (sh), 1512, 1391, 1364, 1310, 1297, 823, 776, 774, 556.

### 4.13 Synthesis of Complex InL1


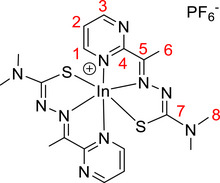


Compound **HL1** (75 mg, 0.34 mmol) and In(NO_3_)_3_ (37 mg 0.16 mmol) were dissolved in EtOH (15 mL) and stirred for 1 h at room temperature. KPF_6_ (31 mg, 0.16 mmol) was then added to the solution which was left to stir for a further hour. Precipitate was collected by vacuum filtration and washed with cold EtOH, to afford the desired product as a bright yellow solid (75 mg, 80%).

**^1^H NMR** (400 MHz, CD_3_CN, 298 K): δ 9.09 (m, 2H, H‐1/3), 8.94 (m, 2H, H‐1 /3), 7.69 (t, 2H, *J *= 5.1 Hz, H‐2), 3.38 (s, 12H, H‐8), 2.65 (s, 6H, H‐6).

**^13^C{^1^H} NMR** (101 MHz, (CD_3_)_2_SO, 298 K) δ: 175.2 (C‐7), 155.9 (C‐5), 142.9 (C‐4), 123.5 (C‐2), 121.5 (C‐1/3), 43.2 (C‐8), 13 (C‐6).

**^19^F NMR** (376 MHz, DMSO) δ −70.15 (P*F6*, d*, J = *711 Hz).

**^31^P NMR** (162 MHz, DMSO) δ −144.19 (sept, *J* = 711 Hz)

**ESI‐MS**: [M]^+^ C_18_H_24_InN_10_S_2_, calc. 599.0660, found: 559.0662.

**HPLC** (Method A): Rt (min) 9.14.

**IR** (solid): *ν* (cm^−1^) 2927, 2851, 1582, 1552, 1503, 1446, 1385, 1364, 1297, 829, 793, 556, 433.

### 4.14 Synthesis of Complex InL2


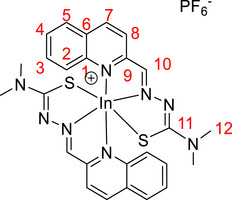


Compound **HL2** (50 mg, 0.20 mmol) and In(NO_3_)_3_ (22 mg 0.20 mmol) were dissolved in EtOH (15 mL) and stirred for 1 h at room temperature. KPF_6_ (19 mg, 0.20 mmol) was then added to the solution which was left to stir for a further hour. The precipitate was collected by vacuum filtration and washed with cold EtOH, to afford the desired product as an orange solid (47 mg, 74%).

**^1^H NMR** (400 MHz, (CD_3_)_2_SO, 298 K): δ 9.13 (d, *J* = 8.8 Hz, 1H, H‐8), 8.76 (d, *J* = 8.5 Hz, 1H, H‐7), 8.64 (s, 1H, H‐10), 8.17–8.1 (m, 1H, H‐5), 8.01–7.90 (m, 2H, H‐2,4), 7.74 (t, *J *= 7.5 Hz, 1H, H‐3), 3.34 (s, 6H, H‐12).

**^13^C{^1^H} NMR** (101 MHz, (CD_3_)_2_SO, 298 K) δ: 183.2 (C‐11), 177 (C‐9), 148.8 (C‐1), 143.2 (C‐8), 143 (C‐10), 137.7 (C‐3), 133.9 (C‐7), 129.9 (C‐6), 128.3 (C‐4), 124.2 (C‐2), 122.5 (C‐5), 40.1 (C‐12).

**^19^F NMR** (376 MHz, DMSO) δ −70.15 (P*F6*, d*, J *= 711 Hz).

**^31^P NMR** (162 MHz, DMSO) δ −144.19 (sept, *J* = 711 Hz)

**ESI^+^‐MS**: [M]^+^ C_26_H_26_InN_8_S_2_ calc. 629.0753, found: 629.0751.

**HPLC** (Method A): Rt (min) 8.05.

**IR** (solid): *ν* (cm^−1^) 3349 (weak), 3188 (weak), 2978.5, 2930, 1653, 1635, 1592, 1506, 1394, 1367, 1295, 900, 898, 750, 625, 487, 462.

**(E)‐4‐(5,5‐difluoro‐1,3,7,9‐tetramethyl‐5H‐4l4,5l4‐dipyrrolo[1,2‐c:2',1'‐f][1,3,2]diazaborinin‐10‐yl)‐N‐(2‐(2‐(1‐(pyrimidin‐2‐yl)ethylidene)hydrazine‐1‐carbothioamido)ethyl)benzamide, HL3**


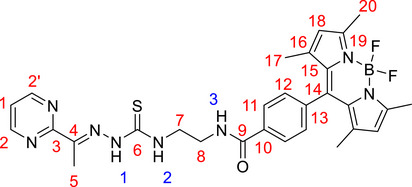


Compound **HL1** (130 mg, 0.58 mmol) and compound **BODIPY‐En** (300 mg, 0.73 mmol) were suspended in MeCN (30 mL) and refluxed for 16 h at 90°C. The resulting solution was dried under vacuum, and the residual solid redissolved in the minimum amount of CH_2_Cl_2_. Flash chromatography was used to purify the crude mixture using DCM/MeOH (0%–10%). The desired product was obtained as a red solid (200 mg, 58%).

**^1^H NMR** (400 MHz, (CD_3_)_2_SO, 298 K): δ 13.97 (s, 1H, NH‐1), 8.95–8.91 (m, 2H, H‐11), 8.81 (d, *J* = 4.9 Hz, 1H, H‐2), 8.25 (t, *J* = 6.5 Hz, 1H, NH‐2), 8.05 (d, *J* = 8.2 Hz, 1H, H‐2), 7.83 (t, *J* = 6.5 Hz, 1H, NH‐3), 7.39–7.34 (m, 3H, H‐1/12), 5.96 (s, 2H, H‐18), 4.19–4.11 (m, 2H, H‐7), 3.81–3.77 (m, 2H, H‐8), 2.55 (s, 6H, H‐20), 2.47 (s, 3H, H‐5), 1.34 s, 6H, H‐17).

**^19^F{^1^H} NMR** (376 MHz, (CD_3_)_2_SO, 298 K): δ −146.26 (dd, *J* = 65.4, 32.3 Hz).

**^11^B{^1^H} NMR** (128 MHz, (CD_3_)_2_SO, 298 K): δ 0.77 (t, *J* = 33 Hz).

**ESI‐MS**: [M+H]^+^ C_29_H_31_BF_2_N_8_OS, calc. 589.2840, found: 589.2480.

**HPLC** (Method B): Rt (min) 6.63

**IR** (solid): *ν* (cm^−1^) 3255 (NH), 2962, 2924, 2850, 1645, 1543, 1505, 1466, 1407, 1306, 1193, 1150, 1093, 804, 734, 475

**(E)‐4‐(5,5‐difluoro‐1,3,7,9‐tetramethyl‐5H‐4l4,5l4‐dipyrrolo[1,2‐c:2',1'‐f][1,3,2]diazaborinin‐10‐yl)‐N‐(2‐(2‐(quinolin‐2‐ylmethylene)hydrazine‐1‐carbothioamido)ethyl)benzamide HL4**


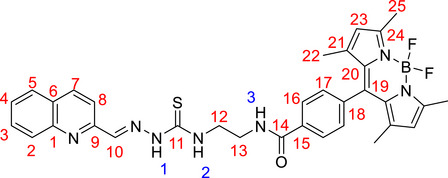


Compound **HL2** (125 mg, 0.48 mmol) and compound **BODIPY‐En** (224 mg, 0.55 mmol) were suspended in MeCN (30 mL) and refluxed for 16 h. The resulting solution was dried under reduced pressure, and the residual solid redissolved in DCM. Flash chromatography was used to purify the crude mixture using DCM/MeOH (0%–10%). The desired product was obtained as a red solid (195 mg, 65%).

**^1^H NMR** (400 MHz, (CD_3_)_2_SO, 298 K): δ 11.95 (s, 1H, NH‐1), 9.07 (t, *J* = 5.3 Hz, 1H, NH‐2), 8.94 (t, *J* = 5.6 Hz, 1H, NH‐3), 8.53 (d, *J* = 8.7 Hz, 1H, H‐8), 8.40 (d, *J* = 8.7 Hz, 1H, H‐7), 8.24 (s, 1H, H‐10), 8.13–8.09 (m, 2H, H‐16), 8.02–7.97 (m, 2H, H‐2), 7.79–7.76 (m, 1H, H‐4), 7.64–7.60 (m, 1H, H‐3), 7.54–7.51 (m, 2H, H‐17), 6.16 (s, 2H, H‐23), 3.80 (q, *J* = 5.9 Hz, 2H, H‐13), 3.60 (q, *J* = 5.8 Hz, 2H, H‐12), 2.45 (s, 6H, H‐25), 1.31 (s, 6H, H‐22). 194.28, 178.22, 166.95, 163.52, 155.64, 147.39, 143.11, 141.45, 138.52, 137.53, 135.13, 131.38, 130.23, 128.86, 127.76, 121.98, 118.47, 117.63, 49.06, 44.56, 41.45, 34.86, 29.44, 14.68, 14.53.

**^19^F{^1^H} NMR** (376 MHz, (CD_3_)_2_SO, 298 K): δ −145.86 (dd, *J* = 65.4, 32.3 Hz).

**^11^B{^1^H} NMR** (128 MHz, (CD_3_)_2_SO, 298 K): δ 0.75 (t, *J *= 33 Hz).

**ESI‐MS**: [M+H]^+^ C_33_H_32_BF_2_N_7_OS calc. 624.2525, found: 624.2525.

**HPLC** (Method B): Rt (min) 6.11, (Method C): Rt (min) 11.72 min

**IR** (solid): *ν* (cm^−1^) 3247 (NH), 2970, 2920, 1655, 1540, 1502, 1476, 1410, 1306, 1190, 1153, 1090, 805, 735, 476.

### 4.15 Synthesis of Complex GaL3


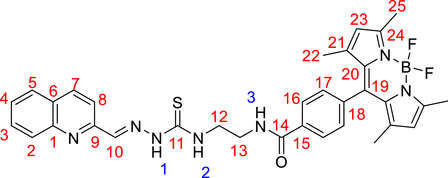


Compound **HL3** (20 mg, 0.04 mmol) and Ga(NO_3_)_3_ (4.6 mg, 0.02 mmol) were dissolved in MeOH (8 mL) and stirred at room temperature for 3 h. KPF_6_ (3.2 mg, 0.02 mmol) was then added to the reaction mixture which was left stirring for a further hour. The precipitate was then collected by vacuum filtration and washed with cold MeOH to afford the desired complex as a red solid. Product was obtained as a red solid (18 mg, 67%) which was further recrystallized from CHCl_3_.

**^1^H NMR** (500 Hz, (CD_3_)_2_SO, 298 K): δ 11.87 ((b, protonation of C═N(H) in solution), 8.82 (dd, 2H, H‐2), 8.07 (d, ^3^ *J* = 8 Hz, 4H, H‐12), 7.87 (m, 4H, H‐1, H‐3), 7.54 (d, ^3^ *J* = 8 Hz, 4H, H‐13), 6.20 (s, 4H, H‐19), 3.54 (m, 4H, H‐8), 3.04 (m, 4H, H‐9), 2.52 (s, 6H, H‐6), 2.46 (s, 12H, H‐21), 1.34 (s, 12H, H‐18). **^13^C{^1^H} NMR** (151 MHz, DMSO‐*d*_6_) δ 197.85, 179.13, 178.07, 166.74, 162.25, 159.88, 158.03, 155.66, 147.22, 146.43, 143.79, 141.42, 139.31, 137.20, 135.36, 130.87, 128.67, 122.17, 121.38, 43.75, 14.58.

**^19^F{^1^H} NMR** (376 MHz, (CD_3_)_2_SO, 298 K): δ −143.64 (dd, B*F2, J = *65.4, 32.3 Hz); −70.15 (P*F6, d, J *= *711 Hz*).

**^11^B{^1^H} NMR** (128 MHz, (CD_3_)_2_SO, 298 K): *δ* 0.59 (t, *J *= 32.6 Hz).

**^31^P NMR** (162 MHz, (CD_3_)_2_SO) *δ* −144.19 (sept, *J *= 711)

**Mass Spectrum**: ESI‐MS Calc. For C58H60B2F4GaN16O2S2 [M]+: 1243.3981, Found: 1243.4024

**HPLC** (Method B): Rt (min) 9.88

**IR** (solid): *ν* (cm^−1^) 2962, 1648, 1541, 1506, 1469, 1403, 1361, 1305, 1186, 1158, 1054.

### 4.16 Synthesis of Complex GaL4


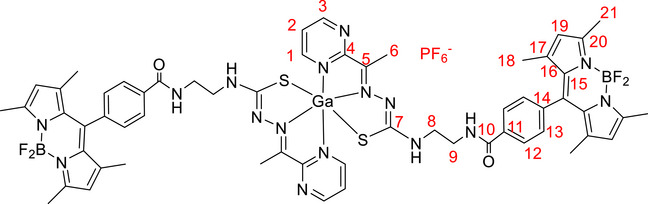


Compound **HL4** (25 mg, 0.04 mmol) and Ga(NO_3_)_3_ (4.6 mg, 0.02 mmol) were dissolved in MeOH (8 mL) and stirred at room temperature for 3 h. KPF_6_ (3.2 mg, 0.02 mmol) was then added to the reaction mixture which was left stirring for a further hour. The precipitate was then collected by vacuum filtration and washed with cold MeOH to afford the desired complex as a red solid (16 mg, 74%) which was further recrystallized from CHCl_3_.

**^1^H NMR** (400 MHz, (CD_3_)_2_SO, 298 K): δ 11.92 (b, protonation of C═N(H) in solution), 9.02 (t, *J* = 5.3 Hz, 1H, *NH‐1*), 8.93 (t, *J* = 5.3 Hz, 1H, NH‐2), 8.48 (d, *J* = 8.7 Hz, 1H, H‐8), 8.40 (d, *J* = 8.7 Hz, 1H, H‐7), 8.22 (s, 1H, H‐10), 8.16–8.05 (m, 2H, H‐16), 8.02–7.97 (m, 2H, H‐2), 7.80–7.75 (m, 1H, H‐4), 7.65–7.60 (m, 1H, H‐3), 7.54–7.51 (m, 2H, H‐17), 6.17 (s, 2H, H‐23), 3.80 (q, *J* = 5.9 Hz, 2H, H‐13), 3.60 (q, *J* = 5.8 Hz, 2H, H‐12), 2.45 (s, 6H, H‐25), 1.28 (s, 6H, H‐22).

**^13^C{^1^H}** (126 MHz, DMSO‐*d*_6_): δ 197.85, 179.13, 178.07, 166.74, 162.25, 159.88, 158.03, 155.66, 147.22, 146.17, 143.79, 143.27, 143.00, 141.42, 139.31, 137.20, 135.36, 130.87, 128.50, 122.17, 121.38, 39.90, 39.63, 14.58

**^19^F{^1^H} NMR** (376 MHz, (CD_3_)_2_SO, 298 K): δ −143.64 (dd, B*F2, J = *65.4, 32.3 Hz); −70.14 (P*F6*, d, *J* = 711 Hz).

**^11^B{^1^H} NMR** (128 MHz, (CD_3_)_2_SO, 298 K): δ 0.57 (t, *J *= 32.3 Hz).

**^31^P NMR** (162 MHz, DMSO) δ −142.06 (septet, *J* = 711 Hz).

**^71^Ga NMR** (122 MHz, DMSO) δ −1.93.

**ESI‐MS**: [M+H]^+^ C_66_H_62_B_2_F_4_GaN_14_O_2_S_2_ calc. 1313.4000, found: 1313.4015.

**IR** (solid): *ν* (cm^−1^) 2961, 1645, 1542, 1506, 1474, 1404, 1371, 1304, 1190, 1155, 1071.

**HPLC** (Method C): Rt (min) 13.69 min

### 4.17 Synthesis of Complex InL3


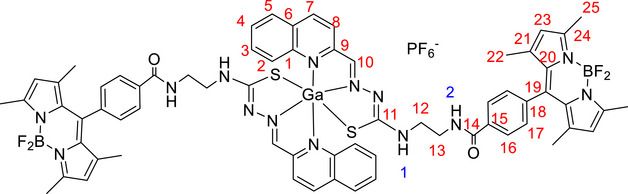


Compound **HL3** (20 mg, 0.04 mmol) and In(NO_3_)_3_ (6 mg, 0.02 mmol) were dissolved in MeOH (8 mL) and stirred at room temperature for 3 h. KPF_6_ (3.2 mg, 0.02 mmol) was then added to the reaction mixture which was left stirring for a further hour. The precipitate was then collected by vacuum filtration and washed with cold MeOH to afford the desired complex as a dark red solid (24 mg, 84%) which was further recrystallized from CHCl_3_.

**^1^H NMR** (400 Hz, DMSO‐d6, 25°C): δ 11.93 ((b, protonation of C═N(H) in solution), 8.83 (m, 2H, H‐2), 8.05 (d, ^3^ *J* = 8 Hz, 4H, H‐12), 7.85 (m, 4H, H‐1, H‐3), 7.55 (d, ^3^ *J* = 8 Hz, 4H, H‐13), 6.25 (s, 4H, H‐19), 3.56 (m, 4H, H‐8), 3.10 (m, 4H, H‐9), 2.55 (s, 6H, H‐6), 2.49 (s, 12H, H‐21), 1.36 (s, 12H, H‐18).

**^1^H NMR** (400 MHz, CDCl_3_) δ 13.90 (b, protonation of C═N(H) in solution), 8.95 (s, 1H, H‐2), 8.87 (*dd*, *J* = 4.9, 3.4 Hz, 2H H‐1, H‐3), 8.19–7.77 (broad m, H11‐H13), 5.90 (s, 2H, H‐19), 4.09 (m, *H‐8*), 3.81–3.38 (m, 4H, H‐9), 2.48 (s, 6H, H‐6), 2.40 (broad s, 12H, H‐21), 1.27 (s, 12H, H‐18)

**^19^F NMR** (376 MHz, CDCl_3_) δ −72.59 (d, *J* = 48.2 Hz), −146.26 (dd, *J* = 34, *J = *65.4 Hz).

**^11^B NMR** (128 MHz, CDCl_3_) δ 0.75 (t, *J* = 34 Hz).

**ESI‐MS**: [M]^+^ C_58_H_60_B_2_F_4_InN_16_O_2_S_2,_ calc. 1289.3700, found: 1289.3700.

**HPLC** (Method B): Rt (min) 12.96.

**IR** (solid): *ν* (cm^−1^) 2961, 1644, 1542, 1506, 1467, 1400, 1360, 1304, 1181, 1154, 1048, 970, 832, 825, 788, 732.

### 4.18 Synthesis of Complex InL4


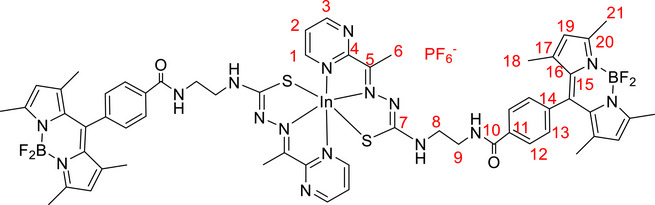


Compound **HL4** (25 mg, 0.04 mmol) and In(NO_3_)_3_ (6 mg, 0.02 mmol) were dissolved in MeOH (8 mL) and stirred at room temperature for 3 h. KPF_6_ (3.2 mg, 0.02 mmol) was then added to the reaction mixture which was left stirring for a further hour. The precipitate was then collected by vacuum filtration and washed with cold MeOH to afford the desired complex as a dark red solid (22 mg, 82%) which was further recrystallized from CHCl_3_.

**^1^H NMR** (400 MHz, (CD_3_)_2_SO, 298 K): δ 9.06 (*t*, *J* = 5.3 Hz, 1H, N*H‐1*), 8.94 (*t*, *J* = 5.3 Hz, 1H, *NH‐2*), 8.59 (d, *J* = 8.7 Hz, 1H, H‐8), 8.52 (d, *J* = 8.7 Hz, 1H, H‐7), 8.24 (s, 1H, H‐10), 8.20–8.01 (m, 2H, H‐16), 8.02–7.95 (m, 2H, H‐2), 7.80–7.75 (m, 1H, H‐4), 7.75–7.65 (m, 1H, H‐3), 7.64–7.55 (m, 2H, H‐17), 6.15 (s, 2H, H‐23), 3.80 (*q*, *J* = 5.9 Hz, 2H, H‐13), 3.60 (*q*, *J *= 5.8 Hz, 2H, H‐12), 2.45 (s, 6H, H‐25), 1.29 (s, 6H, H‐22). **^13^C{^1^H}** (126 MHz, DMSO‐*d*_6_):197.85, 179.13, 178.07, 166.74, 162.25, 159.88, 158.03, 155.66, 147.22, 146.17, 143.79, 143.27, 141.42, 139.31, 137.20, 135.36, 130.87, 128.50, 122.17, 121.38, 39.90, 39.63, 14.58

**^19^F NMR** (376 MHz, (CD_3_)_2_SO) δ −70.12 (*d*, *J* = 48.2 Hz), −143.62 (*d*, *J* = 31, 65.4 Hz).

**^11^B NMR** (128 MHz, (CD_3_)_2_SO) δ 0.58 (*t*, *J* = 32.9 Hz).

**^31^P NMR** (162 MHz, DMSO) δ −144.23 (septet, *J* = 711 Hz).

**ESI‐MS**: [M]^+^ C_66_H_62_B_2_F_4_InN_14_O_2_S_2_ calc. 1359.8696, found: 1359.8700.

**IR** (solid): ν (cm^−1^) 2961, 1644, 1542, 1506, 1467, 1400, 1360, 1304, 1181, 1154, 1048, 975, 821, 736.

**HPLC** (Method C): Rt (min) 13.67 min

### 4.19 Synthesis of Complex FeL3


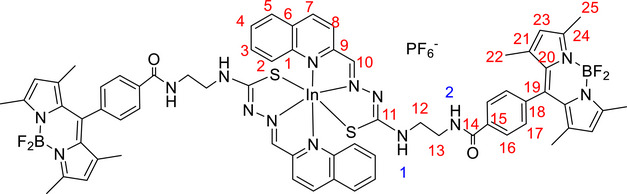


Compound **HL3** (25 mg, 0.04 mmol) and Fe(NO_3_)_3_ (8.5 mg, 0.02 mmol) were dissolved in MeOH (5 mL) and stirred at room temperature for 3 h. KPF_6_ (4 mg, 0.02 mmol) was then added to the reaction mixture which was left stirring for a further hour. The precipitate was then collected by vacuum filtration and washed with cold MeOH to afford the desired complex as a dark red solid (22 mg, 85%), which was further recrystallized from CHCl_3_.

**ESI‐MS**: [M]+ C_58_H_60_B_2_F_4_FeN_16_O_2_S_2_, calc. 1230.4012, found: 1230.4066.

**HPLC** (Method B): Rt (min) 8.77

**IR** (solid): ν (cm^−1^) 2963, 1643, 1543, 1508, 1468, 1402, 1363, 1306, 1192, 1155, 1084, 975, 832, 736.

### 4.20 Synthesis of Complex FeL4


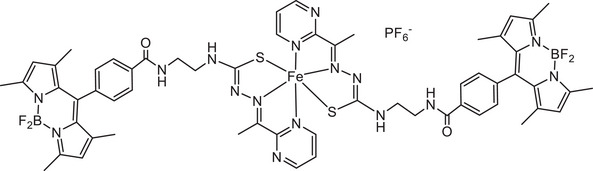


Compound **HL4** (18 mg, 0.03 mmol) and Fe(NO_3_)_3_ (6 mg, 0.015 mmol) were dissolved in MeOH (4 mL) and stirred at room temperature for 3 h. KPF6 (4 mg, 0.02 mmol) was then added to the reaction mixture which was left stirring for a further hour. The precipitate was then collected by vacuum filtration and washed with cold MeOH to afford the desired complex as a dark red solid (15 mg, 82%) which was further recrystallized from CHCl_3_.

**ESI‐MS**: [M]+ C_66_H_62_B_2_F_4_FeN_14_O_2_S_2_, calc. 1230.4109, found: 1230.4066.

**HPLC** (Method B): Rt (min) 9

**IR** (solid): ν (cm^−1^) 2952, 1637, 1536, 1500, 1468, 1399, 1359, 1306, 1184, 1151, 1089, 970, 814, 732.

### 4.21 Computed logP (clogP)

Computed logP values for the set of compounds **HL1‐HL4** and corresponding metal complexes were determined from SwissADME [99, 100] with the exception of the metal complexes of **HL4** because the SMILES strings of these complexes exceeded 200 characters. For these compounds, clogP values were obtained from vcclabs (https://vcclab.org/web/alogps/) [101]. All clogP values (Table 5) appear to be within expectations overall, given the lipophilic nature of both, the BODIPY framework and the heteroaromatic groups. Unless otherwise stated, they refer to the consensus Log *P*o/w. In the case of metal complexes, only WLOGP (atomistic method from Wildman and Crippen) values were obtained. For the purposes of comparison, both computed and experimental logP values for triapine and cisplatin [102] were also provided.

**TABLE 5:** Estimated clogP values for relevant compounds and metal complexes [102].

Compound	clogP
Triapine	0.06
Cisplatin	0.06, ‐0.16^a^, ‐2.2^c^
HL1	0.94, 0.64^a^, 0.98^b^
HL2	2.50, 2.39^a^, 2.95^b^
GaL1	0.95^a^; 1.39^b^
GaL2	4.47^a^, 3.60^b^
InL1	1.33^a^; 1.39^b^
InL2	4.85^a^, 3.55^b^
BODIPY‐COOH	2.79
BODIPY‐NHS	2.44
BODIPY‐EN	2.15
HL1	2.77, 4.40^a^, 2.41^b^
HL2	3.91, 5.77^a^, 4.13^b^
GaL3	4.46^a^, 3.97^b^
GaL4	5.35^b^
InL3	4.45 ^a^, 3.97^b^
InL4	5.35^b^
FeL3	4.46^a^, 3.97^b^
FeL4	5.35^b^

^a^ WLOGP.

^b^ Value obtained from https://vcclab.org/web/alogps/.

^c^ Experimental value.

## Conflicts of Interest

The authors declare no conflict of interest.

## Supporting information




**Supporting File**: advs75212‐sup‐0001‐SuppMat.pdf.

**Supporting File**: advs75212‐sup‐0002‐Data.zip.

## Data Availability

The data that support the findings of this study are available from the corresponding author upon reasonable request.

## References

[1] Y. Zheng , H. An , J. Qi , and J. Li , “Recent Progress in Thiocarbazone Metal Complexes for Cancer Therapy via Mitochondrial Signalling Pathway,” Frontiers in Chemistry 12 (2024): 1424022, 10.3389/fchem.2024.1424022. 38873408 PMC11169589

[2] X. Jiang , L. A. Fielding , H. Davis , W. Carroll , E. C. Lisic , and J. E. Deweese , “Inhibition of Topoisomerases by Metal Thiosemicarbazone Complexes,” International Journal of Molecular Sciences 24 (2023): 12010, 10.3390/ijms241512010. 37569386 PMC10419228

[3] C. A. Kunos , E. Chu , J. H. Beumer , M. Sznol , and S. P. Ivy , “Phase I Trial of Daily Triapine in Combination With Cisplatin Chemotherapy for Advanced‐Stage Malignancies,” Cancer Chemotherapy and Pharmacology 79, no. 1 (2017): 201–207, 10.1007/s00280-016-3200-x. 27878356 PMC5226891

[4] C. A. Leath , W. Deng , L. K. Mell , et al., “Incorporation of Triapine (T) to Cisplatin Chemoradiation (CRT) for Locally Advanced Cervical and Vaginal Cancer: Results From NRG‐GY006, a Phase III Randomized Trial,” Gynecologic Oncology 195 (2025): 122–133, 10.1016/j.ygyno.2025.03.007. 40101606 PMC12009185

[5] E. S. Ratner , Y. L. Zhu , P. G. Penketh , et al., “Triapine Potentiates Platinum‐Based Combination Therapy by Disruption of Homologous Recombination Repair,” British Journal of Cancer 114 (2016): 777–786, 10.1038/bjc.2016.54. 26964031 PMC4984868

[6] C. R. Kowol , R. Trondl , V. B. Arion , M. A. Jakupec , I. Lichtscheidl , and B. K. Keppler , “Fluorescence Properties and Cellular Distribution of the Investigational Anticancer Drug Triapine (3‐Aminopyridine‐2‐Carboxaldehyde Thiosemicarbazone) and Its Zinc(II) Complex,” Dalton Transactions 39 (2010): 704–706. 20066211 10.1039/b919119b

[7] K. Pelivan , L. Frensemeier , U. Karst , et al., “Understanding the Metabolism of the Anticancer Drug Triapine: Electrochemical Oxidation, Microsomal Incubation and In Vivo Analysis Using LC‐HRMS,” Analyst 142 (2017): 3165–3176, 10.1039/C7AN00902J. 28745337

[8] M. J. Mackenzie , D. Saltman , H. Hirte , et al., “A Phase II Study of 3‐Aminopyridine‐2‐Carboxaldehyde Thiosemicarbazone (3‐AP) and Gemcitabine in Advanced Pancreatic Carcinoma. A Trial of the Princess Margaret hospital Phase II Consortium,” Investigational New Drugs 25 (2007): 553–558, 10.1007/s10637-007-9066-3. 17585372

[9] A. PopovićBijelić , C. R. Kowol , M. E. S. Lind , et al., “Ribonucleotide Reductase Inhibition by Metal Complexes of Triapine (3aminopyridine2carboxaldehyde thiosemicarbazone): A Combined Experimental and Theoretical Study,” Journal of Inorganic Biochemistry 105, no. 11 (2011): 1422–1431. 21955844 10.1016/j.jinorgbio.2011.07.003 PMC3374975

[10] A. Joshi , B. F. Kiesel , N. Chaphekar , et al., “In Vitro Evaluation of the Metabolic Enzymes and Drug Interaction Potential of Triapine,” Cancer Chemotherapy and Pharmacology 86 (2020): 633–640, 10.1007/s00280-020-04154-5. 32989483 PMC7906094

[11] C. A. Kunos and S. P. Ivy , “Triapine Radiochemotherapy in Advanced Stage Cervical Cancer,” Frontiers in Oncology 8 (2018): 149. 29868473 10.3389/fonc.2018.00149 PMC5949312

[12] P. Quach , E. Gutierrez , M. T. Basha , et al., “Methemoglobin Formation by Triapine, Di‐2‐Pyridylketone‐4,4‐Dimethyl‐3‐Thiosemicarbazone (Dp44mT), and Other Anticancer Thiosemicarbazones: Identification of Novel Thiosemicarbazones and Therapeutics That Prevent This Effect,” Molecular Pharmacology 82 (2012): 105–114, 10.1124/mol.112.078964. 22508546

[13] I. Besleaga , I. Stepanenko , T. V. Petrasheuskaya , et al., “Triapine Analogues and Their Copper (II) Complexes: Synthesis, Characterization, Solution Speciation, Redox Activity, Cytotoxicity, and mR2 RNR Inhibition,” Inorganic Chemistry 60 (2021): 11297–11319. 34279079 10.1021/acs.inorgchem.1c01275 PMC8335727

[14] M. Ismail , W. Yang , Y. Li , et al., “Biomimetic Dp44mT‐Nanoparticles Selectively Induce Apoptosis in Cu‐Loaded Glioblastoma Resulting in Potent Growth Inhibition,” Biomaterials 289 (2022): 121760, 10.1016/j.biomaterials.2022.121760. 36044788

[15] S. Krishan , S. Sahni , L. Leck , P. J. Jansson , and D. R. Richardson , “Regulation of Autophagy and Apoptosis by Dp44mT‐Mediated Activation of AMPK in Pancreatic Cancer Cells,” Biochimica et Biophysica Acta (BBA)—Molecular Basis of Disease 1866 (2020): 165657, 10.1016/j.bbadis.2019.165657. 31904416

[16] I. Doumi , L. Lang , B. Vileno , M. Deponte , and P. Faller , “Glutathione Protects Other Cellular Thiols Against Oxidation by Cu II ‐Dp44mT,” Chemistry—A European Journal 30 (2024): 202304212, 10.1002/chem.202304212. 38408264

[17] K. Malarz , A. Mrozek‐Wilczkiewicz , M. Serda , M. Rejmund , J. Polanski , and R. Musiol , “The Role of Oxidative Stress in Activity of Anticancer Thiosemicarbazones,” Oncotarget 9 (2018): 17689–17710, 10.18632/oncotarget.24844. 29707141 PMC5915149

[18] V. A. Rao , S. R. Klein , K. K. Agama , et al., “The Iron Chelator Dp44mT Causes DNA Damage and Selective Inhibition of Topoisomerase IIα in Breast Cancer Cells,” Cancer Research 69 (2009): 948–957, 10.1158/0008-5472.CAN-08-1437. 19176392 PMC7322628

[19] J. C. Yalowich , X. Wu , R. Zhang , R. Kanagasabai , M. Hornbaker , and B. B. Hasinoff , “The Anticancer Thiosemicarbazones Dp44mT and Triapine Lack Inhibitory Effects as Catalytic Inhibitors or Poisons of DNA Topoisomerase IIα,” Biochemical Pharmacology 84 (2012): 52–58, 10.1016/j.bcp.2012.03.021. 22503743 PMC3348365

[20] J. Zhou , Y. Jiang , J. Zhao , et al., “Thiosemicarbazone Derivatives Induce Apoptosis and Cell Cycle Arrest in Cancer Cells,” Cell Oncology 43, no. 3 (2020): 461–475.

[21] M. Haritha and C. H. Suresh , “Hydration Patterns of Rings in Drugs and Relationship to Lipophilicity: A DFT Study,” Journal of Computational Chemistry 43 (2022): 477–490, 10.1002/jcc.26808. 34978337

[22] Y. Yu , D. S. Kalinowski , K. Zaklina , et al., “Thiosemicarbazones From the Old to New: Iron Chelators That Are More Than Just Ribonucleotide Reductase Inhibitors,” Journal of Medicinal Chemistry 52, no. 17 (2009): 5271–5294. 19601577 10.1021/jm900552r

[23] B. M. Zeglis , V. Divilov , and J. S. Lewis , “Role of Metalation in the Topoisomerase IIα Inhibition and Antiproliferation Activity of a Series of α‐Heterocyclic‐ N^4^‐Substituted Thiosemicarbazones and Their Cu(II) Complexes,” Journal of Medicinal Chemistry 54 (2011): 2391–2398, 10.1021/jm101532u. 21391686 PMC4151564

[24] C. R. Kowol , R. Berger , R. Eichinger , et al., “Gallium(III) and Iron(III) Complexes of α‐N‐Heterocyclic Thiosemicarbazones: Synthesis, Characterization, Cytotoxicity, and Interaction With Ribonucleotide Reductase,” Journal of Medicinal Chemistry 50 (2007): 1254–1265, 10.1021/jm0612618. 17315858

[25] C. R. Kowol , E. Reisner , I. Chiorescu , et al., “An Electrochemical Study of Antineoplastic Gallium, Iron and Ruthenium Complexes With Redox Noninnocent α‐N‐Heterocyclic Chalcogensemicarbazones,” Inorganic Chemistry 47 (2008): 11032–11047, 10.1021/ic8013249. 18973290

[26] F. Bisceglie , A. Musiari , S. Pinelli , et al., “Quinoline‐2‐Carboxaldehyde Thiosemicarbazones and Their Cu(II) and Ni(II) Complexes as Topoisomerase IIa Inhibitors,” Journal of Inorganic Biochemistry 152 (2015): 10–19, 10.1016/j.jinorgbio.2015.08.008. 26335598

[27] F. Cortezon‐Tamarit , K. Song , N. Kuganathan , et al., “Structural and Functional Diversity in Rigid Thiosemicarbazones With Extended Aromatic Frameworks: Microwave‐Assisted Synthesis and Structural Investigations,” ACS Omega 8 (2023): 16047–16079. 37179648 10.1021/acsomega.2c08157 PMC10173449

[28] J. Chan , A. L. Thompson , M. W. Jones , and J. M. Peach , “Synthesis and Structural Studies of Gallium(III) and Indium(III) Complexes of 2‐Acetylpyridine Thiosemicarbazones,” Inorganica Chimical Acta 363 (2010): 1140–1149, 10.1016/j.ica.2009.10.020.

[29] S. Sarpaki , F. Cortezon Tamarit , R. M. Exner , et al., “Functional, Aromatic, and Fluorinated Monothiosemicarbazones: Investigations Into Their Structures and Activity Toward the Gallium‐68 Incorporation by Microwave Irradiation,” ACS Omega 7, no. 16 (2022): 13750–13777. 35559172 10.1021/acsomega.1c07396 PMC9088960

[30] M. Serda , D. S. Kalinowski , A. M. Mrozek‐Wilczkiewicz , et al., “Synthesis and Characterization of Quinoline‐Based Thiosemicarbazones and Correlation of Cellular Iron‐Binding Efficacy to Anti‐Tumor Efficacy,” Bioorganic & Medicinal Chemistry Letters 22 (2012): 5527–5531, 10.1016/j.bmcl.2012.07.030. 22858101

[31] W. Cao , J. Qi , K. Qian , L. Tian , Z. Cheng , and Y. Wang , “Structure−Activity Relationships of 2‑Quinolinecarboxaldehyde Thiosemicarbazone Gallium(III) Complexes With Potent and Selective Anticancer Activity,” Journal of Inorganic Biochemistry 191 (2019): 174–182, 10.1016/j.jinorgbio.2018.11.017. 30530178

[32] S. H. Cao , X. H. Chen , L. G. Chen , and J. W. Chen , “?(N)‐Heterocyclic Thiosemicarbazones: Iron Chelators that are Promising for Revival of Gallium in Cancer Chemotherapy,” Anti‐Cancer Agents in Medicinal Chemistry 16 (2016): 973–991, 10.2174/1871520616666160310142012. 26961317

[33] Z. Papadopoulos , Y. Antar , L.‐S. Dieter , F. Peeters , C. Plaza‐Sirvent , and J. Karges , “Gallium(III) Complex Induces Immunogenic Cell Death Hallmarks for Chemoimmunotherapy,” Journal of Medicinal Chemistry 68 (2025): 15980–15990, 10.1021/acs.jmedchem.5c00969. 40676809

[34] X.‐X. Peng , S. Gao , and J.‐L. Zhang , “Gallium (III) Complexes in Cancer Chemotherapy,” European Journal of Inorganic Chemistry 24 (2022): 202100953.

[35] S. B. Owusu , A. Zaher , S. Ahenkorah , D. N. Pandya , T. J. Wadas , and M. S. Petronek , “Gallium Uncouples Iron Metabolism to Enhance Glioblastoma Radiosensitivity,” International Journal of Molecular Sciences 25 (2024): 10047, 10.3390/ijms251810047. 39337531 PMC11432413

[36] T. Yang , Z. Zhang , J. Zhang , et al., “Developing a Gallium(III) Agent Based on the Properties of the Tumor Microenvironment and Lactoferrin: Achieving Two‐Agent Co‐Delivery and Multi‐Targeted Combination Therapy of Cancer,” Journal of Medicinal Chemistry 66, no. 1 (2023): 793–803, 10.1021/acs.jmedchem.2c01684. 36544423

[37] S. Gupta , N. Singh , T. Khan , and S. Joshi , “Thiosemicarbazone Derivatives of Transition Metals as Multi‐Target Drugs: A Review,” Results in Chemistry 4 (2022): 100459, 10.1016/j.rechem.2022.100459.

[38] P. A. Waghorn , M. W. Jones , M. B. M. Theobald , et al., “Shining Light on the Stability of Metal Thiosemicarbazonate Complexes in Living Cells by FLIM,” Chemical Science 4 (2013): 1–10, 10.1039/c2sc21489j.

[39] J. L. Hickey , J. L. James , C. A. Henderson , et al., “Intracellular Distribution of Fluorescent Copper and Zinc Bis(thiosemicarbazonato) Complexes Measured With Fluorescence Lifetime Spectroscopy,” Inorganic Chemistry 54 (2015): 9556–9567, 10.1021/acs.inorgchem.5b01599. 26397162

[40] A. Bergamo , A. Masi , P. J. Dyson , and G. Sava , “Modulation of the Metastatic Progression of Solid Tumours by Ruthenium Compounds,” International Journal of Oncology 33 (2008): 1281–1289. 19020762

[41] C. Wang , D. Chen , Z. Wei , J. Tan , C. Wu , and X. Zhang , “Metal‐Catalyzed Abiotic Cleavage of C═C Bonds for Effective Fluorescence Imaging of Cu(II) and Fe(III) in Living Systems,” Advanced Science 12 (2025): 2412407, 10.1002/advs.202412407. 39784410 PMC11848571

[42] M. Cini , T. D. Bradshaw , and S. Woodward , “Using Titanium Complexes to Defeat Cancer: The View From the Shoulders of Titans,” Royal Society of Chemistry 46 (2017): 1040–1051. 10.1039/c6cs00860g 28124046

[43] S. G. Giuffrida , D. G. Calatayud , F. Cortezon‐Tamarit , et al., “Functional D—and L ‐Naphthalenediimide‐Peptides: Microwave‐Driven Synthesis, Supramolecular Aggregation, and Multiphoton Fluorescence Lifetime Imaging Microscopy in Living Cells,” ACS Bio & Med Chem Au 5 (2025): 947–965, 10.1021/acsbiomedchemau.5c0006. PMC12715529 41425801

[44] P. A. Waghorn , M. W. Jones , A. McIntyre , et al., “Targeting Carbonic Anhydrases With Fluorescent BODIPYLabelled SulfonamidesEur,” European Journal of Inorganic Chemistry 2012 (2012): 2898–2907.

[45] I. S. Alam , R. L. Arrowsmith , F. Cortezon‐Tamarit , et al., “Microwave Gallium68 Radiochemistry for Kinetically Stable Bis(thiosemicarbazone) Complexes: Structural Investigations and Cellular Uptake Under Hypoxia,” Dalton Transactions 45 (2016): 144–155. 26583314 10.1039/c5dt02537k PMC4758186

[46] M. Lledos , D. G. Calatayud , F. Cortezon‐Tamarit , et al., “Tripodal BODIPY‐Tagged and Functional Molecular Probes: Synthesis, Computational Investigations and Explorations by Multiphoton Fluorescence Lifetime Imaging Microscopy,” Chemistry–A European Journal 30 (2024): 202400858. 10.1002/chem.202400858 38887133

[47] R. L. Arrowsmith , P. A. Waghorn , M. W. Jones , et al., “Fluorescent Gallium and Indium Bis(thiosemicarbazonates) and Their Radiolabelled Analogues: Synthesis, Structures and Cellular Confocal Fluorescence Imaging Investigations,” Dalton Transactions 40 (2011): 6238–6252. 21594287 10.1039/c1dt10126a

[48] M. J. Green , H. Ge , S. E. Flower , et al., “Fluorescent Naphthalimide Boronates as Theranostics: Structural Investigations, Confocal Fluorescence and Multiphoton Fluorescence Lifetime Imaging Microscopy in Living Cells,” RSC Chemical Biology 4 (2023): 1082–1095. 38033726 10.1039/d3cb00112a PMC10685793

[49] I. Džajić , T. Tomašič , L. A. Pardo , L. Peterlin Mašič , and A. E. Cotman , “Lipophilic Cations as Mitochondria‐Targeting Moieties: Recent Progress and Design Principles for Medicinal Chemistry,” Journal of Medicinal Chemistry 68, no. 22 (2025): 15980–15990. 41248604 10.1021/acs.jmedchem.5c02076 PMC12670399

[50] D. G. Calatayud , R. L. Arrowsmith , and P. A. Waghorn , Chemical Biology Imaging Tools for Chemical Biology, ed. L. Feng and T. D. James (RSC Publications, 2024), 10.1039/9781837673117-00104.

[51] C. R. Chitambar , “Gallium Compounds as Antineoplastic Agents,” Current Opinion in Oncology 16, no. 6 (2004): 547–552, 10.1097/01.cco.0000142071.22226.d2. 15627016

[52] C. R. Chitambar , W. G. Matthaeus , W. E. Antholine , K. Graff , and W. J. O'Brien , “Inhibition of Leukemic HL60 Cell Growth by Transferrin‐Gallium: Effects on Ribonucleotide Reductase and Demonstration of Drug Synergy With Hydroxyurea [see comments],” Blood 72, no. 6 (1988): 1930–1936, 10.1182/blood.V72.6.1930.1930. 3058232

[53] C. R. Chitambar , M. M. Al‐Gizawiy , H. S. Alhajala , et al., “Gallium Maltolate Disrupts Tumor Iron Metabolism and Retards the Growth of Glioblastoma by Inhibiting Mitochondrial Function and Ribonucleotide Reductase,” Molecular Cancer Therapeutics 17 (2018): 1240–1250, 10.1158/1535-7163.MCT-17-1009. 29592883 PMC5984712

[54] R. Paprocka , M. Wiese‐Szadkowska , S. Janciauskiene , T. Kosmalski , M. Kulik , and A. Helmin‐Basa , “Latest Developments in Metal Complexes as Anticancer Agents,” Coordination Chemistry Reviews 452 (2022): 214307, 10.1016/j.ccr.2021.214307.

[55] P. A. McIntyre , S. M. Larson , E. A. Eikman , M. Colman , U. Scheffel , and B. A. Hodkinson , “Clinical Evaluation of 67Gacitrate Imaging in Malignant Disease,” Journal of Nuclear Medicine 15, no. 10 (1974): 856–862. 4417862

[56] S.‐H. Zhou , W.‐H. Liao , Y. Yang , et al., “(8‐Hydroxyquinoline) Gallium(III) Complex With High Antineoplastic Efficacy for Treating Colon Cancer via Multiple Mechanisms,” ACS Omega 8 (2023): 6945–6958, 10.1021/acsomega.2c07742. 36844596 PMC9948165

[57] A. M. F. Darwesh , C. Imberti , J. J. Bartnicka , et al., “In Vivo Trafficking of the Anticancer Drug Tris(8‐Quinolinolato) Gallium (III) (KP46) by Gallium‐68/67 PET/SPECT Imaging,” Molecules 28, no. 20 (2023): 7217, 10.3390/molecules28207217. 37894695 PMC10609081

[58] H. M. Fahmy , F. M. Abdel‐Rahman , A. A. El‐Sayed , and A. A. El‐Sherif , “Study of Novel Bidentate Heterocyclic Amine‐Based Metal Complexes and Their Biological Activities: Cytotoxicity and Antimicrobial Activity Evaluation,” BMC Chemistry 17, no. 1 (2023): 78, 10.1186/s13065-023-00996-1. 37454081 PMC10349454

[59] S. N. Vorobyeva , S. A. Bautina , N. A. Shekhovtsov , et al., “Luminescent Lanthanide(III) Complexes With βdiketonate Ligands: Structure, Bonding, and Photophysical Properties,” Dalton Transactions 53, no. 19 (2024): 8398–8416. 38683023 10.1039/d4dt00824c

[60] J. R. Masters , “HeLa Cells 50 Years On: The Good, the Bad and the Ugly,” Nature Reviews Cancer 2, no. 4 (2002): 315–319, 10.1038/nrc775. 12001993

[61] O. Desiatkina , G. Boubaker , N. Anghel , et al., “Synthesis, Photophysical Properties and Biological Evaluation of New Conjugates BODIPY: Dinuclear Trithiolato‐Bridged Ruthenium(II)‐Arene Complexes,” ChemBioChem 23 (2022): 202200536. 10.1002/cbic.202200536 36219484

[62] L. H. Davies , B. B. Kasten , P. D. Benny , et al., “Re and 99m Tc Complexes of BodP_3_ – Multi‐Modality Imaging Probes,” Chemical Communications 50 (2014): 15503–15505, 10.1039/C4CC06367H. 25248386 PMC4659712

[63] V. Ćurčić , M. Olszewski , N. Maciejewska , et al., “Discovery of Novel Steroidal Inhibitors Targeting the Androgen Receptor and Epigenetic Regulators,” Archiv der Pharmazie 357, no. 2 (2024): 2300426, 10.1002/ardp.202300426.

[64] L. C. S. Pinheiro , N. Boechat , M. L. G. Ferreira , et al., “Anti‐Plasmodium Falciparum Activity of Quinoline–Sulfonamide Hybrids,” Bioorganic & Medicinal Chemistry 23, no. 17 (2015): 5979–5984, 10.1016/j.bmc.2015.06.056. 26190461

[65] S. Wang , L. Gai , Y. Chen , X. Ji , H. Lu , and Z. Guo , “Mitochondria‐Targeted BODIPY Dyes for Small Molecule Recognition, Bio‐Imaging and Photodynamic Therapy,” Chemical Society Reviews 53 (2024): 3976–4019, 10.1039/D3CS00456B. 38450547

[66] E. R. H. Walter , L. C.‐C. Lee , P. K.‐K. Leung , K. K.‐W. Lo , and N. J. Long , “Mitochondria‐Targeting Biocompatible Fluorescent BODIPY Probes,” Chemical Science 15 (2024): 4846–4852, 10.1039/D3SC06445J. 38550684 PMC10966975

[67] T. C. Salzillo , V. Mawoneke , J. Weygand , et al., “Measuring the Metabolic Evolution of Glioblastoma throughout Tumor Development, Regression, and Recurrence with Hyperpolarized Magnetic Resonance,” Cells 10, no. 10 (2021): 2621. 34685601 10.3390/cells10102621 PMC8534002

[68] M. A. Banu , A. Dovas , M. G. Argenziano , et al., “A Cell State‐Specific Metabolic Vulnerability to GPX4‐Dependent Ferroptosis in Glioblastoma,” EMBO Journal 43 (2024): 4492–4521, 10.1038/s44318-024-00176-4. 39192032 PMC11480389

[69] Z. Liang , S. Zhao , Y. Liu , and C. Cheng , “The Promise of Mitochondria in the Treatment of Glioblastoma: A Brief Review,” Discover Oncology 16, no. 1 (2025): 142, 10.1007/s12672-025-01891-y. 39924629 PMC11807951

[70] M. Holmgren and L. Sheets , “Using the Zebrafish Lateral Line to Understand the Roles of Mitochondria in Sensorineural Hearing Loss,” Frontiers in Cell and Developmental Biology 8 (2020): 628712, 10.3389/fcell.2020.628712. 33614633 PMC7892962

[71] S. B. Pickett , E. D. Thomas , J. Y. Sebe , et al., “Cumulative Mitochondrial Activity Correlates With Ototoxin Susceptibility in Zebrafish Mechanosensory Hair Cells,” eLife 8 (2019): 38062. 10.7554/eLife.38062 PMC6345563 30596476

[72] L. V. Zholudeva , K. G. Ward , M. G. Nichols , and H. J. Smith , “Gentamicin Differentially Alters Cellular Metabolism of Cochlear Hair Cells as Revealed by NAD(P)H Fluorescence Lifetime Imaging,” Journal of Biomedical Optics 20, no. 5 (2015): 051032, 10.1117/1.JBO.20.5.051032. 25688541 PMC4405084

[73] L. L. Chiu , L. L. Cunningham , D. W. Raible , E. W. Rubel , and H. C. Ou , “Using the Zebrafish Lateral Line to Screen for Ototoxicity,” Journal of the Association for Research in Otolaryngology 9, no. 2 (2008): 178–190, 10.1007/s10162-008-0118-y. 18408970 PMC2504598

[74] J. Olt , S. L. Johnson , and W. Marcotti , “In Vivo and In Vitro Biophysical Properties of Hair Cells From the Lateral Line and Inner Ear of Developing and Adult Zebrafish,” Journal of Physiology 592, no. 10 (2014): 2041–2058, 10.1113/jphysiol.2013.265108. 24566541 PMC4027864

[75] R. Esterberg , T. Linbo , S. B. Pickett , et al., “Mitochondrial Calcium Uptake Underlies ROS Generation During Aminoglycoside‐Induced Hair Cell Death,” Journal of Clinical Investigation 126, no. 9 (2016): 3556–3566, 10.1172/JCI84939. 27500493 PMC5004972

[76] K. H. Lim , S. Park , E. Han , et al., “Protective Effects of Fasudil Against Cisplatin‐Induced Ototoxicity in Zebrafish: An In Vivo Study,” International Journal of Molecular Sciences 25, no. 24 (2024): 13363, 10.3390/ijms252413363. 39769128 PMC11678128

[77] D. S. Lee , A. Schrader , M. Warchol , and L. Sheets , “Cisplatin Exposure Acutely Disrupts Mitochondrial Bioenergetics in the Zebrafish Lateral‐Line Organ,” Hearing Research 426 (2022): 108513, 10.1016/j.heares.2022.108513. 35534350 PMC9745743

[78] I. A. Okkelman , D. B. Papkovsky , and R. I. Dmitriev , “Estimation of the Mitochondrial Membrane Potential Using Fluorescence Lifetime Imaging Microscopy,” Cytometry Part A 97 (2020): 471–482, 10.1002/cyto.a.23886. 31486581

[79] M. Gooz and E. N. Maldonado , “Fluorescence Microscopy Imaging of Mitochondrial Metabolism in Cancer Cells,” Frontiers in Oncology 13 (2023): 1152553, 10.3389/fonc.2023.1152553. 37427141 PMC10326048

[80] Y. Liang , Y. Li , Q. Jiao , et al., “Axonal Mitophagy in Retinal Ganglion Cells,” Cell Communication and Signaling 22 (2024): 382, 10.1186/s12964-024-01761-0. 39075570 PMC11285280

[81] T.‐H. Yang , E. Y.‐C. Kang , P.‐H. Lin , et al., “Mitochondria in Retinal Ganglion Cells: Unraveling the Metabolic Nexus and Oxidative Stress,” International Journal of Molecular Sciences 25, no. 16 (2024): 8626, 10.3390/ijms25168626. 39201313 PMC11354650

[82] M. T. Henrich , W. H. Oertel , D. J. Surmeier , and F. F. Geibl , “Mitochondrial Dysfunction in Parkinson's Disease—A Key Disease Hallmark With Therapeutic Potential,” Molecular Neurodegeneration 18, no. 1 (2023): 83, 10.1186/s13024-023-00676-7. 37951933 PMC10640762

[83] D. Paquet , G. Plucińska , and T. Misgeld , “Visualizing Mitochondrial Dynamics In Vivo,” Methods in Enzymology 547 (2014): 151–164. 25416357 10.1016/B978-0-12-801415-8.00009-6

[84] H.‐T. C. Wong , Q. Zhang , A. J. Beirl , R. S. Petralia , Y.‐X. Wang , and K. Kindt , “Synaptic Mitochondria Regulate Haircell Synapse Size and Function,” eLife 8 (2019): 48914. 10.7554/eLife.48914 PMC6879205 31609202

[85] S. B. Pickett , E. D. Thomas , J. Y. Sebe , et al., “Mitochondrial Calcium Uptake Regulates Rapid Aminoglycosideinduced Hair cell,” Death eLife 7 (2018): 38062.

[86] L. Xu , B. Yang , H. Cheng , and S. Li , “Biomaterial Based Strategies to Modulate Mitochondrial Function in Tissue Repair,” Biomaterials 286 (2022): 121576.35598336

[87] F. Deng , M. Yan , Y. Liu , et al., “Mitochondrial Dysfunction in Neurodegenerative Diseases: Mechanisms and Therapeutic Targets,” Vitamins and Hormones 121 (2023): 319–353, 10.1016/bs.vh.2022.10.003. 36707139

[88] K. S. Gkika , D. Cullinane , and T. E. Keyes , “Metal Peptide Conjugates in Cell and Tissue Imaging and Biosensing,” Topics in Current Chemistry 380, no. 5 (2022): 30, 10.1007/s41061-022-00384-8. 35701677 PMC9197911

[89] S. W. Botchway , K. M. Scherer , S. Hook , et al., “A Series of Flexible Design Adaptations to the Nikon E‐C1 and E‐C2 Confocal Microscope Systems for UV, Multiphoton and FLIM Imaging,” Journal of Microscopy 258, no. 1 (2015): 68–78, 10.1111/jmi.12218. 25664385

[90] A. Ahmed , J. Schoberer , E. Cooke , and S. W. Botchway , “Multicolor FRET‐FLIM Microscopy to Analyze Multiprotein Interactions in Live Cells,” *Multiprotein Complexes: Methods and Protocols* (Springer Protocols, 2020): 287–301. 10.1007/978-1-0716-1126-5_16 33301124

[91] CrysAlisPro (Rigaku Oxford Diffraction, 2018).

[92] G. M. Sheldrick , “SHELXT – Integrated Space‐Group and Crystal‐Structure Determination,” Acta Crystallographica, Section A: Foundations and Advances 71 (2015): 3–8. 25537383 10.1107/S2053273314026370 PMC4283466

[93] C. B. Hübschle , G. M. Sheldrick , and B. Dittrich , “ShelXle: A Qt Graphical User Interface for SHELXL,” Journal of Applied Crystallography 44 (2011): 1281–1284, 10.1107/S0021889811043202. 22477785 PMC3246833

[94] American Type Culture Collection (ATCC)‐PC‐3, (accessed: July 2024), https://www.atcc.org/products/crl‐1435.

[95] American Type Culture Collection (ATCC)‐HeLa, (accessed: July 2024), https://www.atcc.org/products/crm‐ccl‐2.

[96] Coriell Institute for Medical Research, (accessed: July 2024), https://www.coriell.org/0/Sections/Search/Sample_Detail.aspx?Ref=AG09429&Product=CC.

[97] R. J. Geraghty , A. Capes‐Davis , J. M. Davis , et al., “Guidelines for the Use of Cell Lines in Biomedical Research,” British Journal of Cancer 111 (2014): 1021–1046, 10.1038/bjc.2014.166. 25117809 PMC4453835

[98] M. Westerfield , The Zebrafish Book: A Guide for the Laboratory Use of Zebrafish (Danio rerio), 3rd Ed. (University of Oregon Press, 1995), 385.

[99] A. Daina , O. Michielin , and V. Zoete , “SwissADME: A Free Web Tool to Evaluate Pharmacokinetics, Drug‐Likeness and Medicinal Chemistry Friendliness of Small Molecules,” Scientific Reports 7 (2017): 42717, 10.1038/srep42717. 28256516 PMC5335600

[100] A. Daina , O. Michielin , and V. Zoete , “iLOGP: A Simple, Robust, and Efficient Description of n‐Octanol/Water Partition Coefficient for Drug Design Using the GB/SA Approach,” Journal of Chemical Information and Modeling 54 (2014): 3284–3301, 10.1021/ci500467k. 25382374

[101] I. V. Tetko , J. Gasteiger , R. Todeschini , et al., “Virtual Computational Chemistry Laboratory—Design and Description,” Journal of Computer‐Aided Molecular Design 19, no. 6 (2005): 453, 10.1007/s10822-005-8694-y. 16231203

[102] J. J. Wilson and S. J. Lippard , “In Vitro Anticancer Activity of Cis‐Diammineplatinum(II) Complexes With β‐Diketonate Leaving Group Ligands,” Journal of Medicinal Chemistry 55 (2012): 5326–5336, 10.1021/jm3002857. 22606945 PMC3375352

