# Supplementary material for: Mitochondria and Neuromast Tagging With Fluorescent Gallium‐Triapine Analogues: In Cellulo MP FLIM and Zebrafish Live Imaging
Source: Adv Sci (Weinh). 2026 Apr 16:e19815. Online ahead of print. doi: 10.1002/advs.202519815 (PMC13335005; doi:10.1002/advs.202519815)
Supplement: Supplementary file 1 — Supporting File: advs75212‐sup‐0001‐SuppMat.pdf. [file ADVS-9999-e19815-s002.pdf]

Supplementary Information

Mitochondria and Neuromast Tagging with Fluorescent Gallium -Triapine Analogues: *In Cellulo* MP FLIM and Zebrafish Live Imaging

Megan J. Green [a =], Michael W. Jones [b =], Merissa Saleem [c =], Haobo Ge [a, c], Melita Tvardauskaite [c], Julia Dudzic [c], Fernando Cortezon-Tamarit [a], Rory L. Arrowsmith [a], Charareh Pourzand [c], Gabriele Kociock-Kohn [a], Nicholas H. Rees[b], Jonathan R. Dilworth [b], Stephen Faulkner [b], Stanley W. Botchway [d], David Gurevich* [c, e] and Sofia I. Pascu* [a, e]

a. Department of Chemistry, University of Bath, Claverton Down, Bath, BA2 7AY, UK, s.pascu@bath.ac.uk
b. Chemistry Research Laboratory, University of Oxford, Mansfield Road, OX2 3TA
c. Department of Life Sciences, University of Bath, Claverton Down, Bath, BA2 7AY, dbg29@bath.ac.uk
d. Central Laser Facility, Rutherford Appleton Laboratory, Research Complex at Harwell, STFC, Didcot OX11 0QX, U.K
e. Centre for Bioengineering & Biomedical Technologies (CBio), University of Bath, Bath, BA2 7AY, UK

List of contents

1. General Methods and Procedures S3
2. Additional Synthetic Methods S8
3. NMR Characterisation Data S16
4. Selected Vibrational Spectroscopy S57
5. Selected Mass Spectrometry Data S67
6. Selected HPLC traces S81
7. UV-Vis and Fluorescence Emission Data S87
8. Selected Kinetic stability assays in biocompatible media S93
9. 2-Photon TCSPC Spectroscopy of Selected BODIPY conjugates S98
10. Cellular viability assays S102
11. Selected Single Photon Confocal Fluorescence Microscopy S118
12. Selected 2-P FLIM Imaging micrographs S141
13. In vivo imaging assays S152
14. X-ray Crystallography S157
15. References S168

1. General Methods and Procedures:

Reagents were obtained from commercial sources (Acros Orgaincs, Alfa Aesar, Sigma Aldrich, Merck, ThermoFisher) and were used as received. All solvents were reagent or HPLC grade and were obtained from either VWR, Sigma Aldrich or ThermoFisher. All water was obtained from a Milli-Q purification system.

Fluorescence Spectroscopy

Fluorescence spectroscopy measurements were performed using a Perkin-Elmer luminescence spectrophotometer LS 55 or Cary Eclipse fluorescence spectrometer, using 4 mL quartz cuvettes with 10 mm path lengths. All solvents used were HPLC grade. Data was collected and processed using the FL WinLab v1.60 software and Origin 9.1.

UV-vis Spectroscopy

Electronic absorption spectroscopy (UV/Vis) was examined using a Perkin-Elmer Lambda 35 spectrometer, with spectra measured using 4 mL quartz cuvettes with 10 mm path lengths. Data was analysed using the UV WinLab software package. All measurements were performed in HPLC grade solvent, and all measurements started with a calibration with the solvent being used.

Nuclear Magnetic Resonance Spectroscopy (NMR)

^{1}H NMR, ^{13}C NMR, ^{19}F NMR, ^{11}B NMR, ^{31}P NMR, ^{1}H-^{1}H COSY, ^{1}H-^{1}H NOESY, HSQC and ^{1}H-^{13}C HSQC spectra were recorded on a Bruker 400 Avance, 400 MHz spectrometer or on Bruker 500 and 600 MHz instruments, at 298 K. Spectra were processed using MestReNova 11.0 and referenced to the residual solvent peak. Splitting patterns are identified as; singlet (s), doublet (d), triplet (t), quartet (q), pentet (p), sextet (sx) and multiplet (m), doublet of doublets (dd), doublet of triplets (dt), doublet of doublets of doublets (ddd), with chemical shifts reported in ppm.

Mass Spectrometry

Electrospray mass spectra were acquired using Bruker MicroTOF apparatus or an Agilent QTOF. Mass spectra were processed using Masshunter. All mass spectra were conducted in

HPLC grade solvents. MALDI-TOF MS spectra were recorded on a Shimadzu Axima iD Plus Performance MALDI-TOF TOF spectrometer fitted with a 6mW laser operating at 337nm. Samples were loaded on a 4 x 48 well ground steel plate. Spectra were recorded in positive ion reflectron mode, with pulsed extraction at 1400 Da and laser power 80. The instrument was calibrated with peptide standards (LaserBiolabs) of known molecular weight ranging from 757 Da to 2465 Da. MALDI-TOF MS samples were prepared as follows. The sample (approx. 5mg) was dissolved in 300 μL methanol and spotted directly onto the wells of a ground steel MALDI plate in the absence of matrix and salt. The sample was left for half an hour to dry under a cover before being inserted into the Shimadzu Axima MALDI-TOF MS instrument for analysis.

High Performance Liquid Chromatography (HPLC)

HPLC was performed using a Gilson HPLC instrument using the following methods:

Method A A 15-minute gradient method was applied using a Dionex C18 Acclaim column (5 μM, 4.6 x 150 mm) in H_2O/ MeCN containing 0.1 % TFA as mobile phases with the following conditions: flow rate 1 mL/min, 0 min 20 % MeCN; 1 min 20 % MeCN; 4 min 95 % MeCN; 11.5 min 95 % MeCN; 13.5 min 15 % MeCN, 15 min 15 % MeCN.

Method B A 35-minute gradient method was applied using a using a Dionex C18 Acclaim column (5 μM, 4.6 x 150 mm) in H_2O/ MeCN each containing 0.1 % TFA as mobile phases with the following conditions: flow rate 1 mL/min, 0 min 5% MeCN; 12 min 95 % MeCN; 26 min 95 % MeCN; 30 min 5 % MeCN, 35 min 5 % MeCN. A variant of this method was also applied (Method C: A 35-minute gradient method was applied using H_2O/ MeCN each containing 0.1 % TFA as mobile phases with the following conditions: flow rate 1 mL/min, 0 min 5% MeCN; 12 min 95 % MeCN; 26 min 95 % MeCN; 30 min 5 % MeCN, 35 min 5 % MeCN.

Method C A 20-minute gradient method was applied using a Dionex C18 Acclaim column (5 μM, 4.6 x 150 mm) in H2O/ MeCN containing 0.1 % TFA as mobile phases with the following conditions: flow rate 1 mL/min, 0 min 20 % MeCN; 1 min 20 % MeCN; 4 min 95 % MeCN; 11.5 min 95 % MeCN; 17.5 min 15 % MeCN, 20 min 15 % MeCN.

Method D was performed using a Waters C-18 column (4.6 × 250 mm) with UV/Vis detection at λobs = 254nm and 410 nm with a 1.0 mL/min gradient elution method (Solvent A: acetonitrile with 0.1 % TFA v/v, Solvent B: water with 0.1% TFA v/v): start 5 % A, gradient over 23 min reaching 95 % A, hold to 25 min at 95 % A, reverse gradient till 27 min reaching 5 % A, then hold to 30 min at 5 % A.

Method E was performed on a Hamilton-PRP1 reverse phase column, (4.1 mm x 150 mm, particle size 5 µm, SN 10843) with UV/Vis detection at λobs = 254nm and 495 nm using a 0.9 mL/min gradient elution method (Solvent A: acetonitrile with 0.1 % TFA v/v, Solvent B: water with 0.1% TFA v/v): start 5 % A, gradient till 15 min reaching 95 % A, hold to 16 min at 95 % A, reverse gradient till 18 min reaching 5 % A, then hold to 20 min at 5 % A.

General Radiolabelling experiments. The positron emitting radiotracer $[^{68}Ga]GaCl_3$ was extracted from a SnO2-based column matrix $^{68}Ge/^{68}Ga$ generator using a 0.6 M HCl solution. The eluted gallium-68 was subsequently trapped on a 30 mg/mL Strata X-C cartridge, which was already activated with 1 mL of HCl solution 0.1 M and washed with 10 mL of water. Then $[^{68}Ga]GaCl3$ was eluted from the cartridge with 0.8 mL of a THF/HCl (0.02 M) solution (98%) or Acetone/HCl (0.02 M) solution (98%) and dried for 15 minutes under a stream of nitrogen at 110°C. Radio-TLC was performed for both methods on a LabLogic's PET/SPECT radio-TLC Scanner system (LabLogic, Sheffield, UK) and a Laura software (LabLogic, Sheffield, UK). The radio-TLC was developed on C18 chromatography paper (Method 1) or on Whatman 3MM chromatography papers (Method 2) with 0.35 M ethylenediaminetetraacetic acid (EDTA) as the mobile phase. Radio-HPLC was performed on an Agilent 1100 series HPLC system (Agilent Technologies, Stockport, UK) equipped with a γ-RAM Model 3 gamma-detector (IN/US Systems Inc, Florida, USA) and a Laura 3 software (LabLogic, Sheffield, UK). A reverse gradient was applied starting with solvent A (0.1% TFA milli-Q water) at 95% and solvent B (0.1% TFA MeCN) at 5% for 2 minutes. Then, until 12 minutes solvent A went up to 5%, and then isocratic until 14 minutes, and afterwards gradient until 95% A at 16 minutes, and held to 25 minutes.

Quantum Yield estimations

Quantum yields in solutions of 500 nM concentration were calculated using the following equation:

$$\phi_F = \phi_{ref} \cdot \frac{\eta^2{}_{sample}}{\eta^2{}_{ref}} \cdot \frac{E_{sample}}{A_{sample}} \cdot \frac{A_{ref}}{E_{ref}}$$

where:

ϕ_{ref} = quantum yield of reference, η^2 = refractive index of solvents used, A = absorbance at the excitation wavelength, E = integration of emission spectra. Reference was fluorescein in 0.1M NaOH (Q.Y. = 0.95 at 495 nm)

Flash Chromatography

Flash chromatography was performed using a Biotage Isolera system equipped with 10 g, 25 g or 50 g Biotage SNAP silica cartridges or 30 g reverse-phase C18-silica cartridges.

Cell Culture and Preparation

Cells were purchased from American Type Culture Collection (ATCC) .[1, 2]. Cells were cultured at 37 °C, 5 % CO_2 and high humidity. Once confluence was greater than 70 % cells were collected. PC-3 (human prostate cancer cells) were cultured in Roswell Park Memorial Institute (RMPI) 1640 serum and HeLa in EMEM serum. Media used contained 10 % foetal calf serum (FCS), 0.5 % penicillin/streptomycin (10,000 IU mL^{-1}/10,000 $mgmL^{-1}$), and 1 % 200 mM L-glutamine. Phenol red was absent in all preparation steps. Supernatant containing dead cells and excess protein was aspirated. Live adherent cells were washed with 10 mL of phosphate buffer saline (PBS), removing any remaining media containing FCS. Cells were incubated with 6 mL trypsin solution (0.25% trypsin in PBS) for 8-10 minutes at 37 °C. After this time, 6 mL of 10 % RMPI medium was added to inactivate the trypsin, with the resultant solution centrifuged for 7 minutes (1000 rpm, room temperature), to remove any remaining dead cell matter. The supernatant liquid was aspirated and 4 mL of 10 % RMPI medium was added to the remaining cells. Cells were counted using a haemocytometer and seeded as appropriate.

In the case of AG09429 (human gingival fibroblasts), cells were purchased from the Coriell Institute for Medical Research (Camden, New Jersey) [3]. AGO9429 were cultured using the same procedure Eagle's Minimum Essential Medium (EMEM). The media contained FCS (15%), 0.5% v/v penicillin/streptomycin (10,000 IU mL^{-1}/10,000 mg mL^{-1}) and 1% v/v L-Glutamine (200 mM).

MTT Assays

Cells cultured as described above were plated (7 x 10^3 cells mL^{-1}), in a 96 well plate and were left to adhere for 48 hours at 37 °C and 5 % CO_2. Compounds were subsequently loaded at different concentrations into wells and were cultured for either 30 minutes, 24 hours, or 48 hours. Final concentrations were 250 μM, 100 μM, 50 μM, 10 μM, 1 μM, 0.5 μM, 0.1 μM, and 1 nM (1 % compound in DMSO, 99 % of 10 % FCS media). After incubation for the specified time point, supernatant was aspirated from each well, which were then washed twice with PBS. 3-(4,5-dimethylthiazol-2-yl)-2,5-diphenyltetrazolium bromide (MTT) was added to each well (0.5 mg/mL, 10 % serum free media (SFM)), followed by a three-hour incubation.

After aspiration of the MTT, 100 µL of DMSO was added to the wells. Plates were then read using a BMG LABTECH FLUOstar Optima microplate reader.
Data was obtained from 3 plates per time point and was analysed using origin 9.1 to calculate IC_{50} values. Error was reported as the standard error. #

Confocal Microscopy

Fluorescence microscopy images were captured using a Nikon eclipse TE2000 epifluorescence microscope or a *Nikon A1Rsi Laser Scanning Confocal Microscope* system fitted with 60X oil objective lens. The confocal microscope was also fitted with a motorised piezo z-stage, halogen lamp and mercury lamp for visual fluorescence microscope.

Cells were cultured as described above and plated in a glass bottom petri dish (35 mm diameter and 1.5 mm thickness) at $1.5x10^5$ – $2.5x10^5$ cells per dish and were left 48 hours for cells to adhere. Prior to microscopy observation, cultured cells were washed with PBS three times and refilled with 990 µL of serum free media. Subsequently, compounds (10 µL in DMSO) were loaded to make the final volume 1 mL, at the appropriate concentration. Final concentrations were incubated with 1 % DMSO. After the appropriate incubation time at either 37 °C or 4°C, cells were washed with PBS three times, refilled with fresh SFM (1 mL) with confocal images captured immediately afterwards. Images were processed using Nikon NIS elements-AR Analysis 4.30.02 software.

Co-localisation assays

To glass bottom petri dishes loaded with compound and washed as described above, 10 µL of desired tracking dye in 990 µL SFM was added, to give final, working concentrations as stated by the manufacturer. Dyes used were: ER-Tracker™ Red (BODIPY™ TR Glibenclamide), MitoTracker™ Red CMXRos, Invitrogen™ LysoTracker™ Red DND-99, ER-Tracker™ Green (BODIPY™ FL Glibenclamide), and Invitrogen™ MitoTracker™ Green FM. Microscope dishes were then incubated for 20 minutes at 37 °C. Plates were then washed with PBS twice, refilled with 1 mL fresh SFM and immediately imaged. Images were processed using Nikon NIS elements-AR Analysis 4.30.02 software. Manders' colocalisation coefficients were calculated from 3 random fields of view over 3 independent experiments. Error was reported as the standard deviation.

Two-photon Fluorescence Lifetime Imaging (FLIM)

For the *in vitro* time-correlated single-photon counting (TCSPC) and multiphoton fluorescence lifetimes imaging measurements, PC-3 cells were seeded in 4-well chamber slides at a density of 1 x 10^5 cells/well and cultured for 48 h to adhere fully. Cells were then treated with compounds with the final concentration at 1 μM (1% DMSO and 99% conditioned media) for 20 minutes, followed by washing with PBS. Chambers were then filled with 1 mL serum-free medium. Images were immediately recorded on a Nikon TE2000-U microscope with a single-photon excitation laser at 488 nm or 640 nm and a two-photon excitation laser of 910 or 1010 nm. Lifetime calculations were processed using SPCImage software.

2. Additional Synthetic Methods

Alternative synthesis of HL3

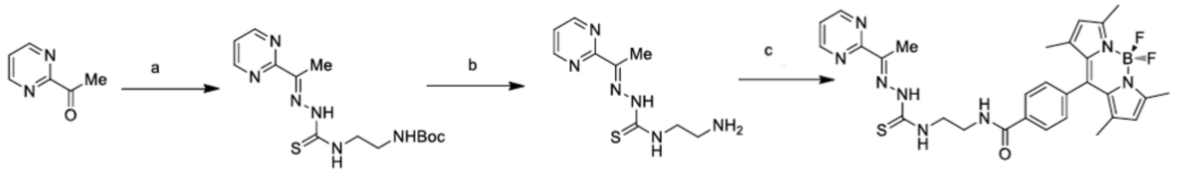

Ia **IIa** **IIIa** **HL3**

Scheme S.6. Alternative synthesis of BODIPY conjugates of pyrimidine thiosemicarbazones **HL3** Conditions/Reagents (a) $BocNHCH_2CH_2C(S)NHNH_2$, EtOH, (b) TFA, CH_2Cl_2, rt (c) BODIPY-COOH, BOP, DIPEA, DMF, 0°C→RT. Similar method was applied for the synthesis of **HL4** via this route (involving corresponding quinoline-functionalised intermediates, **Ib-IIIb**) as described below.

Boc-Ethylenediamine

Boc-ethylenediamine was synthesised according to a literature procedure [4].

Di-^{t}butyldicarbonate (8.74 g, 40.0 mmol) was dissolved in CHCl3 (200 mL). The resulting solution was added dropwise to a solution of ethylene diamine (28 ml, 428 mmol) in CHCl3 (400 mL) at 0 °C over a 3 h period. The mixture was allowed to warm to room temperature and stirred for 16 h, during which a white precipitate formed. The mixture was washed with brine (5 x 100 ml) followed by water (100 ml). The combined organics were dried over anhydrous magnesium sulphate, filtered and concentrated under reduced pressure to yield an off-white oil which solidified into a white powder over the course of several days.

Yield: 5.95 g, 37.1 mmol, 93 %.

^{1}**H NMR** (300 MHz, d6 –DMSO, 20 ºC): δ 4.85 (br. S, 1H, NHBoc), 3.10 (m, 2H, NH2CH2), 2.73 (m, 2H, CH2NH), 1.38 (s, 9H, Boc), 1.25 (br. s, 2H, NH2).

4-N-(2-^{t}Butoxycarbonylaminoethyl)-3-thiosemicarbazide

4-N-(2-^{t}Butoxycarbonylaminoethyl)-3-thiosemicarbazide was synthesised via a methyl-N-(2-tbutoxycarbonylaminoethyl)dithiocarbamate intermediate. Carbon disulphide (1.90 mL, 31.6 mmol) was added dropwise to a stirring solution of Boc-ethylenediamine (5.06 g, 31.6 mmol) and NEt_3 (4.41 mL, 31.50 mmol) in EtOH (100 mL) whilst maintaining the reaction at 25 ºC with a water bath. After stirring for 90 min, iodomethane (1.97 mL, 31.60 mmol) was added and the resulting mixture stirred for a further 2 h. The solvent was removed under reduced pressure and the residue re-suspended in EtOAc. The suspension was washed with 1 M HCl (100 mL), saturated aqueous NaHCO3 (100 mL) and water (100 mL). The organic phase was filtered, dried over anhydrous magnesium sulphate and the solvent removed under reduced pressure to give the intermediate methyl-N-(2-tbutoxycarbonylaminoethyl)dithiocarbamate as an off-white solid. 7.10 g, 28.4 mmol, 90 %.

^{1}**H NMR** (300 MHz, d6 –DMSO, 20 ºC): δ 8.33 (s, 1H, NHC=S), 4.94 (br s, 1H, NHBoc), 3.73 (dt, 2H, J = 5.5, 5.2, CH2NHC=S), 3.36 (dt, 2H, J = 6.10 5.5, CH2NHBoc), 2.53 (s, 3H, CH3S), 1.39 (s, 9H, Boc).

Methyl-N-(2-tbutoxycarbonylaminoethyl)dithiocarbamate (7.00 g, 27.9 mmol) and hydrazine monohydrate (2.20 mL, *ca.* 38.50 mmol) were dissolved in EtOH (100 mL) and heated together under reflux for 2 h. The solvent was removed under reduced pressure, the residue re-dissolved in CHCl3 and passed through a short plug of silica. The silica was washed with CHCl3 before the desired compound was washed through with MeOH. The MeOH fraction was evaporated to give an oil which solidified over a few days. Yield: 6.24 g, 26.8 mmol, 96 %.

^{1}**H NMR** (300 MHz, CDCl3, 20 ºC): δ 7.76 (br s, 1H, NHNH2), 7.58 (br s, 1H, CH2NHCS), 4.98 (br s, 1H, CH2NHBoc), 3.76 (br. s, 2H, NHNH2), 3.69 (m, 2H, CH2NHCS), 3.32 (m, 2H, CH2NHBoc), 1.38 (s, 9H, Boc). ^{13}**C{^{1}H} NMR** (75.5 MHz, CDCl3, 20 ºC): δ 182.6, 156.6, 79.6, 44.4, 40.1, 28.3. **Mass Spectrum** ESI-MS calcd for C8H18NaN4O2S [M + Na]+ 257.1048, found 257.1040.

Compound II (denoted Pyr-enBoc)

A mixture of precursor I (0.050 g, 0.224 mmol) and Boc-protected ethylene diamine thiosemicarbazide (0.072 g, 0.448 mmol) in MeCN (5 mL) was heated at reflux with stirring for 30 h. The mixture was then concentrated to dryness in vacuo to afford an off-white solid. This was purified using flash chromatography with 1:3 Et_2O/CH_2Cl_2 as eluent, to afford the desired product as a colourless powder (0.106 g, 0.216 mmol, 96 %). ^1H NMR (300 MHz, $CDCl_3$, 25 °C): 1.44 (12H, s, Boc), 1.59 (3H, br. s., CH_3), 3.46 (2H, q, J = 6.0 Hz, CH_2NHC=S), 3.86 (2H, q, J = CH_2NHBoc), 4.96 (1H, br. s., NHBoc), 7.33 (1H, t, J = 5.0 Hz, pyrim-CH), 8.26 (1H, br. s., CH_2NHC=S), 8.91 (2H, d, J = 5.0 Hz, 2×pyrimid-CH), 13.83 (1H, br. s., NHN=C). ^{13}C NMR (75.5 MHz, d_6 –DMSO, 25 °C): Mass Spectrum ESI-MS calcd for $C_{14}H_{22}N_6O_2S$ $[M + Na]^+$ 361.1417, found 361.1414.

Compound **IIb** (denoted Quin-enBoc)

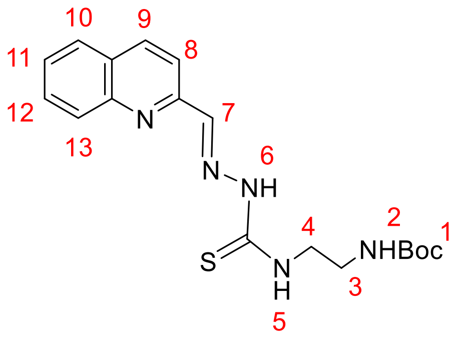

4-N-(2-tbutoxycarbonylaminoethyl)-3-thiosemicarbazide (0.72 mol, 0.17 g) was added to a stirring solution of quinoline-2-carboxaldehyde (0.72 mmol, 0.11 g) in EtOH (10 mL). A drop of conc. HCl was added and the mixture heated under reflux for 4 hours under an atmosphere of N_2. The solvent was reduced to ca. 1 mL and excess MilliQ water was added to give an orange precipitate. This was isolated by filtration washed with copious amounts of water and dried under reduced pressure. Yield: 0.23 g, 96%.

^1H NMR (300 MHz, d_6-DMSO, 25 °C): 11.94 (s, 1H, H6), 8.91 (t, 1H, J=4.7 Hz, H5), 8.43 (dd, 2H, J = 8.8 Hz, J = 21.6 Hz, H8, H9), 8.24 (s, 1H, H7), 8.02 (dd, 2H, J = 2.4 Hz, J = 8.3 Hz H10, H13), 7.78 (ddd, 1H, J = 1.4 Hz, J = 6.9 Hz, J = 8.4 Hz, H11), 7.63 (ddd, 1H J = 1.3 Hz, J = 7.2 Hz, J = 7.9 Hz, H12), 7.10 (t, 1H, J = 5.5 Hz, H2), 3.60 (m, 2H, H4), 3.23 (m, 2H, H3), 1.38 (s, 9H, Boc)

$^{13}C\{^1H\}$ (75 MHz, d$_6$-DMSO, 25 °C) 177.50 (C=S), 156.36 (C=O), 153.72 (C=Nquin), 147.15 (Cq), 142.03 (C-H7), 136.30 (C-H12), 130.03, 128, 127.94. 127.77. 127.18 (Cq), 117.95, 77.95 (Cq-^{t}Bu), 44.66 (C-H14), 38.99 (C-H3), 28.15 (boc-(CH3)3);

HPLC (0.1% TFA) Rt = 13.7 min

HR-MS$^+$ m/z = 396.1451 [M+Na$^+$] Calc m/z = 396.1465

Elemental analysis: Found C: 57.83%, 6.14%, N 18.71%; calc C18H23N5O2S: C 57.89%, H 6.21%, N 18.75%.

Synthesis of 4,4-Difluoro-8-(4'-carboxyphenyl)-1,3,5,7-tetramethyl-4-bora-3a,4a-diaza-s-indacene (BODIPY-COOH)

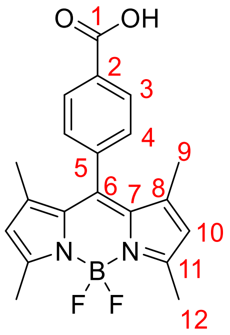

Adapted from a modified literature procedure.[6] 2,4-Dimethyl pyrrole (641 µL, 5.25 mmol) and 4-formylbenzoic acid were dissolved in DCM (200 mL) and stirred at room temperature under an argon atmosphere. After 5 minutes TFA (75 µL) was added to the solution which was left to stir for 2 hours. 2,3-Dichloro-5,6-dicyano-1,4-benzoquinone (DDQ) (510 mg, 2.24 mmol) was then added portion-wise and the solution was left to stir for 45 minutes. Triethylamine (5 mL) was subsequently added followed by addition of $BF_3.OEt_2$ (5 mL) 15 minutes later, with the reaction then left stirring for a further 2 hours. The reaction was quenched with water (250 mL) and the aqueous phase was extracted with DCM (3 x 50 mL). The organic phase was dried with $MgSO_4$ and the solvent removed under vacuum. The crude mixture was purified by flash chromatography using DCM/MeOH (0-20 %). After recrystallisation with THF/hexane the product was obtained as a red solid (0.314 g, 38%).

^{1}H NMR (400 MHz, $(CD_3)_2SO$, 298 K): δ 8.10 (d, *J* = 8.2 Hz, 2H, H-3), 7.53 (d, *J* = 8.2 Hz, 2H, H-4), 6.20 (s, 2H, H-10), 2.46 (s, 6H, H-12), 1.33 (s, 6H, H-9)

^{19}F NMR (376 MHz, $CDCl_3$) δ -146.25 (dd, *J* = 65.6, 32.6 Hz).

^{11}B NMR (128 MHz, $CDCl_3$) δ 0.75 (t, *J* = 33.2 Hz),

$^{13}C\{^1H\}$ NMR (100 MHz, $(CD_3)_2SO$, 298 K): δ 166.8 (C-1), 155.3 (C-11), 142.6 (C-5), 138.4 (C-6), 131.5 (C-7), 130.3 (C-2), 130.1 (C- 3), 128.4 (C-4), 121.6 (C-10), 14.2, 14.1 (C-9, C-12)
ESI^--MS: $[M-H]^-$ $C_{20}H_{19}BF_2N_2O_2$ calc. 367.1438, found: 367.1431.
HPLC (Method B): Rt (min) 11.52.

Synthesis of 2,5-dioxopyrrolidin-1-yl 4-(5,5-difluoro-1,3,7,9-tetramethyl-5H-4l4,5l4-dipyrrolo[1,2-c:2',1'-f][1,3,2]diazaborinin-10-yl)benzoate (BODIPY-NHS)

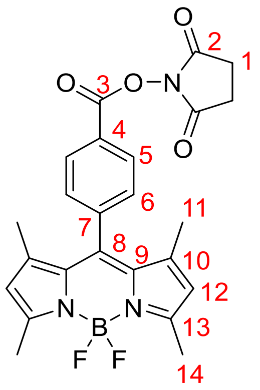

Compound **BODIPY-COOH** (400 mg, 1.09 mmol), N-hydroxysuccinimide (189 mg, 1.65 mmol) and EDCl (420 mg, 2.20 mmol) were dissolved in DCM (20 mL) and stirred at room temperature for 2 hours. The solvent was removed under vacuum and the residue purified by flash column chromatography using hexane/ethyl acetate (1:1) as eluent. The product was obtained as a crystalline orange solid (500 mg, 98 %).

^1H NMR (400 MHz, $CDCl_3$, 298 K): δ 8.27 (d, J = 8.5 Hz, 2H, H-5), 7.50 (d, J = 8.5 Hz, 2H, H- 6), 6.01 (s, 2H, H-12), 2.95 (s, 4H, H-1), 2.56 (s, 6H, H-14), 1.38 (s, 6H, H-11)
$^{13}C\{^1H\}$ NMR (100 MHz, $CDCl_3$, 298 K): δ 169.3 (C-2), 161.3 (C-3), 156.2 (C-13), 143.1 (C-10), 142.2(C- 8), 139.3 (C-7), 131.4 (C-5), 130.8 (C-4), 129.2 (C-6), 125.9 (C-9), 121.8 (C-12), 25.8 (C-1), 14.9 (C- 14), 14.7 (C-11).
^{11}B NMR (128 MHz, $CDCl_3$) δ 0.73 (t, J = 32.4 Hz).
^{19}F NMR (376 MHz, $CDCl_3$) δ -146.24 (dd, J = 65.4, 32.3 Hz).
ESI-MS: **$[M+H]^+$** $C_{24}H_{22}BF_2N_3O_4$ calc. 465.1671, found: 465.1670.
HPLC (Method B): Rt (min) 6.76. (Method C): Rt (min) 14.52.

Synthesis of 4,4-Difluoro-8-(40-((200-aminoethylcarbamoyl)methoxy))phenyl-1,3,5,7-tetramethyl-2,6-diethyl-4-bora-3a,4a-diaza-s-indacene (BODIPY-En)

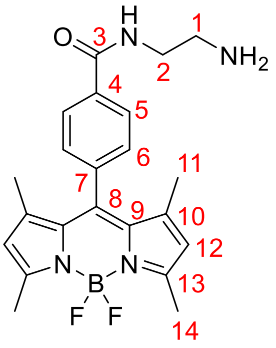

Compound **BODIPY-NHS**(650 mg, 1.40 mmol) and ethylenediamine (4.5 mL, 55.7 mmol) were suspended in DCM (30 mL) and reacted under reflux at 45 °C for 2 hours. The reaction mixture was then washed with water and the organic phase was extracted (5 x 20 mL DCM) and dried over $MgSO_4$, with the solvent then removed by vacuum. The product was purified by flash chromatography with DCM/MeOH (0-25 %) to afford an dark red solid (498 mg, 87 %).

^{1}H NMR (400 MHz, $CDCl_3$, 298 K): δ 7.97 (d, *J* = 11.0 Hz, 2H, H-5), 7.36 (d, *J* 11.0 Hz, 2H, H-6), 7.14 (m, 1H, NH), 5.97 (s, 2H, H-12), 3.57 (q, *J* = 7.7 Hz, 2H, H-2), 3.04 (t, *J* = 7.9 Hz, 2H, H-1), 2.54 (s, 6H, H-14), 1.33 (s, 6H, H-11).

ESI-MS: $[M+H]^+$ $C_{22}H_{25}BF_2N_4O$ calc. 411.2169, found: 411.2168

HPLC (Method B): Rt (min) 4.77.

(E)-4-(5,5-difluoro-1,3,7,9-tetramethyl-5H-4l4,5l4-dipyrrolo[1,2-c:2',1'-f][1,3,2]diazaborinin-10-yl)-N-(2-(2-(1-(pyrimidin-2-yl)ethylidene)hydrazine-1-carbothioamido)ethyl)benzamide (HL3)

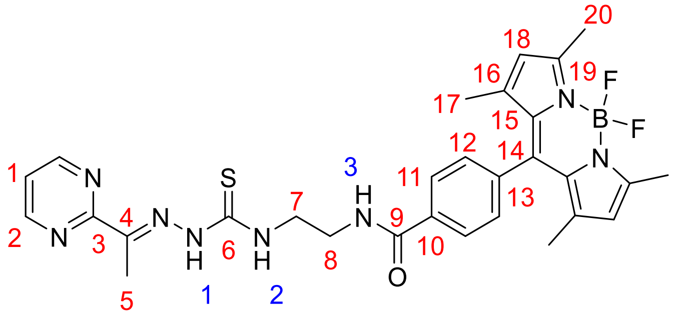

Method 1: General procedure for amide coupling

DIPEA (1.5 mol. eqv.) was added to a stirred solution of **BODIPY-COOH** (1.0 mol. eqv.) in DMF (5 mL) and the solution cooled to 0 ºC in an ice bath. BOP (1.5 mol. eqv.) was added and

the solution stirred at 0 ºC for 30 min. Amine 'III' (1.2 mol. eqv.) was added and the solution was allowed to warm to room temperature and stirred for 12 h. The DMF was removed under reduced pressure. The crude product was dissolved in $CHCl_3$ (25 mL) and washed with 1M HCl (20 mL), saturated aqueous sodium bicarbonate (100 mL), water (100 mL), brine (100 mL) and dried over anhydrous magnesium sulphate. The chloroform was removed under reduced pressure and the crude residue was purified by silica gel chromatography using DCM/MeOH (0-10 %) in very low yield (<10%).

Method 2: Compound **HL1** (130 mg, 0.58 mmol) and compound **BODIPY-En** (300 mg, 0.73 mmol) were suspended in MeCN (30 mL) and refluxed for 16 hours at 90 °C. The resulting solution was dried under vacuum and the residual solid redissolved in the minimum amount of DCM. Flash chromatography was used to purify the crude mixture using DCM/MeOH (0-10 %). Product was obtained as a red solid (200 mg, 58 %).

^{1}H NMR (400 MHz, $(CD_3)_2SO$, 298 K): δ 13.97 (s, 1H, NH-1), 895-8.91 (m, 2H, H-11), 8.81 (d, *J* = 4.9 Hz, 1H, H-2), 8.25 (t, *J* = 6.5 Hz, 1H, NH-2), 8.05 (d, *J* = 8.2 Hz, 1H, H-2), 7.83 (t, *J* = 6.5 Hz, 1H, NH-3), 7.39 – 7.34 (m, 3H, H-1/12), 5.96 (s, 2H, H-18), 4.19 – 4.11 (m, 2H, H-7), 3.81-3.77 (m, 2H, H-8), 2.55 (s, 6H, H-20), 2.47 (s, 3H, H-5), 1.34 s, 6H, H-17).
ESI-MS: $[M+H]^+$ $C_{29}H_{31}BF_2N_8OS$, calc. 589.2840, found: 589.2480.
^{19}F{^{1}H} NMR (376 MHz, $(CD_3)_2SO$, 298 K): δ -146.26.
^{11}B{^{1}H} NMR (128 MHz, $(CD_3)_2SO$, 298 K): δ 0.77 (t, *J* = 33.0 Hz).
HPLC (Method C): Rt (min) 6.63
IR (solid): ν (cm^{-1}) 2962, 2920, 1645, 1543, 1505, 1466, 1407, 1306, 1193, 1150, 1093.

(E)-4-(5,5-difluoro-1,3,7,9-tetramethyl-5H-4l4,5l4-dipyrrolo[1,2-c:2',1'-f][1,3,2]diazaborinin-10-yl)-N-(2-(2-(quinolin-2-ylmethylene)hydrazine-1-carbothioamido)ethyl)benzamide (HL4)

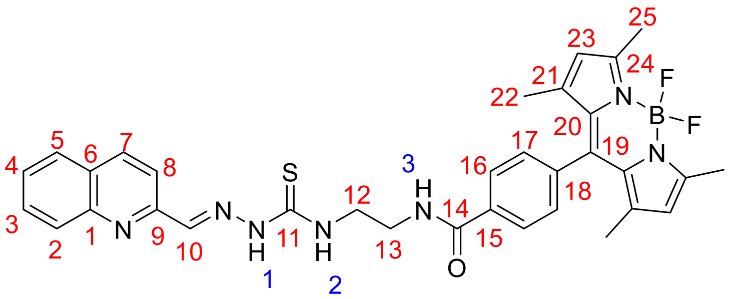

Compound **HL2** (125 mg, 0.48 mmol) and compound **BODIPY-En** (224 mg, 0.55 mmol) were suspended in MeCN (30 mL) and refluxed for 16 hours. The resultant solution was dried under vacuum and the residual solid redissolved in DCM. Flash chromatography was used to purify the crude mixture using DCM/MeOH (0-10 %). Product was obtained as a red solid (195 mg, 65 %).

^{1}H NMR (400 MHz, $(CD_3)_2SO$, 298 K): δ 11.95 (s, 1H, NH-1), 9.07 (t, J = 5.3 Hz, 1H, NH-2), 8.94 (t, J = 5.6 Hz, 1H, NH-3), 8.53 (d, J = 8.7 Hz, 1H, H-8), 8.40 (d, J = 8.7 Hz, 1H, H-7), 8.24 (s, 1H, H-10), 8.13 – 8.09 (m, 2H, H-16), 8.02 – 7.97 (m, 2H, H-2), 7.79 – 7.76 (m, 1H, H-4), 7.64 – 7.60 (m, 1H, H-3), 7.54 – 7.51 (m, 2H, H-17), 6.16 (s, 2H, H-23), 3.80 (q, J = 5.9 Hz, 2H, H-13), 3.60 (q, J = 5.8 Hz, 2H, H-12), 2.45 (s, 6H, H-25), 1.31 (s, 6H, H-22).

^{19}F{^{1}H} NMR (376 MHz, $(CD_3)_2SO$, 298 K): δ -145.86

^{11}B{^{1}H} NMR (128 MHz, $(CD_3)_2SO$, 298 K): δ 0.75 (t, J = 33.0 Hz).

ESI-MS: $[M+H]^+$ $C_{33}H_{32}BF_2N_7OS$ calc. 624.2525, found: 624.2525.

HPLC (Method C): Rt (min) 6.11.

IR (solid): ν (cm^{-1}) 2970, 2920, 1655, 1540, 1502, 1476, 1410, 1306, 1190, 1153, 1090.

3. NMR Characterisation Data

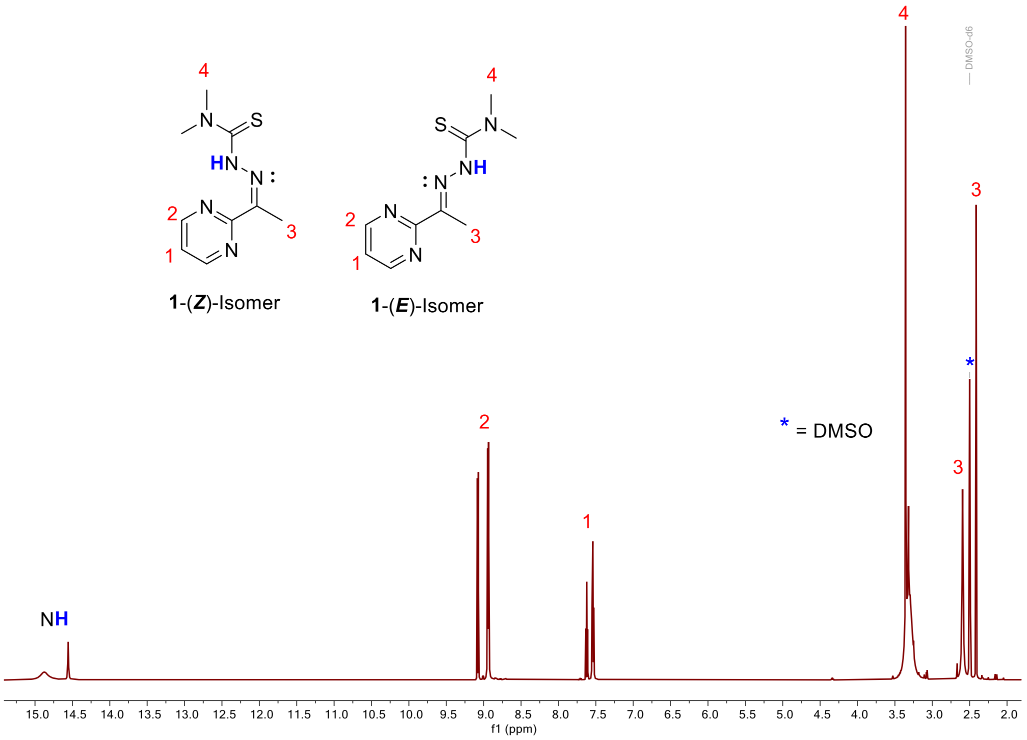

Figure S.1. ^{1}H NMR spectrum (400 MHz, $(CD_3)_2SO$, 298 K) of compound **HL1**.

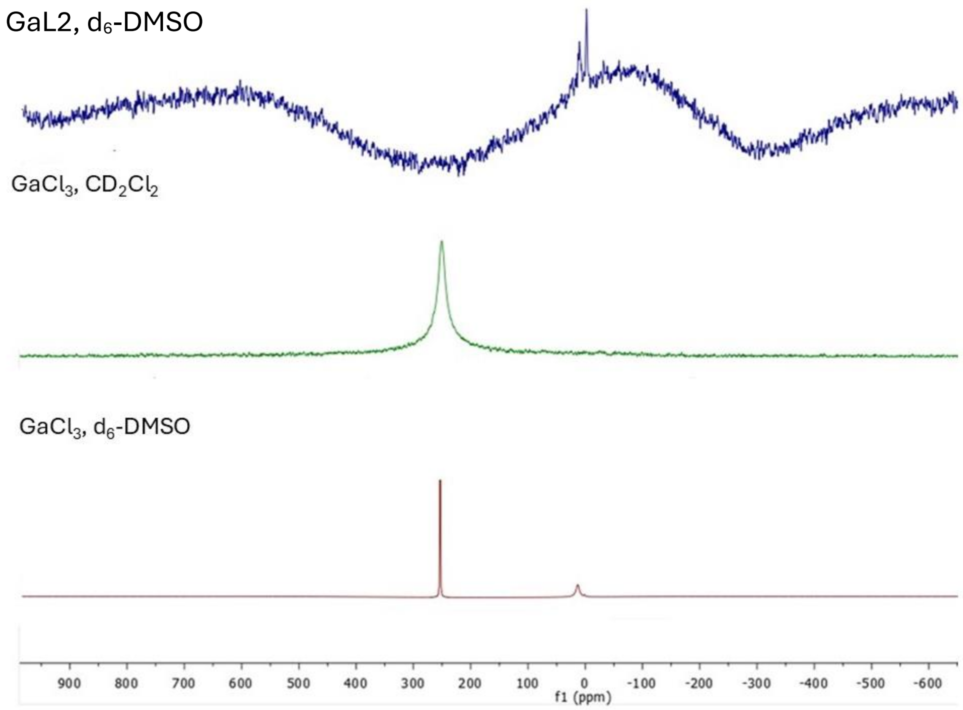

Figure S.2. ^{71}Ga NMR spectroscopy of compound **GaL2** in d$_6$-DMSO and a comparison with free Ga(III) NMR spectroscopies in d$_6$-DMSO and CD_2Cl_2.

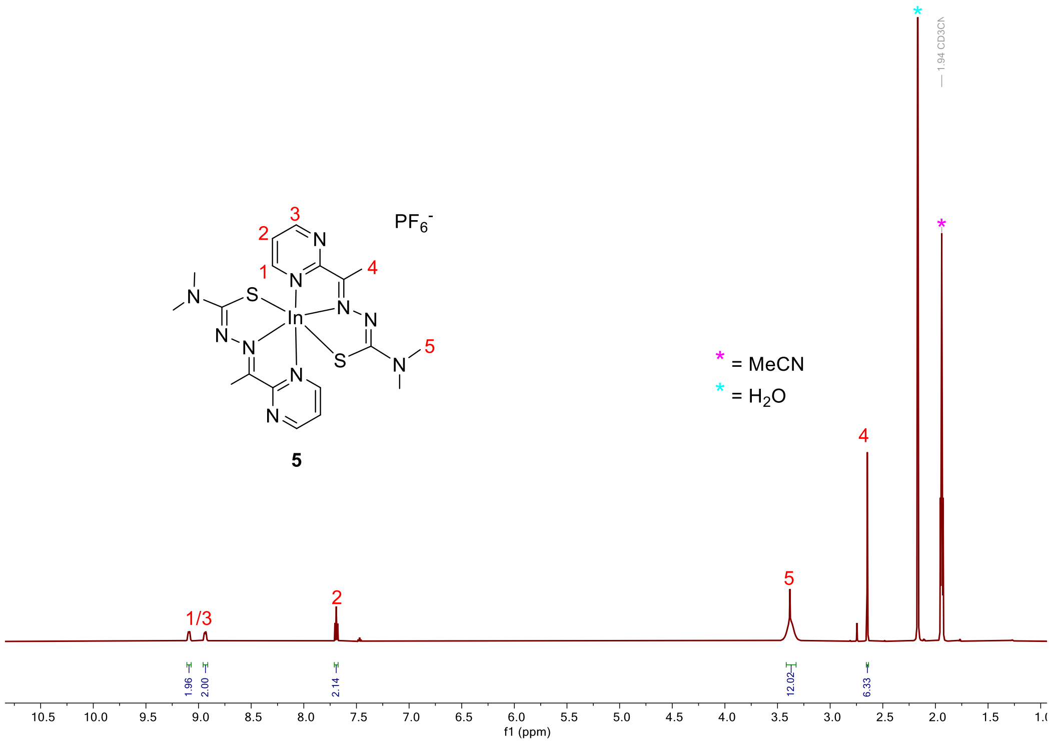

Figure S.3. ^{1}H NMR spectrum (400 MHz, CD_3CN, 298 K) of complex **InL1**.

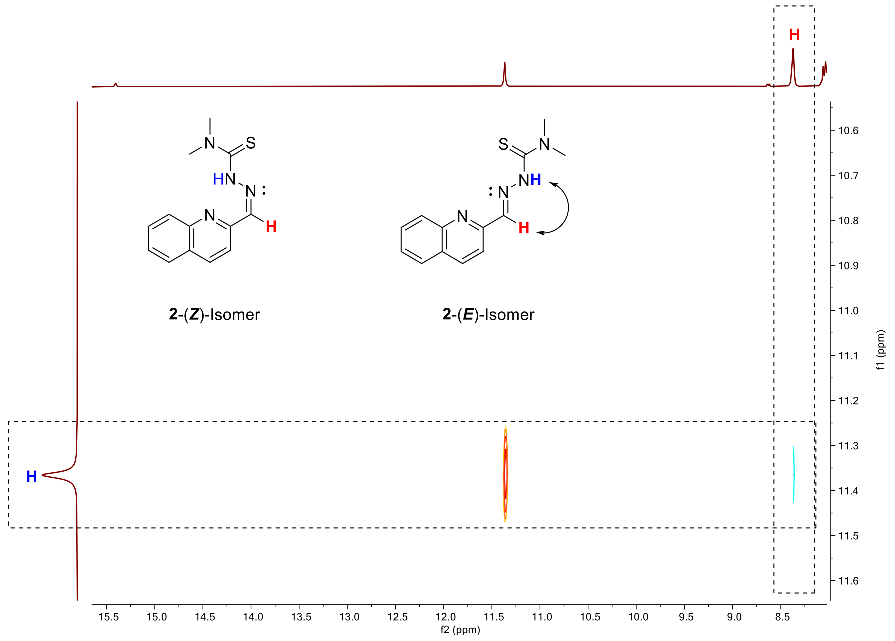

Figure S.4. ^{1}H-^{1}H NOESY NMR spectrum (400 MHz, $(CD_3)_2SO$, 298 K) of compound **HL2**.

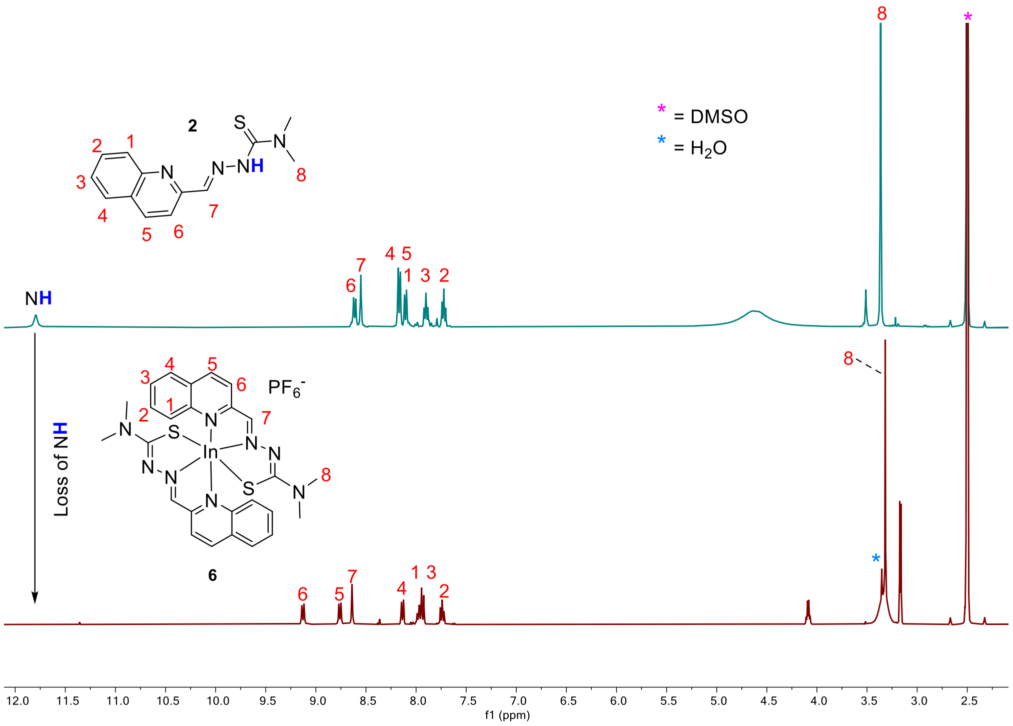

Figure S.5. ^{1}H NMR spectra (400 MHz, $(CD_3)_2SO$, 298 K) of: (top) compound HL**2**, (bottom) complex **InL2**.

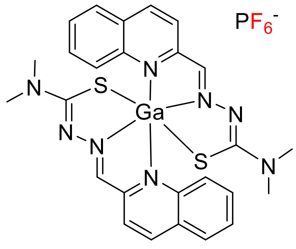

4

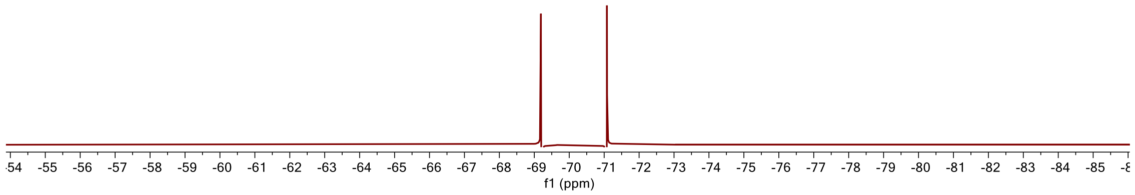

Figure S.6. ^{19}F NMR spectrum (376 MHz, $(CD_3)_2SO$, 298 K) of compound **GaL2**.

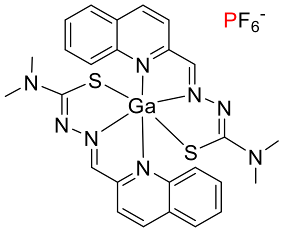

4

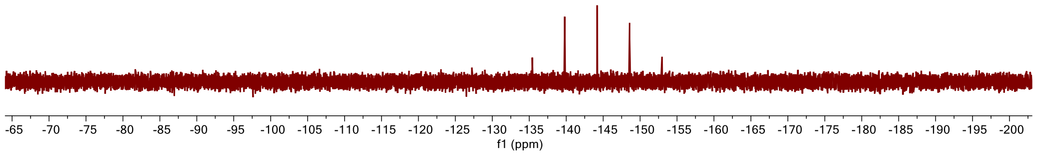

Figure S.7. ^{31}P NMR spectrum (162 MHz, $(CD_3)_2SO$, 298 K) of compound **GaL2**.

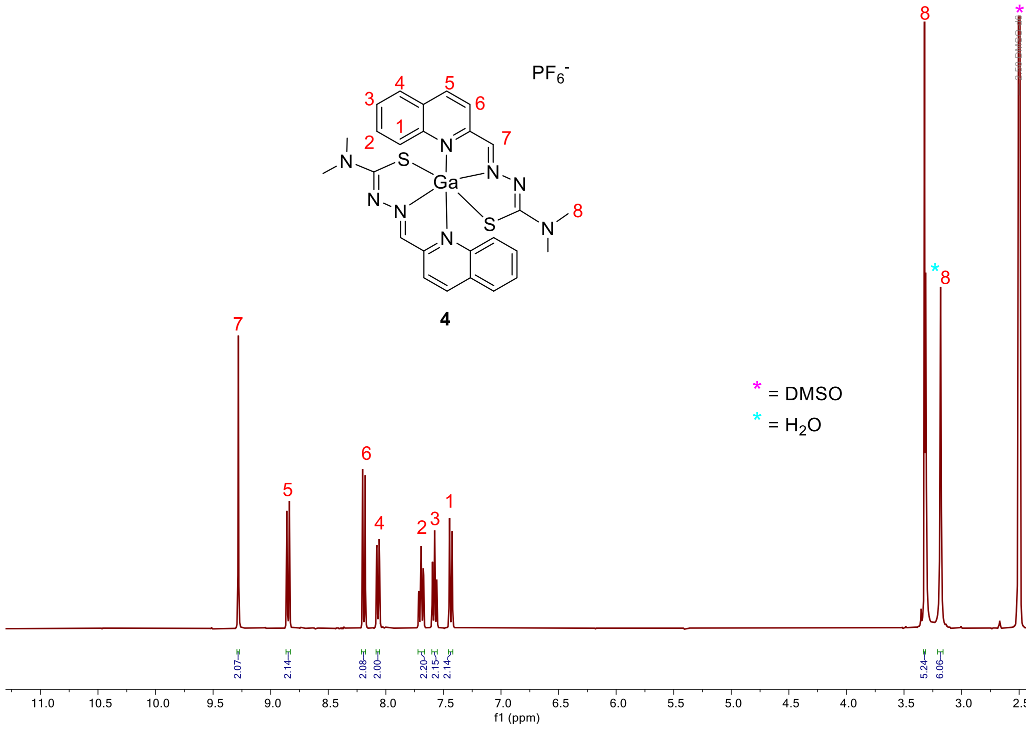

(a)

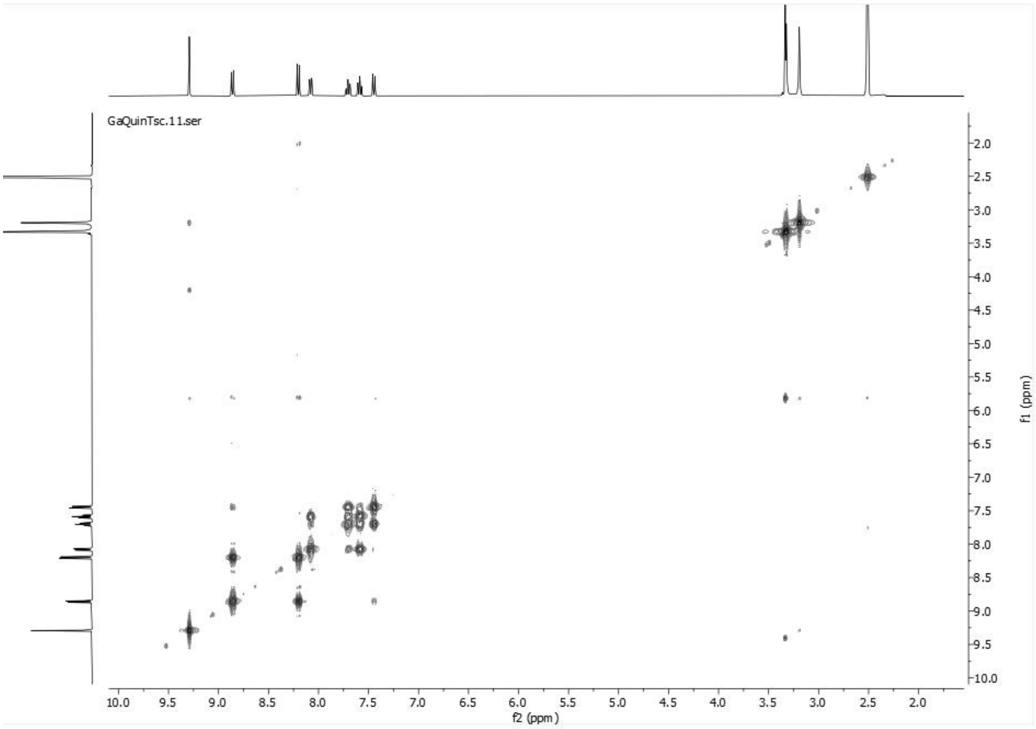

(b)

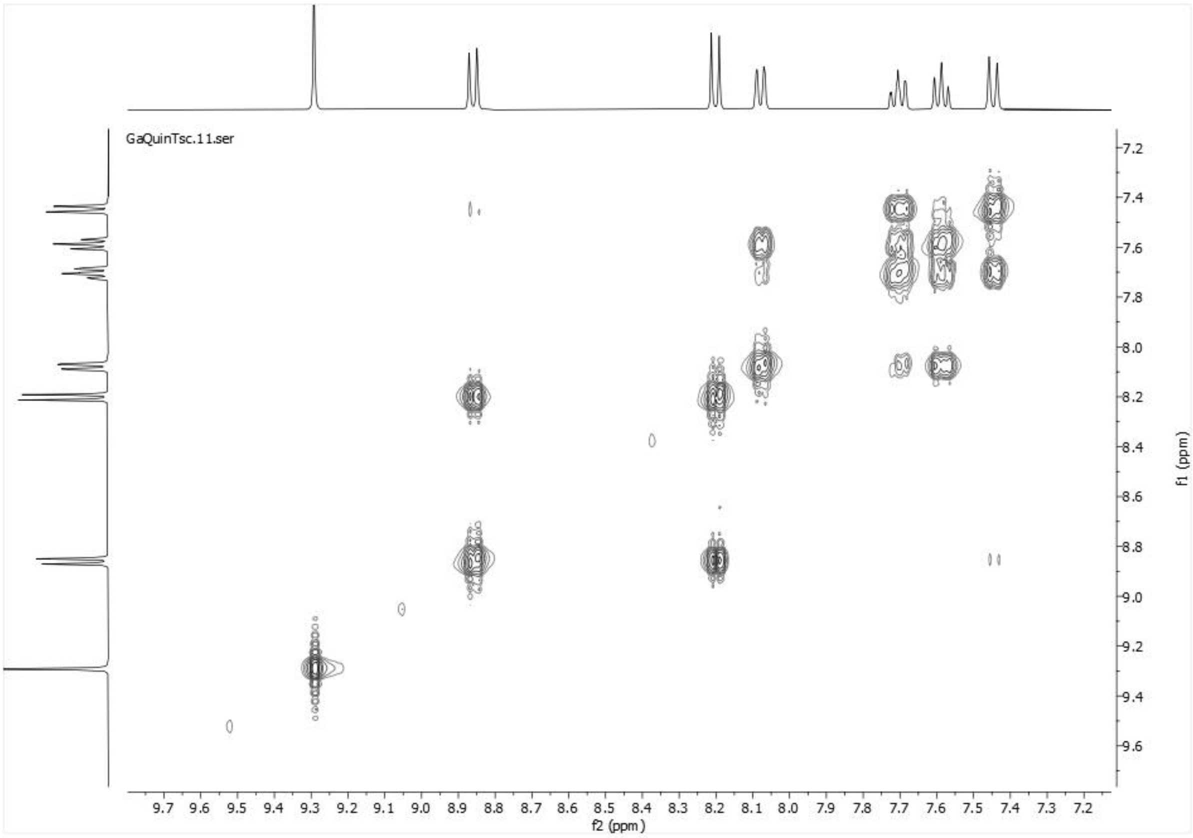

(c)

Figure S.8. (a) ^{1}H NMR spectrum (400 MHz, $(CD_3)_2SO$, 298 K) of complex **GaL2**; **(b)** and **(c)**: ^{1}H-^{1}H COSY of complex **GaL2**.

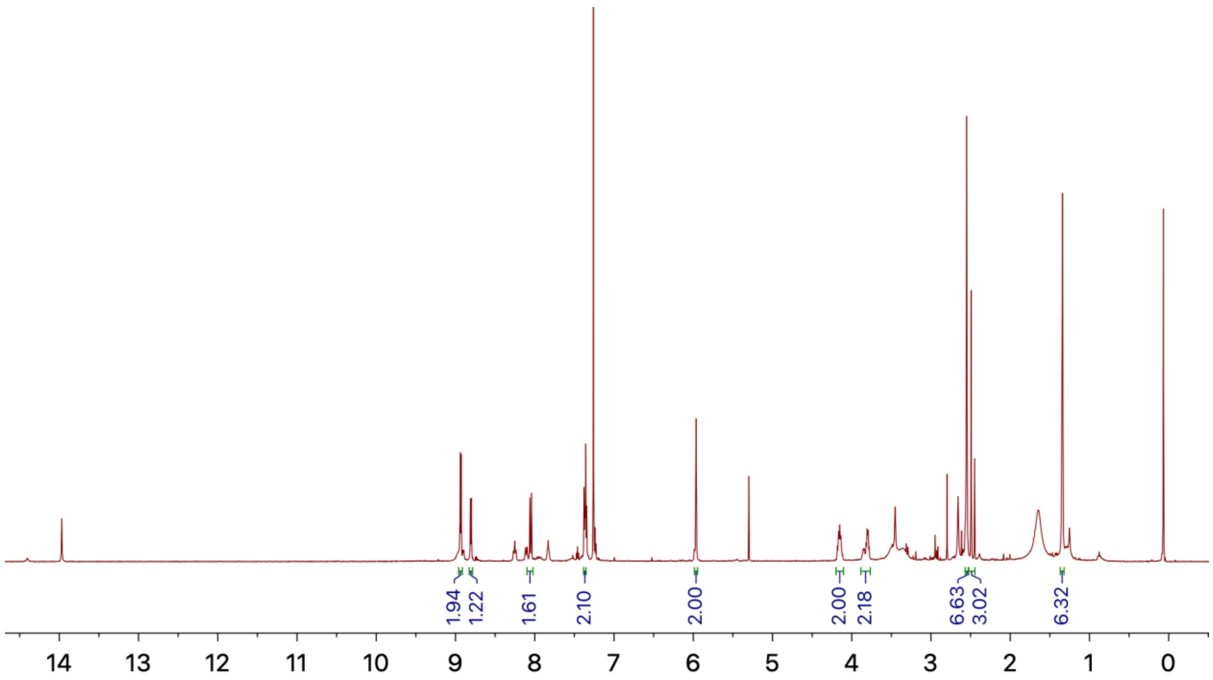

1.94
1.22
1.61
2.10
2.00
2.00
2.18
6.63
3.02
6.32
14 13 12 11 10 9 8 7 6 5 4 3 2 1 0

(a)

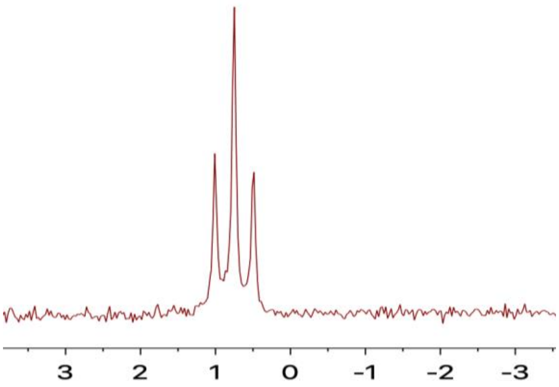

3 2 1 0 -1 -2 -3

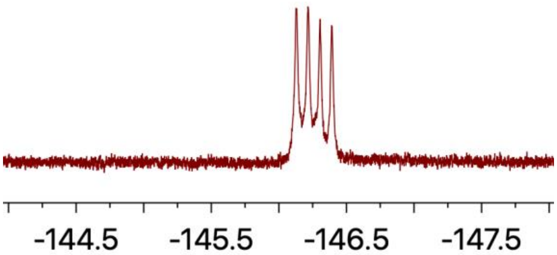

-144.5 -145.5 -146.5 -147.5

(b) **(c)**

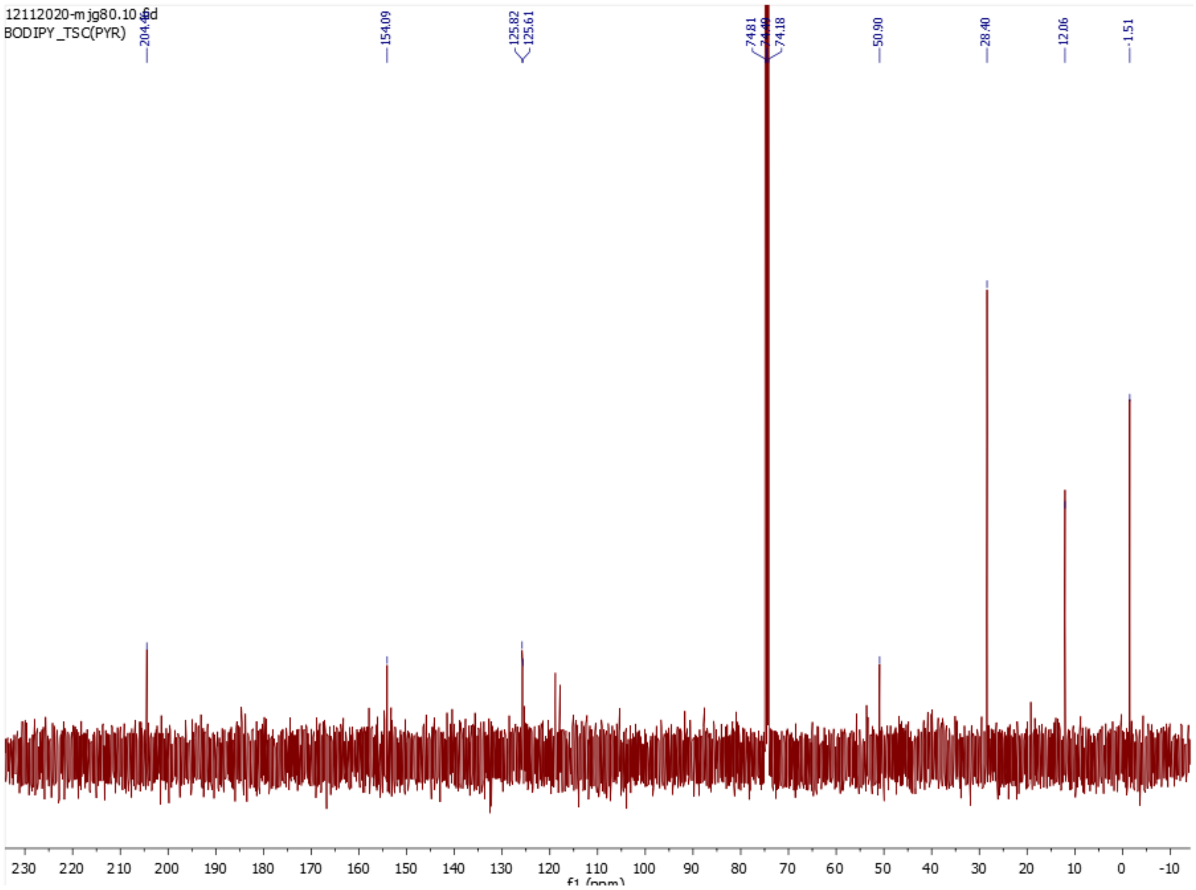

12112020-mjg80.10.fid
BODIPY_TSC(PYR)
204.46
154.09
125.82
125.61
74.81
74.49
74.18
50.90
28.40
12.06
-1.51
230 220 210 200 190 180 170 160 150 140 130 120 110 100 90 80 70 60 50 40 30 20 10 0 -10
f1 (ppm)

(d)

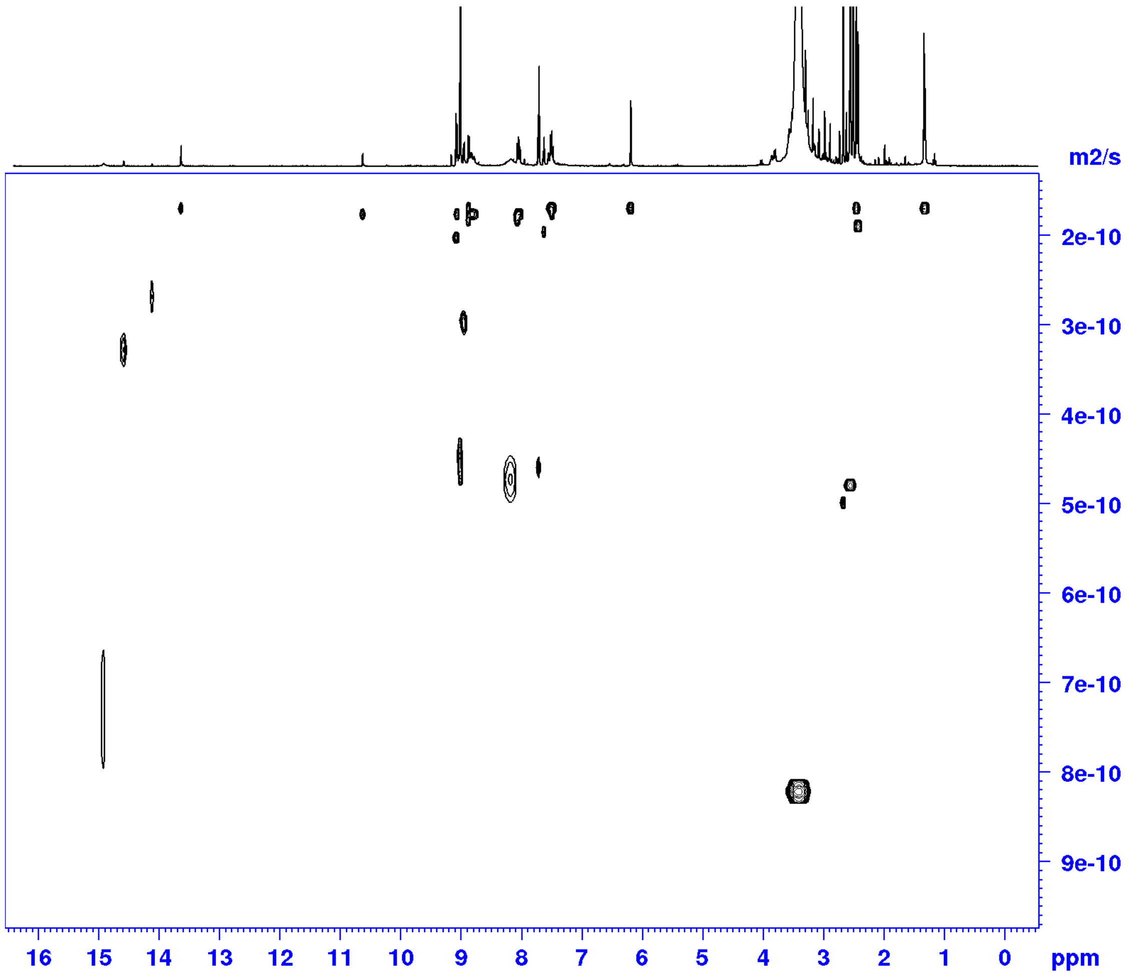

(e)

Figure S.9. (a) ^{1}H NMR spectrum (400 MHz, $CDCl_3$, 298 K) of complex **HL3**; **(b)** ^{11}**B NMR of HL3,** (128 MHz**,** $CDCl_3$, 298 K) **(c)** ^{19}F NMR spectrum (376 MHz, $CDCl_3$, 298 K) and **(d)** $^{13}C\{^{1}H\}$NMR in Methanol-D_4 of compound **HL3**; **(e)** DOSY spectroscopy of **HL3**, d_6-DMSO.

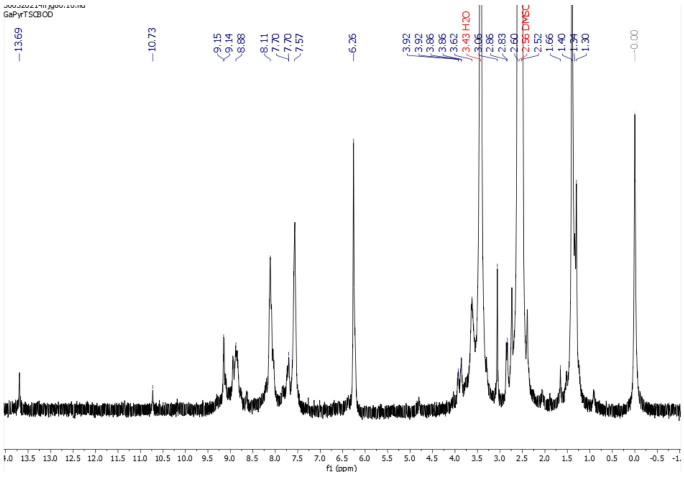
GaPyrTSCBOD
f1 (ppm)

(a)

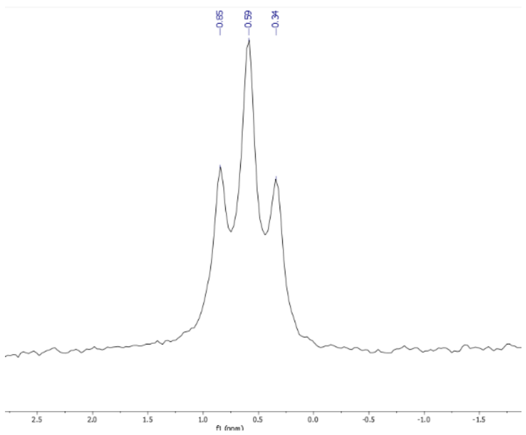

(b)

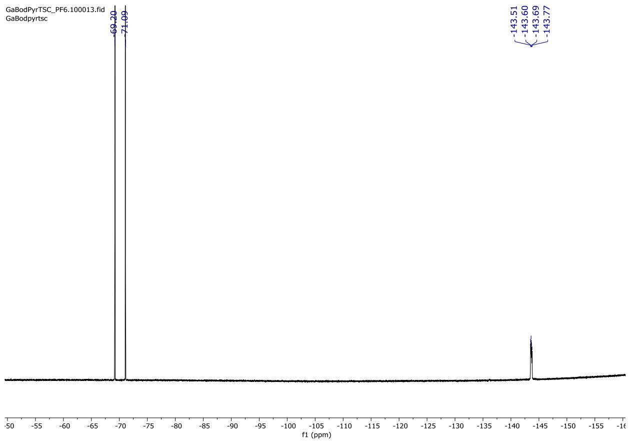
GaBodPyrTSC_PF6.100013.fid
GaBodpyrtsc
f1 (ppm)

(c)

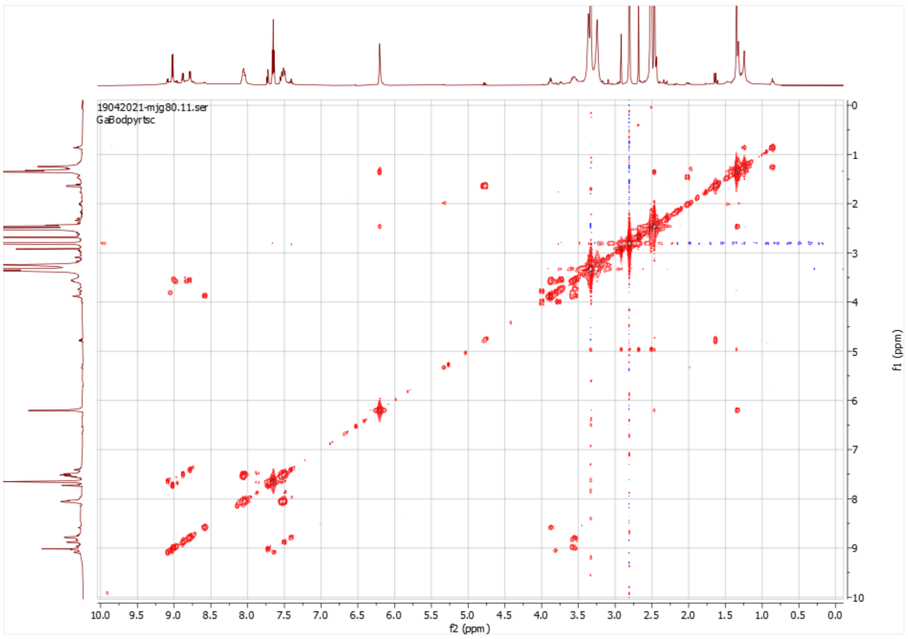
19042021-mjg80.11.ser
GaBodpyrtsc
f2 (ppm)
f1 (ppm)

(d)

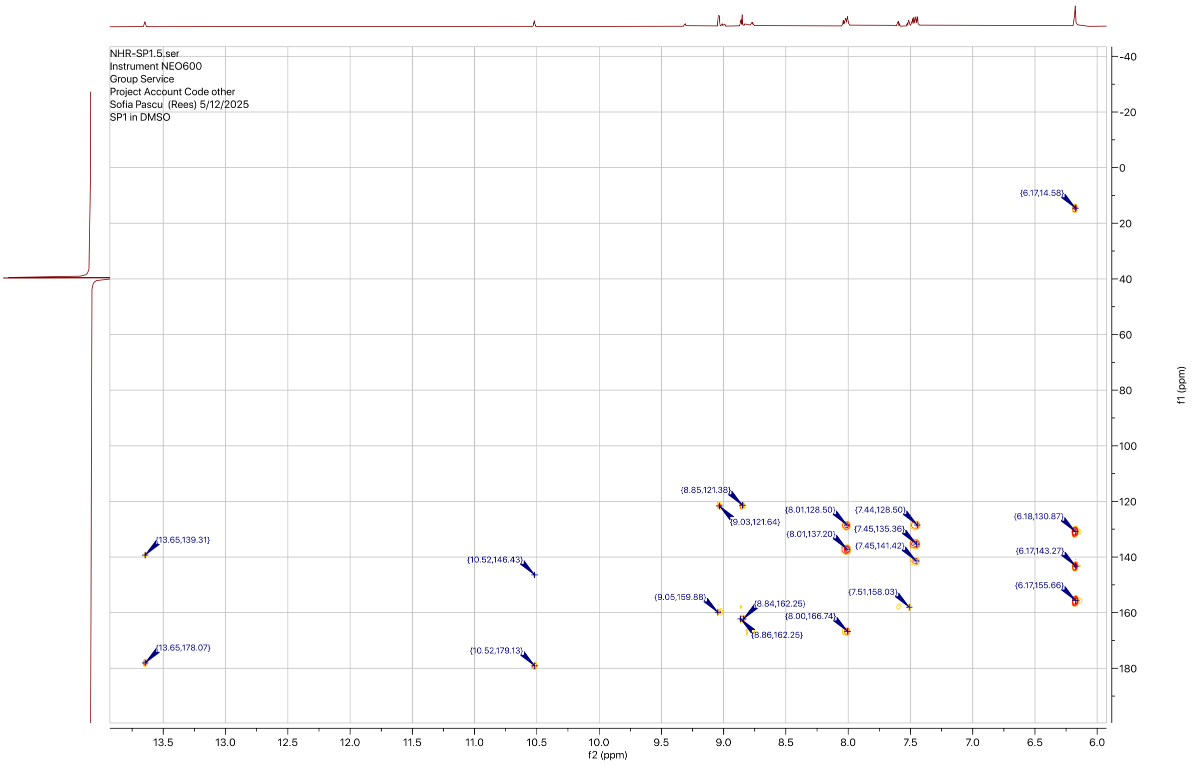

(e)

Figure S.10. (a) ^{1}H NMR spectrum (400 MHz, $(CD_3)_2SO$, 298 K) of complex **GaL3**; **(b)** ^{11}**B NMR (c)** ^{19}F NMR spectrum (376 MHz, $(CD_3)_2SO$, 298 K) **(d)** ^{1}H-^{1}H COSY **(e)** ^{1}H-^{13}C HMBC (600 MHz)

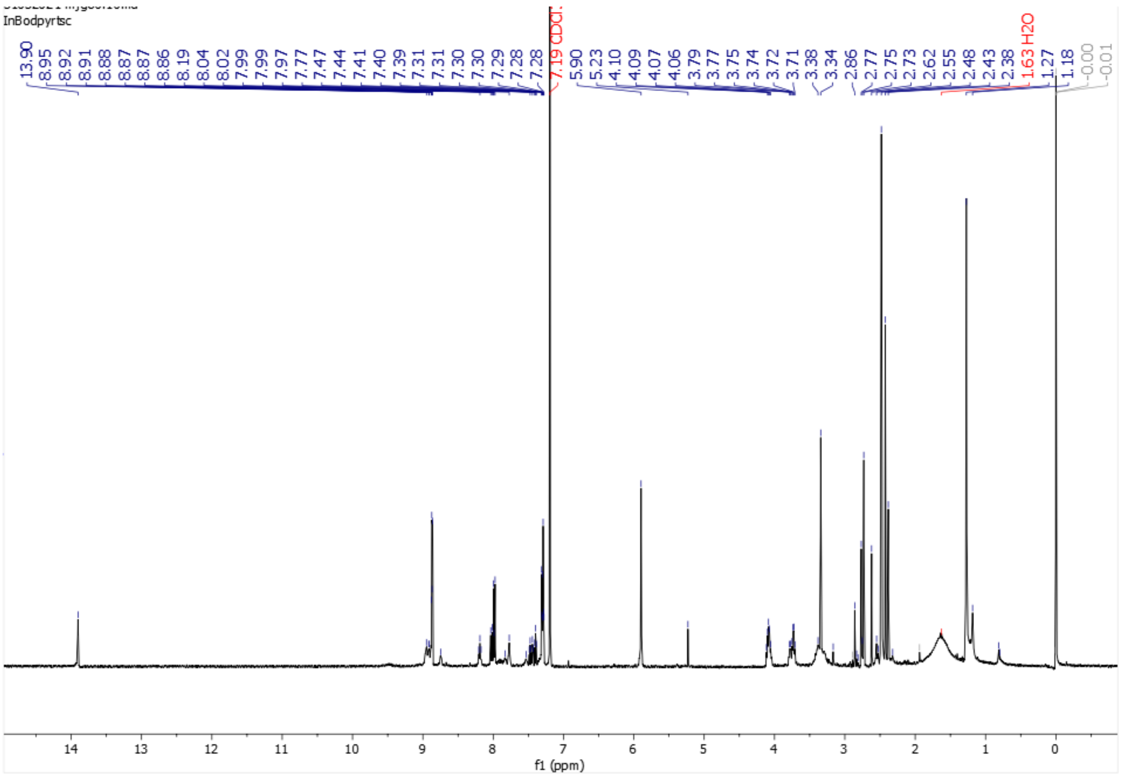

(a)

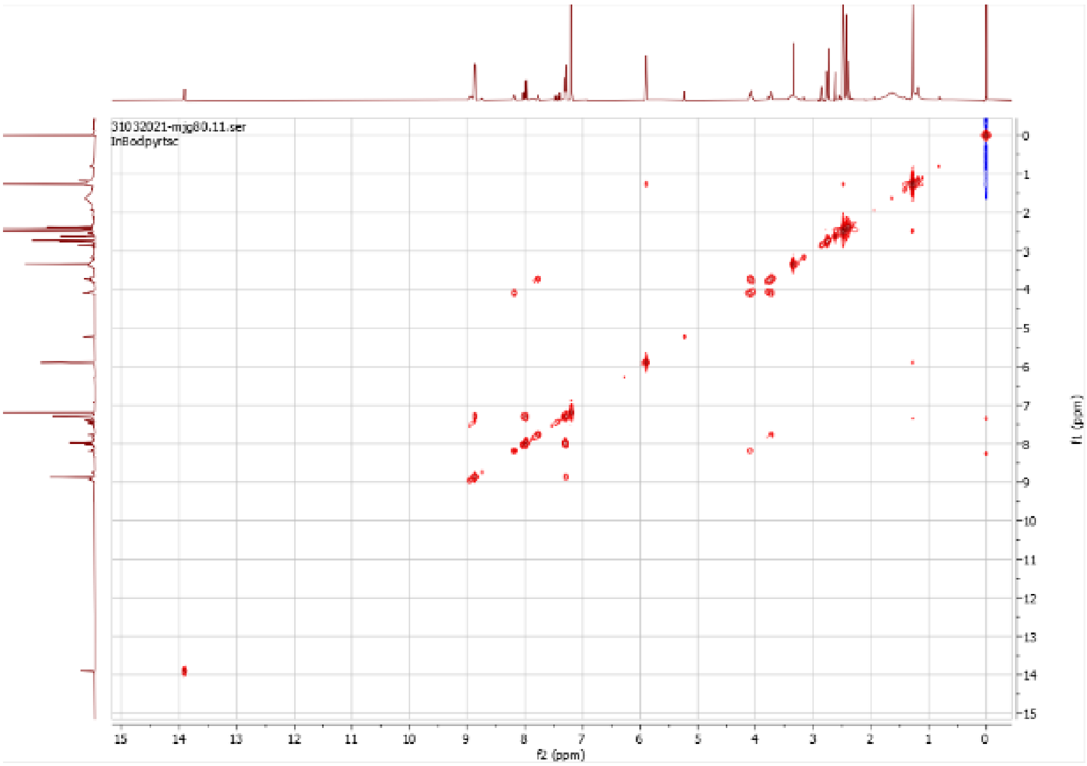

(b)

Figure S.11. (a) ^{1}H NMR spectrum (400 MHz, $(CD_3)_2SO$, 298 K) of complex **InL3**; **(b)** ^{1}H-^{1}H COSY of complex **InL3.**

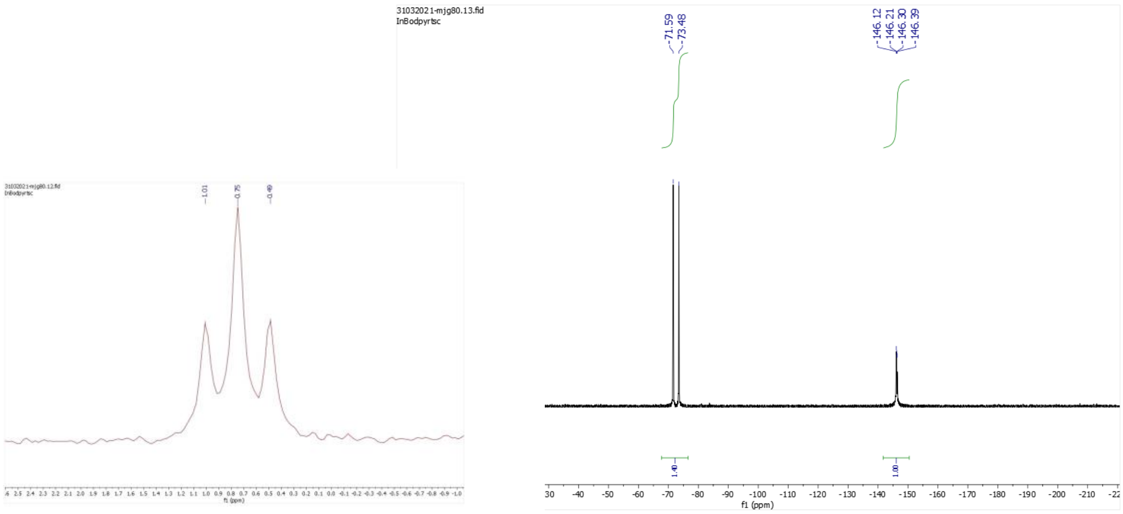

(a) (b)

Figure S.12. (a) ^{11}B NMR of complex **InL3 (b)** ^{19}F NMR spectrum (376 MHz, $(CD_3)_2SO$, 298 K) of compound **InL3**.

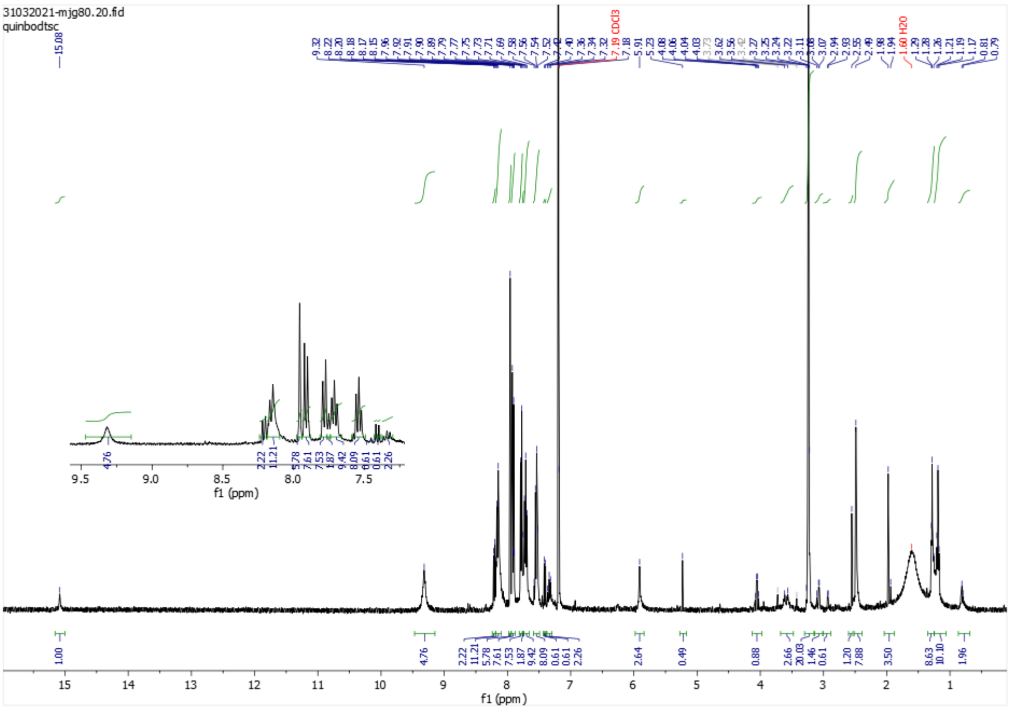
31032021-mjg80.20.fid
quinbodtsc
f1 (ppm)

(a)

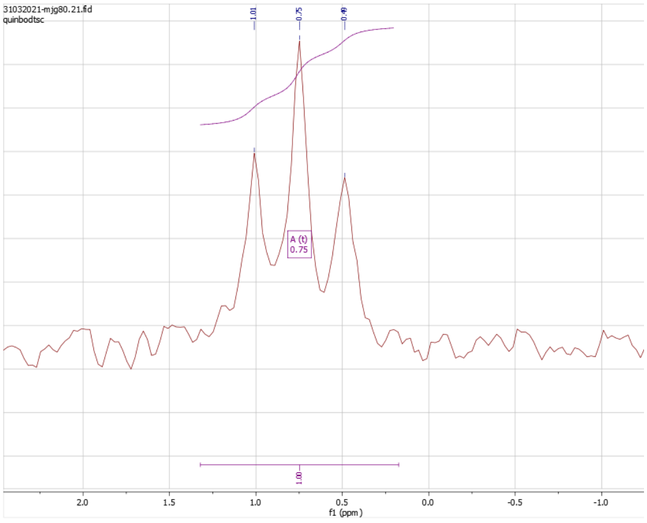
31032021-mjg80.21.fid
quinbodtsc
A (t)
0.75
f1 (ppm)

(b)

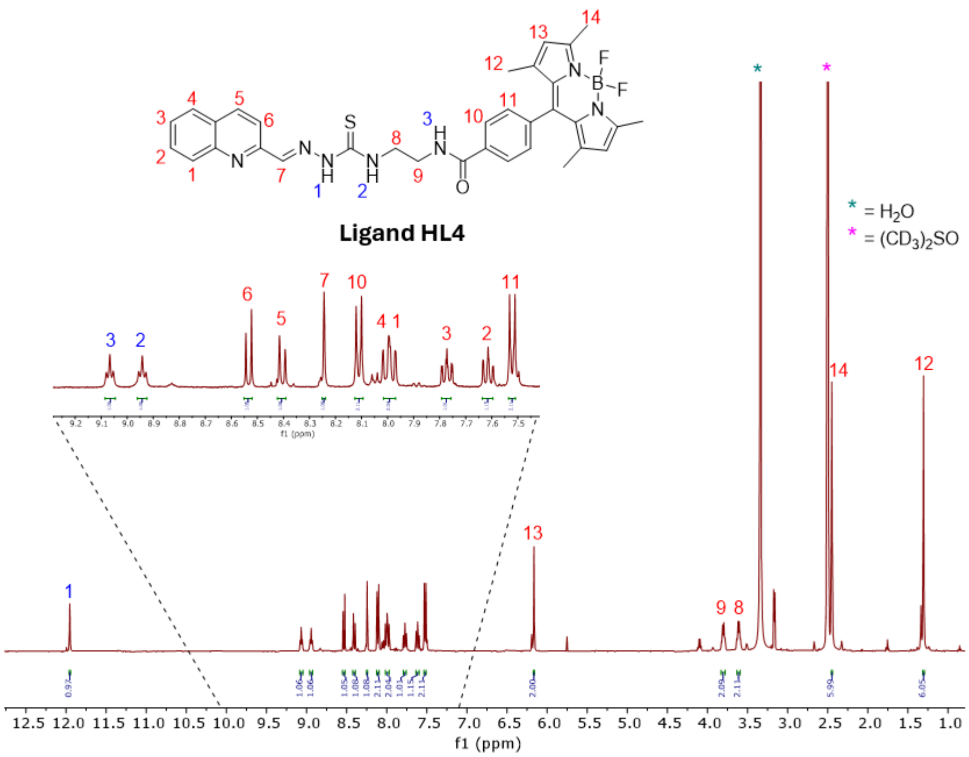

(c)

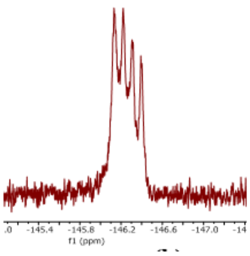

(d)

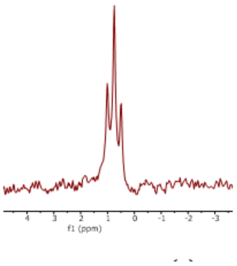

(e)

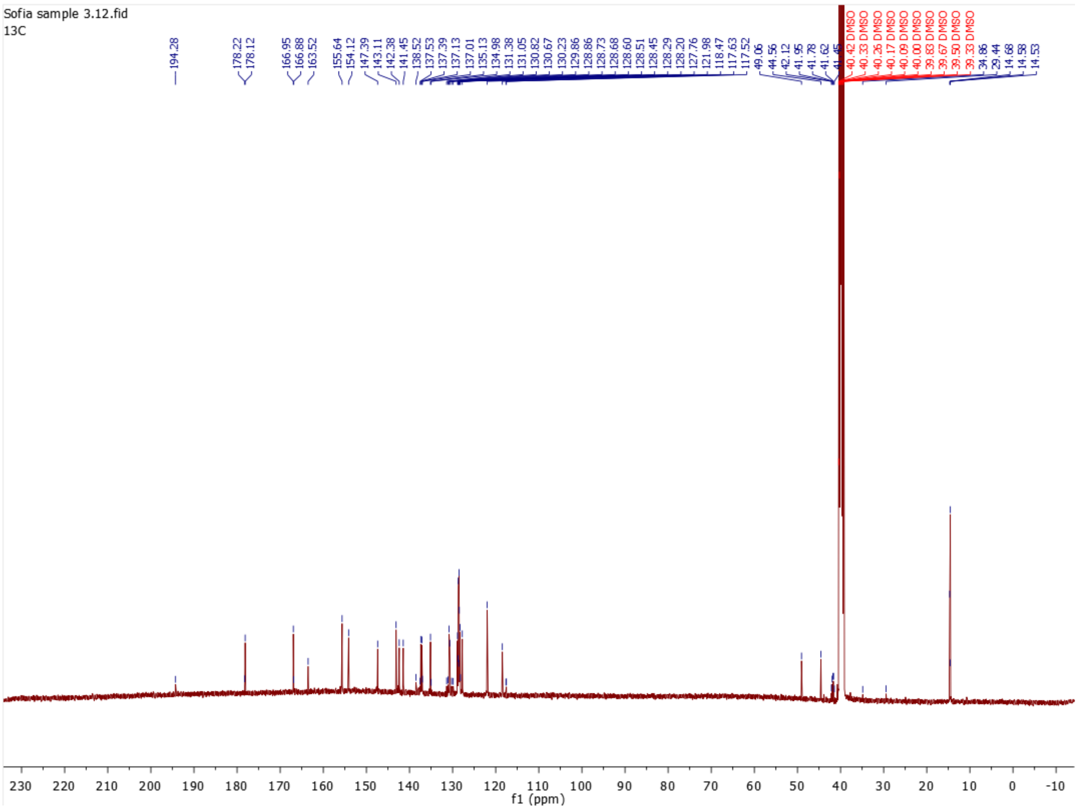
Sofia sample 3.12.fid
13C
f1 (ppm)

(f)

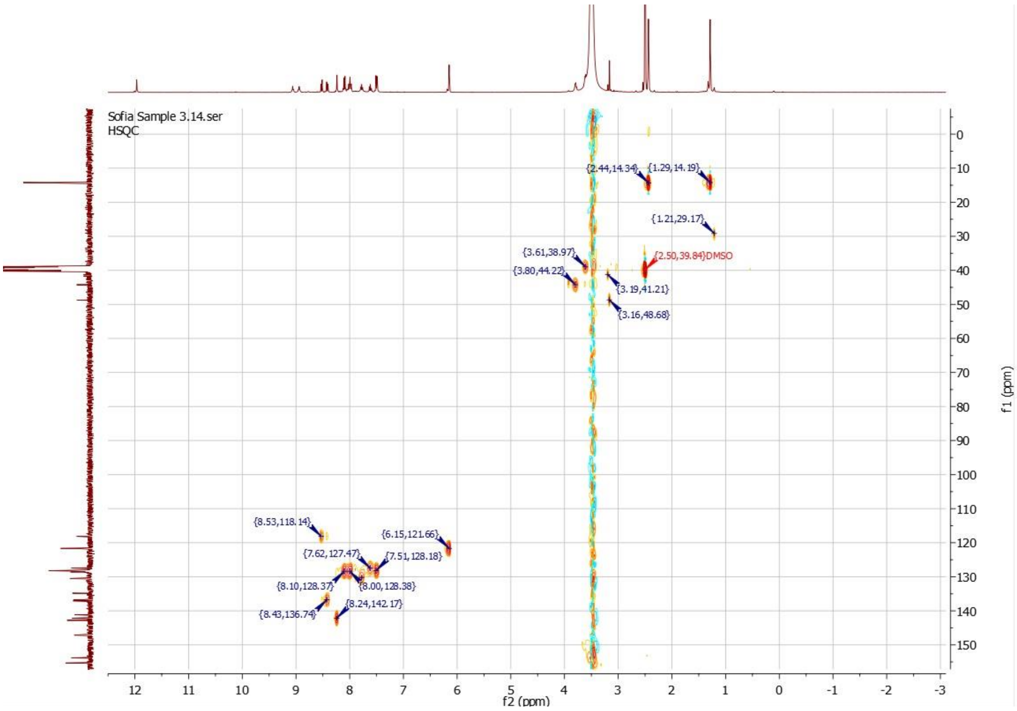
Sofia Sample 3.14.ser
HSQC
f2 (ppm)
f1 (ppm)

(g)

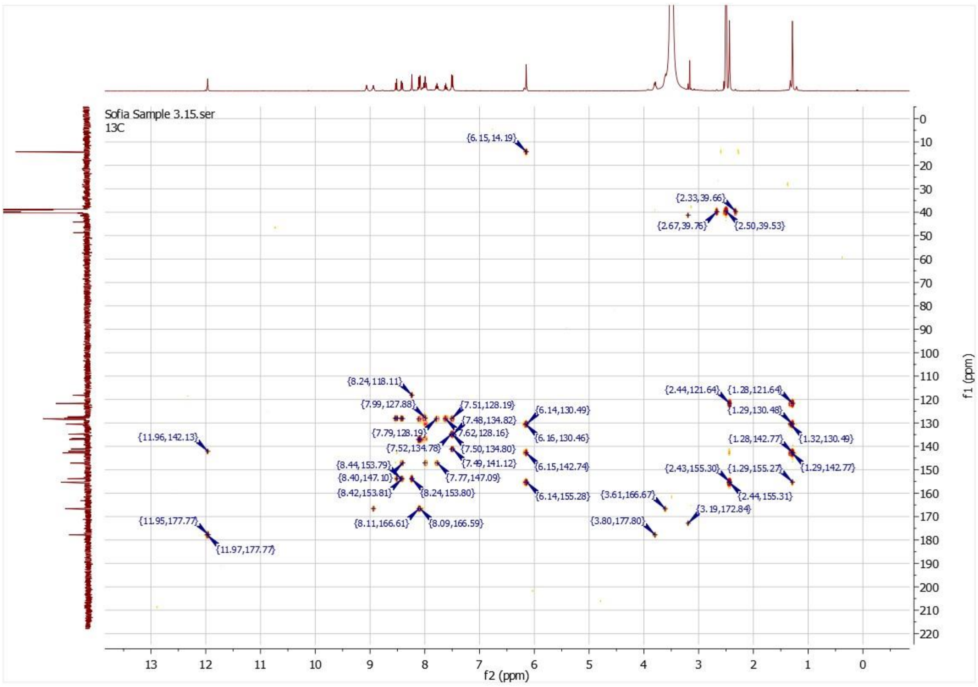

(h)

Figure S.13. NMR spectroscopy of ligand **HL4**: **(a-b)** Data recorded in fresh $CDCl_3$ solutions within 5 min of dissolving from dried powder, **(a)** ^{1}H NMR spectrum, 400 MHz, $CDCl_3$, 298 K: **(b)** ^{11}B NMR of **HL4** (128 MHz, $CDCl_3$); (**c-h**) NMR spectroscopy of **HL4** in a stabilised sample held in d_6-DMSO solution over 1 h: **(c)** ^{1}H NMR, 500 MHz, $(CD_3)_2SO$, 298 K), **(d)** ^{11}B NMR of **HL4** (128 MHz, DMSO-d_6) **(e)** ^{19}F NMR spectrum (376 MHz, $(CD_3)_2SO$, 298 K) of compound **HL4**, **(f)** $^{13}C\{^{1}H\}$ NMR, **(g)** ^{1}H-$^{13}C\{^{1}H\}$ HSQC, 500 MHz **(h)** ^{1}H-$^{13}C\{^{1}H\}$ HMBC of compound **HL4**.

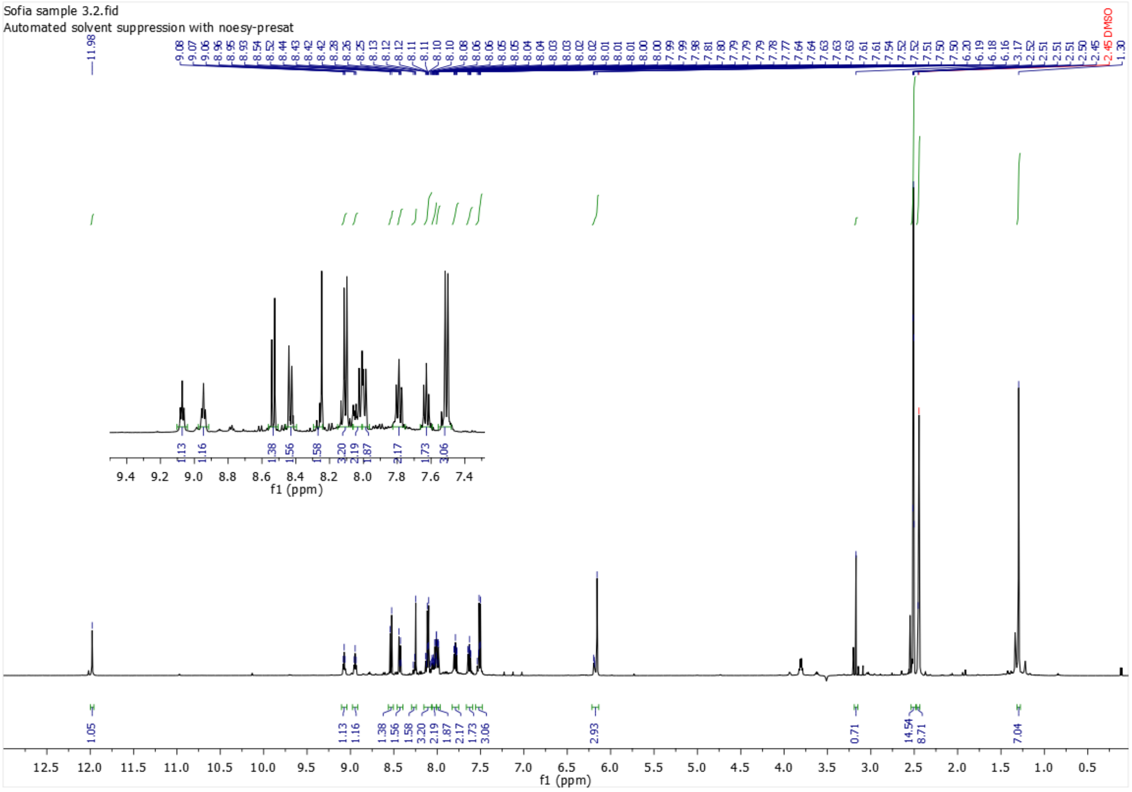
Sofia sample 3.2.fid
Automated solvent suppression with noesy-presat
2.45 DMSO
f1 (ppm)
f1 (ppm)

(a)

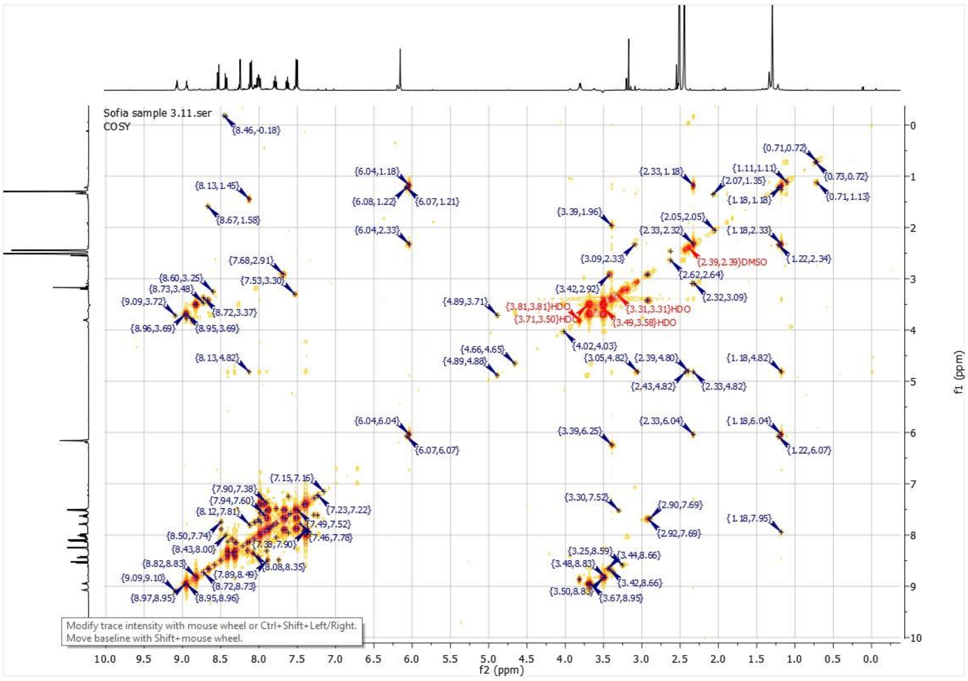
Sofia sample 3.11.ser
COSY
f2 (ppm)
f1 (ppm)
Modify trace intensity with mouse wheel or Ctrl+Shift+Left/Right.
Move baseline with Shift+mouse wheel.

(b)

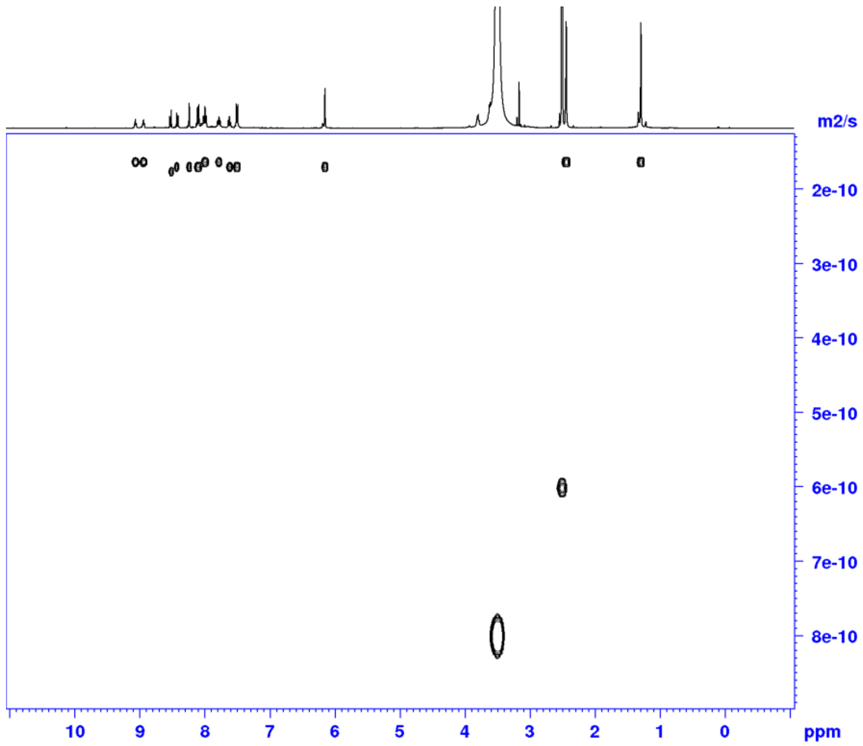

(c)

Figure S.14. NMR spectroscopy of ligand **HL4**: **(a-c)** Data recorded d_6-DMSO solutions after one week of dissolving from dried powder, **(a)** ^1H NMR spectrum, 500 MHz, $(CD_3)_2SO$, 298 K with solvent suppression **(b)** ^1H-^1H COSY with corresponding assignments and **(c)** NMR DOSY spectroscopy of **HL4**, d_6-DMSO.

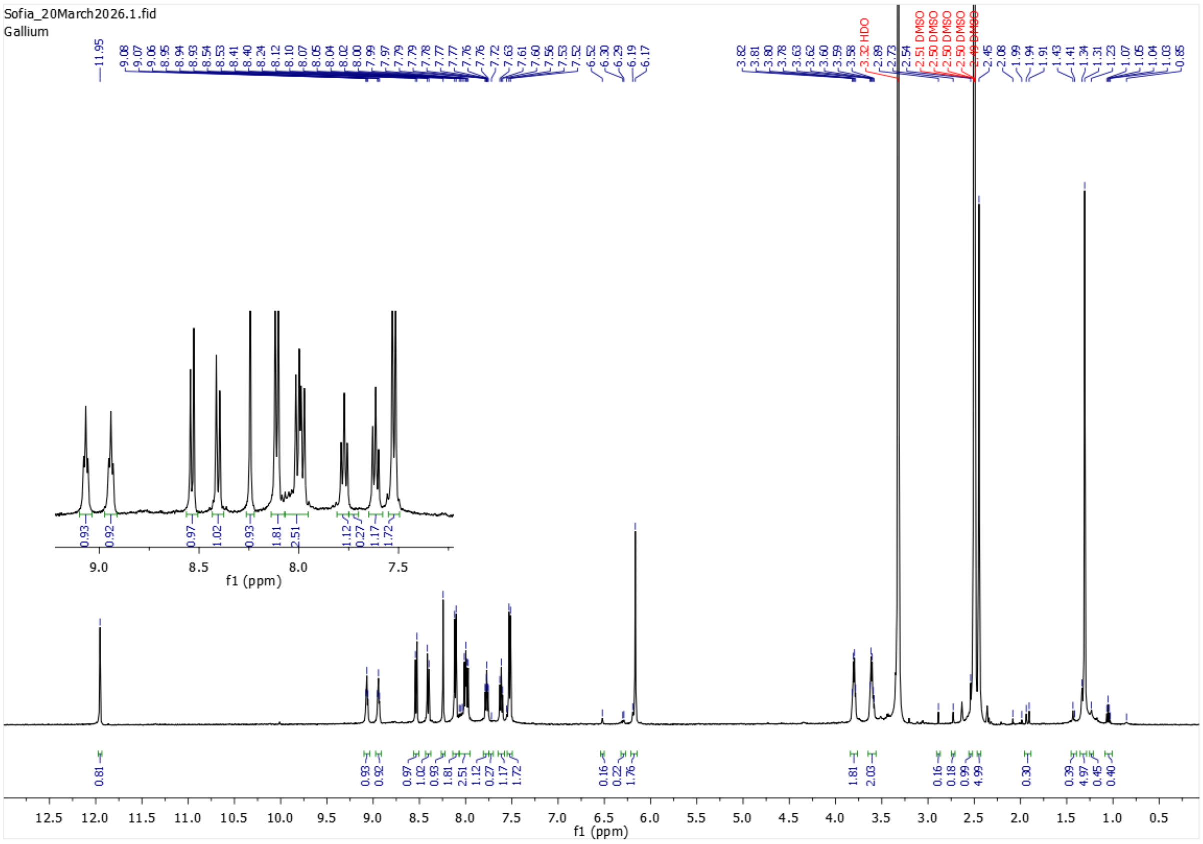
Sofia_20March2026.1.fid
Gallium
f1 (ppm)

(a)

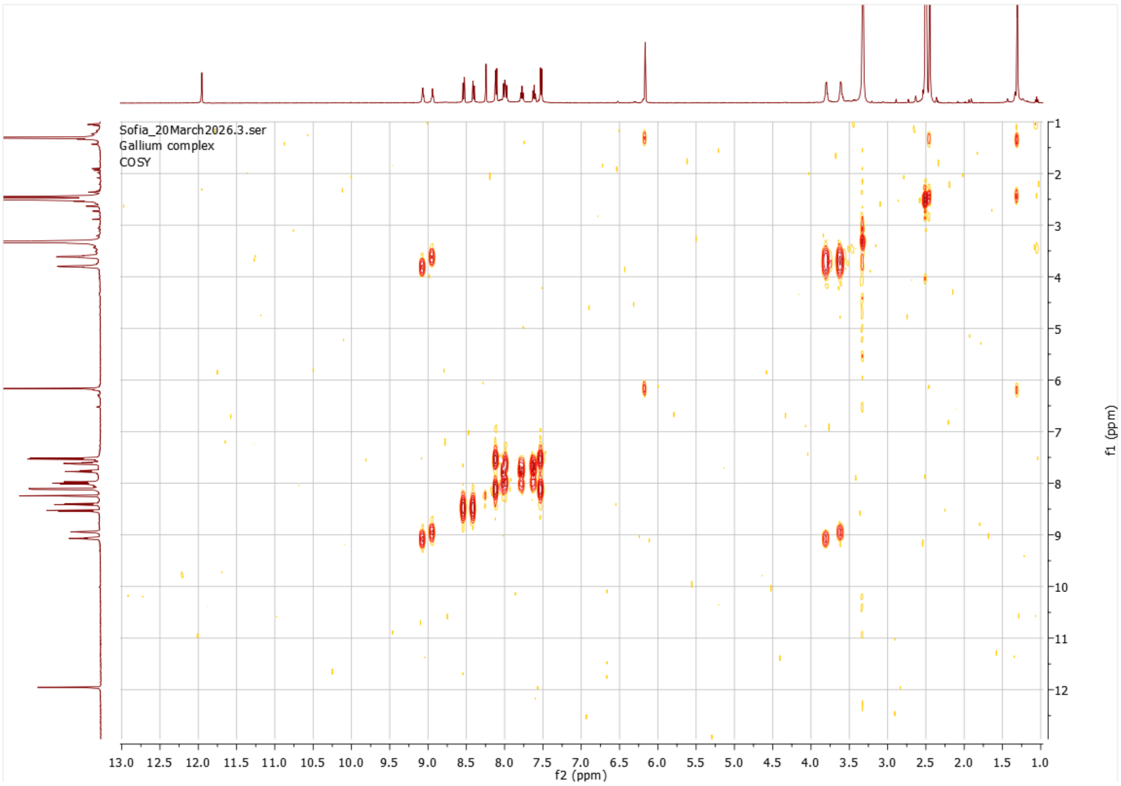
Sofia_20March2026.3.ser
Gallium complex
COSY
f2 (ppm)
f1 (ppm)

(b)

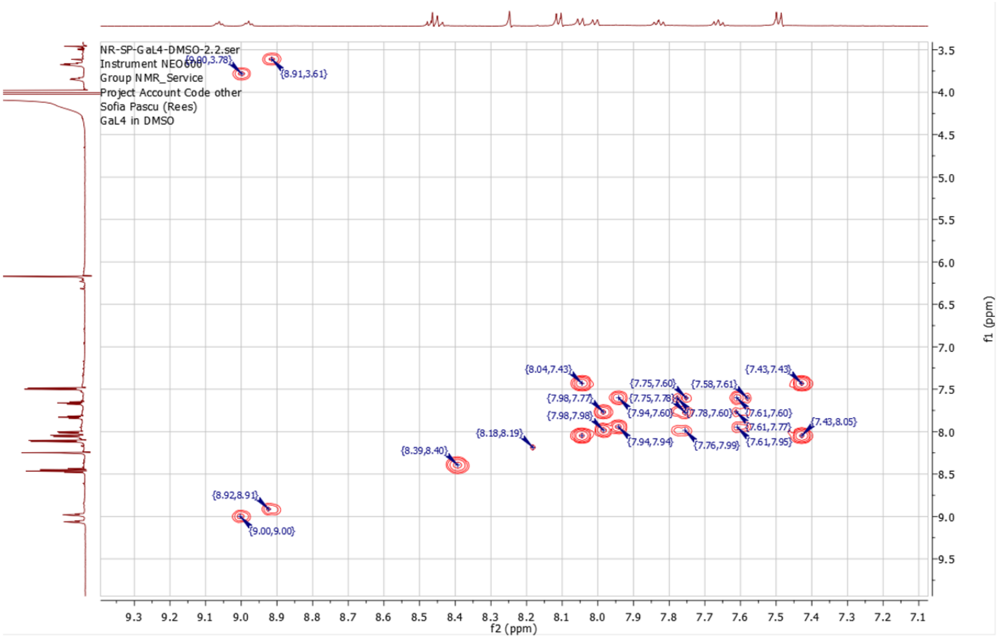
NR-SP-GaL4-DMSO-2.2.ser
Instrument NEO600
Group NMR_Service
Project Account Code other
Sofia Pascu (Rees)
GaL4 in DMSO
f2 (ppm)
f1 (ppm)

(c)

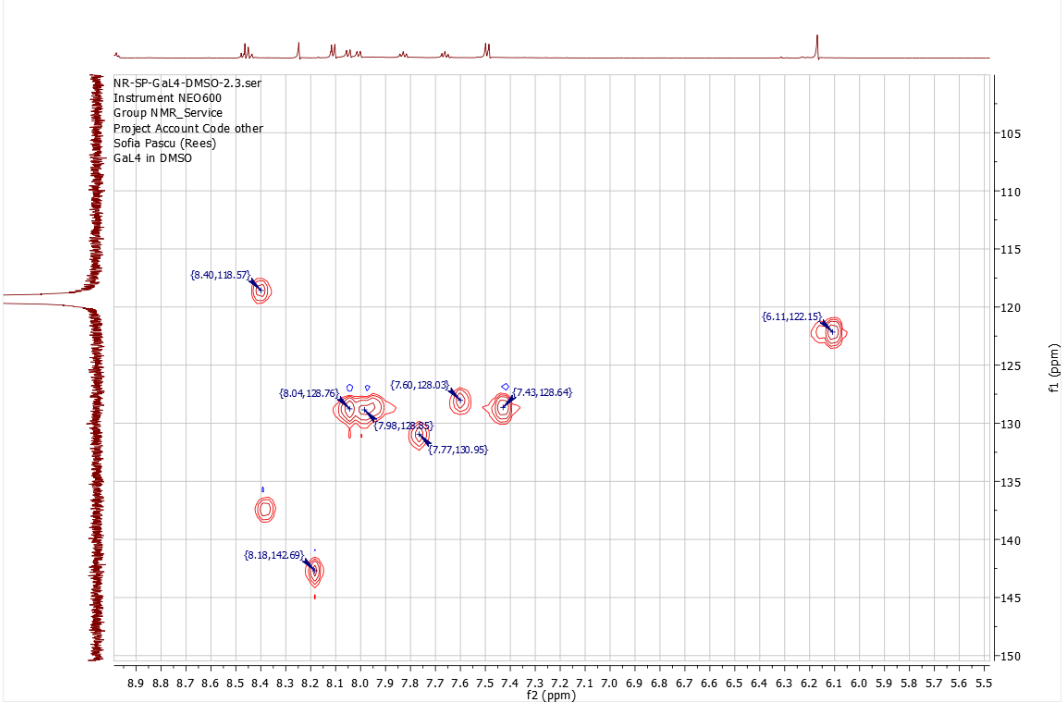
NR-SP-GaL4-DMSO-2.3.ser
Instrument NEO600
Group NMR_Service
Project Account Code other
Sofia Pascu (Rees)
GaL4 in DMSO
{8.40,118.57}
{6.11,122.15}
{8.04,128.76}
{7.60,128.03}
{7.43,128.64}
{7.98,128.85}
{7.77,130.95}
{8.18,142.69}
f2 (ppm)
f1 (ppm)

(d)

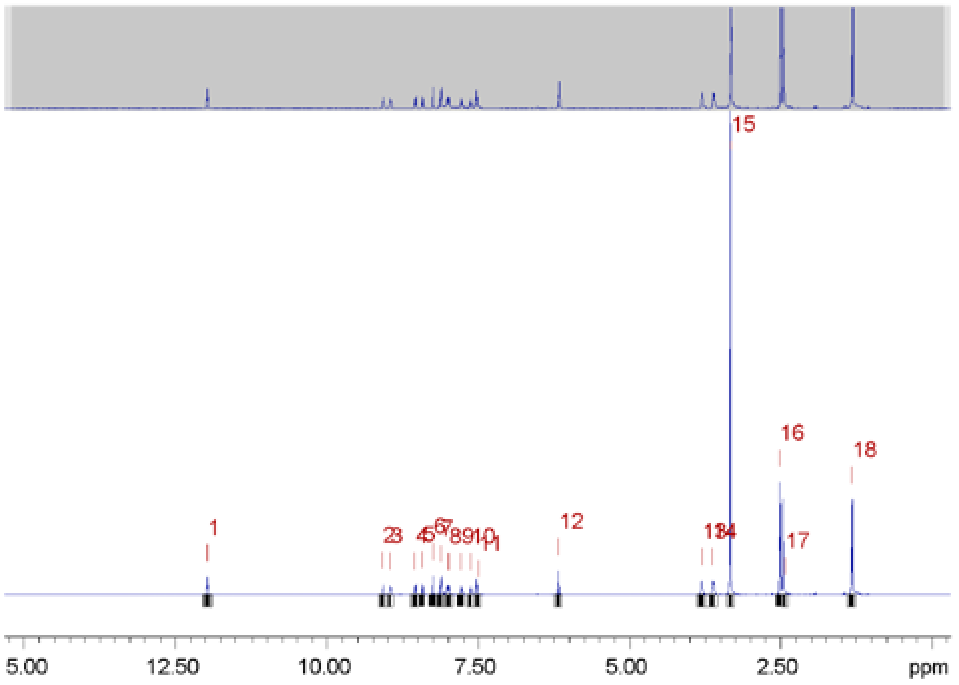

15
16
18
1
23
45
67
8910
11
12
1314
17
15.00
12.50
10.00
7.50
5.00
2.50
ppm

Dosy/Fit

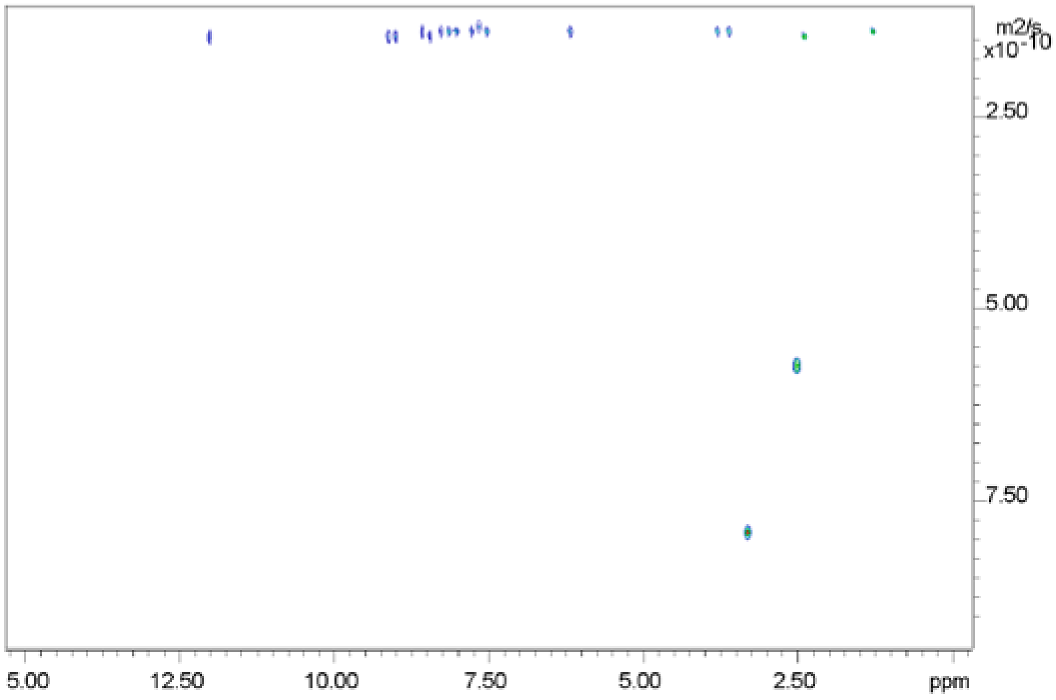

m2/s
x10-10
2.50
5.00
7.50
15.00
12.50
10.00
7.50
5.00
2.50
ppm

(e)

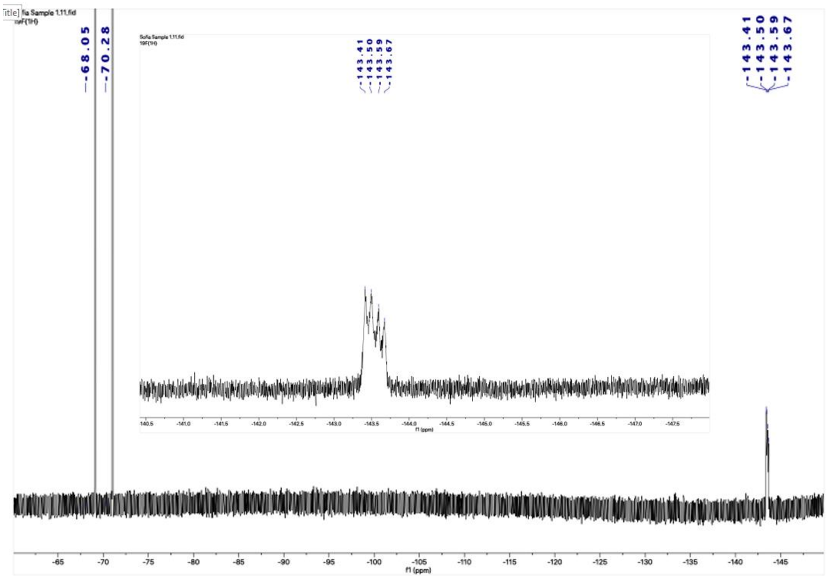

fia Sample 1.11.fid
-68.05
-70.28
-143.41
-143.50
-143.59
-143.67
f1 (ppm)

(f)

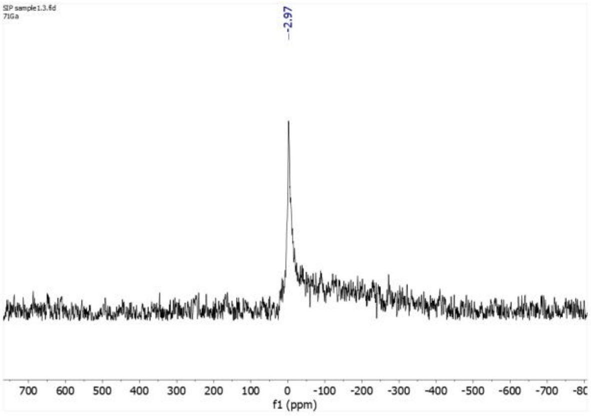

71Ga
-2.97
f1 (ppm)

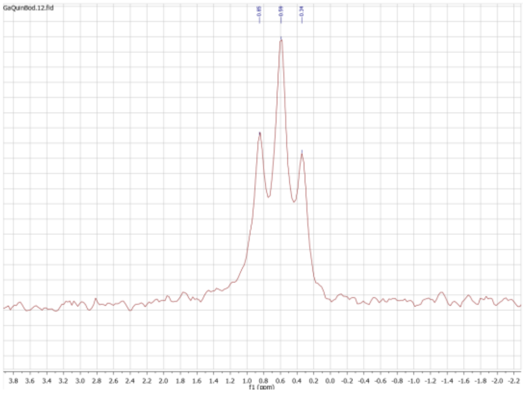

(g) (h)

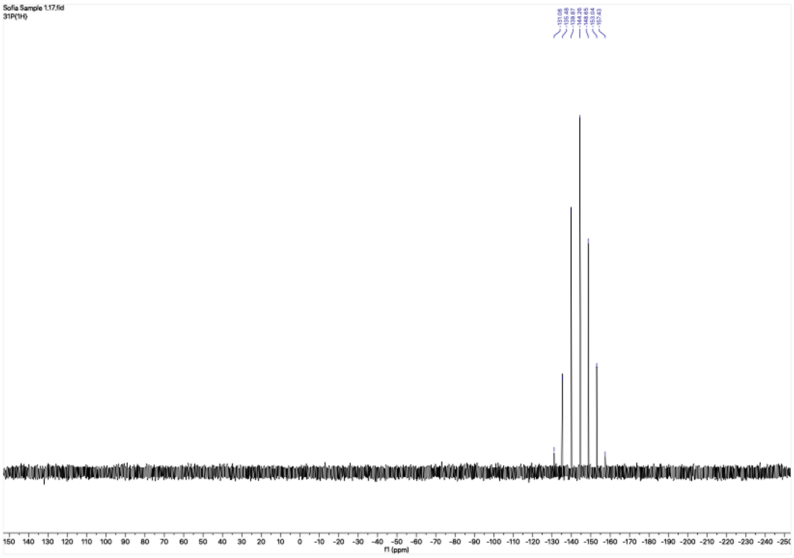

Sofia Sample 1.17.fid
31P(1H)
f1 (ppm)

(i)

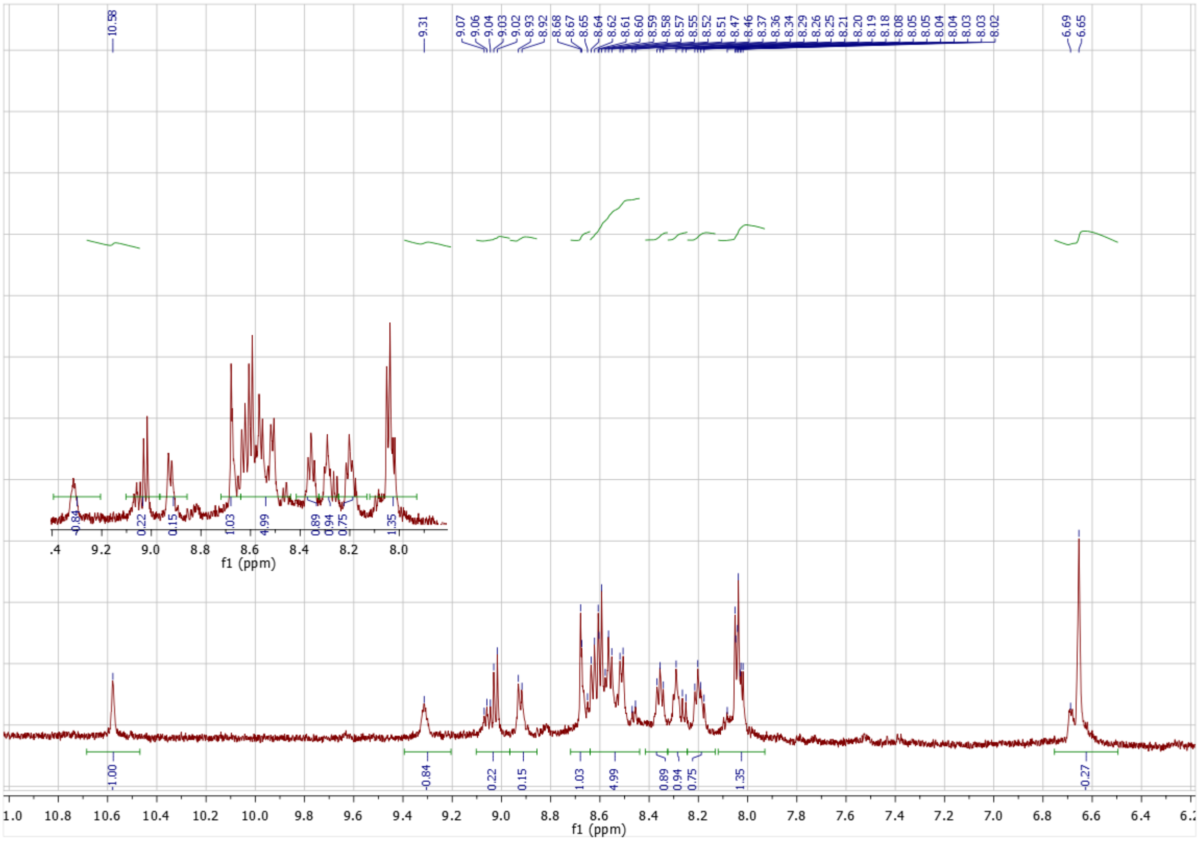

(j)

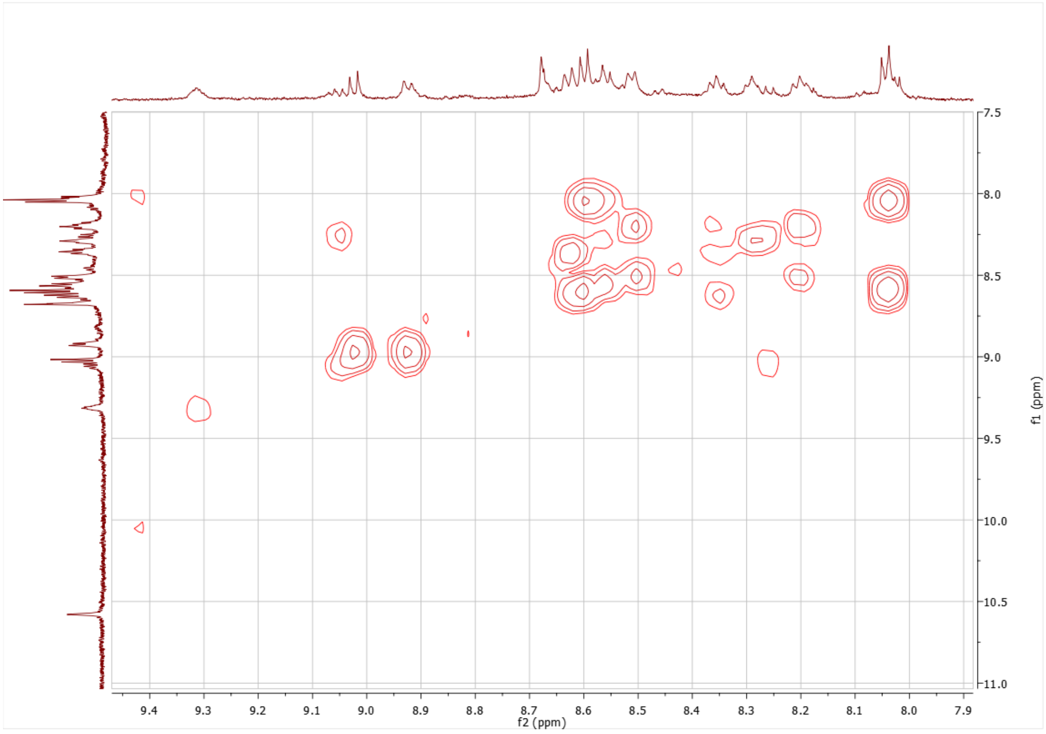

(k)

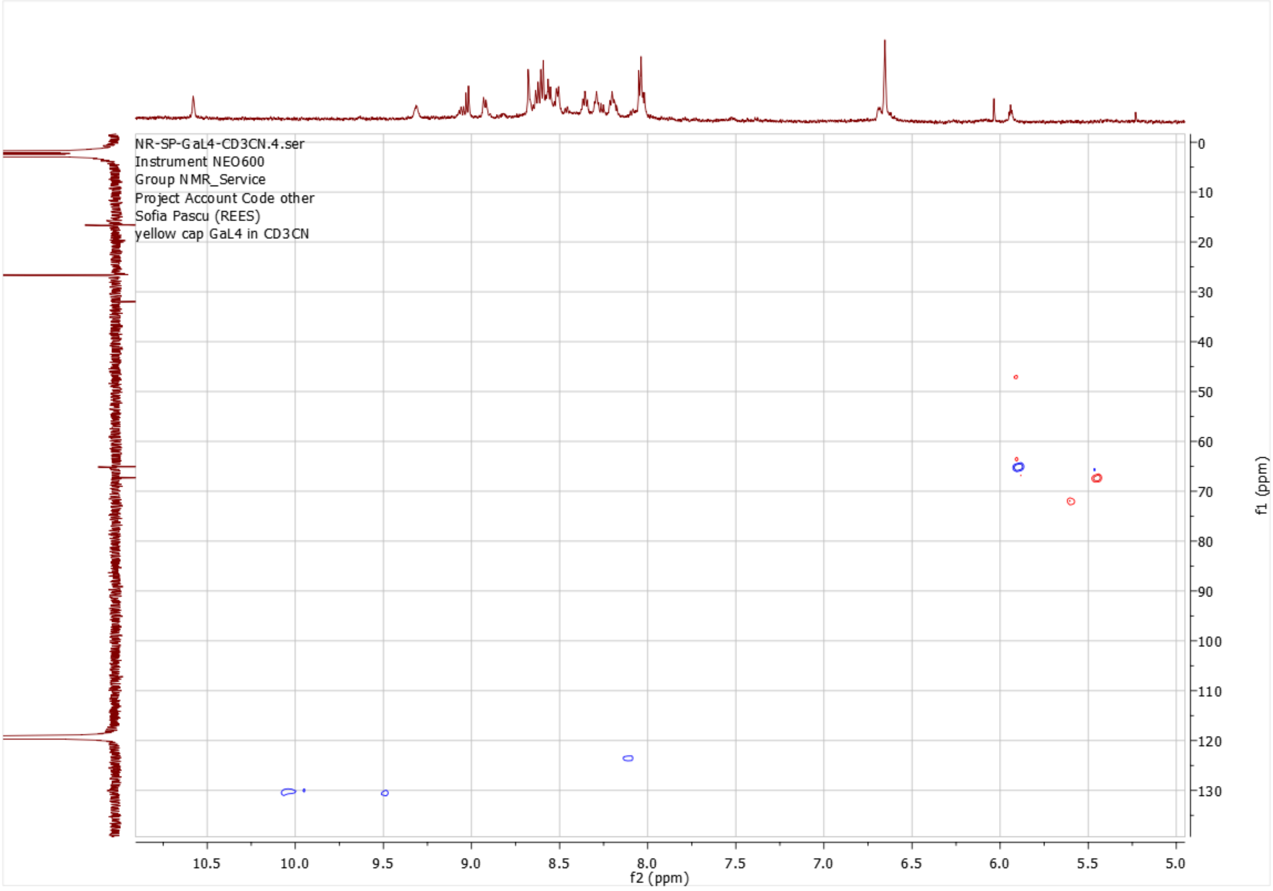
NR-SP-GaL4-CD3CN.4.ser
Instrument NEO600
Group NMR_Service
Project Account Code other
Sofia Pascu (REES)
yellow cap GaL4 in CD3CN
f2 (ppm)
f1 (ppm)

(l)

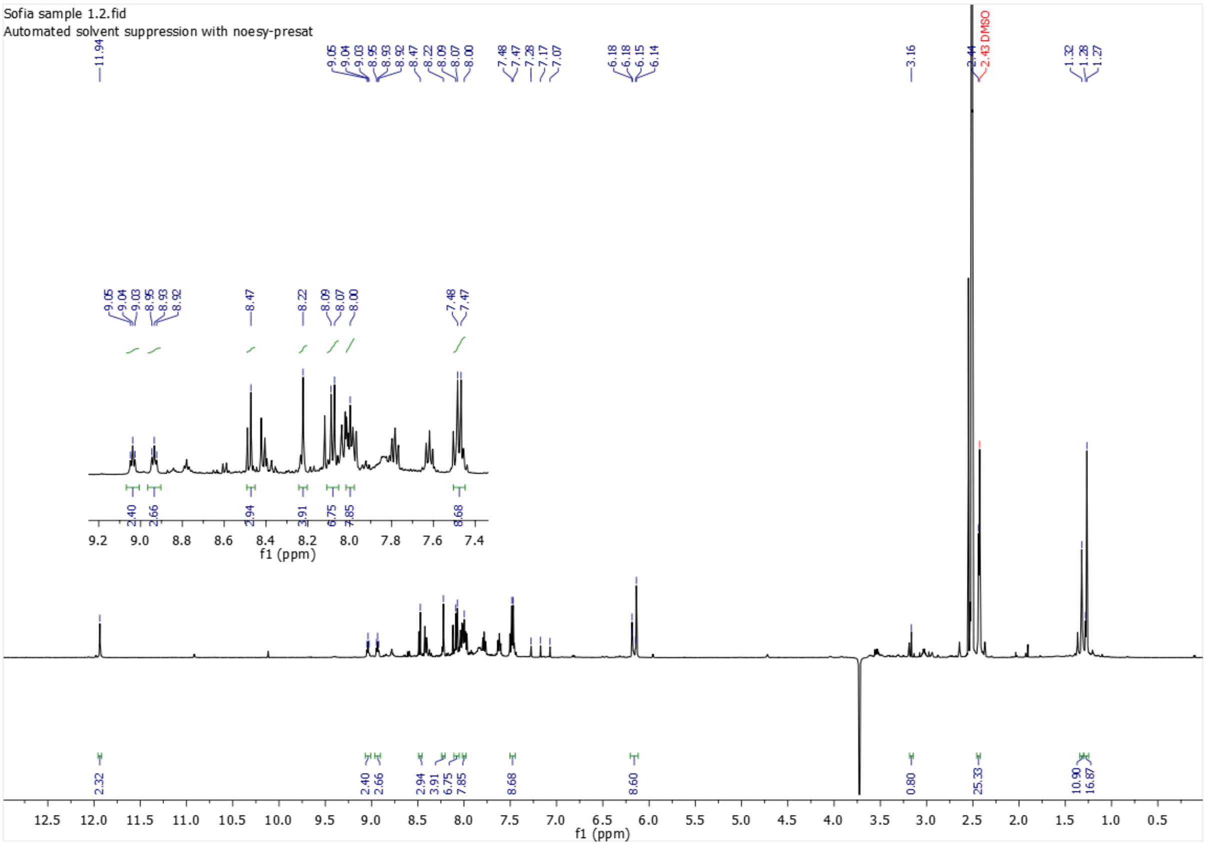
Sofia sample 1.2.fid
Automated solvent suppression with noesy-presat
2.43 DMSO
f1 (ppm)

(m)

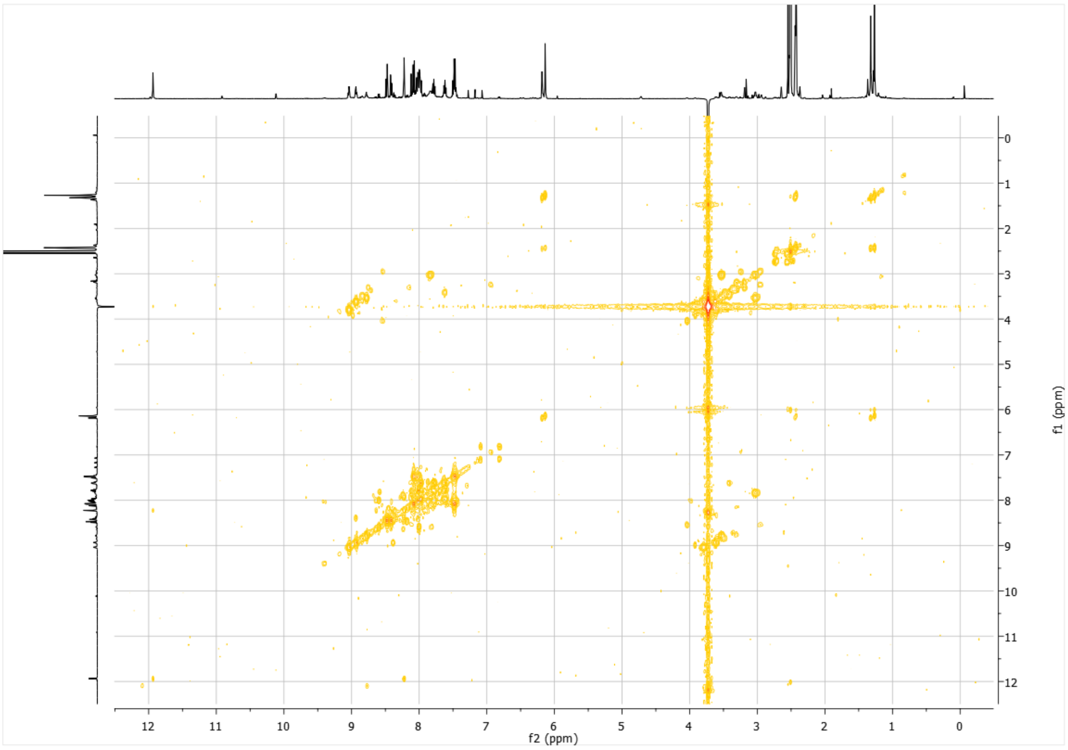
f2 (ppm)
f1 (ppm)

(n)

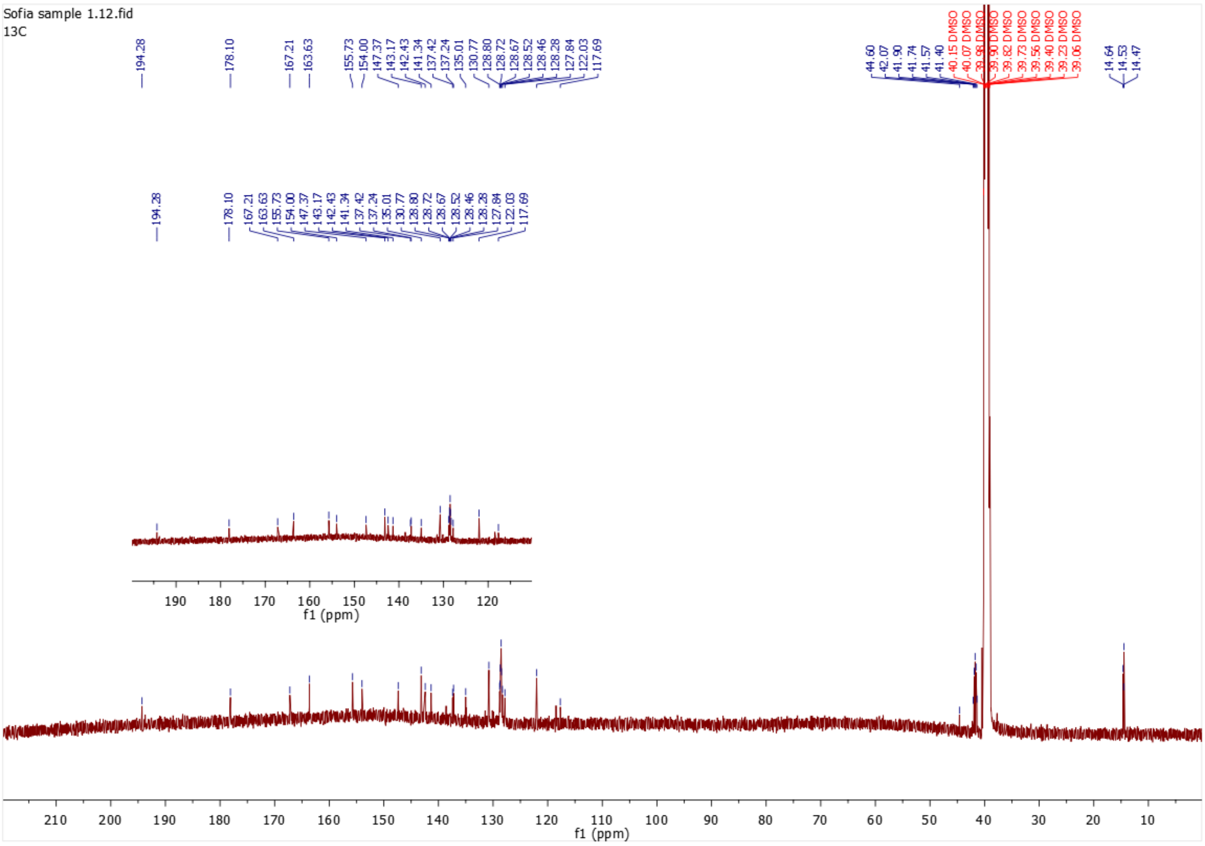
Sofia sample 1.12.fid
13C
f1 (ppm)

(o)

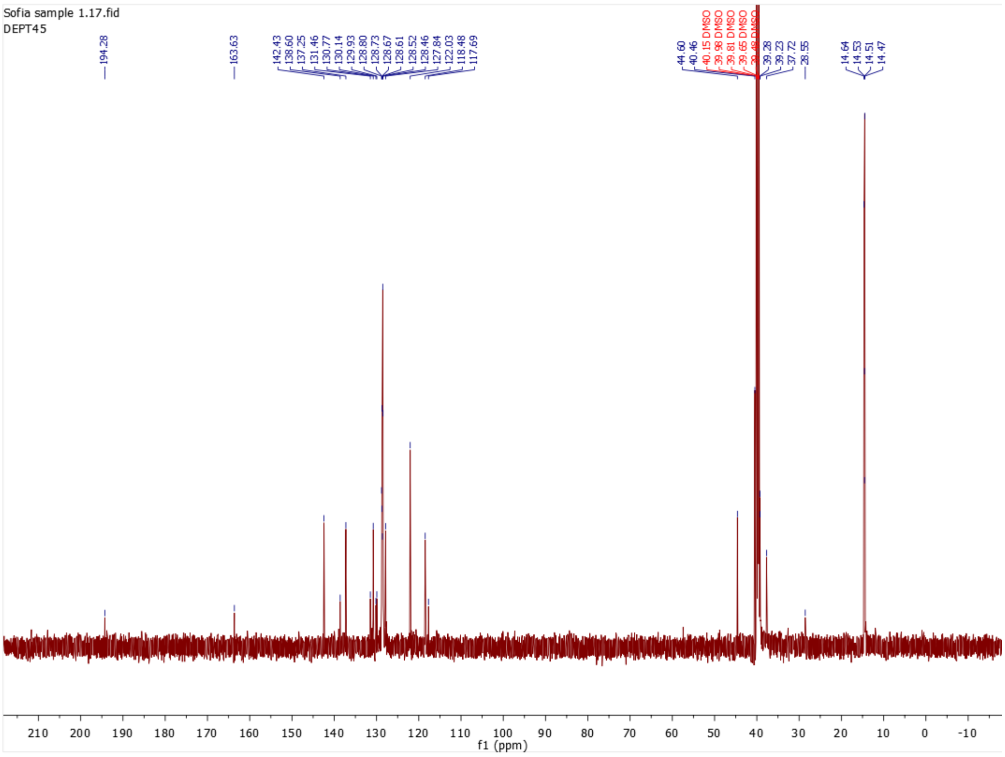
Sofia sample 1.17.fid
DEPT45
f1 (ppm)

(p)

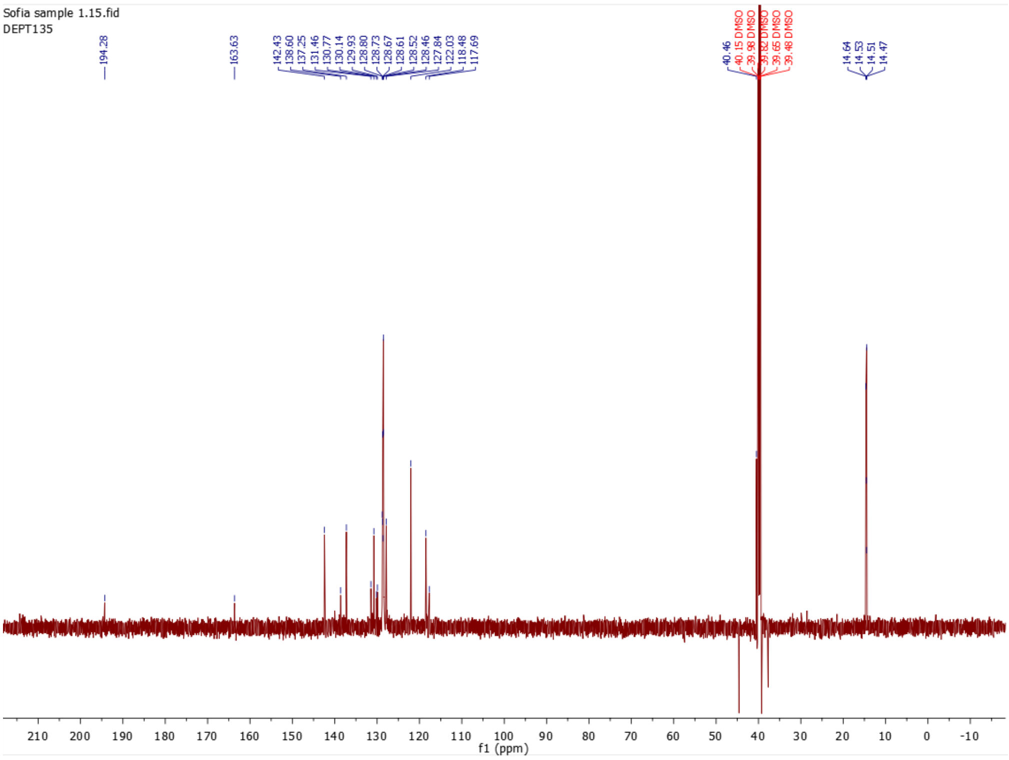
Sofia sample 1.15.fid
DEPT135
194.28
163.63
142.43
138.60
137.25
131.46
130.77
130.14
129.93
128.80
128.73
128.67
128.61
128.52
128.46
127.84
122.03
118.48
117.69
40.46
40.15 DMSO
39.98 DMSO
39.82 DMSO
39.65 DMSO
39.48 DMSO
14.64
14.53
14.51
14.47
f1 (ppm)

(q)

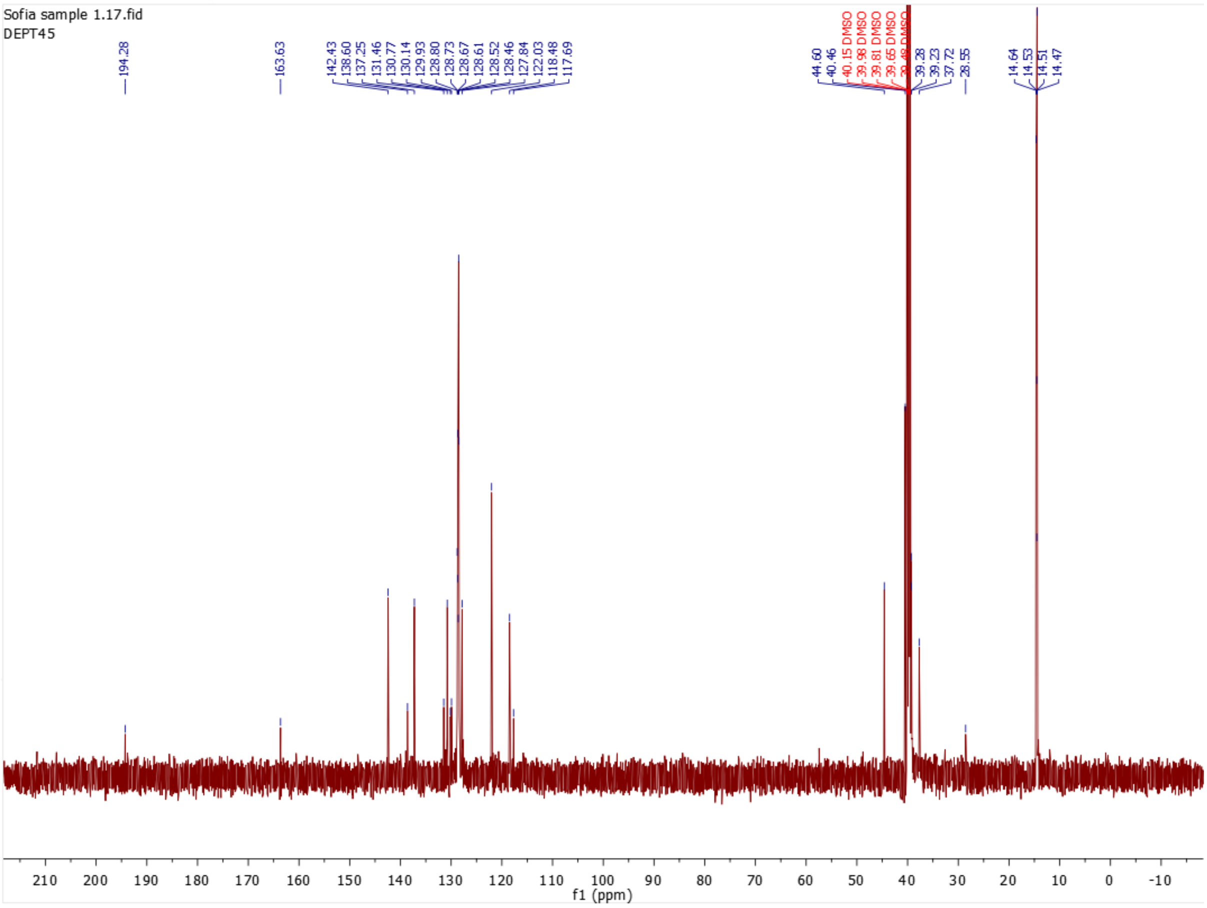
Sofia sample 1.17.fid
DEPT45
194.28
163.63
142.43
138.60
137.25
131.46
130.77
130.14
129.93
128.80
128.73
128.67
128.61
128.52
128.46
127.84
122.03
118.48
117.69
44.60
40.46
40.15 DMSO
39.98 DMSO
39.81 DMSO
39.65 DMSO
39.28
39.23
37.72
28.55
14.64
14.53
14.51
14.47
f1 (ppm)

(r)

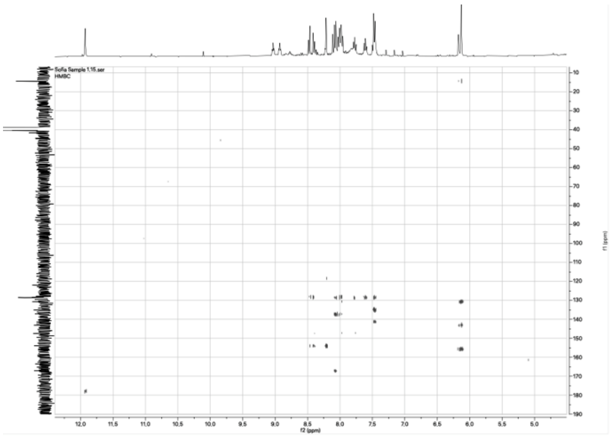

(s)

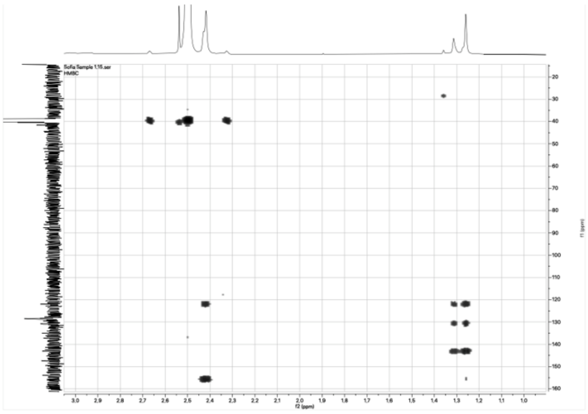

(t)

Figure S15. NMR spectroscopic characterisation of **GaL4** in fresh and aged solutions, monitored over one month. Spectra recorded in fresh DMSO-d_6 **(a–i)** and CD_3CN **(j–l)** at 500 and 600 MHz, and in 1 week-old DMSO-d_6 solutions, together with proposed speciation route supported by ^{1}H NMR **(v)**: **(a)** ^{1}H NMR spectrum with expansion; **(b)** ^{1}H–^{1}H COSY; **(c)** ^{1}H–^{1}H COSY with solvent suppression; **(d)** DOSY spectrum of fresh solutions; **(e)** ^{1}H–^{13}C HSQC (600 MHz); **(f)** ^{19}F NMR (376 MHz, DMSO-d_6); **(g)** ^{71}Ga NMR (122 MHz, DMSO-d_6), $\delta = -2.97$; **(h)** ^{11}B NMR (128 MHz, DMSO-d_6); **(i)** ^{31}P NMR. Spectra in CD_3CN: **(j)** ^{1}H NMR with expansion (recorded within 2 h of dissolution); **(k)** ^{1}H–^{1}H COSY; **(l)** ^{1}H–^{13}C HSQC. Measurements of aged DMSO-d_6 solutions (1 week): **(m)** ^{1}H NMR (500 MHz); **(n)** ^{1}H–^{1}H COSY; **(o–q)** ^{13}C DEPT experiments; **(r-t)** ^{1}H–^{13}C HSQC and HMBC experiments and corresponding expansion (600 MHz);

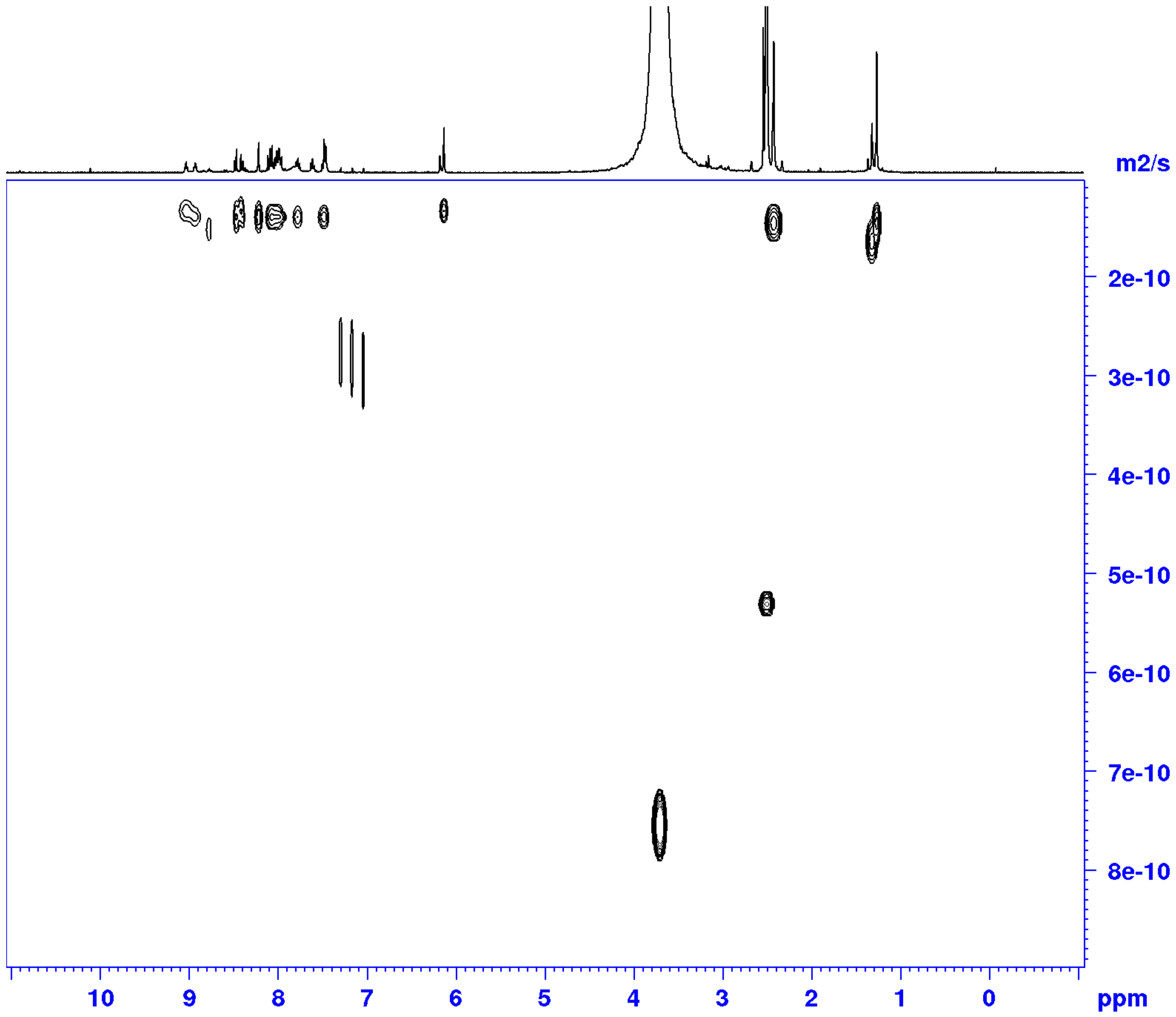

(a)

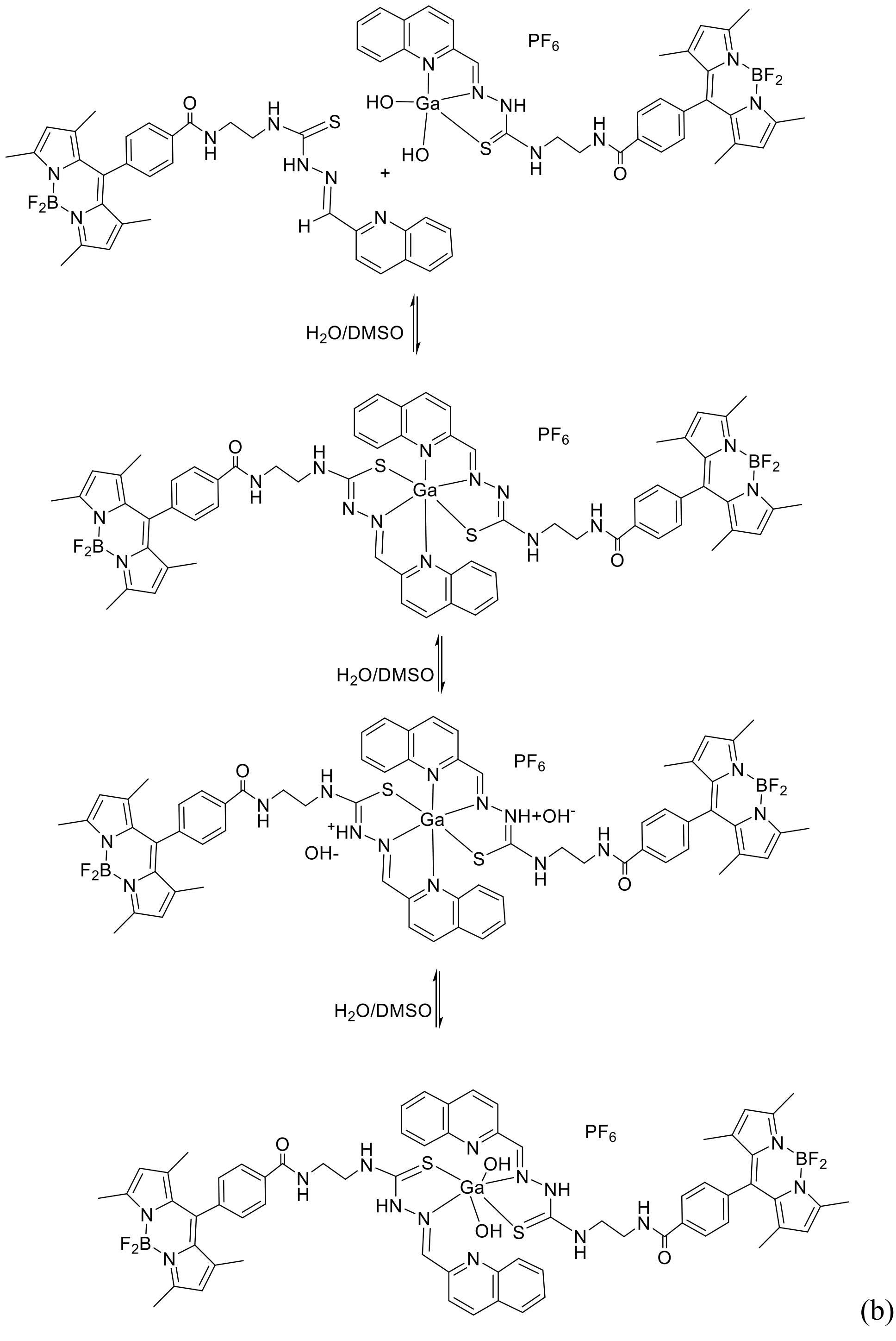

(b)

Figure S16. (a) DOSY spectrum, indicating high kinetic stability over >1 week. **(b)** Proposed speciation pathway in aqueous DMSO based on NMR data from samples monitored from 20 minutes and over 1 month. Additionally to the growth of the signals in the 11-12 ppm region of the spectra assignable to ligand protonation at hydrazinic sites, a small 1:1:1 triplet was observed in ^{1}H NMR spectra of aged DMSO/H_2O solutions, absent in fresh samples and not detected in CD_3CN; this signal collapses upon solvent suppression experiments and is tentatively assigned to quinoline protonation or NH_4^+ formation. This is consistent with slow thiosemicarbazone cyclisation and/or decomposition mediated by Ga(III), which in wet media forms $(GaOH)_x$ species, hydrolytic oligomers, and mildly acidic solutions.

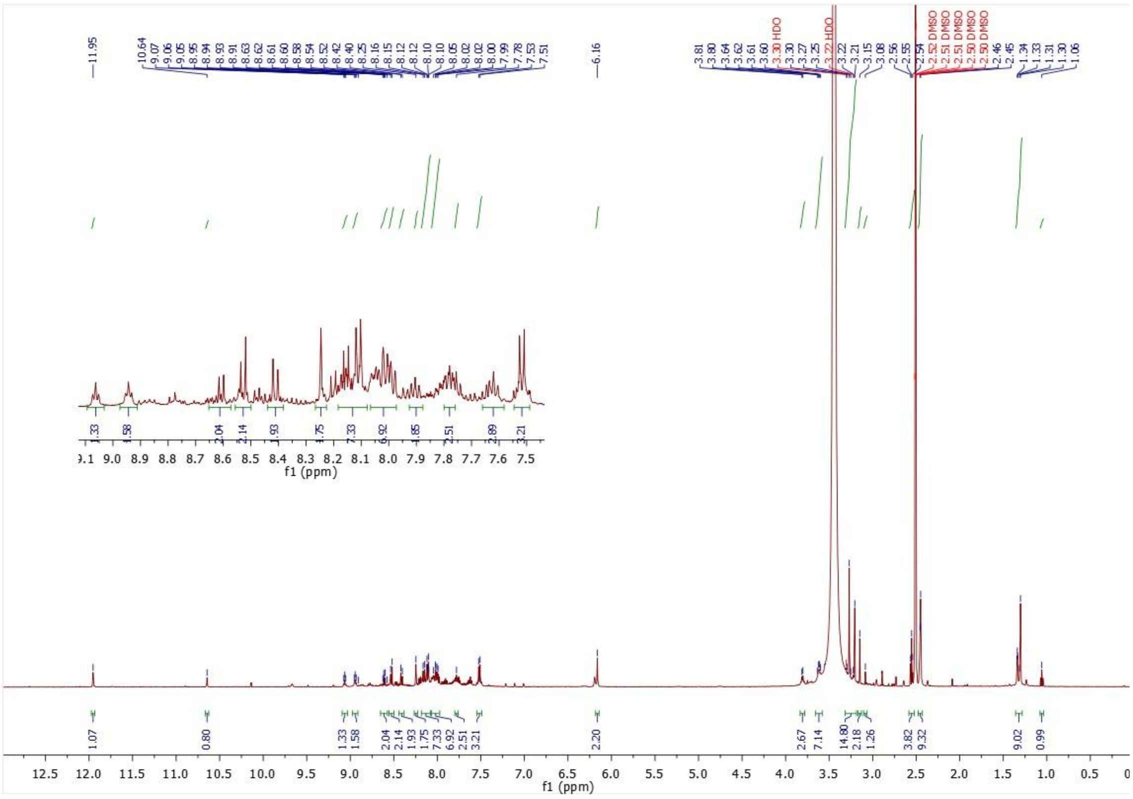

(a)

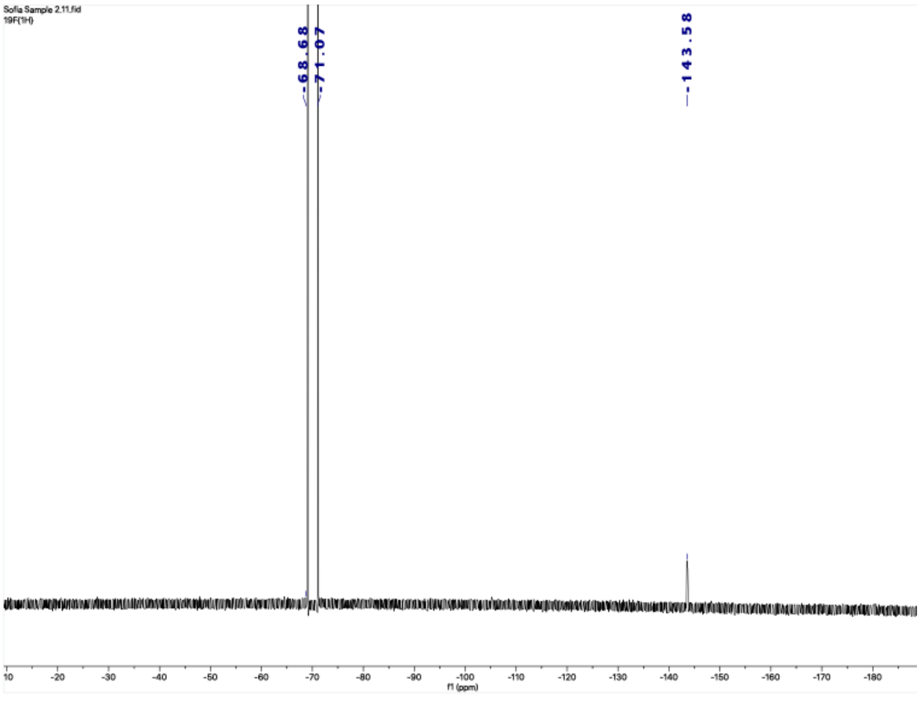

(b)

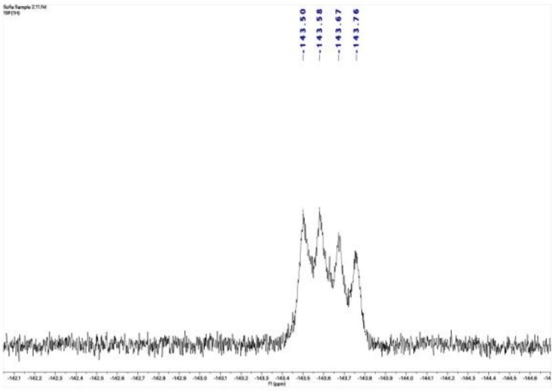

-143.50
-143.58
-143.67
-143.76
f1 (ppm)

(c)

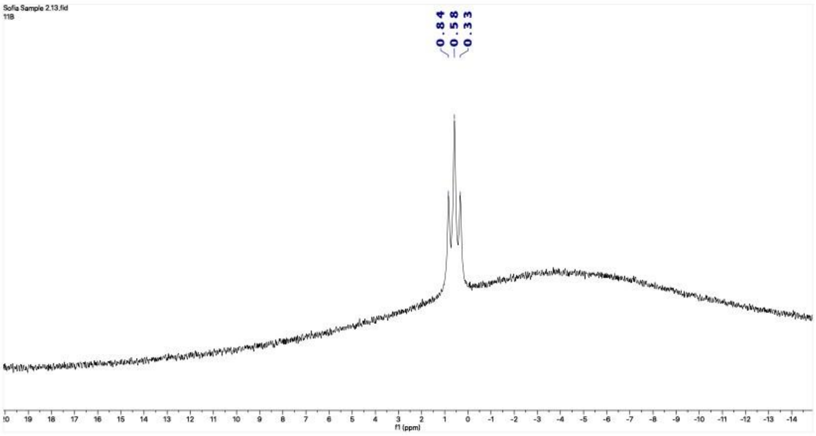

Sofia Sample 2.13.fid
11B
0.84
0.58
0.33
f1 (ppm)

(d)

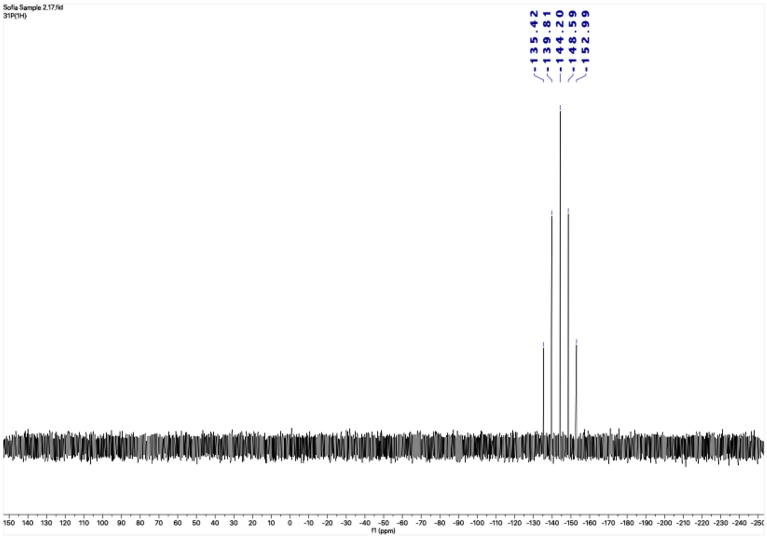

Sofia Sample 2.17.fid
31P{1H}
-135.42
-139.81
-144.20
-148.59
-152.99
f1 (ppm)

(e)

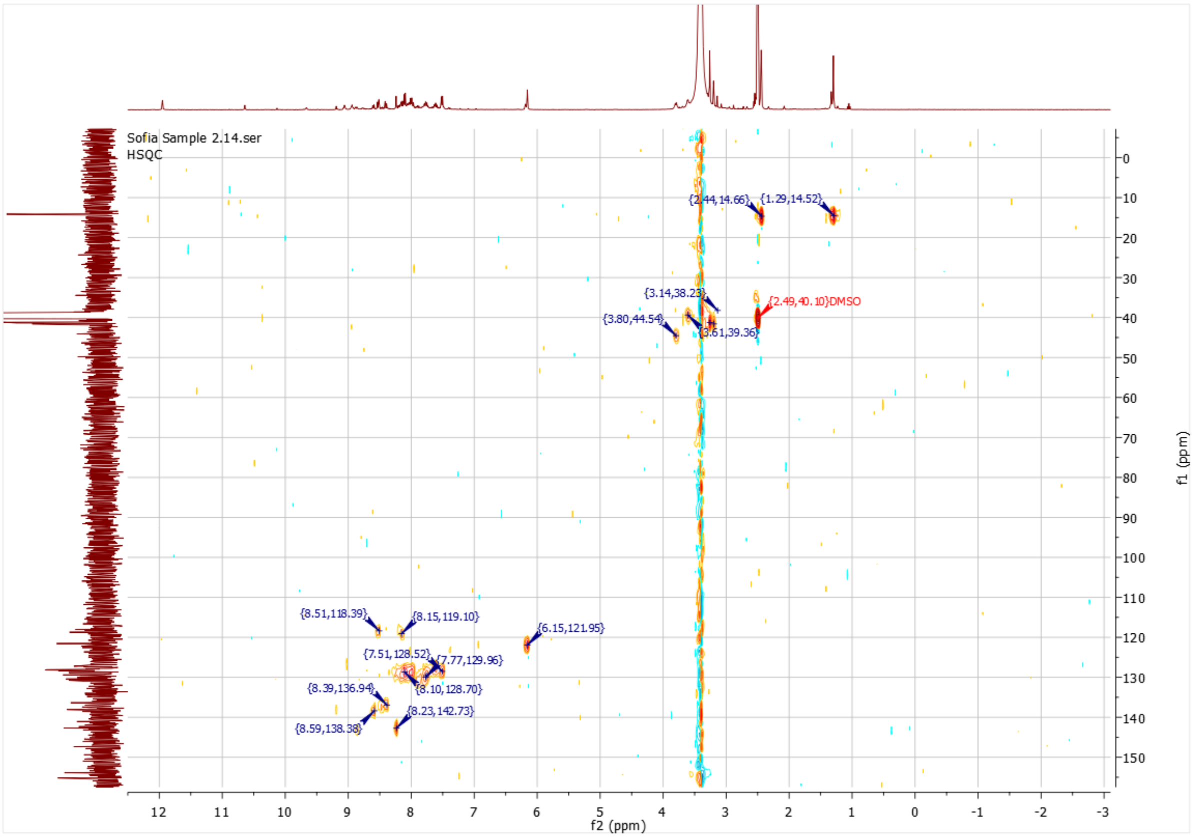
Sofia Sample 2.14.ser
HSQC
{2.44,14.66}
{1.29,14.52}
{3.14,38.23}
{2.49,40.10}DMSO
{3.80,44.54}
{3.61,39.36}
{8.51,118.39}
{8.15,119.10}
{6.15,121.95}
{7.51,128.52}
{7.77,129.96}
{8.10,128.70}
{8.39,136.94}
{8.23,142.73}
{8.59,138.38}
f2 (ppm)
f1 (ppm)

(f)

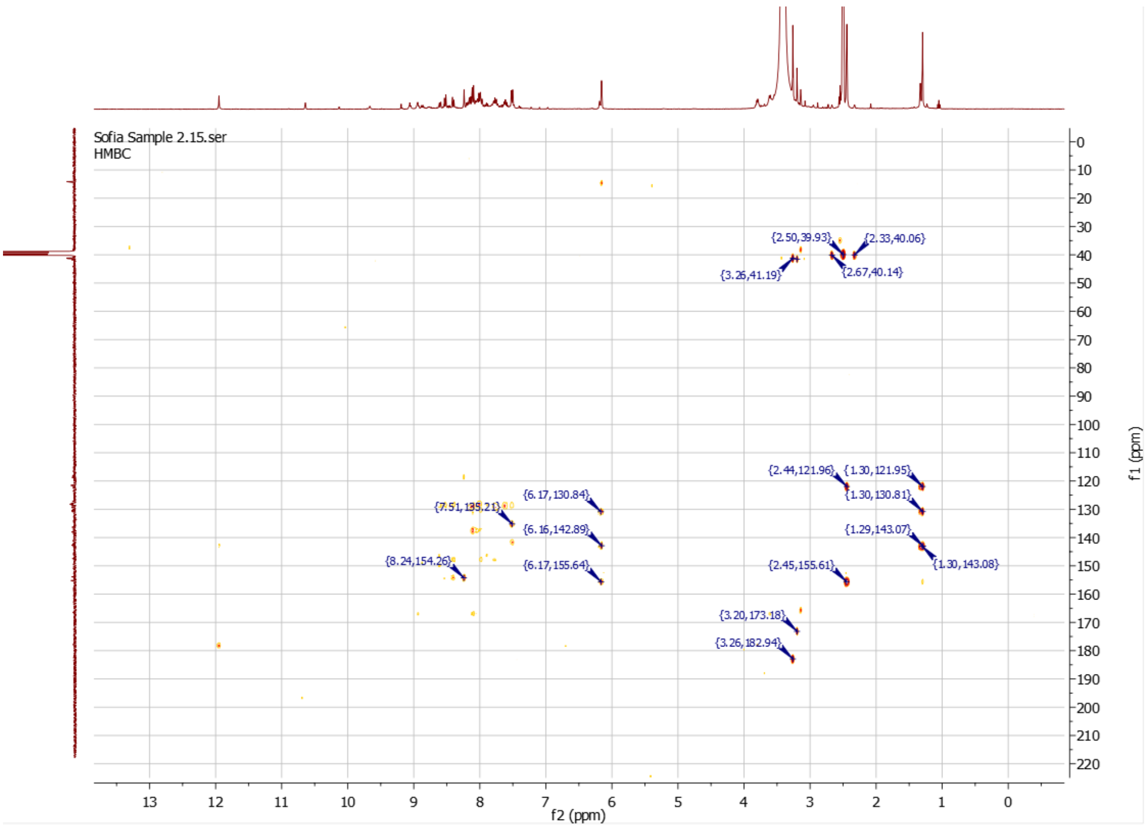
Sofia Sample 2.15.ser
HMBC
{2.50,39.93}
{2.33,40.06}
{3.26,41.19}
{2.67,40.14}
{2.44,121.96}
{1.30,121.95}
{6.17,130.84}
{1.30,130.81}
{7.51,135.21}
{6.16,142.89}
{1.29,143.07}
{1.30,143.08}
{8.24,154.26}
{6.17,155.64}
{2.45,155.61}
{3.20,173.18}
{3.26,182.94}
f2 (ppm)
f1 (ppm)

(g)

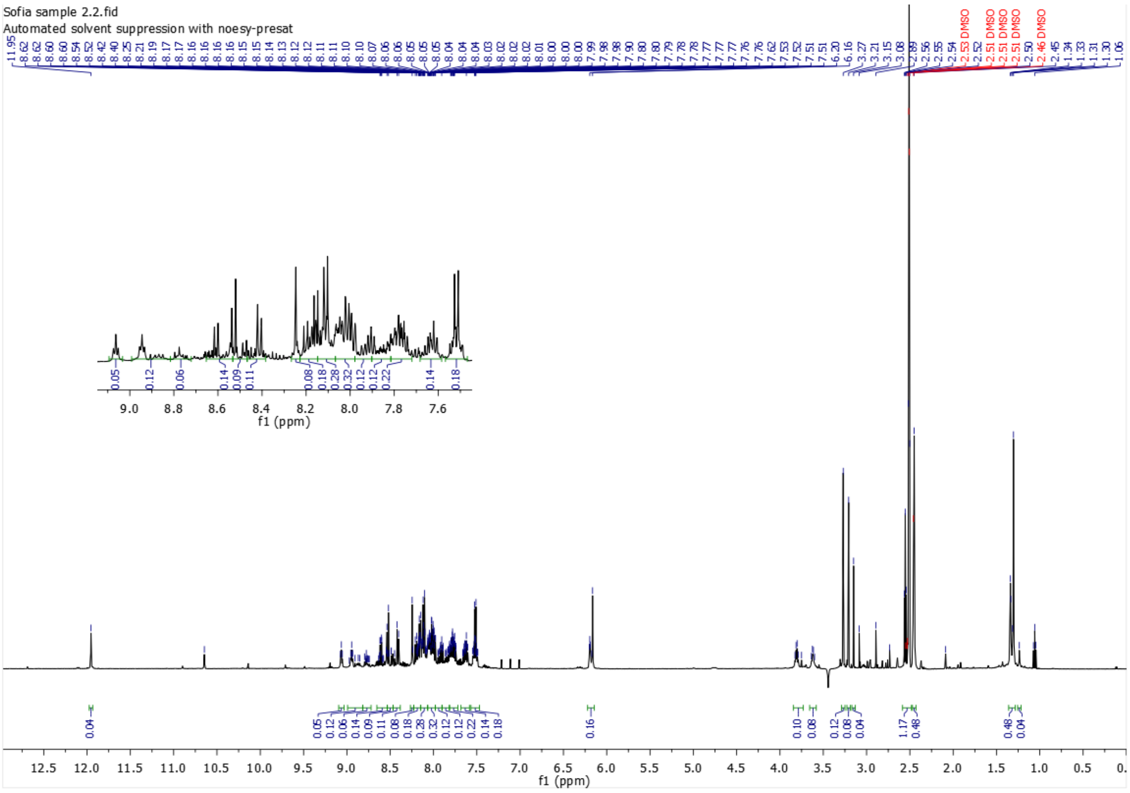

Sofia sample 2.2.fid
Automated solvent suppression with noesy-presat
f1 (ppm)

(h)

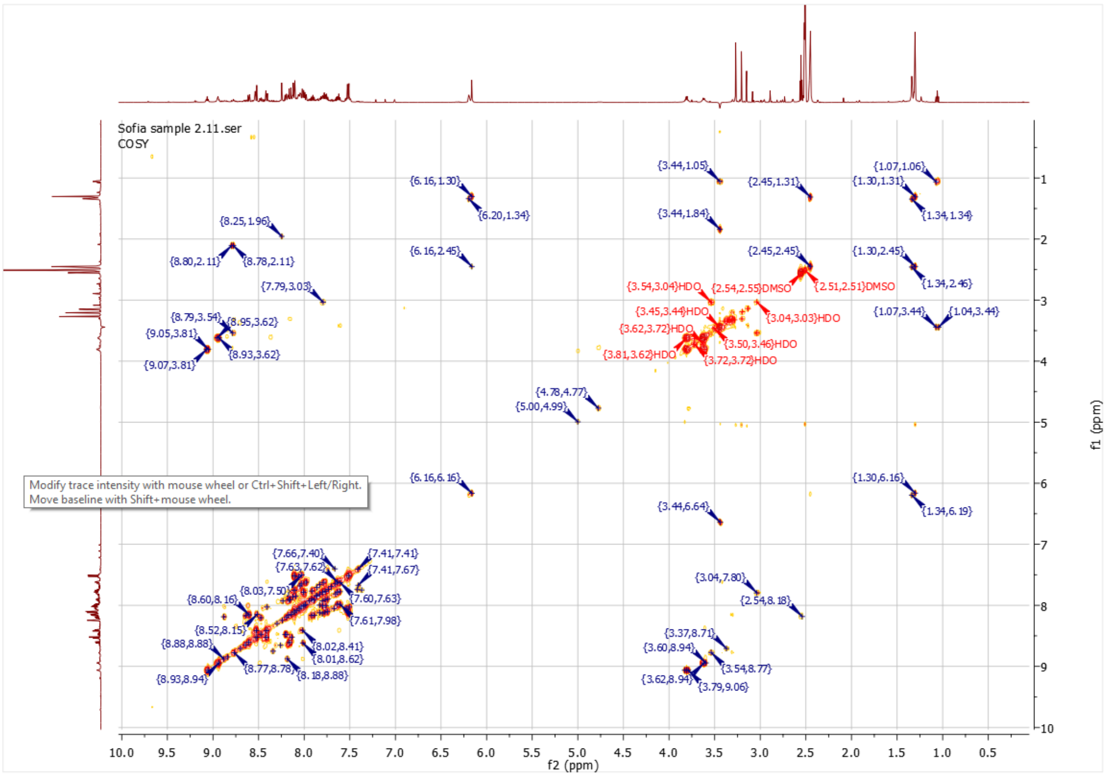

Sofia sample 2.11.ser
COSY
Modify trace intensity with mouse wheel or Ctrl+Shift+Left/Right.
Move baseline with Shift+mouse wheel.
f2 (ppm)
f1 (ppm)

(i)

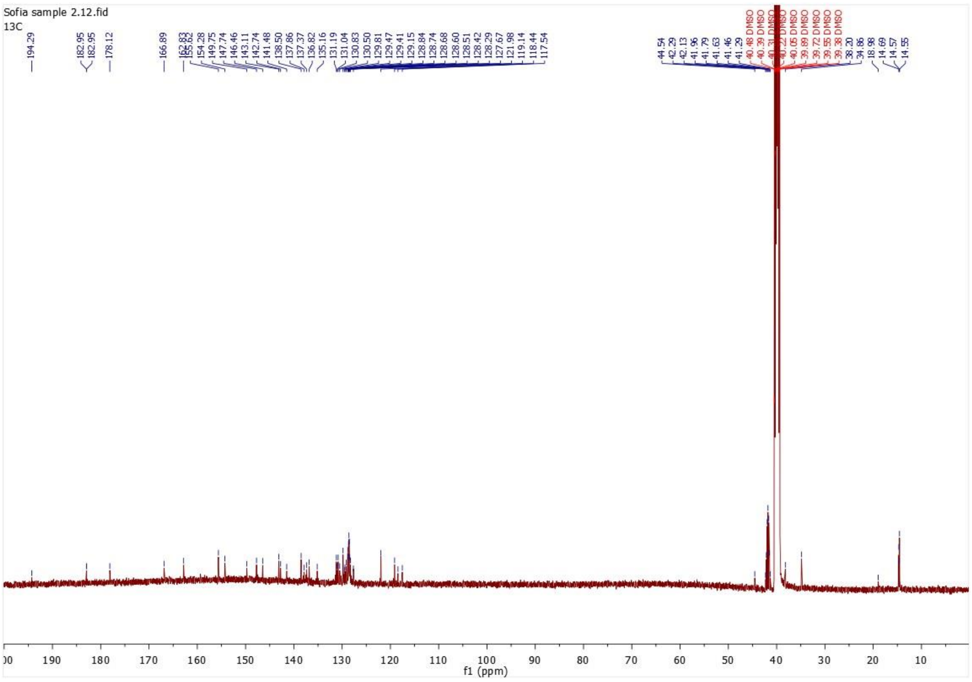
Sofia sample 2.12.fid
13C
f1 (ppm)

(j)

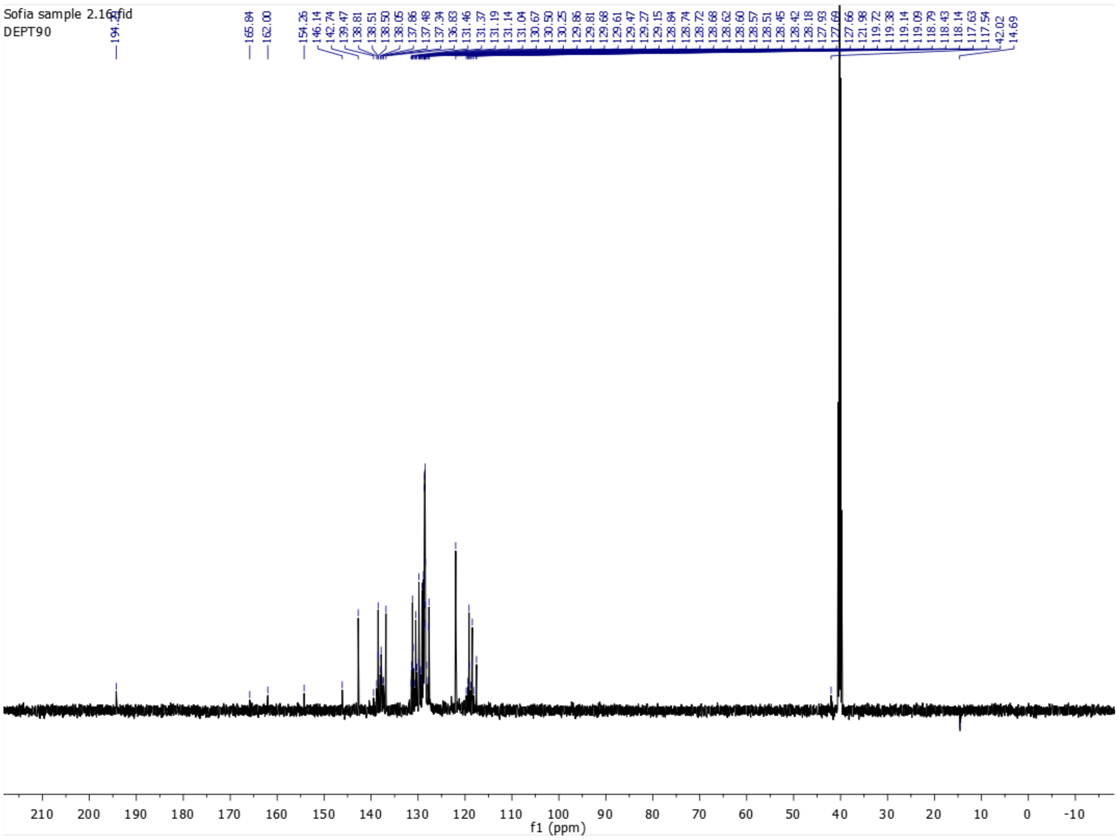
Sofia sample 2.16.fid
DEPT90
f1 (ppm)

(k)

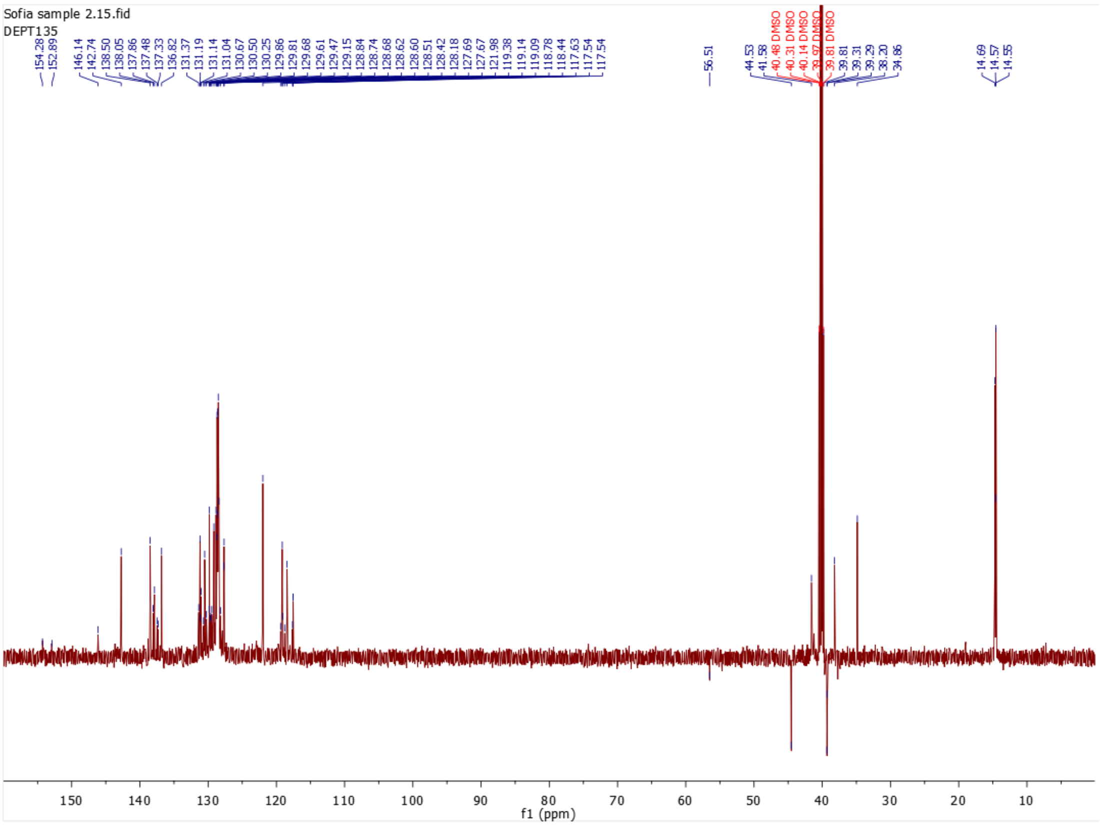
Sofia sample 2.15.fid
DEPT135
154.28
152.89
146.14
142.74
138.50
138.05
137.86
137.48
137.33
136.82
131.37
131.19
131.14
131.04
130.67
130.50
130.25
129.86
129.81
129.68
129.61
129.47
129.15
128.84
128.74
128.68
128.62
128.60
128.51
128.42
128.18
127.69
127.67
121.98
119.38
119.14
119.09
118.78
118.44
117.63
117.54
117.54
56.51
44.53
41.58
40.48 DMSO
40.31 DMSO
40.14 DMSO
39.97 DMSO
39.81 DMSO
39.81
39.31
39.29
38.20
34.86
14.69
14.57
14.55
f1 (ppm)

(l)

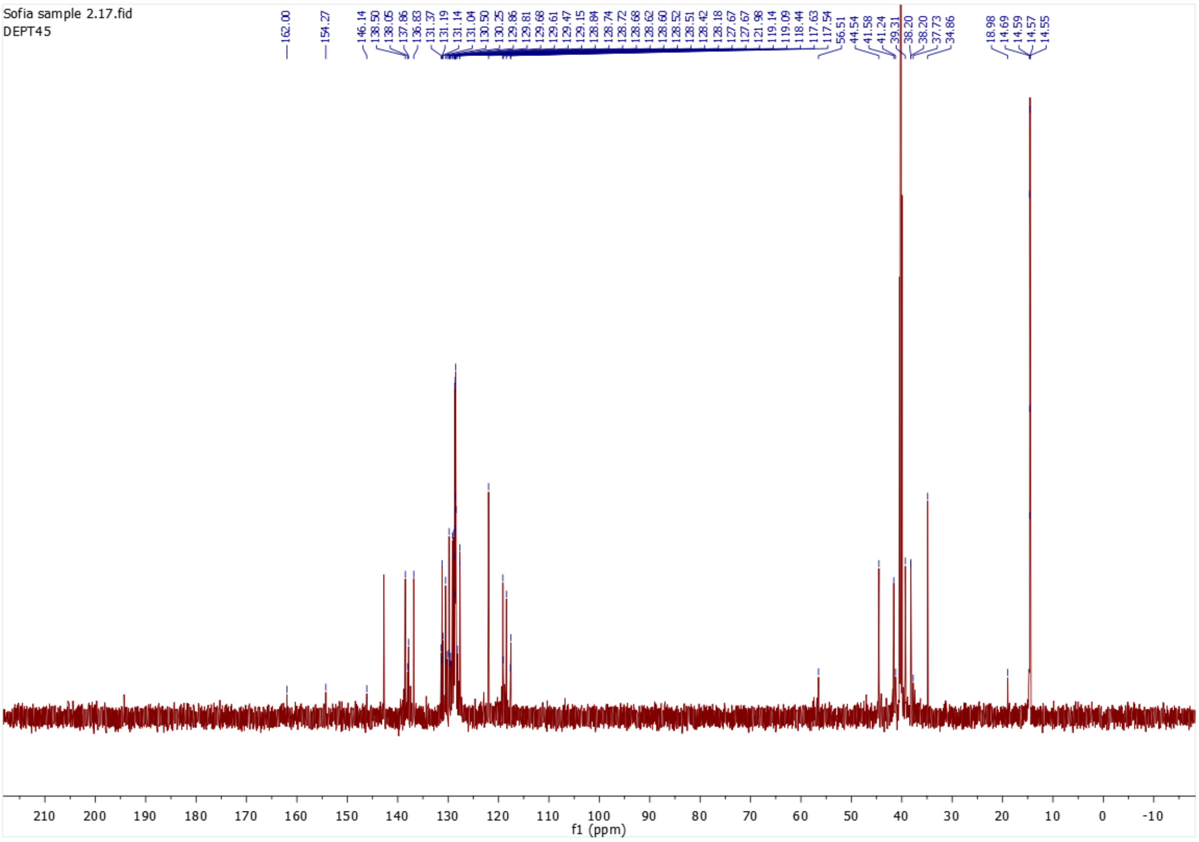
Sofia sample 2.17.fid
DEPT45
162.00
154.27
146.14
138.50
138.05
137.86
136.83
131.37
131.19
131.14
131.04
130.50
130.25
129.86
129.81
129.68
129.61
129.47
129.15
128.84
128.74
128.72
128.68
128.62
128.60
128.52
128.51
128.42
128.18
127.67
127.67
121.98
119.14
119.09
118.44
117.63
117.54
56.51
44.54
41.58
41.24
39.31
38.20
38.20
37.73
34.86
18.98
14.69
14.59
14.57
14.55
f1 (ppm)

(m)

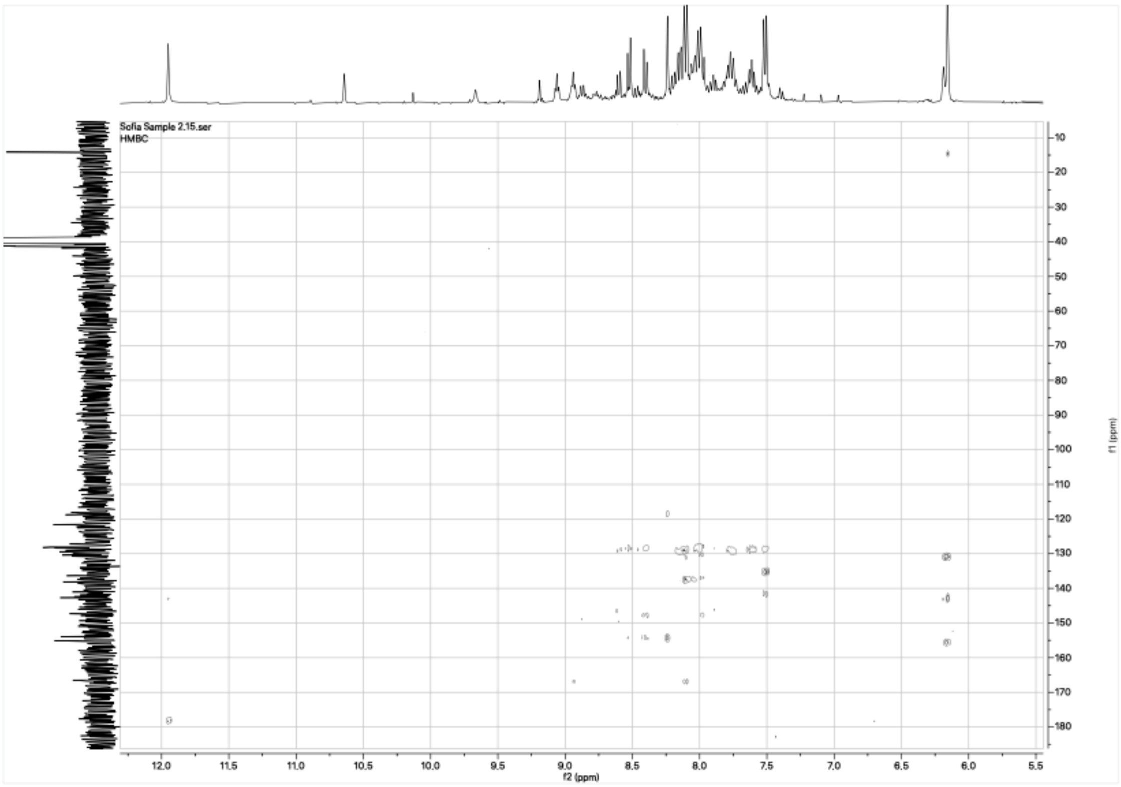

Sofia Sample 2.15.ser
HMBC
f1 (ppm)
f2 (ppm)

(n)

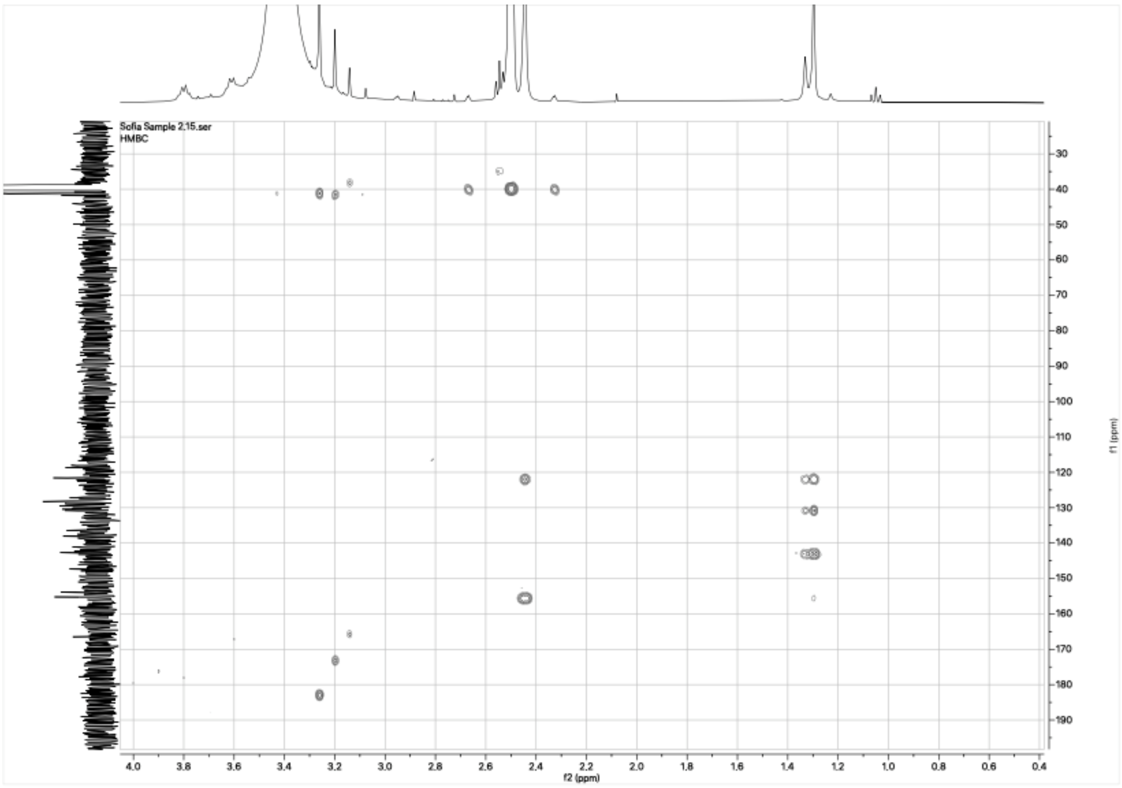

Sofia Sample 2.15.ser
HMBC
f1 (ppm)
f2 (ppm)

(o)

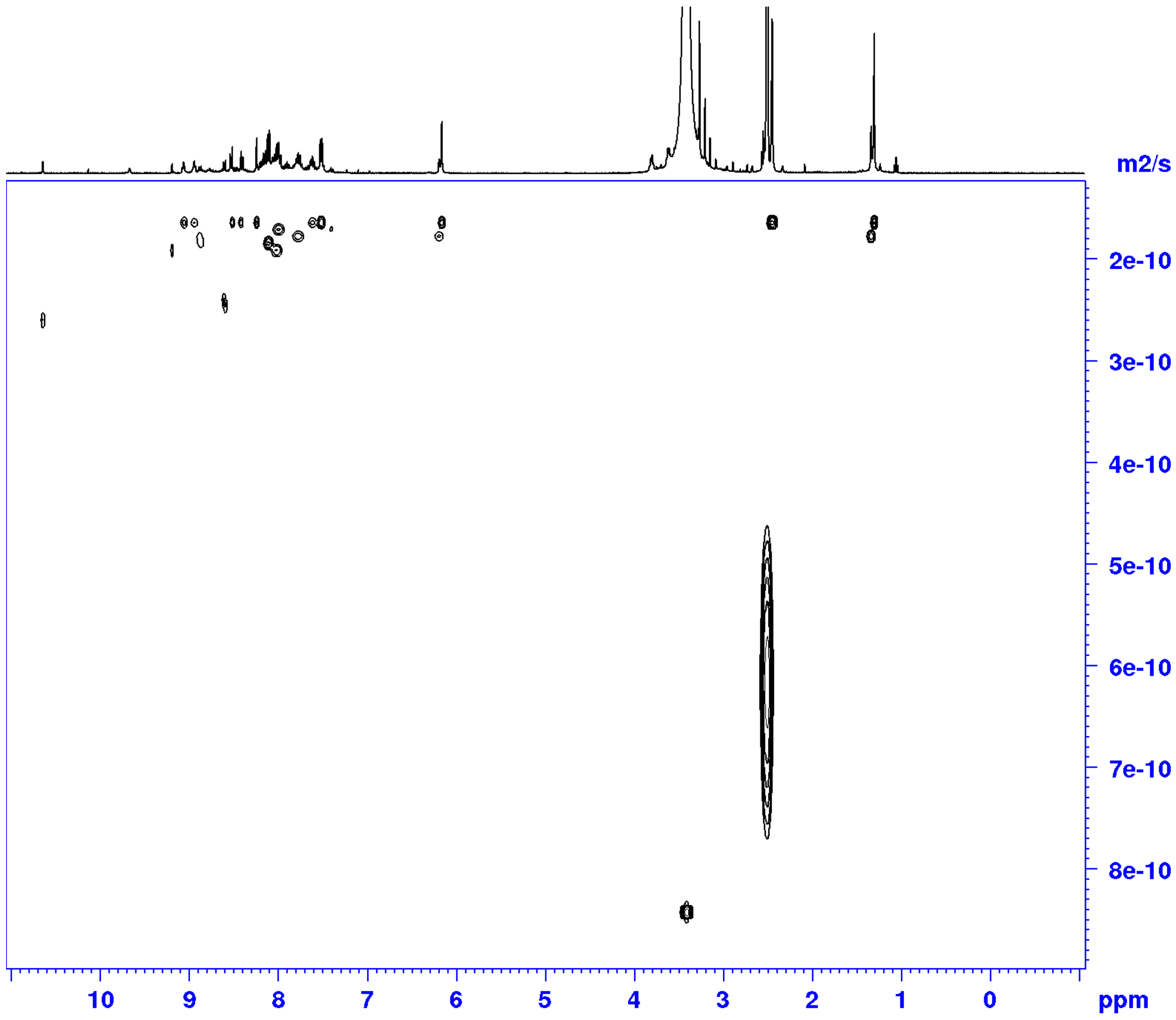

(p)

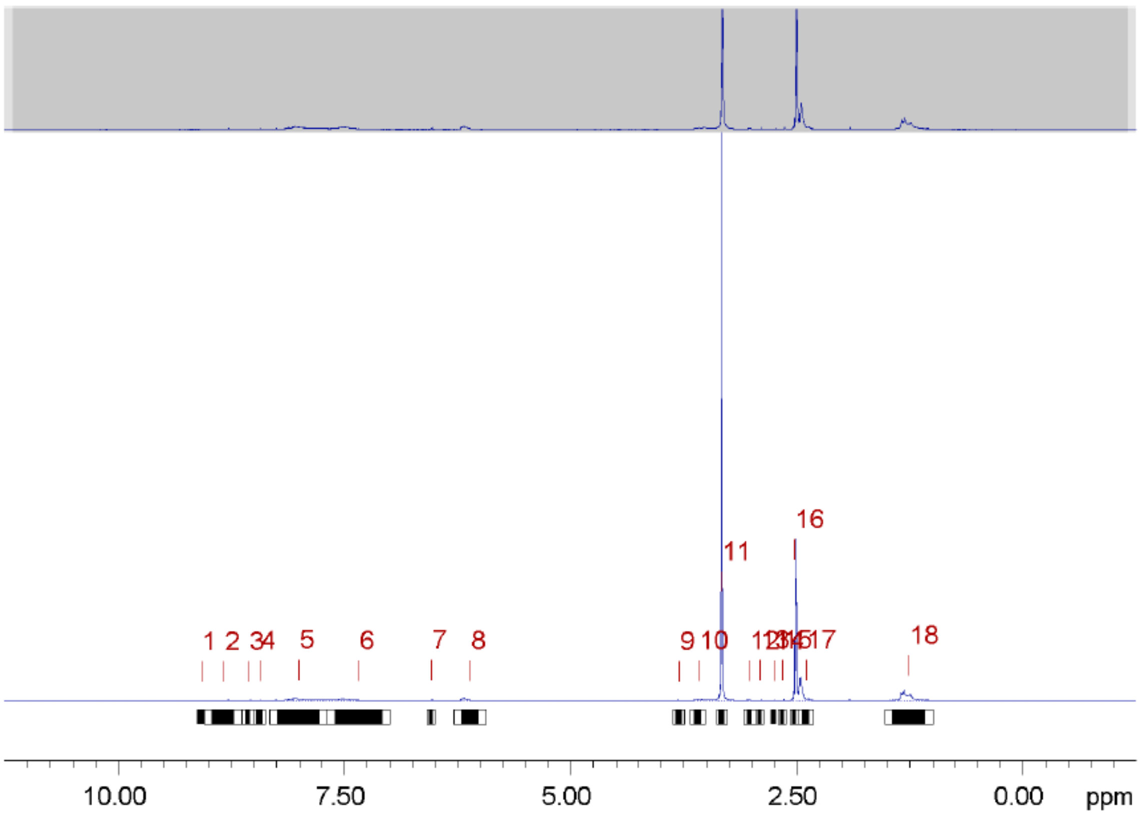

16
11
1 2 34 5 6 7 8
9 10
18
10.00
7.50
5.00
2.50
0.00
ppm

Dosy/Fit

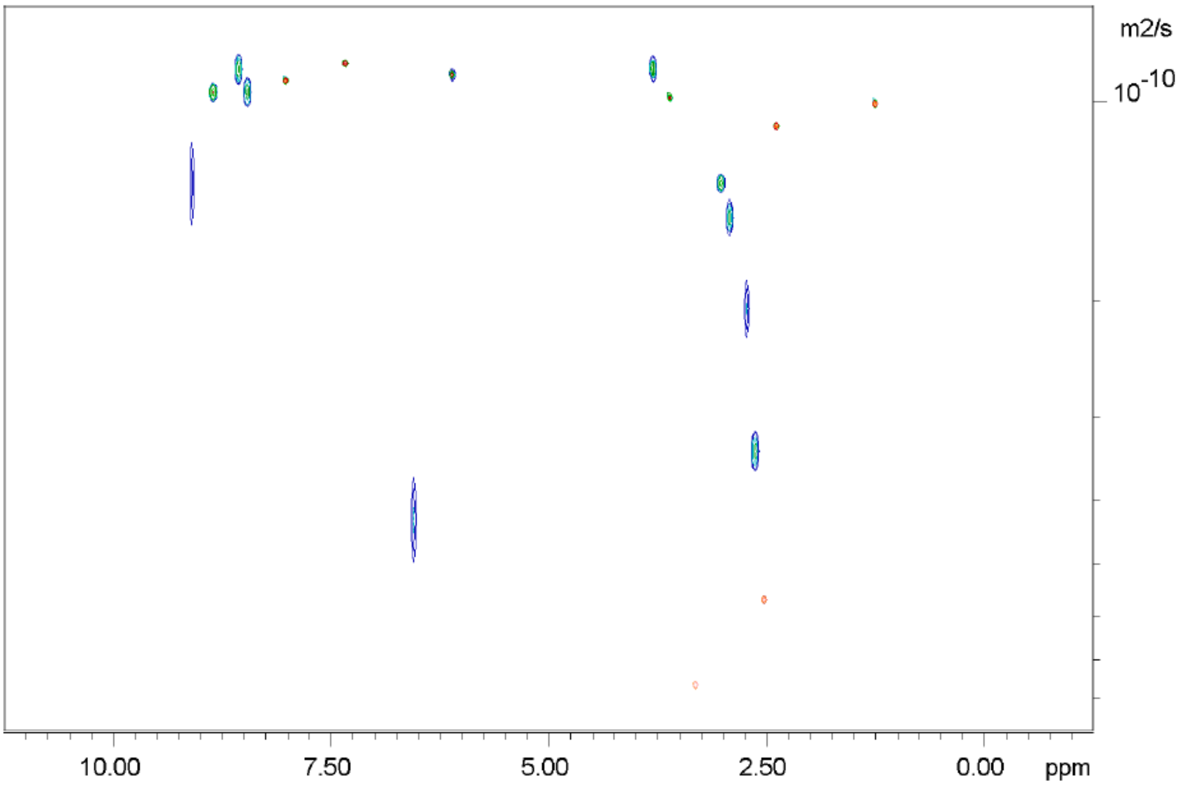

m2/s
10^{-10}
10.00
7.50
5.00
2.50
0.00
ppm

(q)

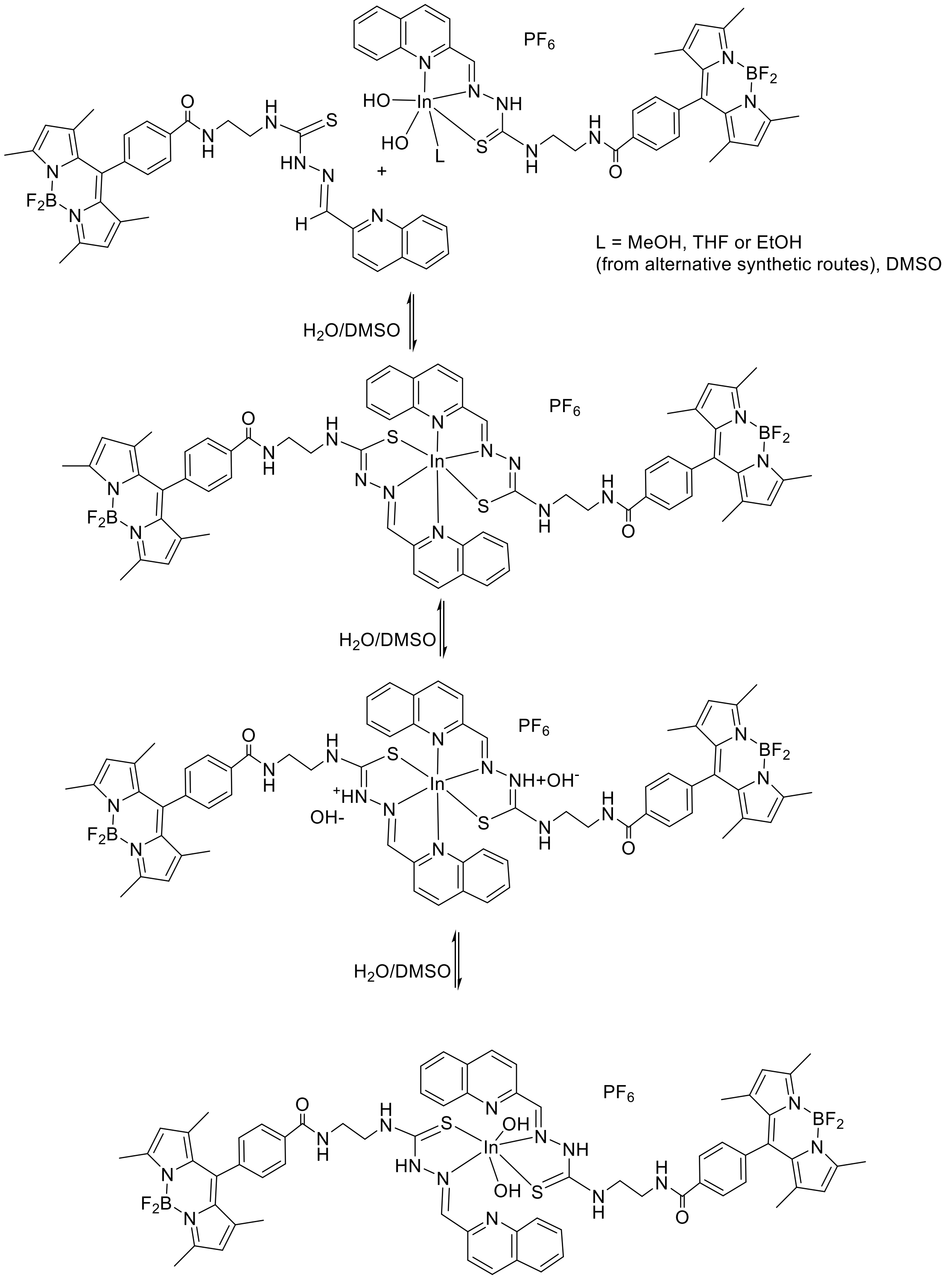

(r)

Figure S17. NMR spectroscopic characterisation of **InL4** in DMSO-d_6 in fresh (1 h) and aged solutions (monitored over several weeks). **(a)** ^{1}H NMR spectrum with expansion of aromatic

proton resonances; **(b,c)** ^{19}F NMR (376 MHz, DMSO-d_6); **(d)** ^{11}B NMR (128 MHz, DMSO-d_6); **(e)** ^{31}P NMR; **(f)** ^{1}H–^{13}C HSQC; **(g)** ^{1}H–^{13}C HMBC with relevant expansions. Aged solutions (1 month, with solvent suppression): **(h)** ^{1}H NMR; **(i)** ^{1}H–^{1}H COSY. **(j–m)** ^{13}C{^{1}H} and DEPT experiments enabling assignment of the major species persisting over weeks. **(n)** DOSY spectra of **InL4** recorded after 1 h in solution **(p)**, and in 1 week-aged DMSO-d_6 solutions **(q)**; **(r)** Proposed speciation based on NMR data from samples monitored over 1 month. Additional to the occurrence of a resonance at 10-12 ppm corresponding to protonation of the hydrazinic sites in aqueous DMSO, a small 1:1:1 triplet is observed in ^{1}H NMR spectra of aged DMSO/H_2O solutions, absent in fresh samples; this signal collapses upon solvent suppression and is tentatively assigned to quinoline protonation or NH_4^+ formation. This compound is only very weakly soluble in CD_3CN and $CDCl_3$. Its behaviour is consistent with the slow thiosemicarbazone cyclisation and/or decomposition mediated by In(III), which in wet media forms $In(OH)_x$ species, accesses coordination numbers >6, and generates hydrolytic oligomers and mildly acidic solutions [Ref 10-21].

4. Selected Vibrational Spectroscopy Data

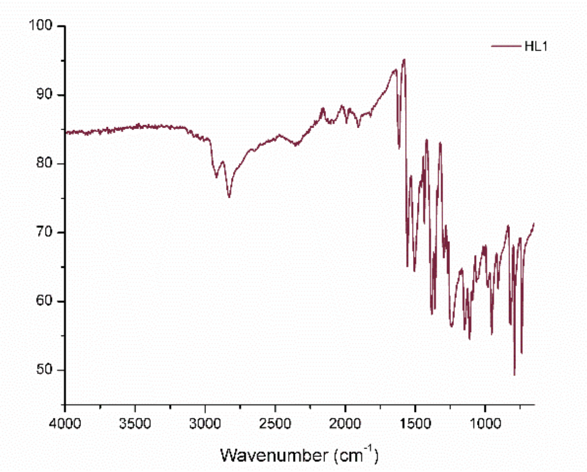

(a)

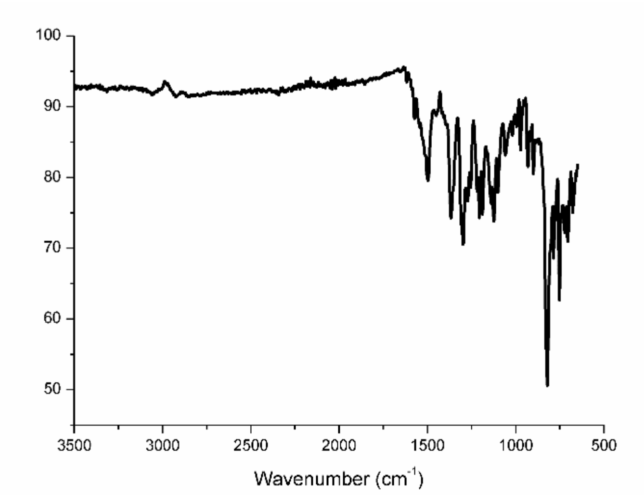

(b)

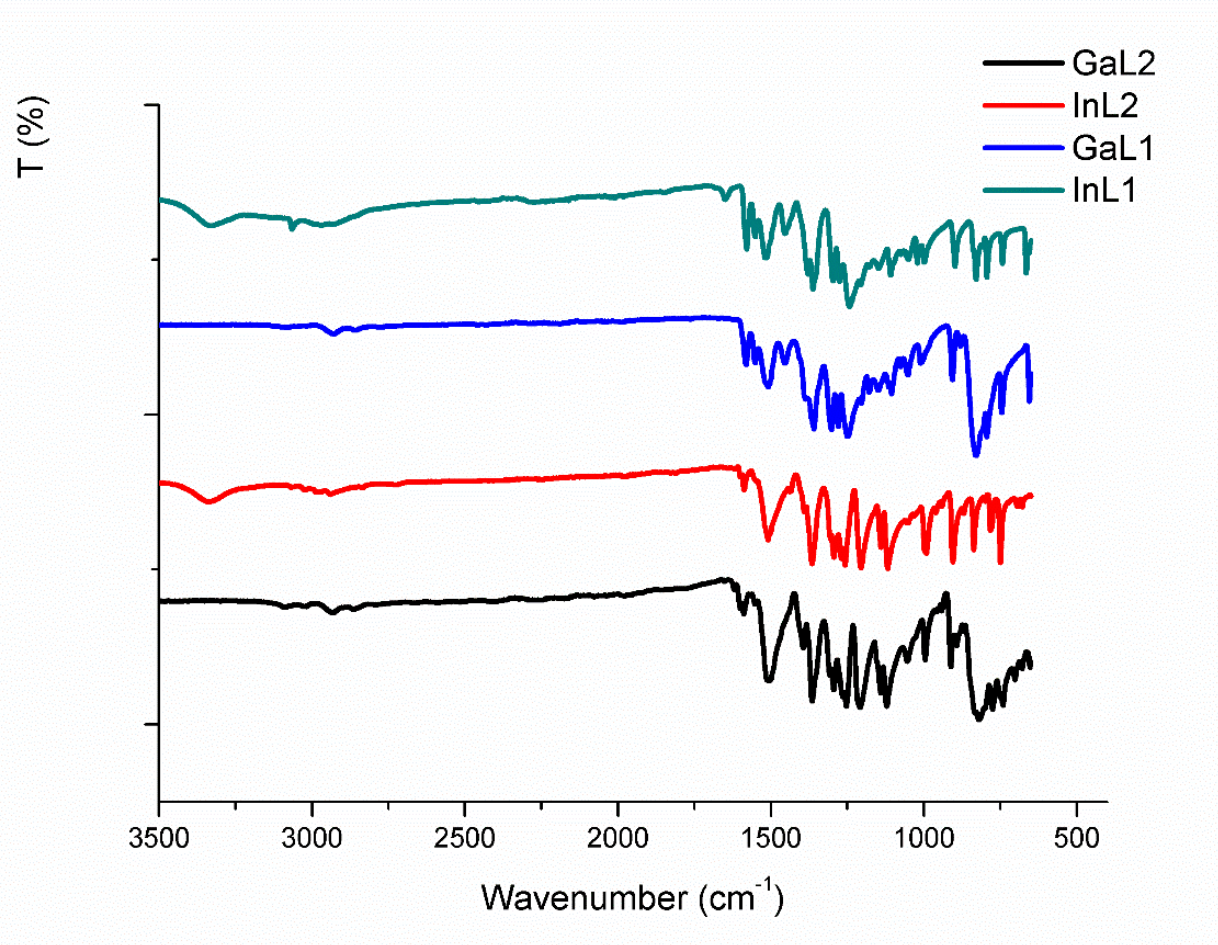

GaL2
InL2
GaL1
InL1
T (%)
3500
3000
2500
2000
1500
1000
500
Wavenumber (cm^{-1})

(c)

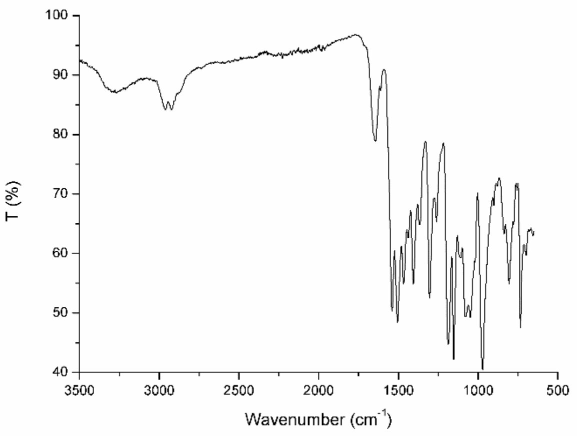
T (%)
Wavenumber (cm-1)

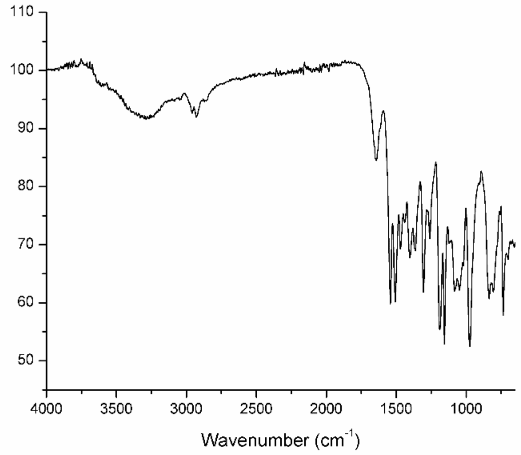
Wavenumber (cm-1)

(d)

(e)

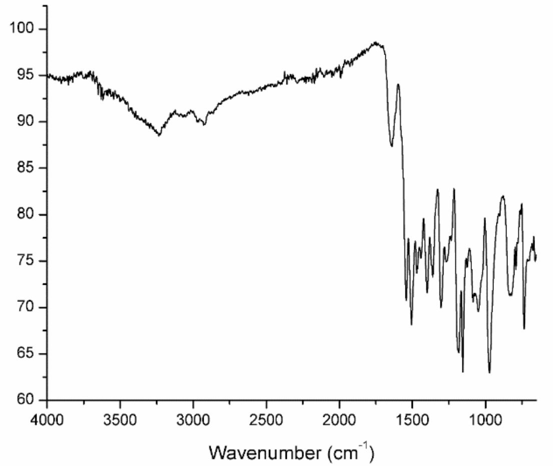
Wavenumber (cm-1)

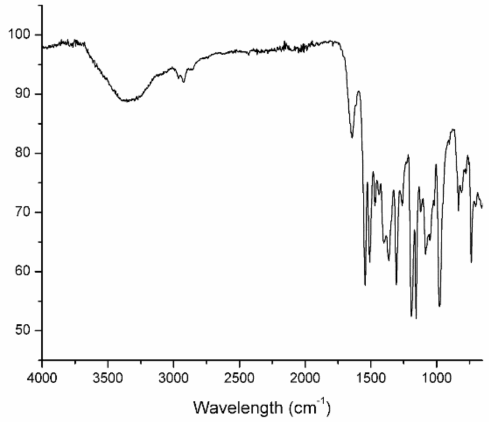
Wavelength (cm-1)

(f)

(g)

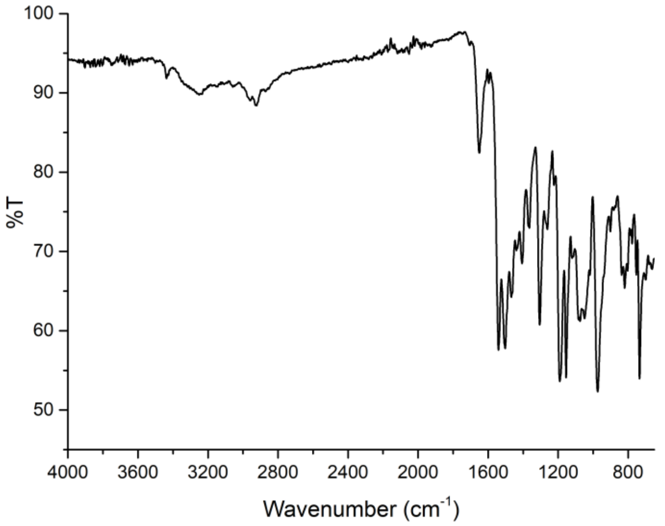
100
90
80
70
60
50
%T
4000
3600
3200
2800
2400
2000
1600
1200
800
Wavenumber (cm-1)

(h)

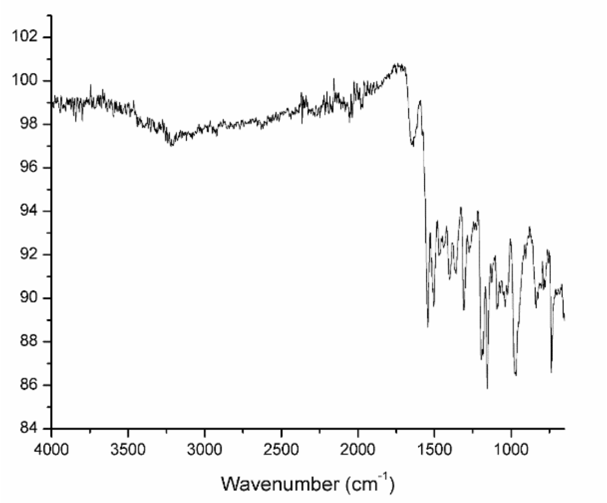
102
100
98
96
94
92
90
88
86
84
4000
3500
3000
2500
2000
1500
1000
Wavenumber (cm-1)

(i)

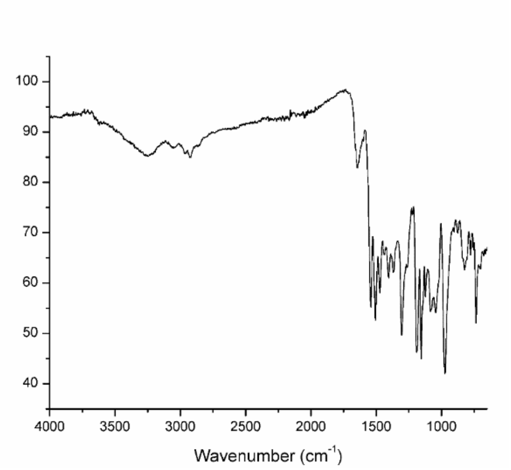
100
90
80
70
60
50
40
4000
3500
3000
2500
2000
1500
1000
Wavenumber (cm-1)

(j)

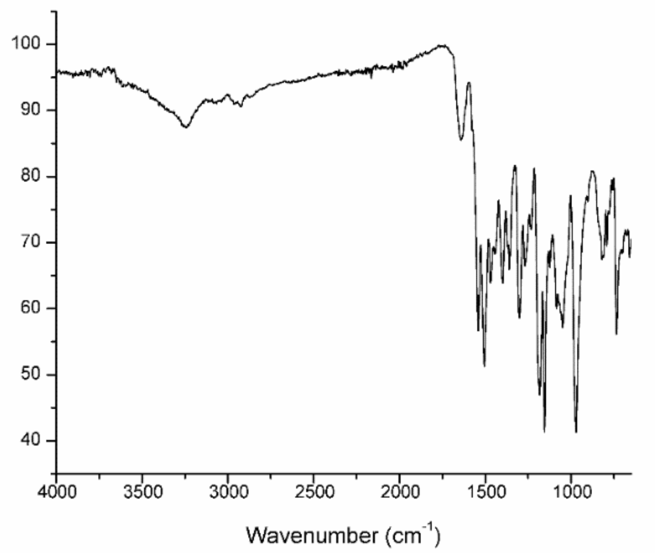

(k)

Figure S.18. Solid state IR spectroscopy of compounds: **HL1 (a)**; **HL2 (b)**; overlay of corresponding Ga(III) and Indium(III) complexes of **HL1** and **HL2 (c)**; **HL3 (d)**, **GaL3 (e)**, **InL3 (f)**, **FeL3 (g)**, **HL4 (h)**, **GaL4 (i)**, **InL4 (j)**, **FeL4 (k).**

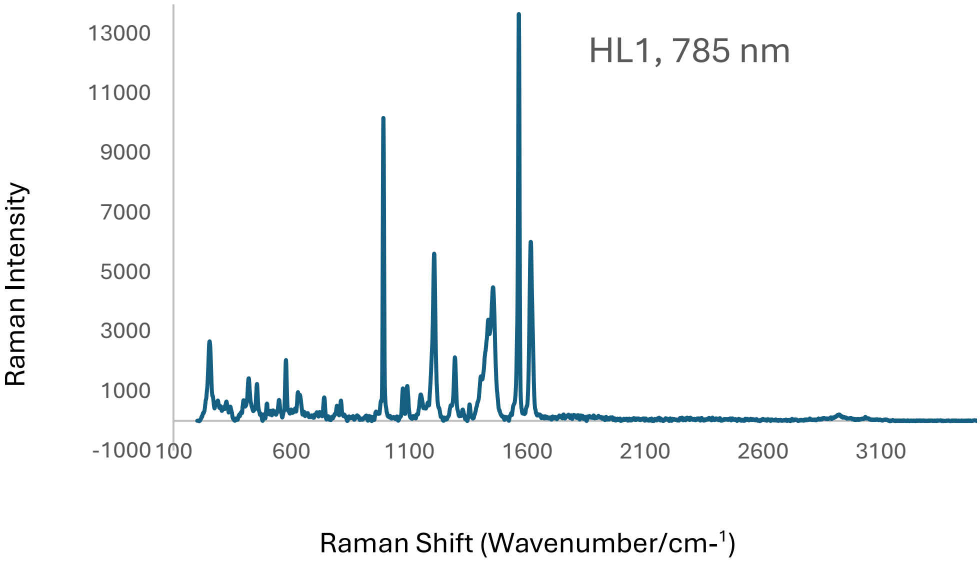

(a)

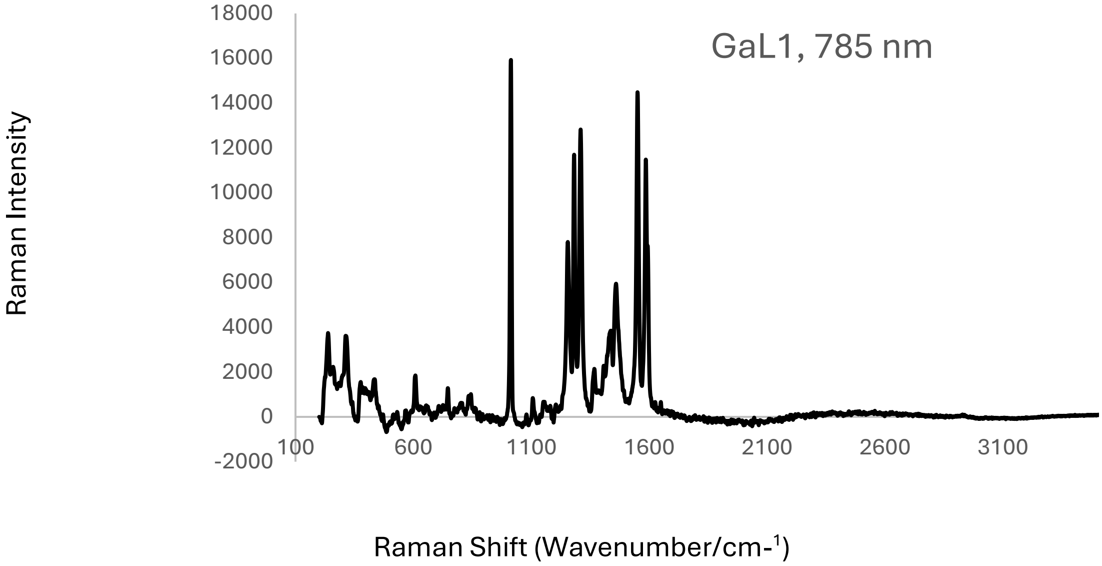
GaL1, 785 nm
Raman Intensity
18000
16000
14000
12000
10000
8000
6000
4000
2000
0
-2000
100
600
1100
1600
2100
2600
3100
Raman Shift (Wavenumber/cm-1)

(b)

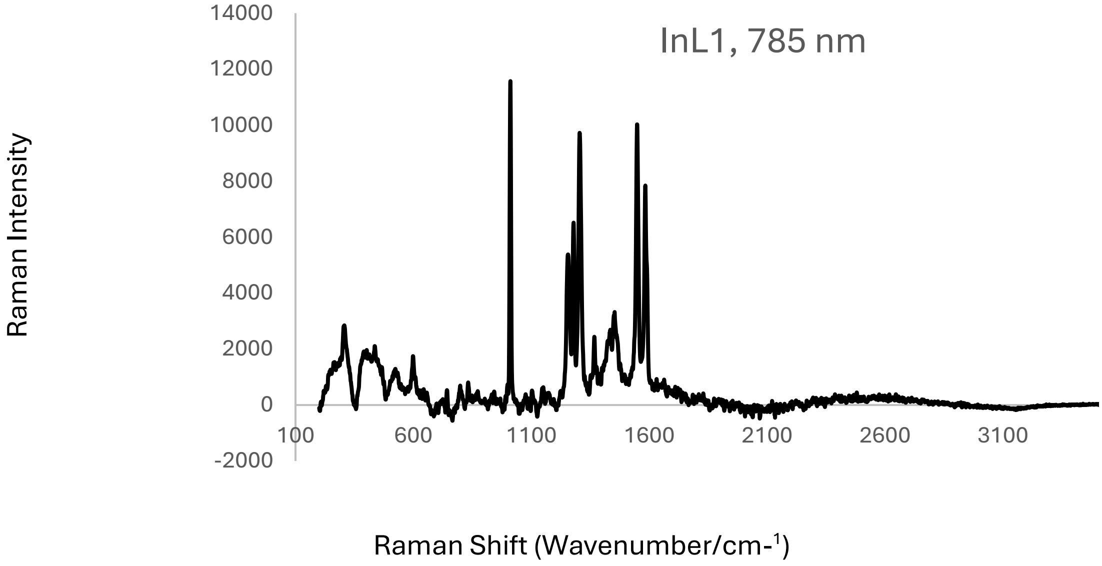
InL1, 785 nm
Raman Intensity
14000
12000
10000
8000
6000
4000
2000
0
-2000
100
600
1100
1600
2100
2600
3100
Raman Shift (Wavenumber/cm-1)

(c)

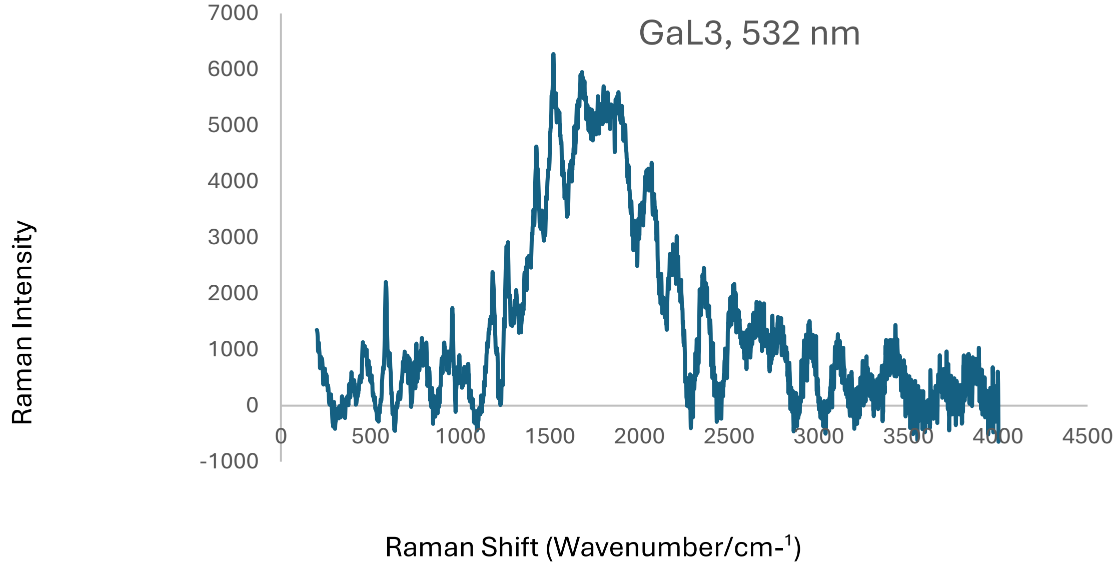
GaL3, 532 nm
Raman Intensity
7000
6000
5000
4000
3000
2000
1000
0
-1000
0
500
1000
1500
2000
2500
3000
3500
4000
4500
Raman Shift (Wavenumber/cm-1)

(d)

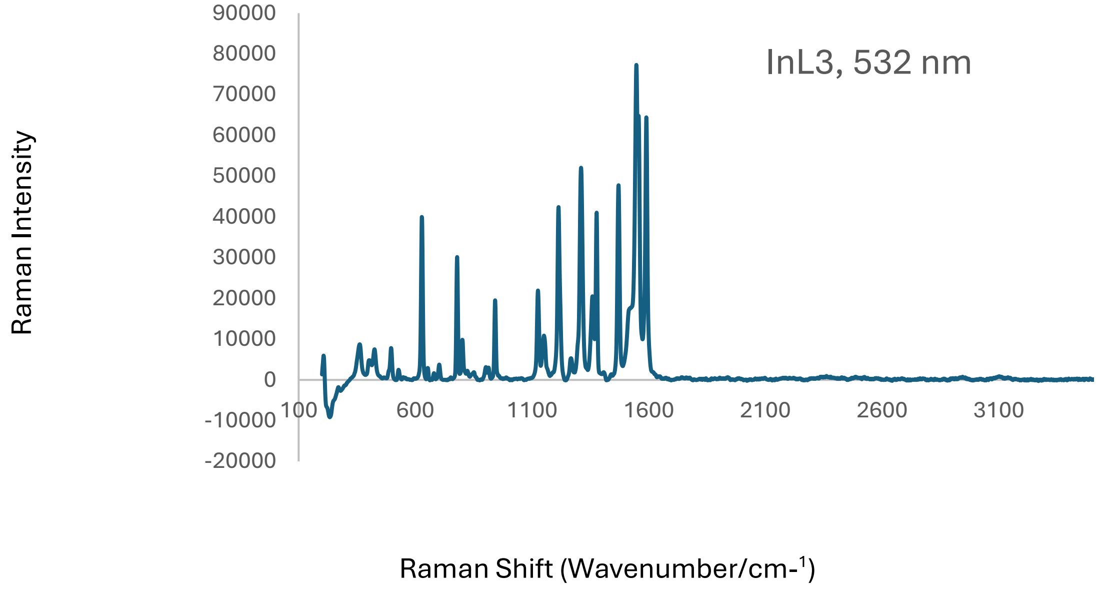
InL3, 532 nm
Raman Intensity
90000
80000
70000
60000
50000
40000
30000
20000
10000
0
-10000
-20000
100
600
1100
1600
2100
2600
3100
Raman Shift (Wavenumber/cm-1)

(e)

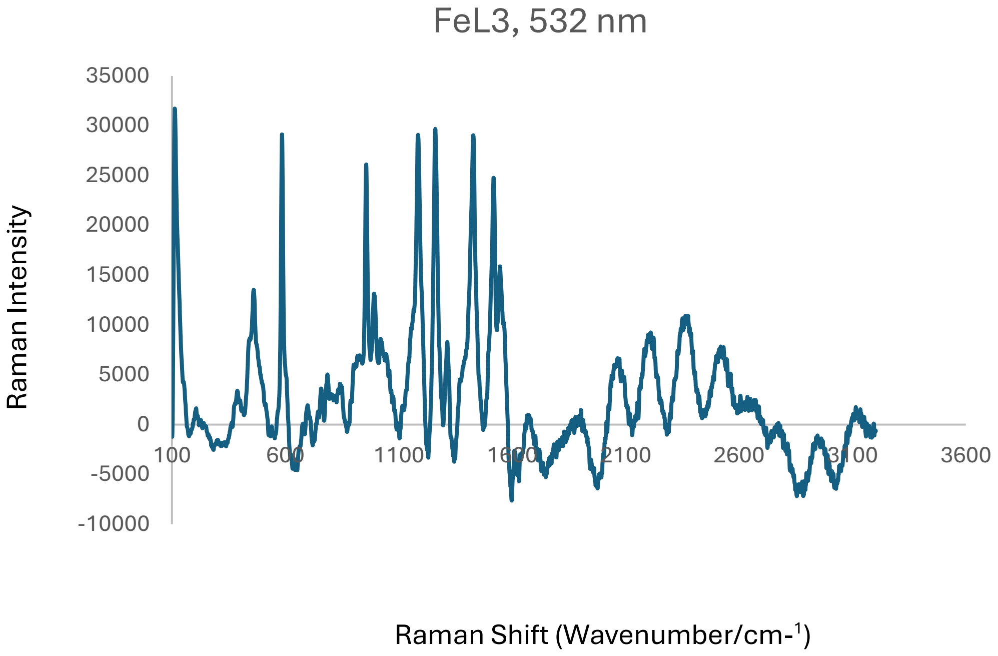
FeL3, 532 nm
35000
30000
25000
20000
15000
10000
5000
0
-5000
-10000
100
600
1100
1600
2100
2600
3100
3600
Raman Intensity
Raman Shift (Wavenumber/cm-1)

(f)

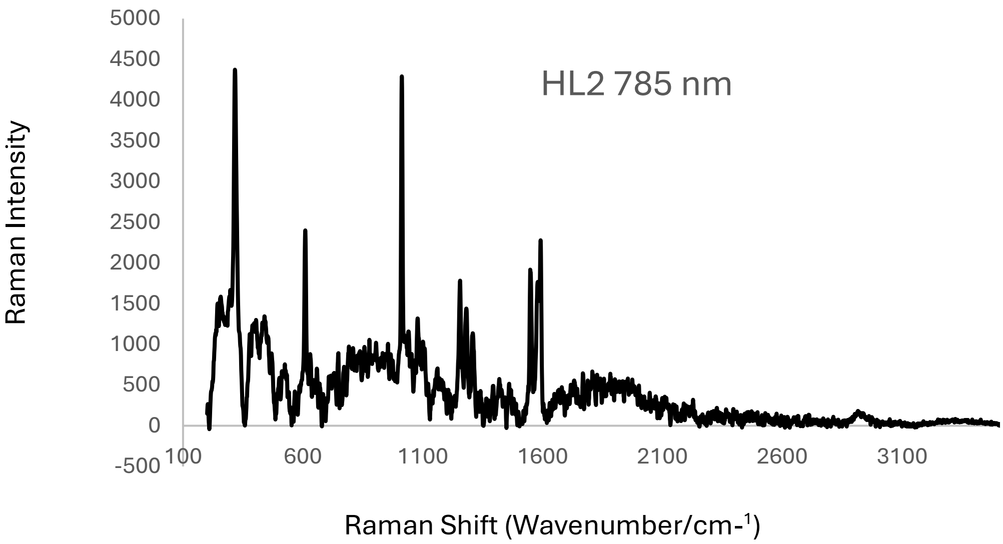
HL2 785 nm
5000
4500
4000
3500
3000
2500
2000
1500
1000
500
0
-500
100
600
1100
1600
2100
2600
3100
Raman Intensity
Raman Shift (Wavenumber/cm-1)

(g)

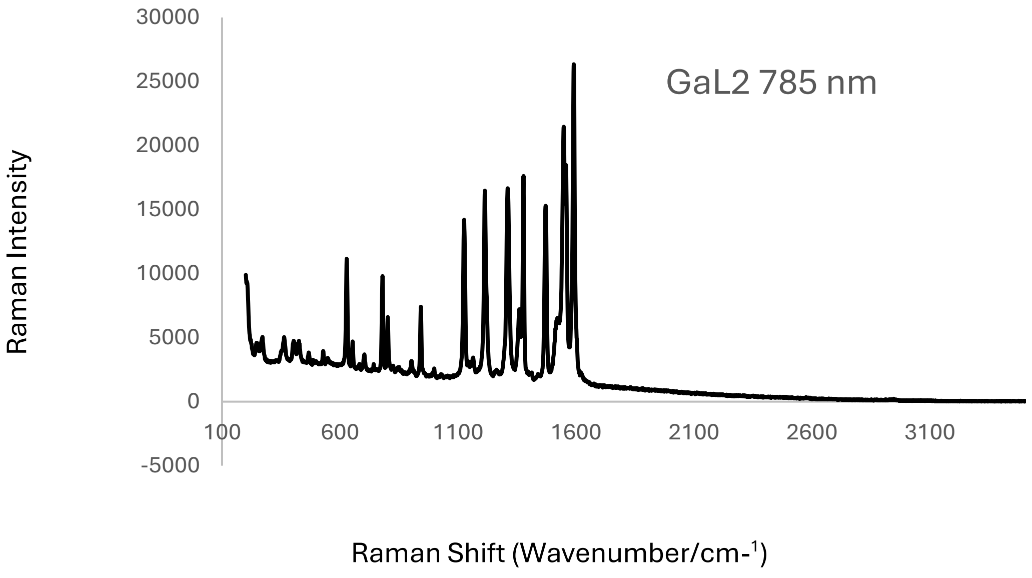
GaL2 785 nm
Raman Intensity
30000
25000
20000
15000
10000
5000
0
-5000
100
600
1100
1600
2100
2600
3100
Raman Shift (Wavenumber/cm-1)

(h)

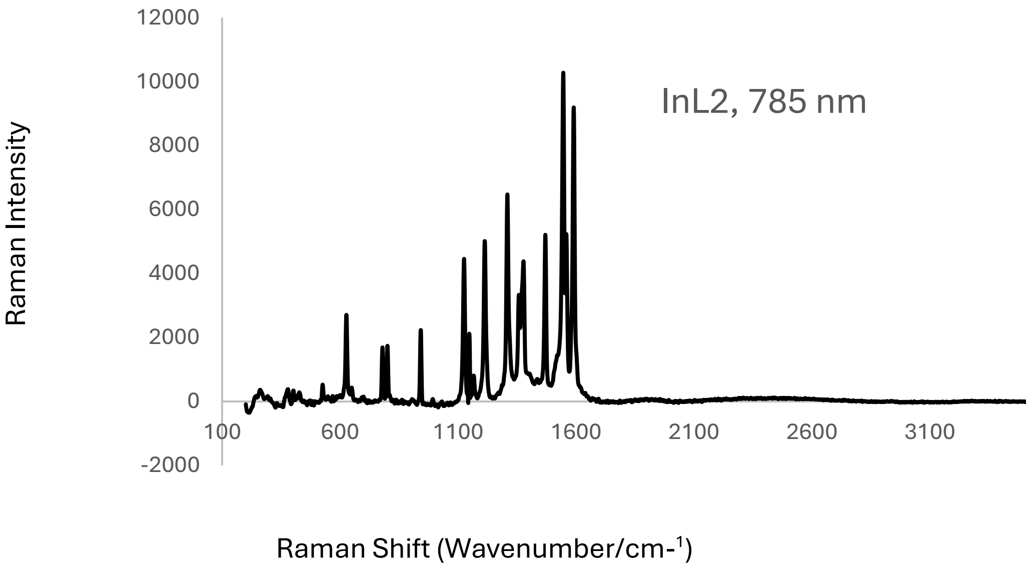
InL2, 785 nm
Raman Intensity
12000
10000
8000
6000
4000
2000
0
-2000
100
600
1100
1600
2100
2600
3100
Raman Shift (Wavenumber/cm-1)

(i)

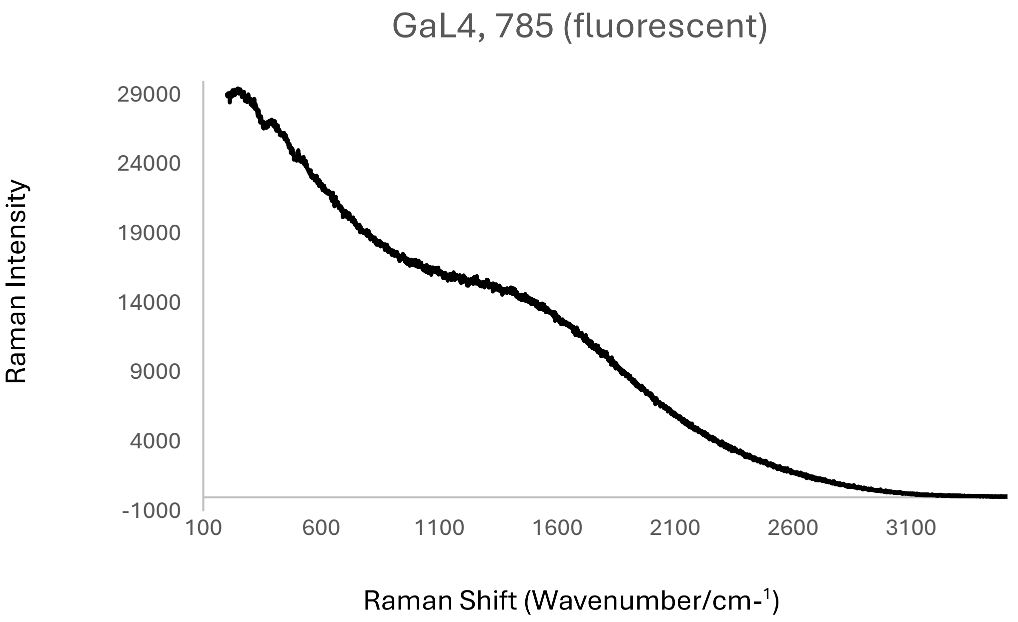

(j)

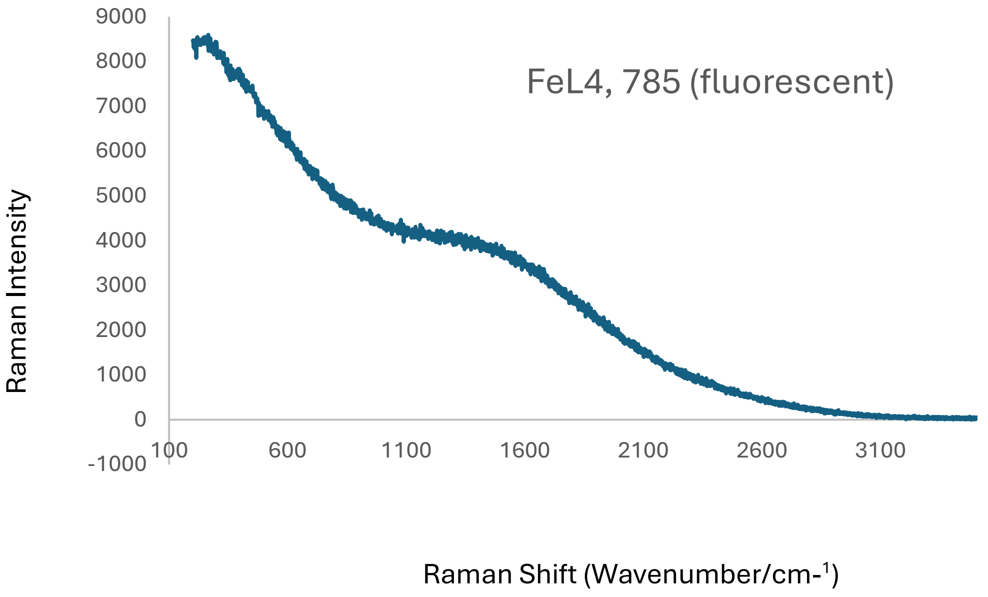

(k)

Figure S.19. Solid state Raman spectroscopy of selected compounds Excitation was either using a 785 or 532nm laser depending on the amount of photoluminescence. Additionally, various laser powers were used in a tuneable mode as required depending on the amount of photoluminescence.

5. Selected ESI-Mass Spectrometry Data

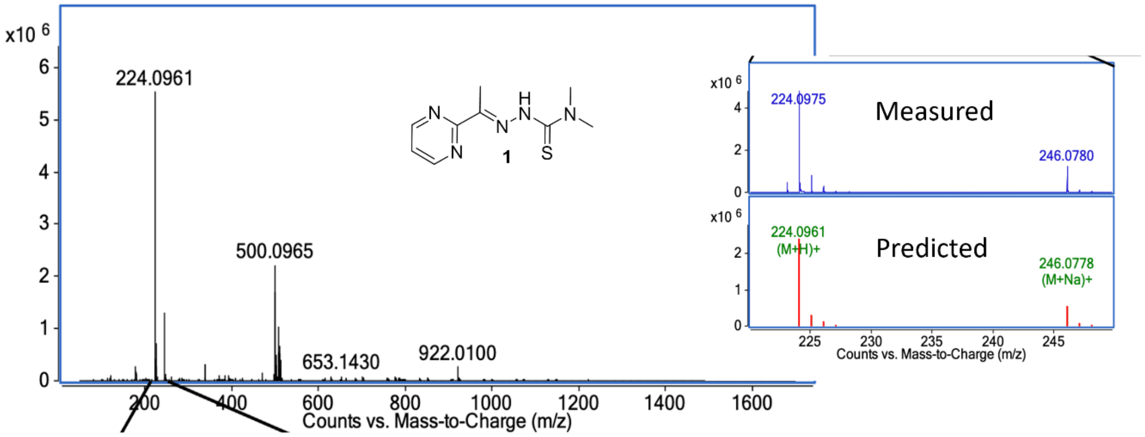

(a)

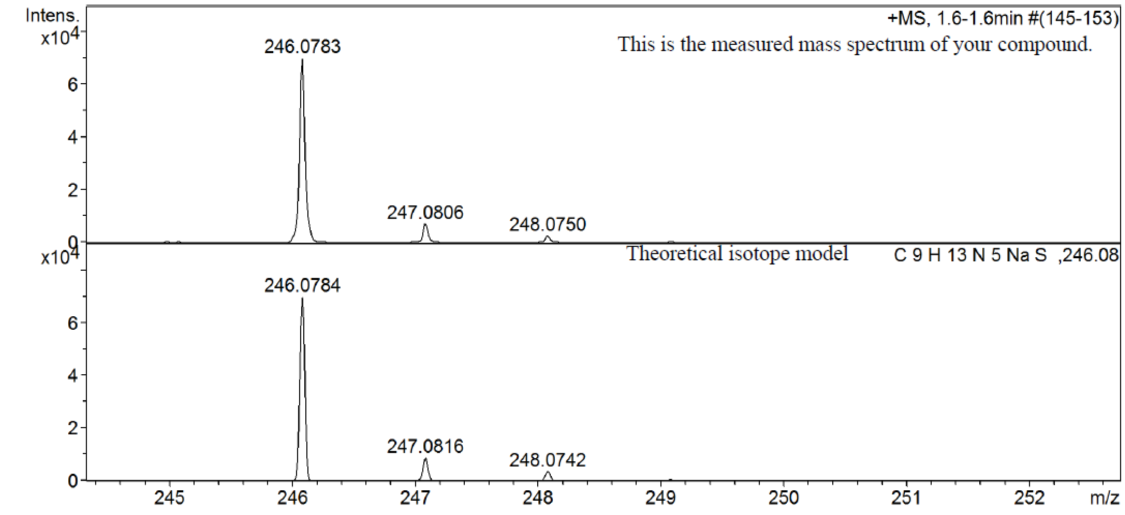

Meas. m/z	#	Formula	m/z	err [ppm]	Mean err [ppm]	rdb	e⁻ Conf	mSigma
246.0783								
	1	C 9 H 13 N 5 Na S	246.0784	0.4	0.6	5.5	even	13.68

(b)

Figure S.20. (a) Positive mode ESI-mass spectrometry of compound **HL1**. Insert shows predicted m/z value and isotopic pattern for the selected compounds formula (b) Accurate mass of **HL1.**

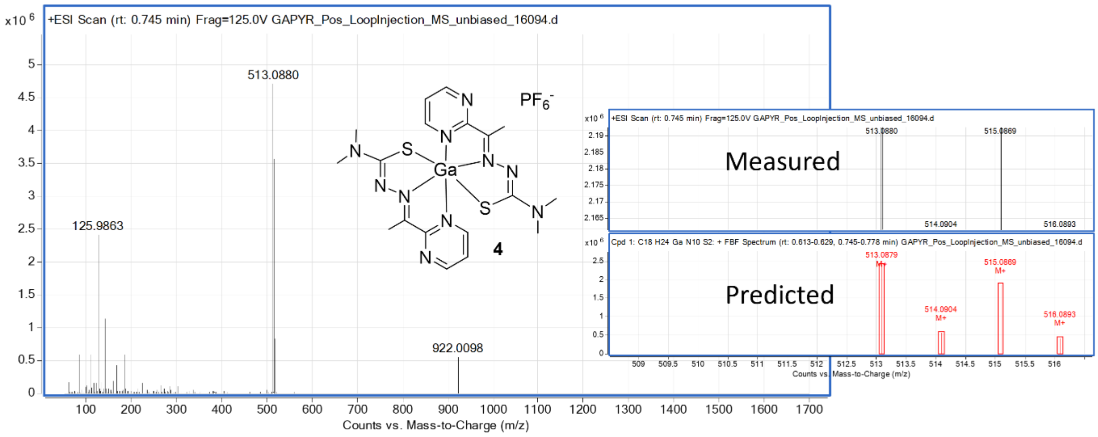

Figure S.21. Positive mode ESI-mass spectrometry of complex **GaL1**. Insert shows predicted m/z value and isotopic pattern for the selected compounds formula.

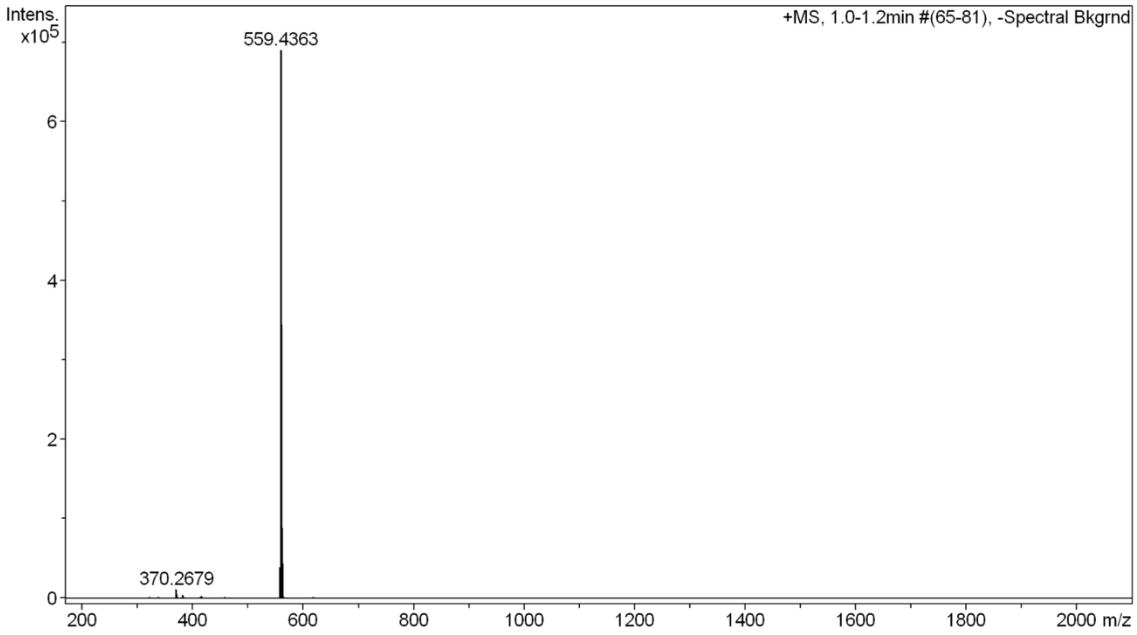

(a)

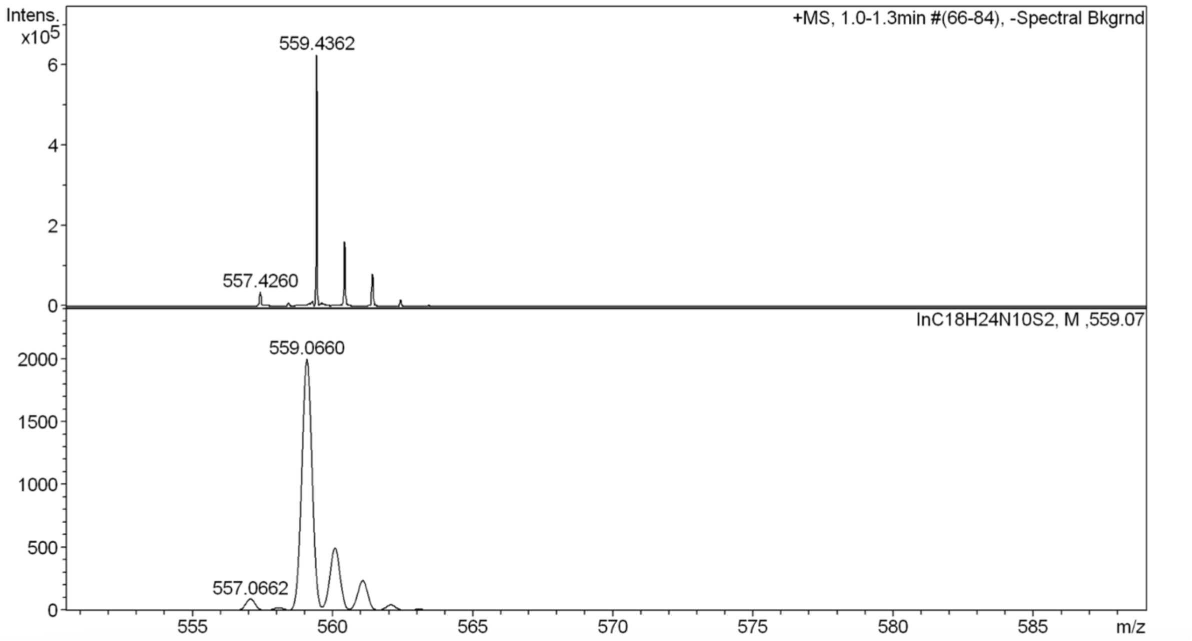

(b)

Figure S.22. Positive mode ESI-mass spectrometry of complex **InL1 (a)**. Insert shows predicted m/z value and isotopic pattern for the selected compounds formula (b).

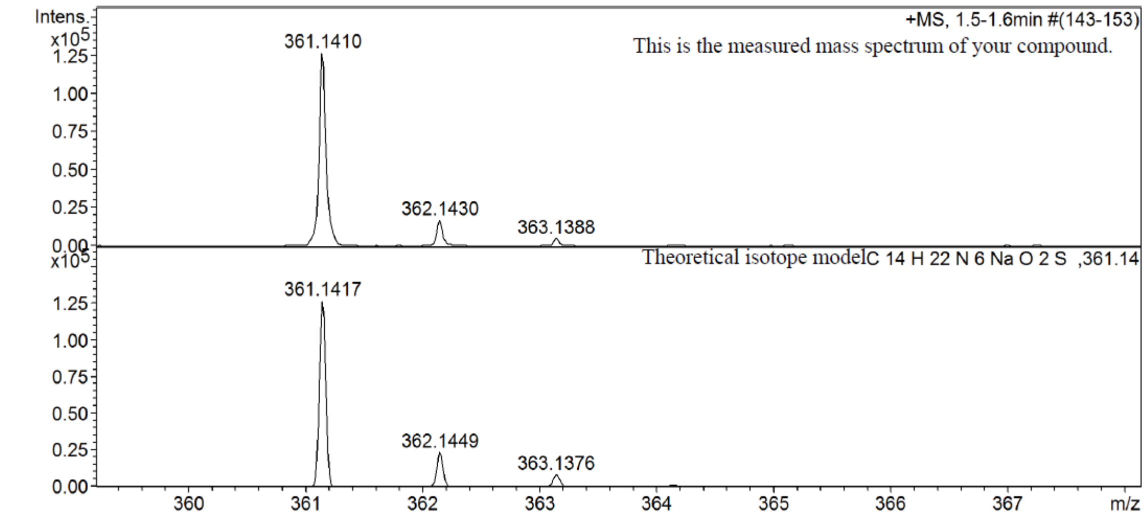

Meas. m/z	#	Formula	m/z	err [ppm]	Mean err [ppm]	rdb	e¯ Conf	mSigma
361.1410								
	1	C 14 H 22 N 6 Na O 2 S	361.1417	1.9	2.1	6.5	even	28.43

Figure S.23. Positive mode ESI-mass spectrometry of precursor **compound II** (denoted Pyr-enBoc) with m/z value and isotopic pattern for the selected compounds formula (b).

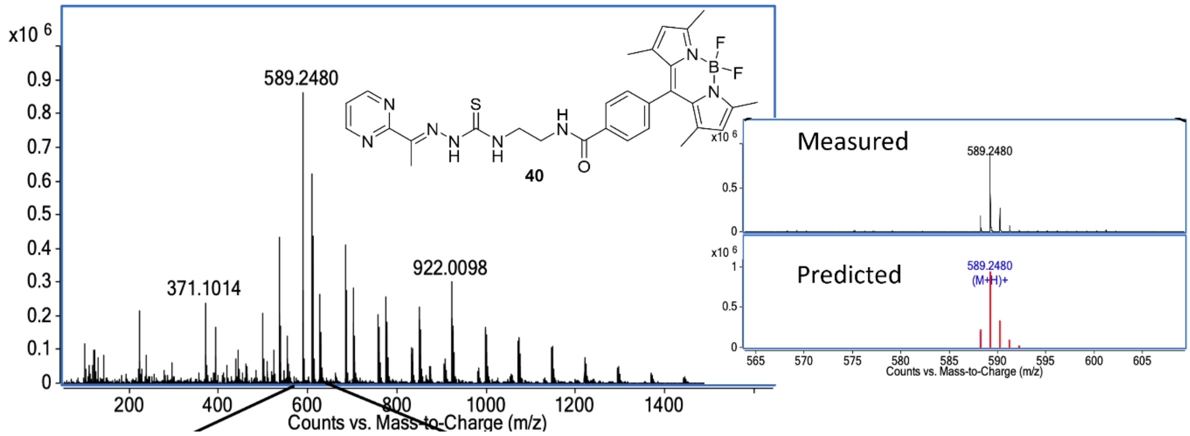

(a)

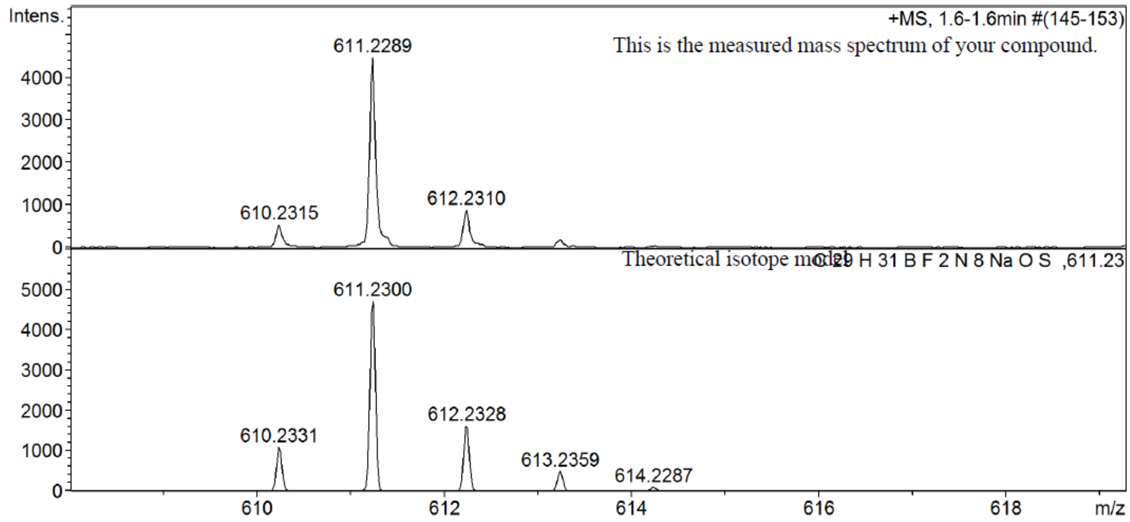

Meas. m/z	#	Formula	m/z	err [ppm]	Mean err [ppm]	rdb	e⁻ Conf	mSigma
611.2289								
	1	C 29 H 31 B F 2 N 8 Na O S	611.2295	1.0	2.0	17.5	even	95.45

Figure S.24. (a) Positive mode ESI-mass spectrometry of compound **HL3**. Insert shows predicted m/z value and isotopic pattern for the selected compounds formula. [The peak at 922.0098 is an internal calibrant]; (b) accurate mass for [M+Na].

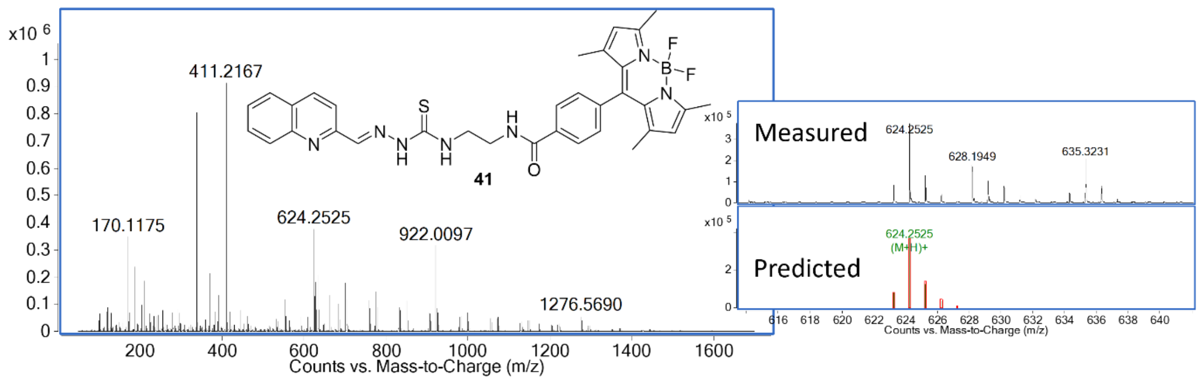

x10 6
411.2167
170.1175
624.2525
922.0097
1276.5690
41
Measured
624.2525
628.1949
635.3231
Predicted
624.2525
(M+H)+
Counts vs. Mass-to-Charge (m/z)

(a)

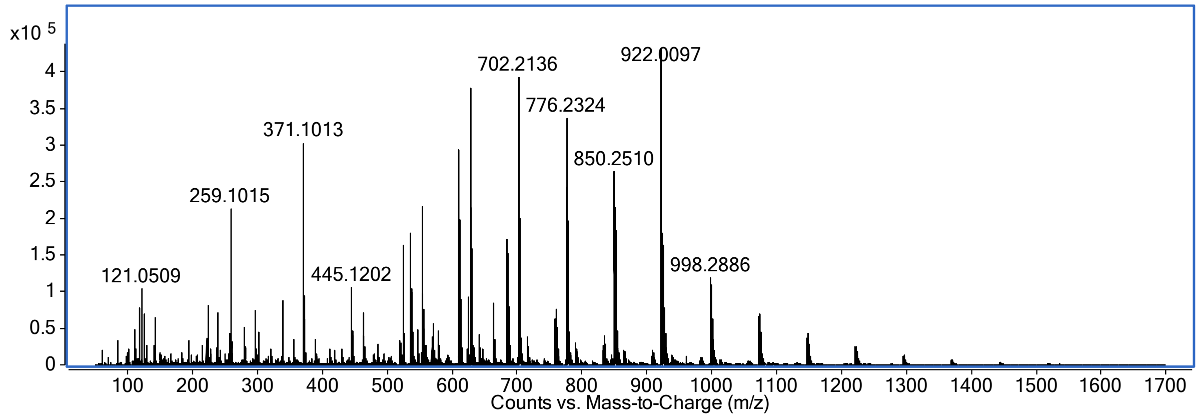

x10 5
702.2136
922.0097
776.2324
371.1013
850.2510
259.1015
121.0509
445.1202
998.2886
Counts vs. Mass-to-Charge (m/z)

(b)

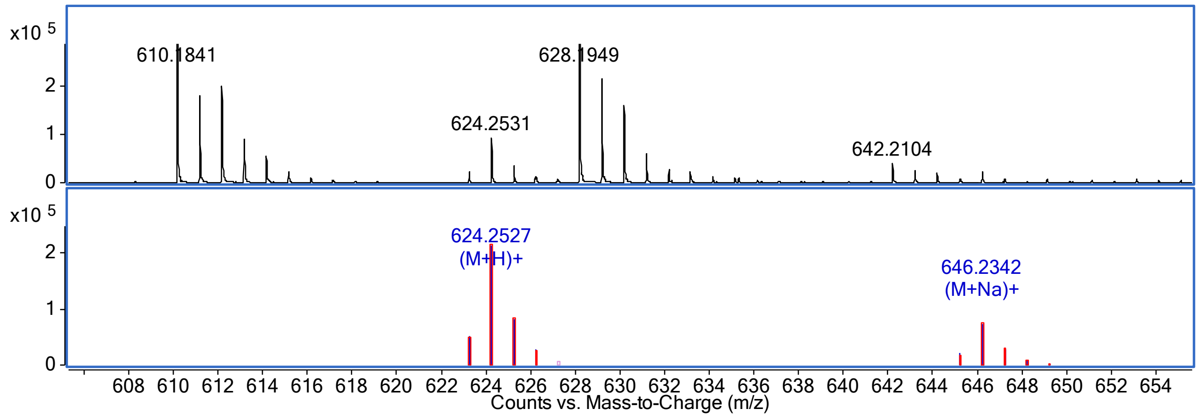

x10 5
610.1841
628.1949
624.2531
642.2104
624.2527
(M+H)+
646.2342
(M+Na)+
Counts vs. Mass-to-Charge (m/z)

(c)

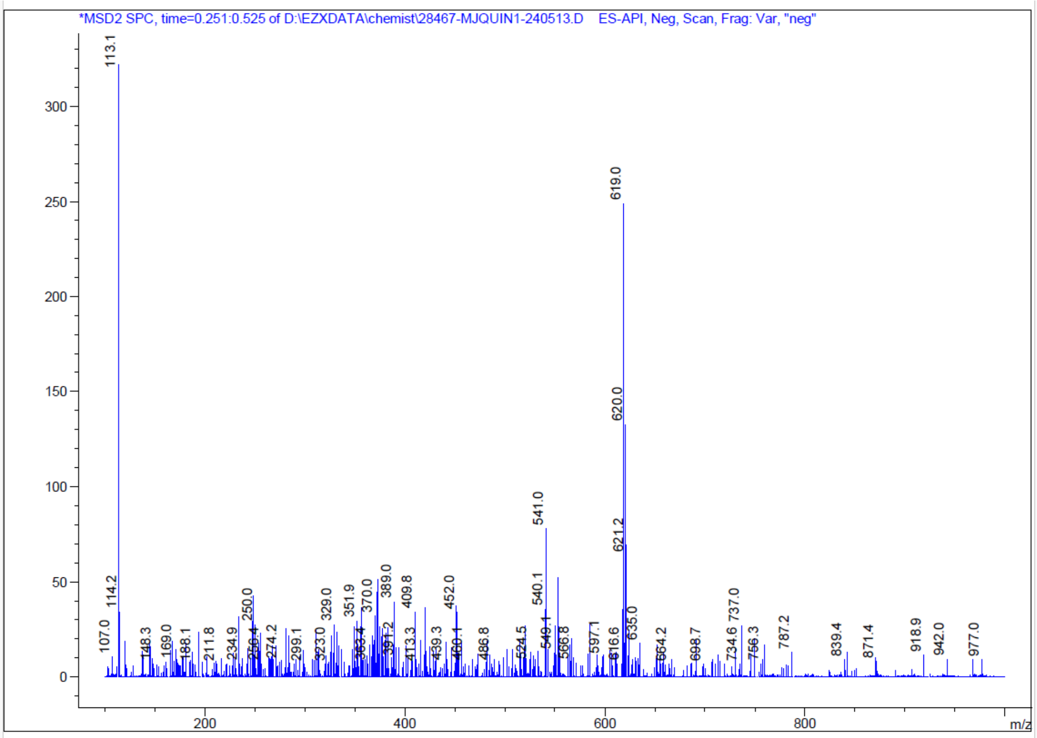

(d)

Figure S.25. Positive mode ESI-mass spectrometry of compound **HL4** (a-b); (c) predicted m/z value and isotopic pattern for the selected compounds formula in HR-MS ESI$^+$(b). mass spectrometry in ES-API mode, negative ionisation, showing main fragment of m/z 620.0 indicative of [M-H$^+$].

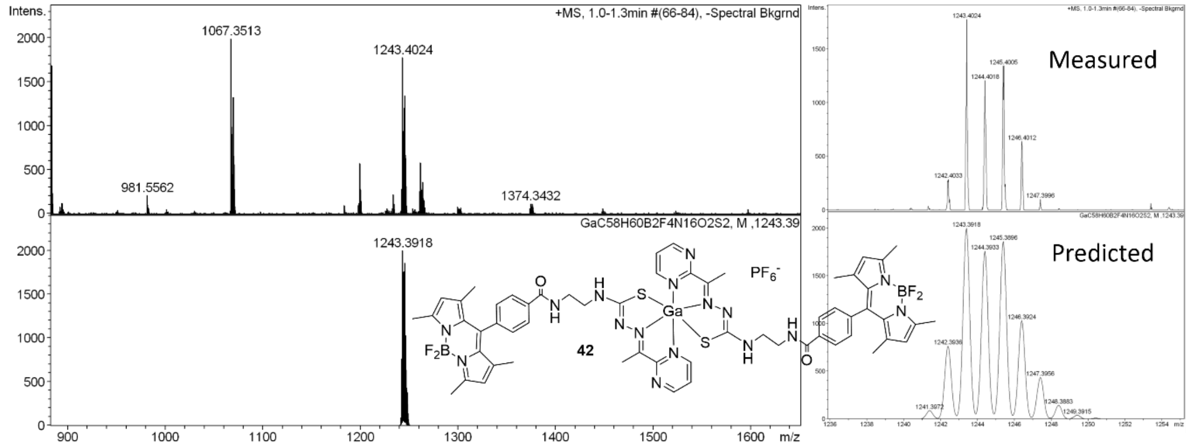

Figure S.25 Positive mode ESI-mass spectrometry of complex **GaL3** with corresponding isotope pattern for the parent ion.

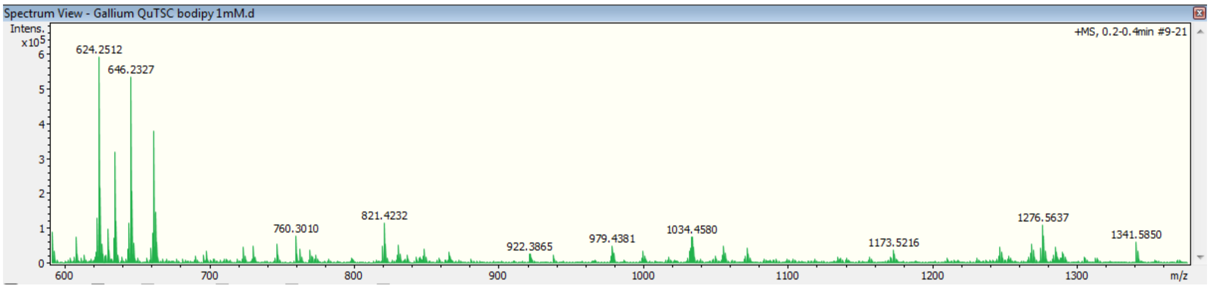

(a)

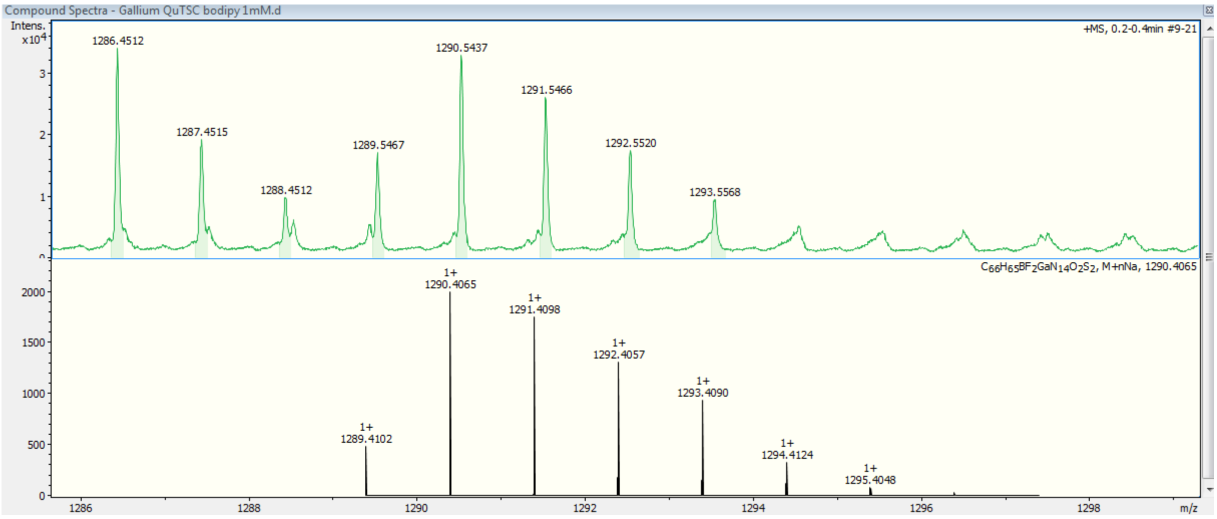

(b)

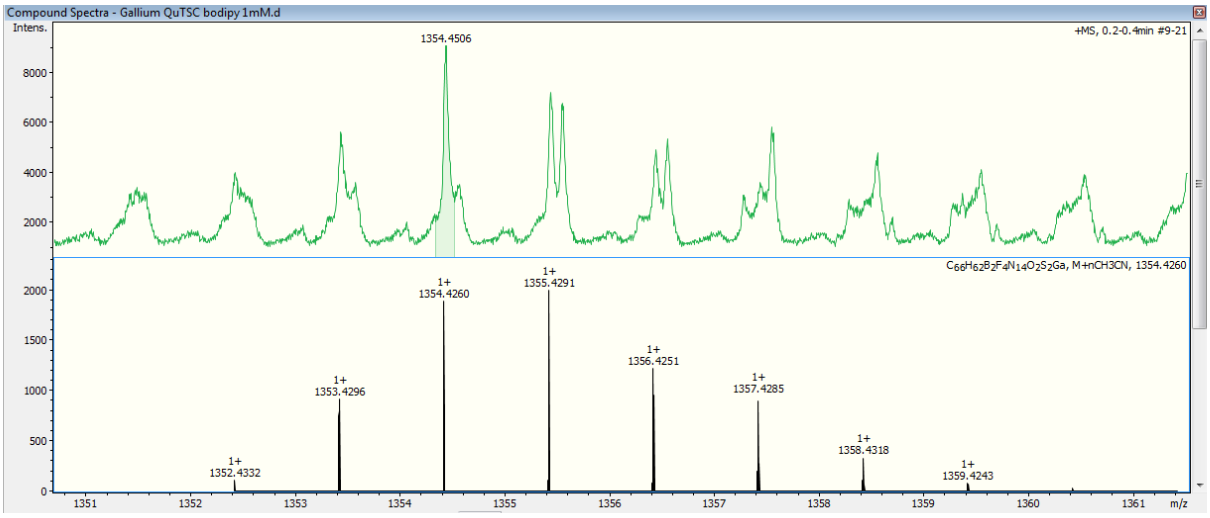
Compound Spectra - Gallium QuTSC bodipy 1mM.d
Intens.
+MS, 0.2-0.4min #9-21
1354.4506
C66H62B2F4N14O2S2Ga, M+nCH3CN, 1354.4260
1+ 1352.4332
1+ 1353.4296
1+ 1354.4260
1+ 1355.4291
1+ 1356.4251
1+ 1357.4285
1+ 1358.4318
1+ 1359.4243
m/z

(c)

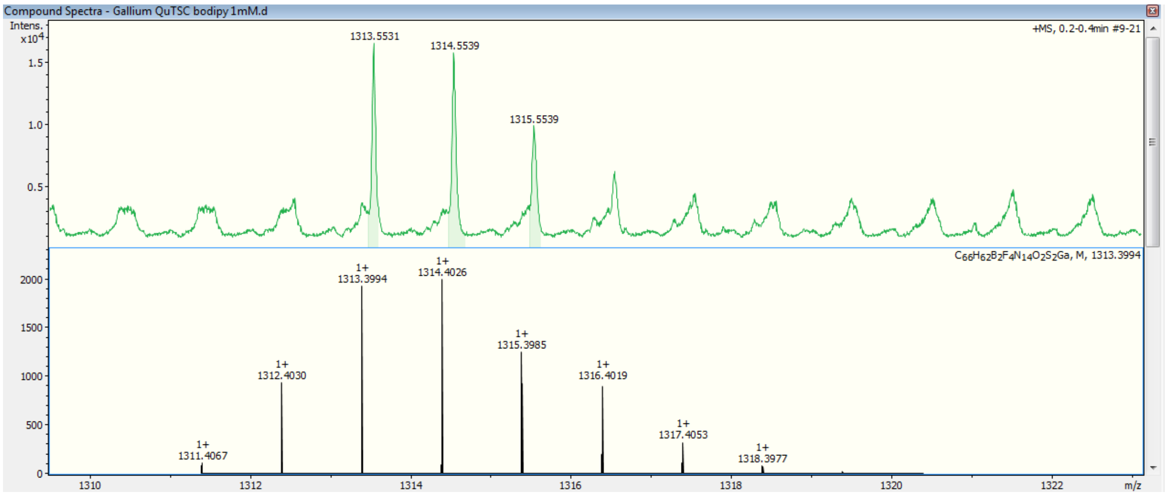
Compound Spectra - Gallium QuTSC bodipy 1mM.d
Intens.
x10^4
+MS, 0.2-0.4min #9-21
1313.5531
1314.5539
1315.5539
C66H62B2F4N14O2S2Ga, M, 1313.3994
1+ 1311.4067
1+ 1312.4030
1+ 1313.3994
1+ 1314.4026
1+ 1315.3985
1+ 1316.4019
1+ 1317.4053
1+ 1318.3977
m/z

(d)

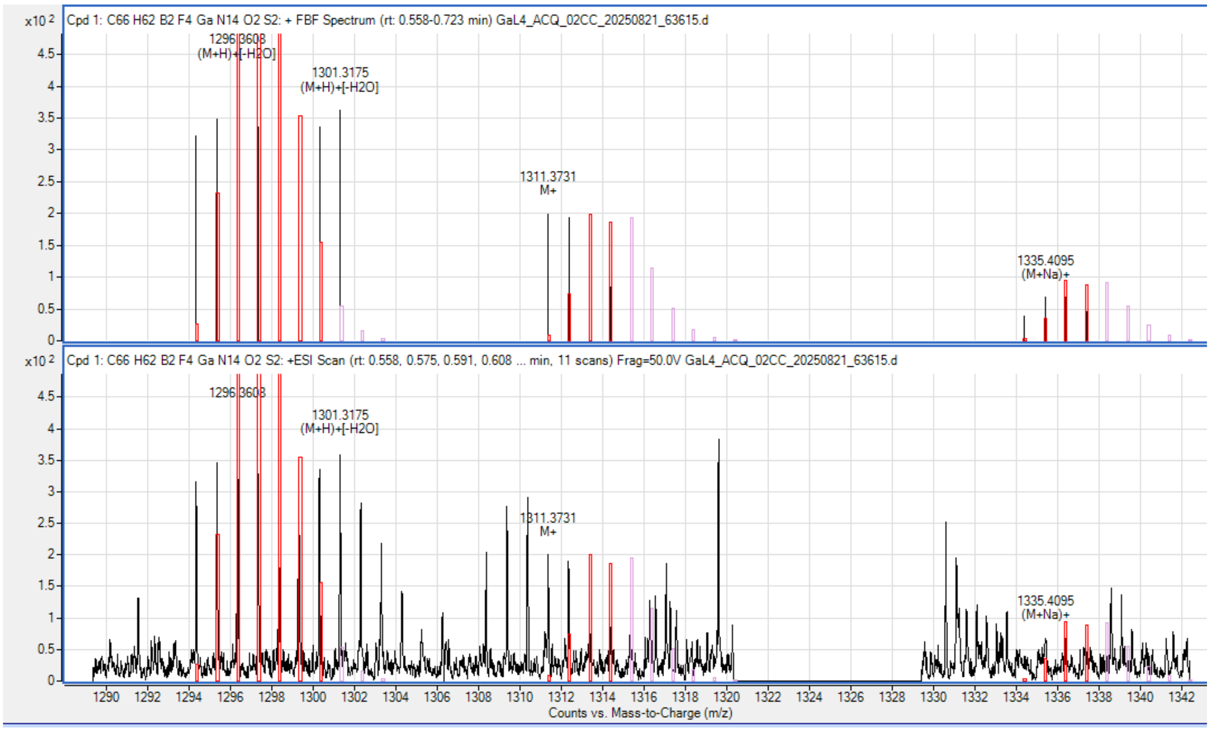

(e)

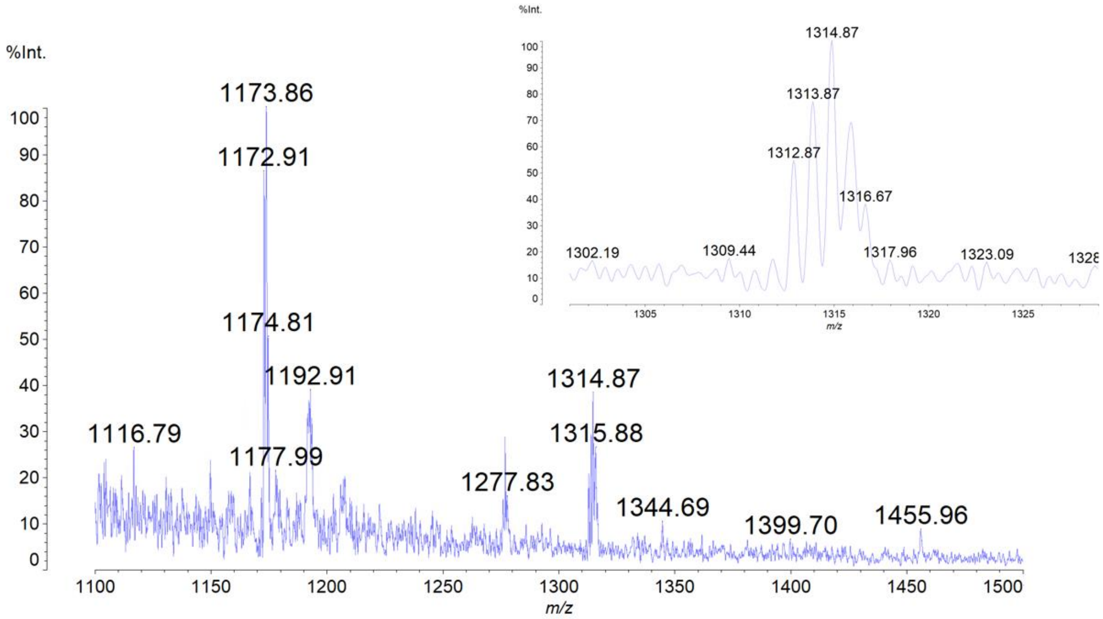

(f)

Figure S.26.(a) Positive mode ESI-mass spectrometry of complex **GaL4**; **(b-e)** Detailed HRMS ESI+ mass spectrometry of **GaL4**; **(c)** extensive fragmentation in mass spectrometry of **GaL4** inclusive of fragments showing [M-BF2+Na] and formation of CH_3CN adductz. **(e)** ESI+ Mass spectrometry fragment indicative of the presence of Ga-OH species in **GaL4**; **(f)** MALDI spectrometry of **GaL4** showing parent ion.

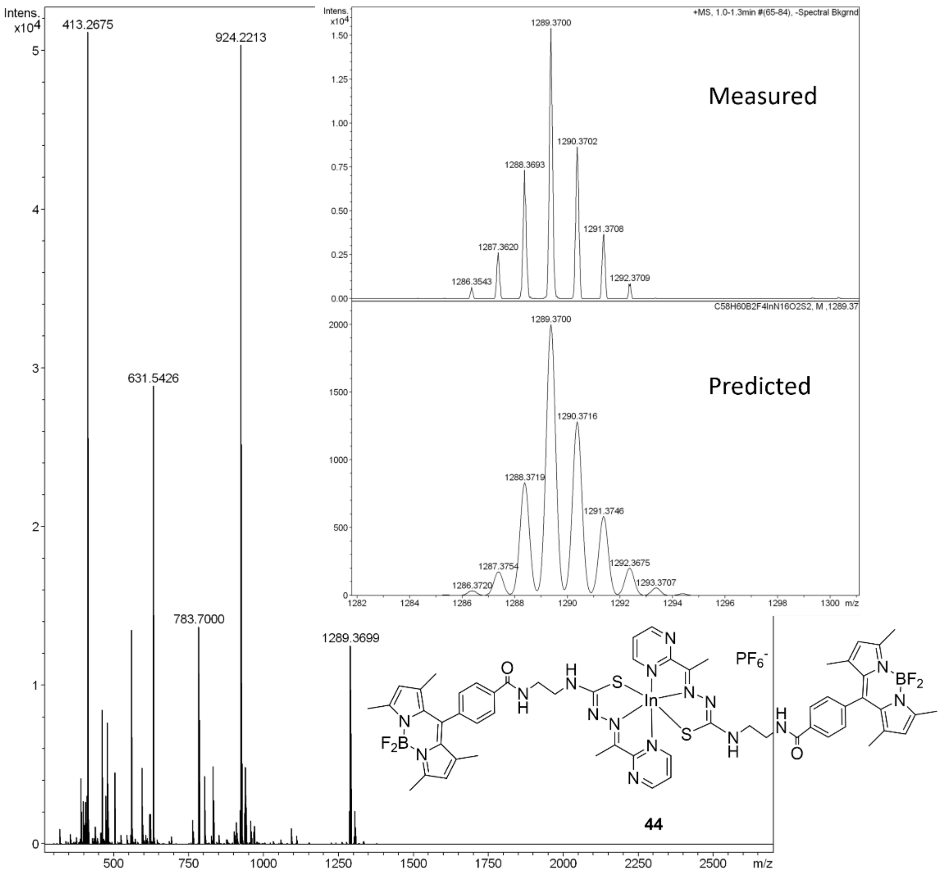

Figure S.27. (a) Positive mode ESI-mass spectrometry of complex **InL3**. Insert shows predicted m/z value and isotopic pattern for the expected M/z for this compound

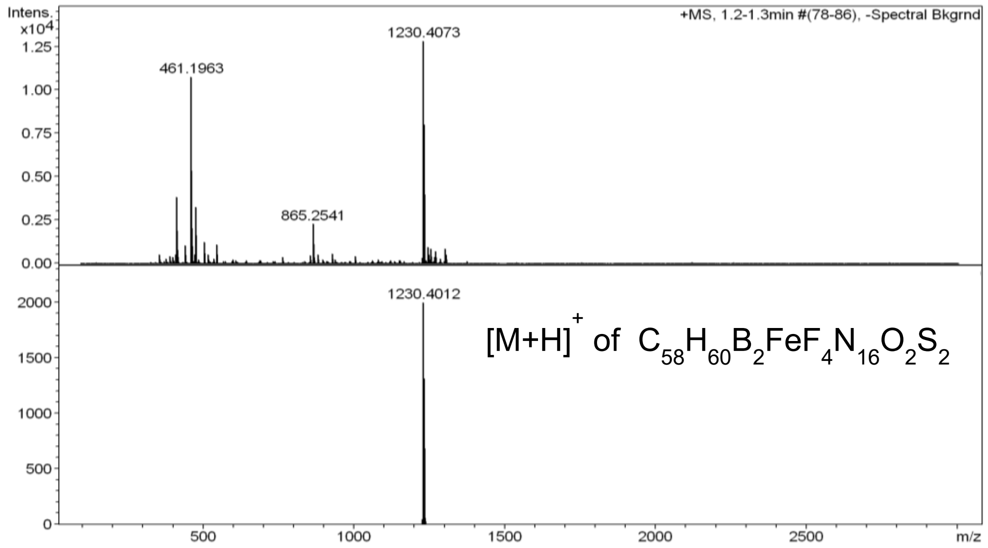

(a)

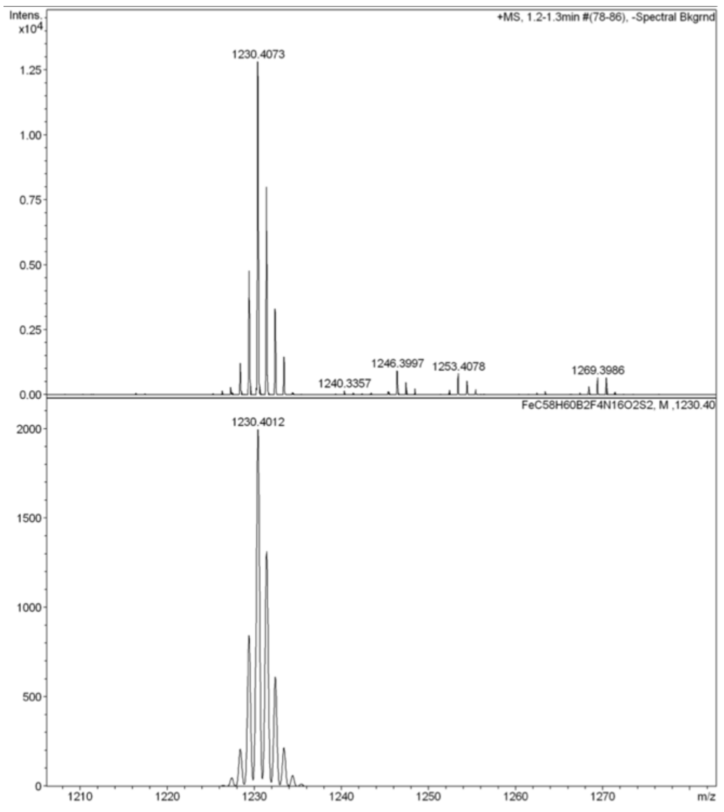

(b)

Figure S.28. Positive mode ESI-mass spectrometry of complex **FeL3 (a**); including the measured vs. predicted isotopic pattern **(b).**

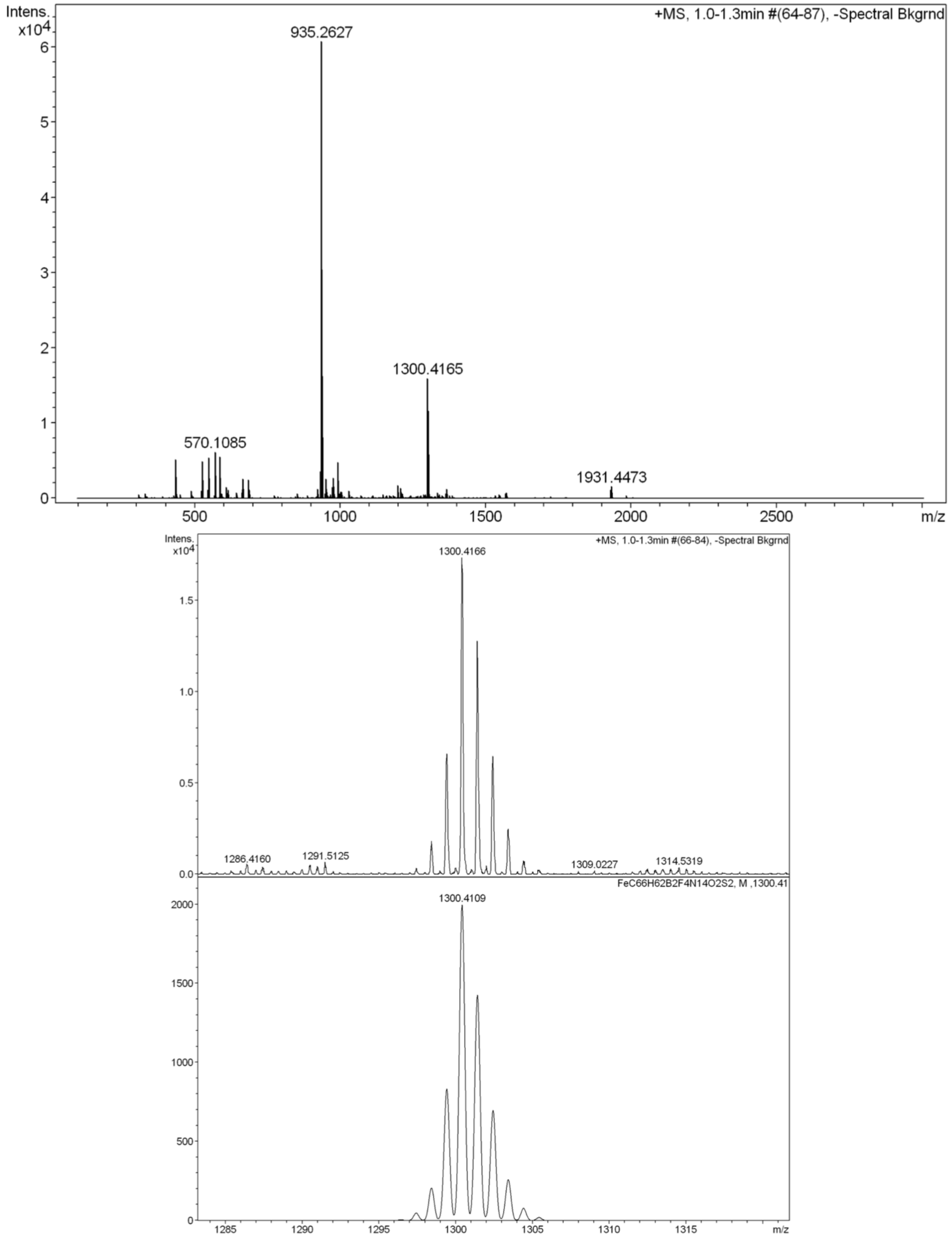

Figure S.29. Positive mode ESI-mass spectrometry of complexes **FeL4 (a)** including the isotopic pattern whereby the peak with 1300.417 m/z represents the $[M+H]^+$ ion for **FeL4**.

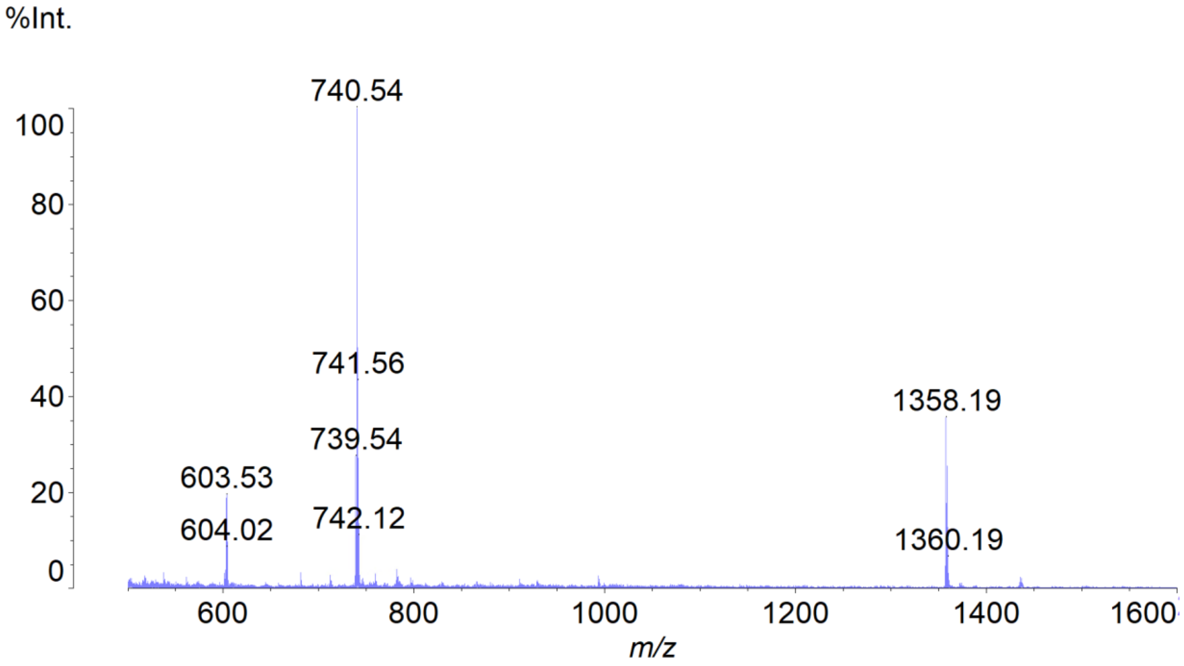
%Int.
740.54
741.56
739.54
742.12
603.53
604.02
1358.19
1360.19
100
80
60
40
20
0
600
800
1000
1200
1400
1600
m/z

(a)

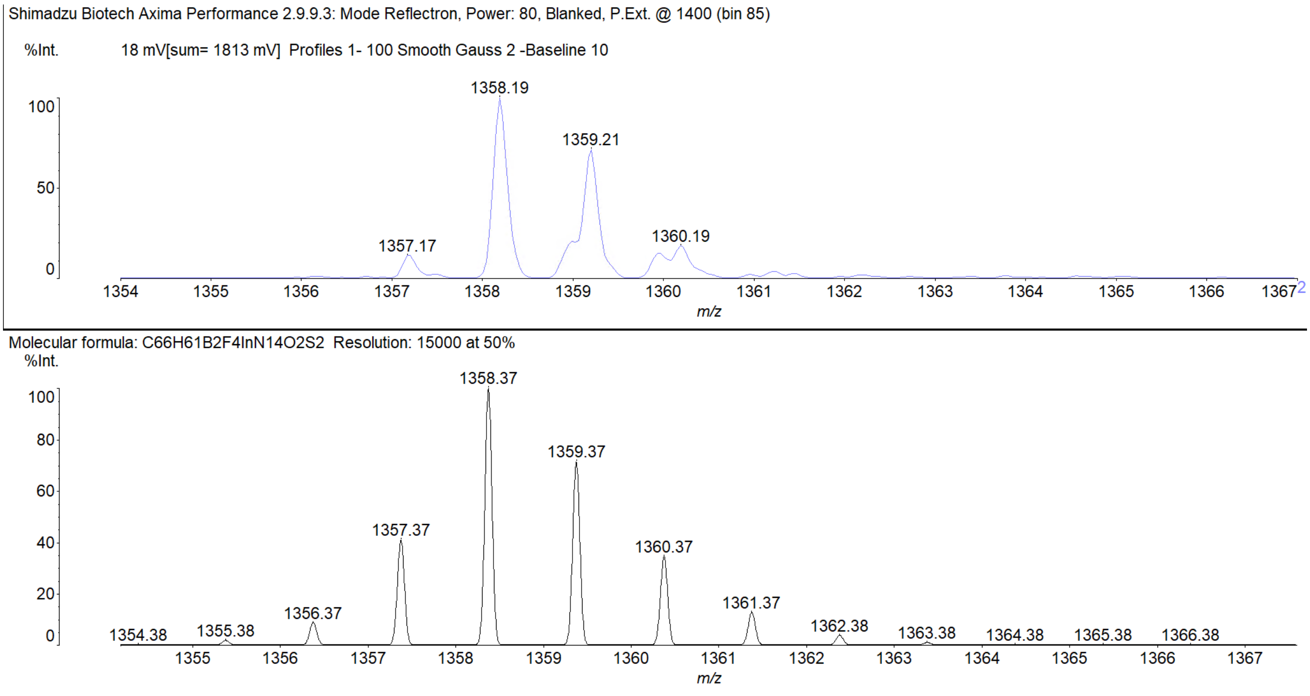
Shimadzu Biotech Axima Performance 2.9.9.3: Mode Reflectron, Power: 80, Blanked, P.Ext. @ 1400 (bin 85)
%Int.
18 mV[sum= 1813 mV] Profiles 1- 100 Smooth Gauss 2 -Baseline 10
1358.19
1359.21
1357.17
1360.19
m/z
Molecular formula: C66H61B2F4InN14O2S2 Resolution: 15000 at 50%
%Int.
1358.37
1359.37
1357.37
1360.37
1356.37
1361.37
1362.38
1354.38
1355.38
1363.38
1364.38
1365.38
1366.38
m/z

(b)

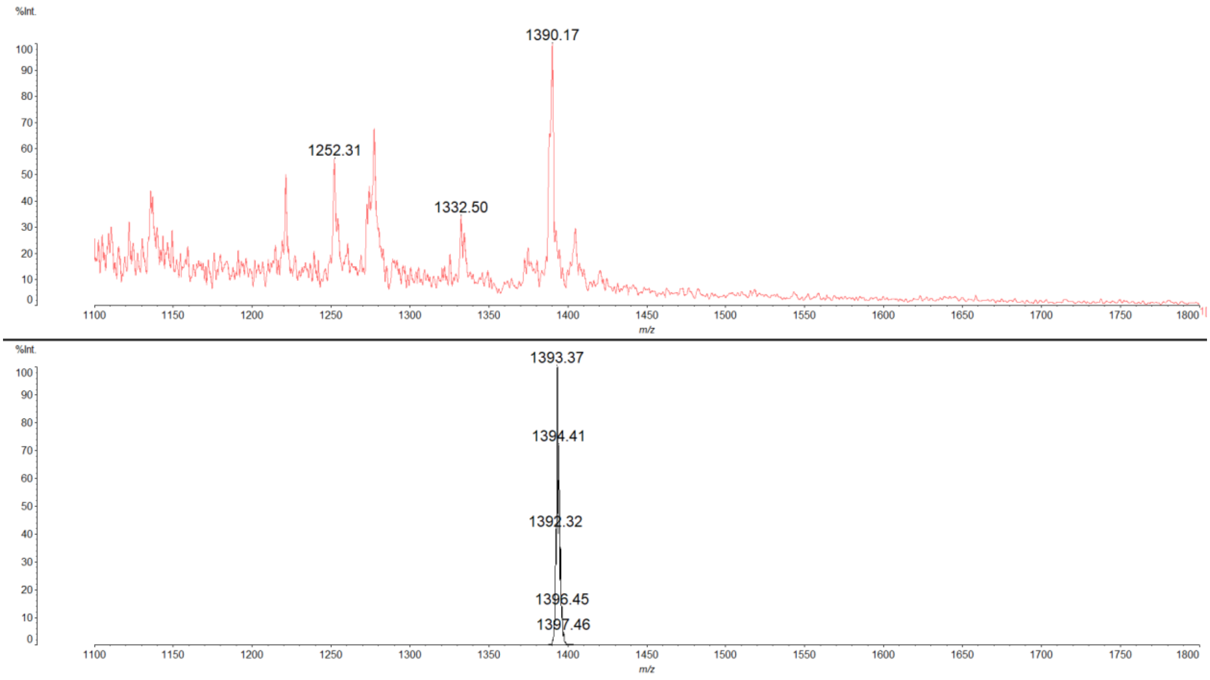

(c)

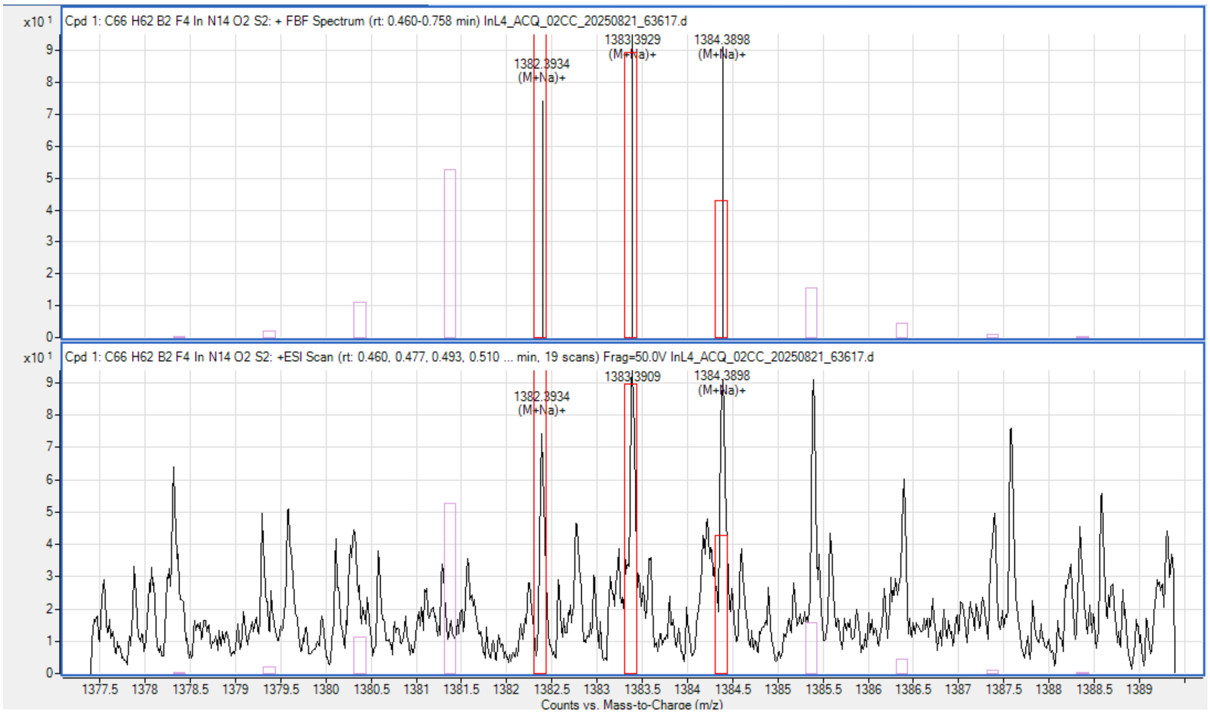

(d)

Figure S.29. (a) MALDI-TOF MS spectrum for **InL4** recorded in reflectron positive ionisation mode. **(b)** MALDI-TOF MS spectrum (top, blue) and theoretical MALDI spectrum (black, bottom) recorded in reflectron positive ionisation mode with isotopic match for theoretical molecular formula m/z of **[M-H]**: $C_{66}H_{61}B_2F_4InN_{14}O_2S_2$ characteristic for **InL4. (d**) HRMS ESI+ mass spectrometry of **InL4**.

6. Selected HPLC Traces

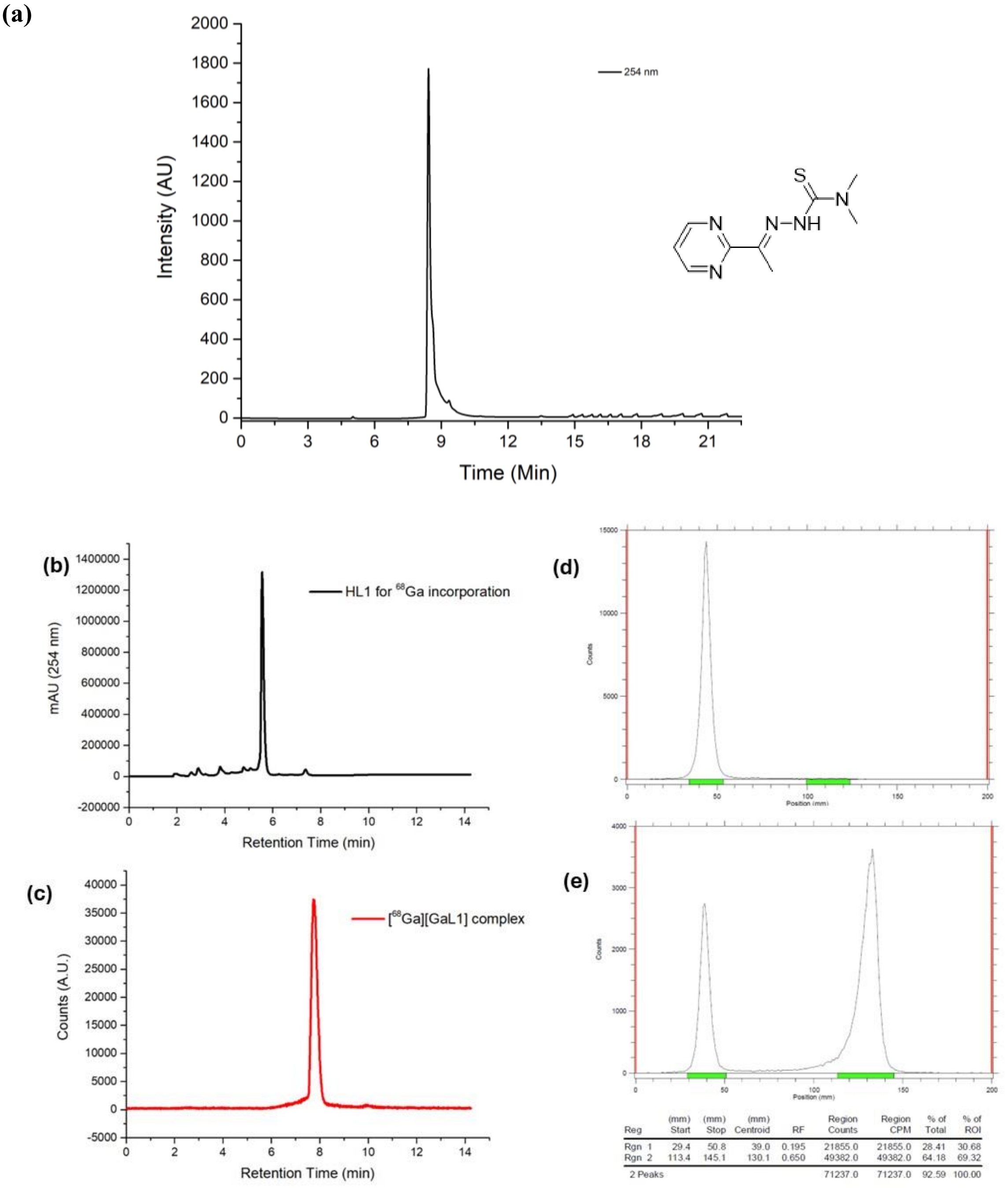

Figure S.30. (a) C-18, reverse phase analytical HPLC trace of **HL1**, 254 nm; Analytical data for the ^{68}Ga Radiolabelling of HL1: (a) UV-Vis detection ((HPLC Method A); (b) radioHPLC trace for [^{68}Ga]**GaL1**, (c) RadioTLC for free aqueous ^{68}Ga and (d) RadioTLC for [^{68}Ga]**GaL1**. Methods were analogues to our previously published protocols, with radio-iTLC carried out on C18 paper TLC, mobile phase: 0.35 M EDTA. [10]

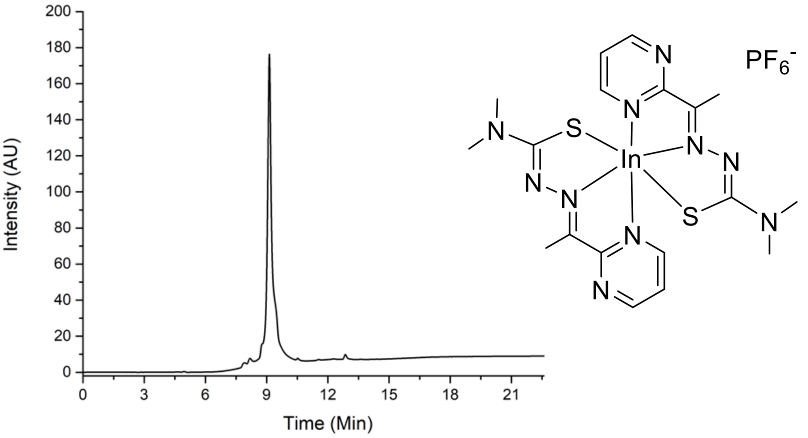

Figure S.31. C-18, reverse phase analytical HPLC trace of **InL1**, 254 nm

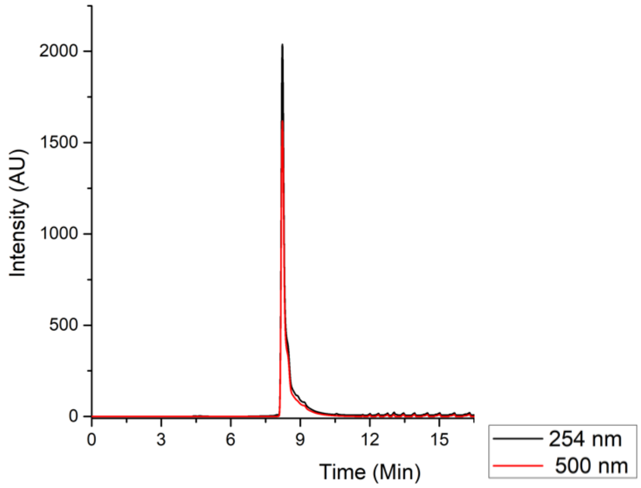

(a)

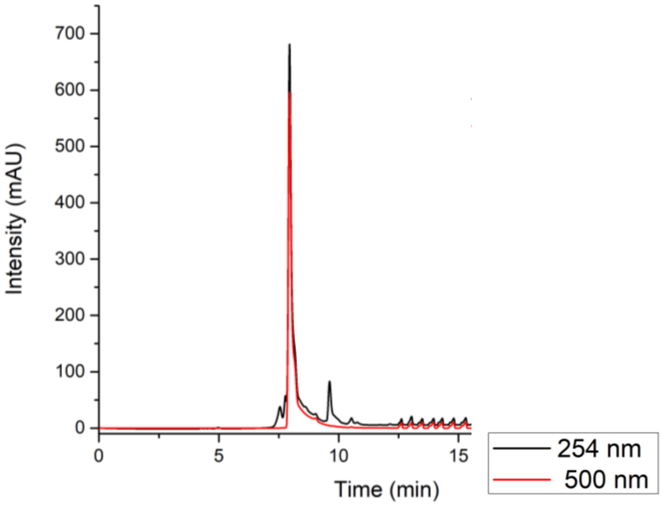

(b)

Figure S.32. C-18, reverse phase analytical HPLC trace of Complex **HL2 (a)** and **GaL2 (b)** at representative wavelengths

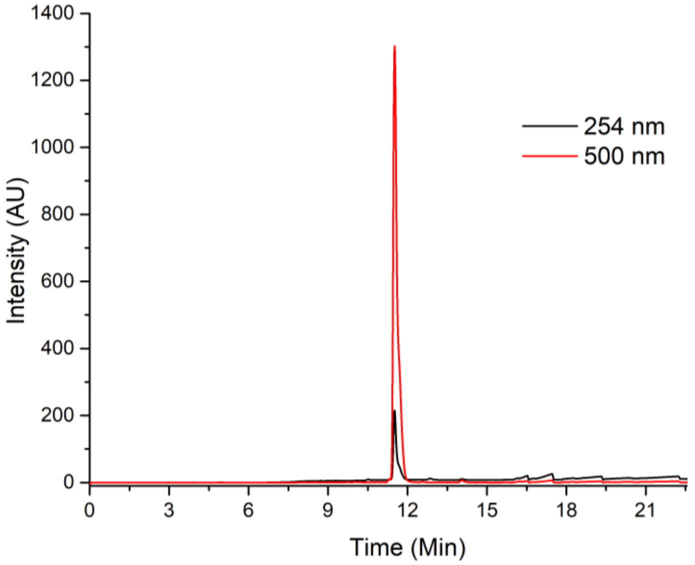

Figure S.33. C-18, reverse phase analytical HPLC trace of BODIPY-COOH at representative wavelengths

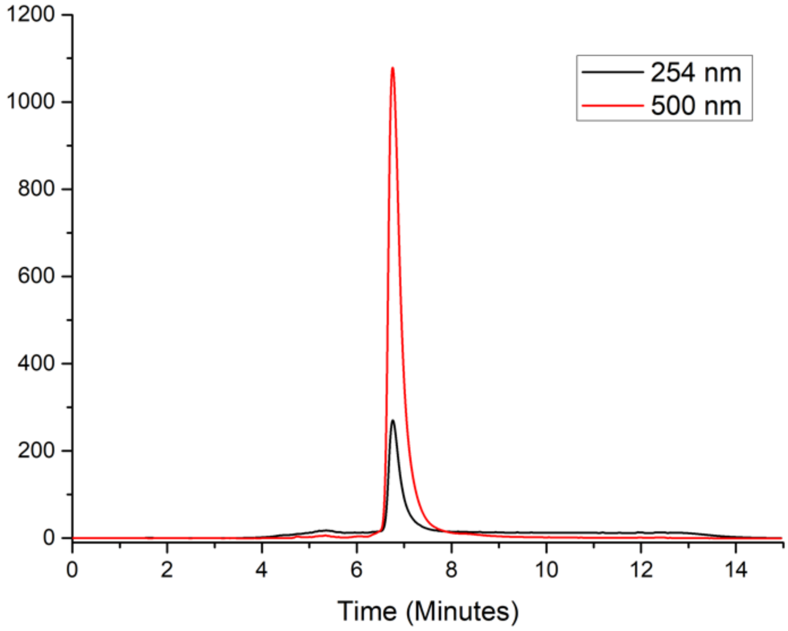

Figure S.34. C-18, reverse phase analytical HPLC trace of BODIPY-NHS at representative wavelengths

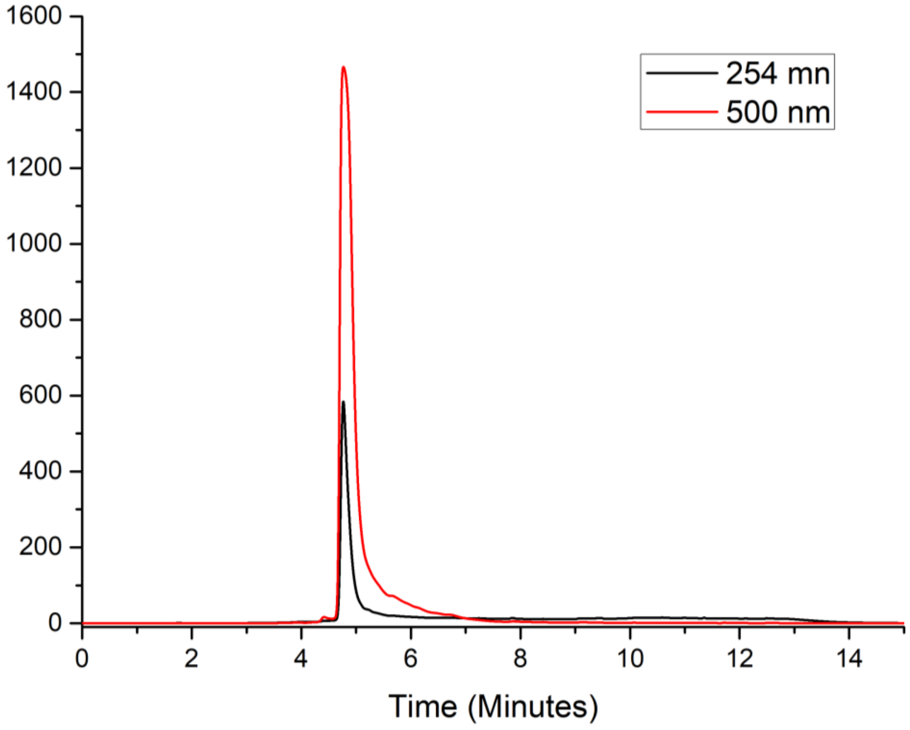

Figure S.35. C-18, reverse phase analytical HPLC trace of BODIPY-En at representative wavelengths

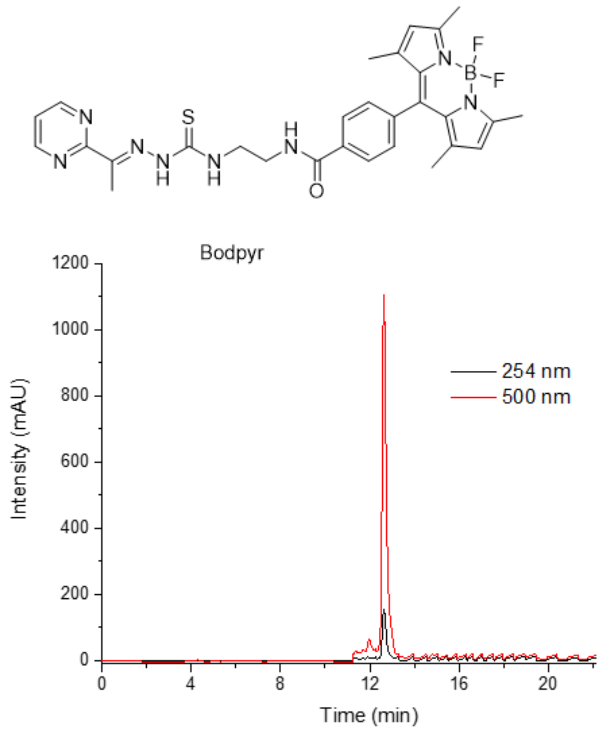

Figure S.36. C-18, reverse phase analytical HPLC trace of **HL3** at representative wavelengths

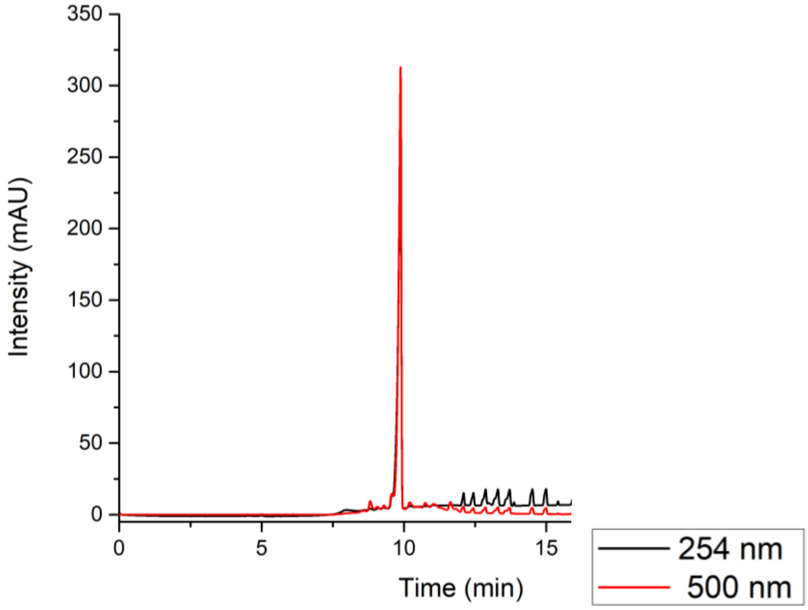

Figure S.37. C-18, reverse phase analytical HPLC trace of complex **GaL3** at representative wavelengths

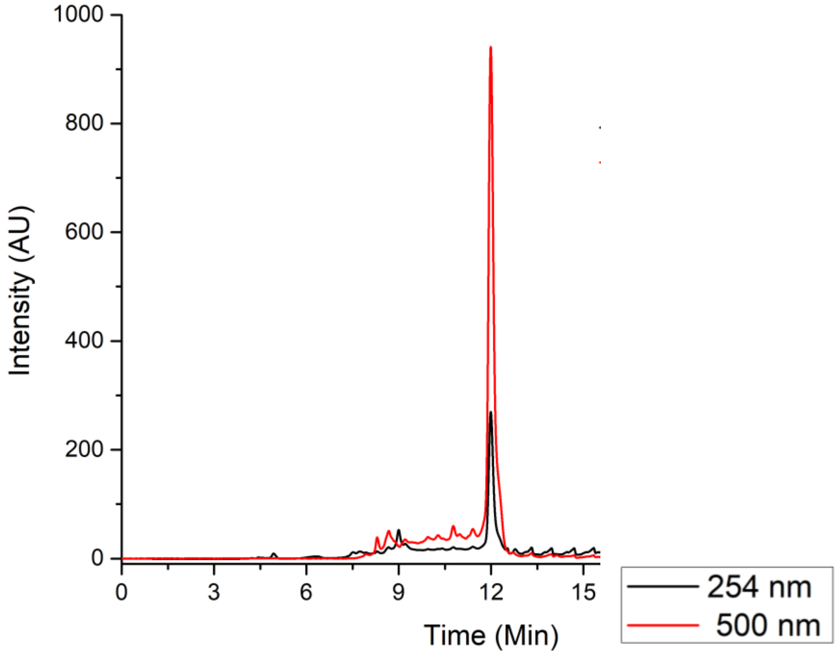

Figure S.38. C-18, reverse phase analytical HPLC trace of complex **InL3** at representative wavelengths

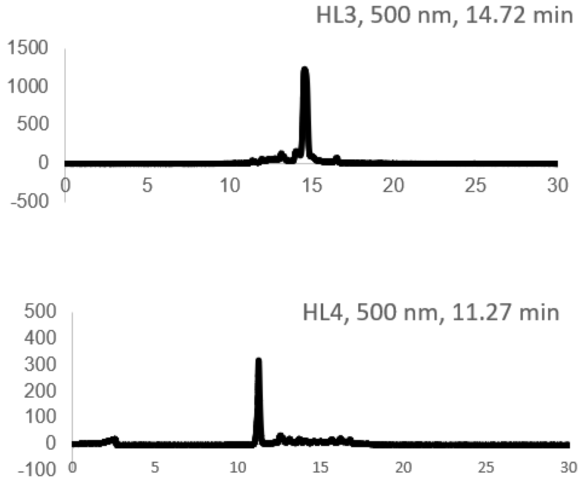

(a)

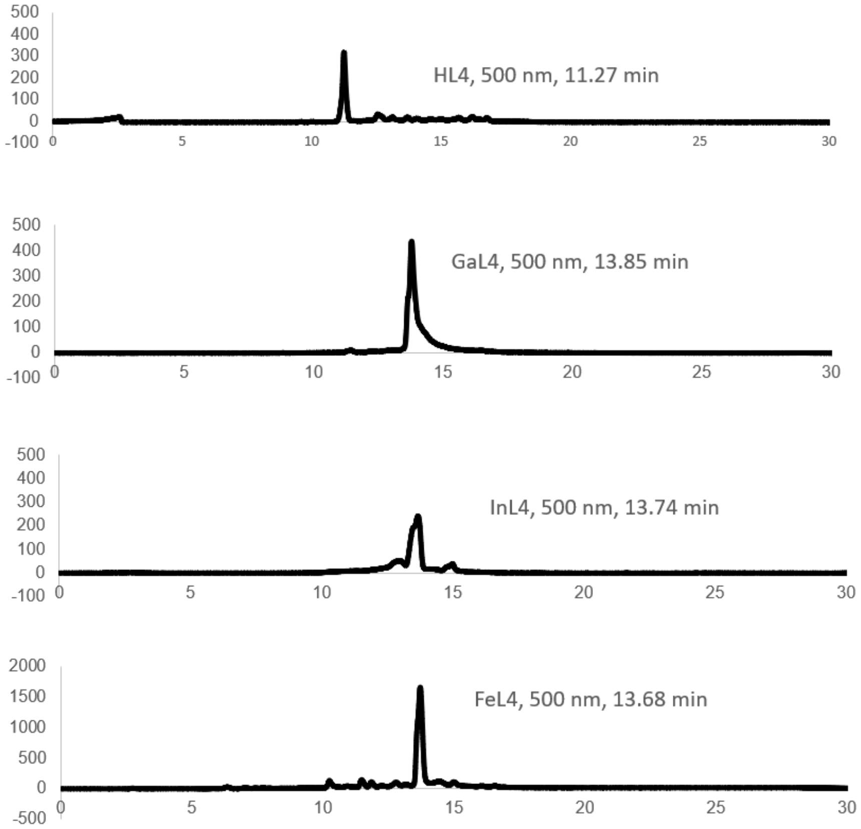

(b)

Figure S39. C18 reverse-phase analytical HPLC traces of BODIPY-tagged species in the HL4 series at representative wavelengths. Conditions: C18 reverse phase; H_2O (0.1% TFA)/CH_3CN (0.1% TFA) Method: The 35-minute gradient method was applied using H_2O/ MeCN each containing 0.1 % TFA as mobile phases with the following conditions: flow rate 1 mL/min, 0 min 5% MeCN; 12 min 95 % MeCN; 26 min 95 % MeCN; 30 min 5 % MeCN, 35 min 5 % MeCN). **(a)** Free ligands **HL3** and **HL4**: spectra recorded within 30 min of dissolution from the solid in fresh DMSO/CH_3CN (1:9). **(b)** A comparison of the HPLC for **HL4** and corresponding complex series: samples recorded within 30 min of dissolution from the solid in fresh DMSO/CH_3CN (1:9).

7. Selected UV-Vis and Fluorescence Emission Data

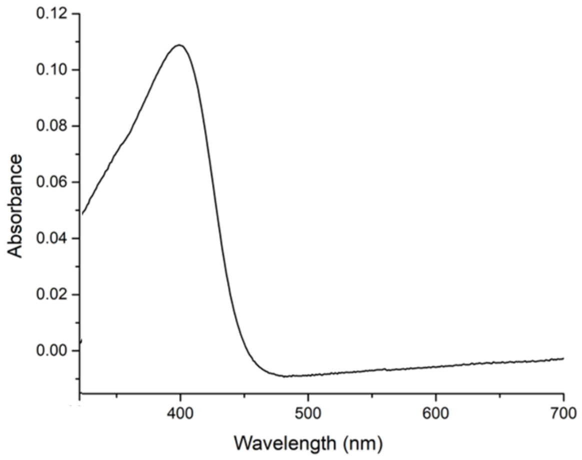

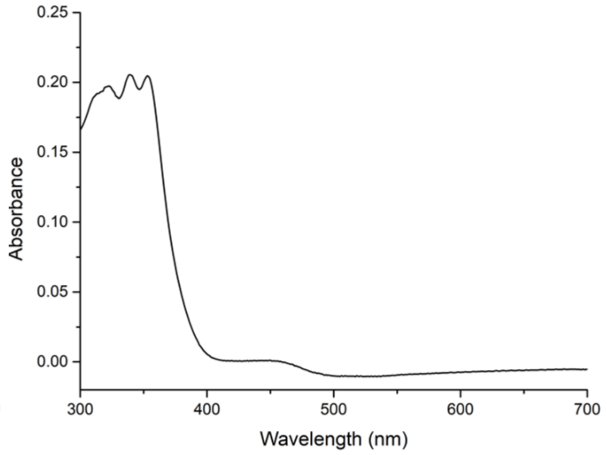

(a) **(b)**

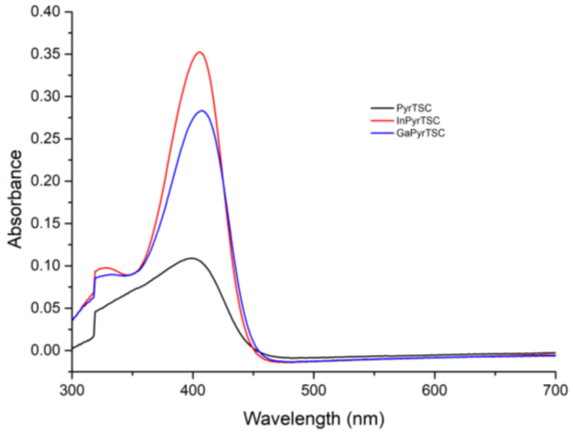

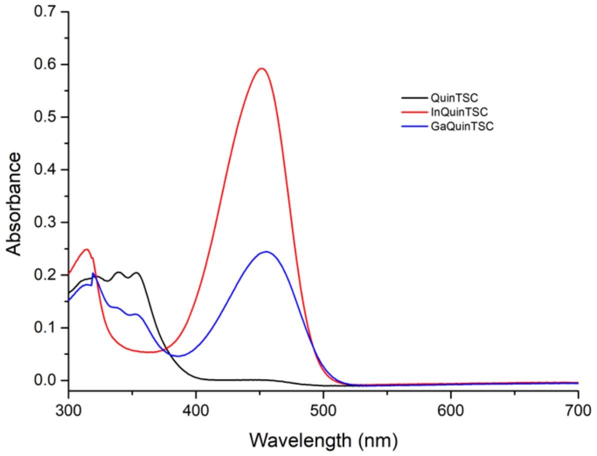

(c) **(d)**

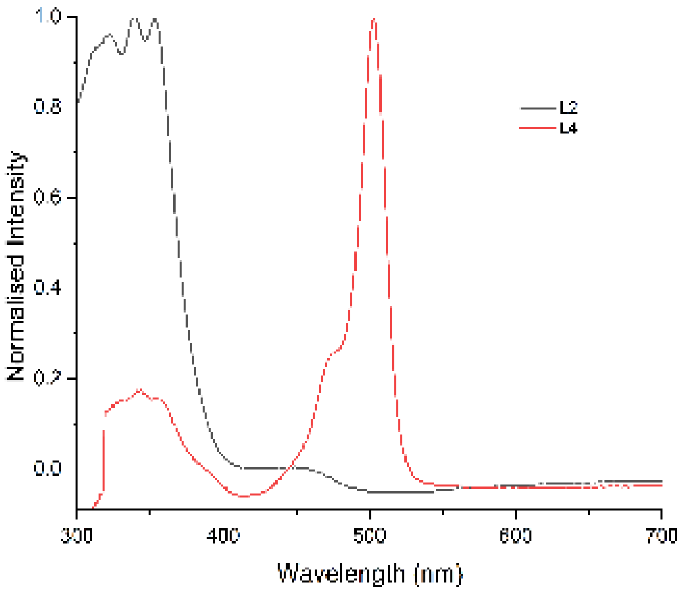

(e)

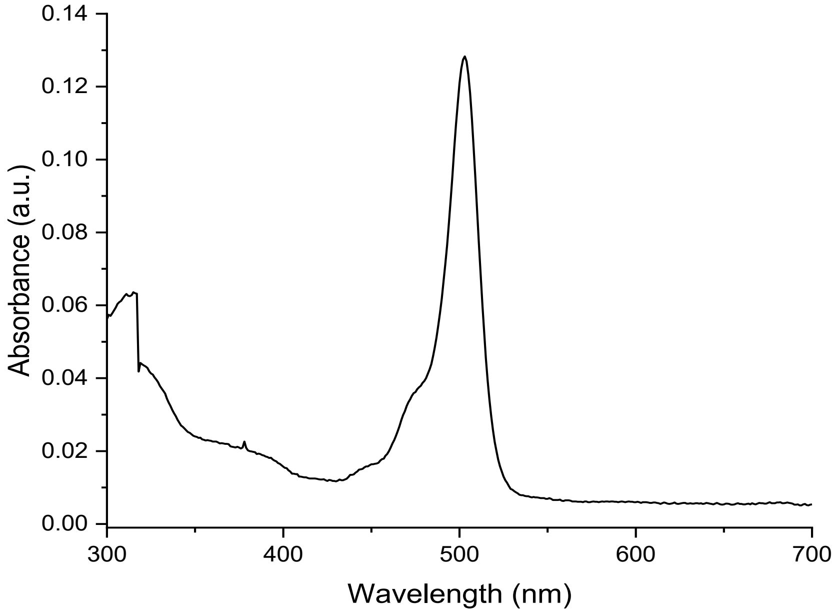

(f)

Figure S.40. (a) UV-Vis spectroscopy of **HL1,** recorded at 10 µM in DMSO; **(b)** UV-Vis spectroscopy of **HL2** recorded at 10 µM in DMSO; **(c)** UV-Vis spectroscopy of **HL1**, **GaL1** and **InL1** (PymTSC series, 10 µM in DMSO), **(d)** UV-Vis spectroscopy of **HL2**, **GaL2** and **InL2** (QuinTSC series 10 µM in DMSO), **(e)** Normalised UV-Vis spectra for **HL2** *vs* **HL4**, where **HL2** data was recorded at a concentration of 10 µM and **HL4** at 500 nM in DMSO; **(f)** UV-Vis spectra for **FeL3** recorded at 1 µM in DMSO.

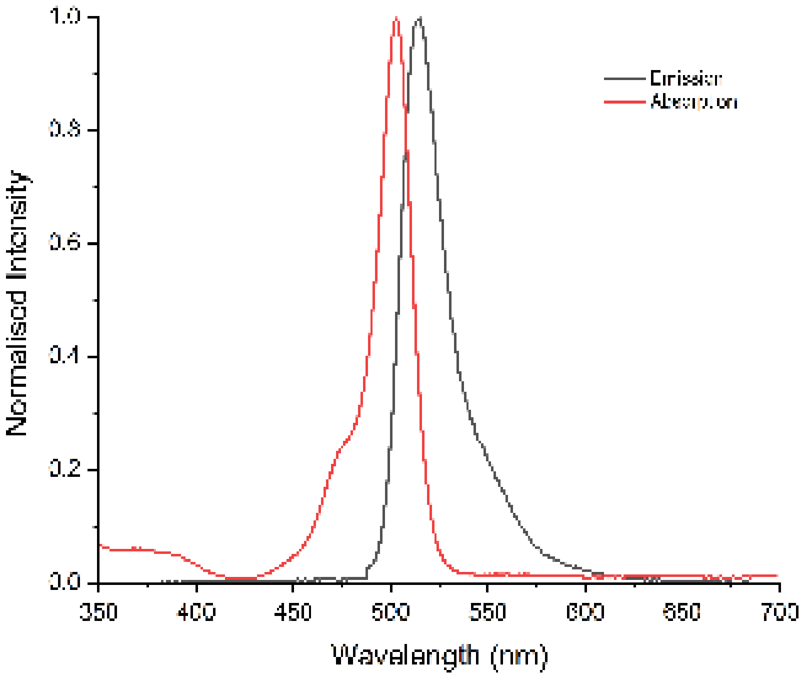

(a)

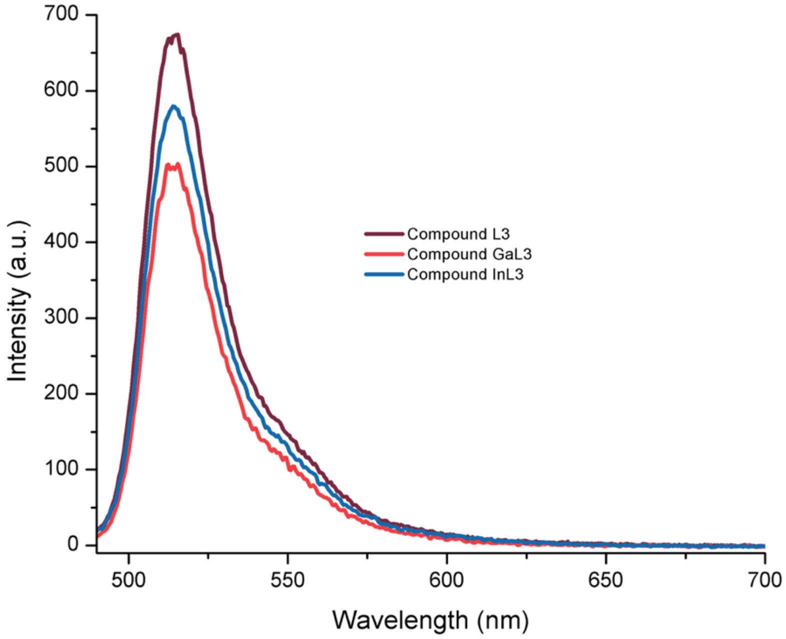

(b)

Figure S.41. (a) Normalised excitation and emission spectra for **HL4** recorded at a concentration of 500 nM in DMSO.**(b)** Overlay of emisson spectra of **HL3, GaL3** and **InL3** recorded at a concentration of 500 nM in DMSO, 480 nm excitation.

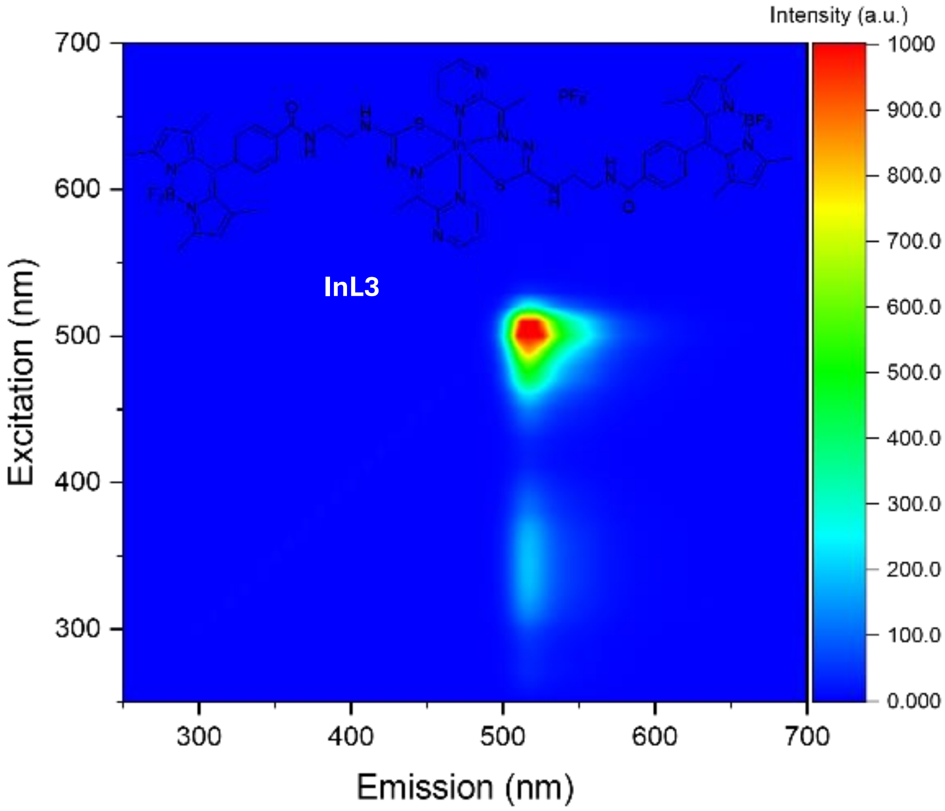

Figure S.42. Excitation-emission profile of **InL3** at a concentration of 750 nM in DMSO.

8. Spectroscopic data for selected kinetic stability assays in biocompatible media

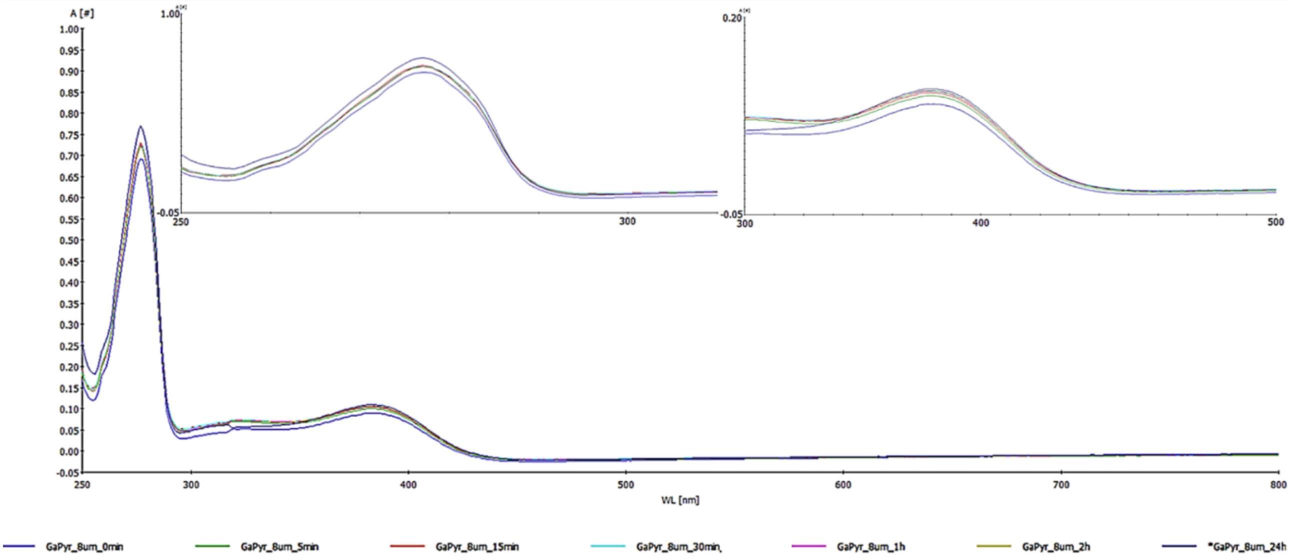

(a) Compound **GaL1** in DMSO: RMPI 1:9

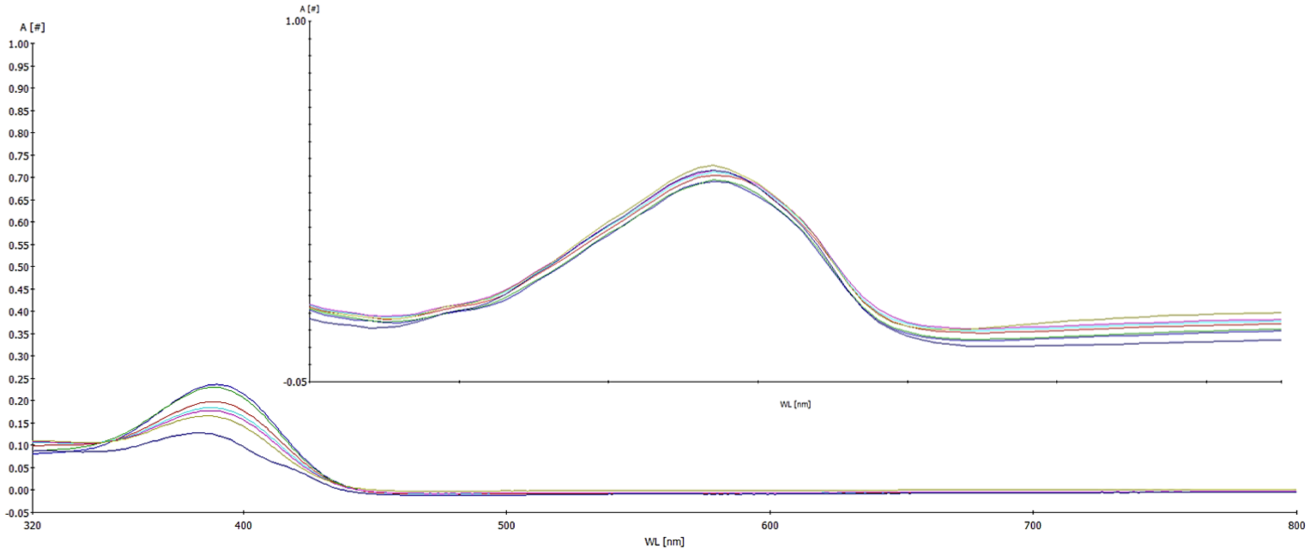

(b) Compound InL1 in DMSO: RMPI 1:9

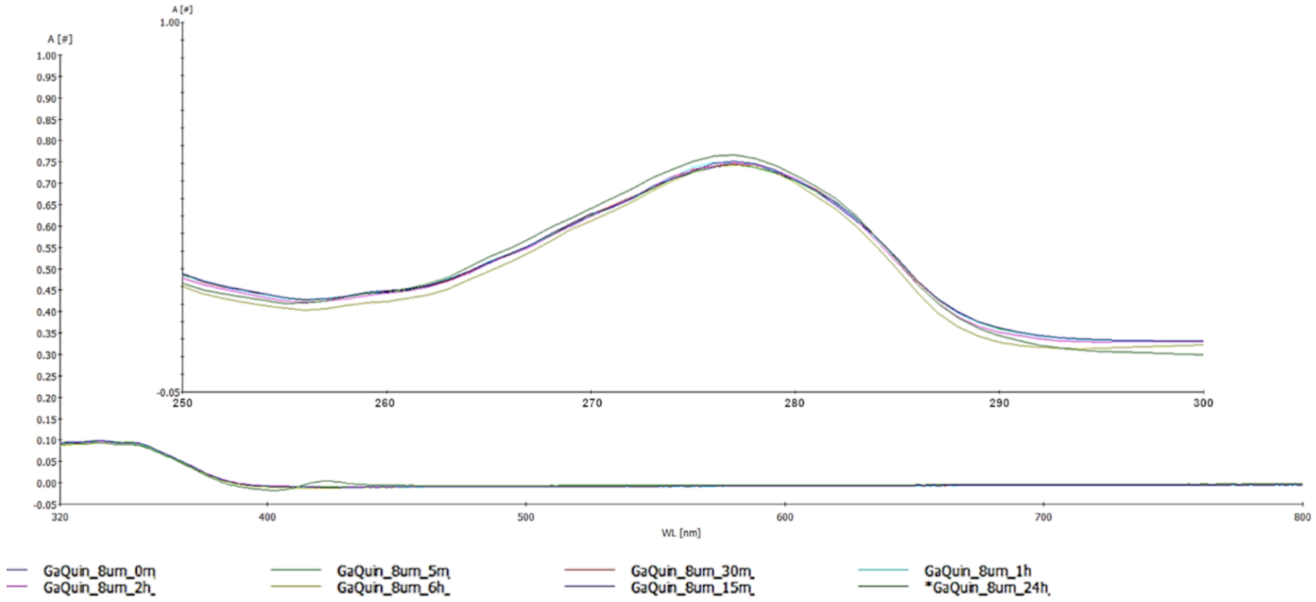

(c) Compound GaL2 in DMSO: RMPI 1:9

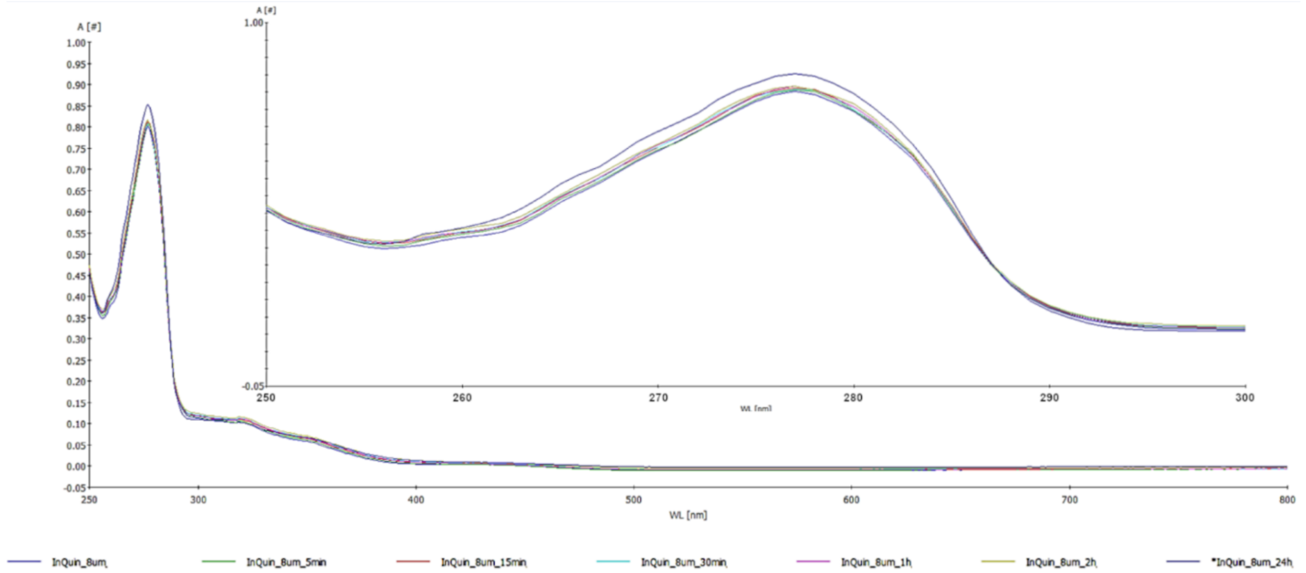

(d) Compound InL2 in DMSO: RMPI 1:9

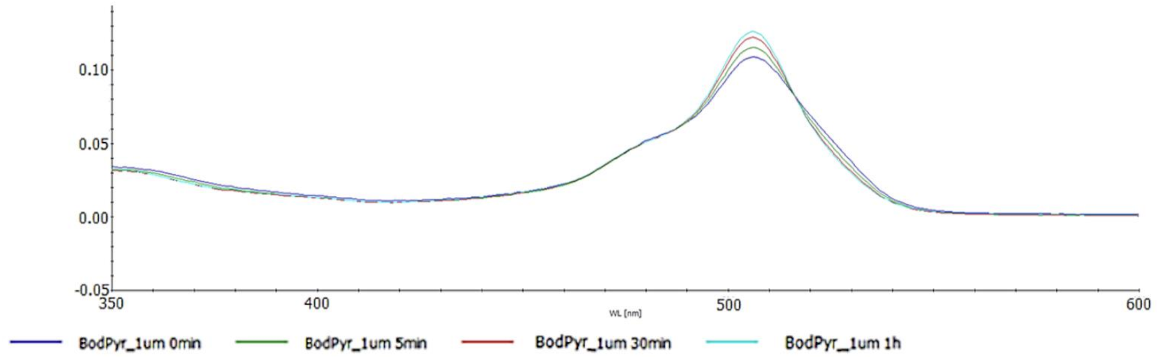

(e) Compound HL3 in DMSO: RMPI 1:9

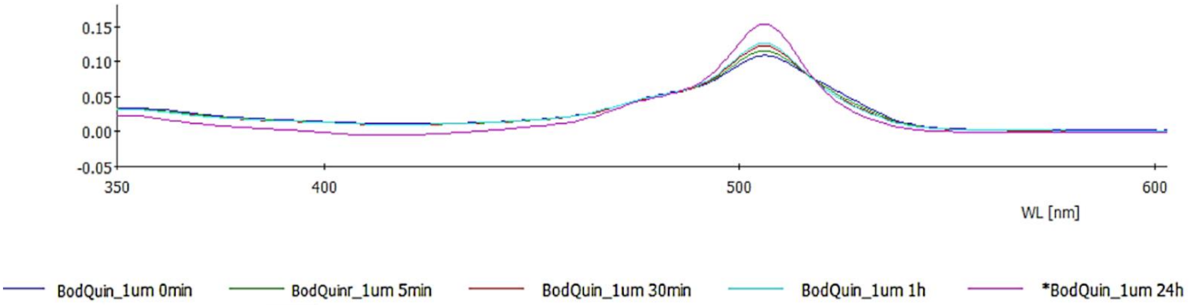

(f) Compound HL4 in DMSO: RMPI 1:9

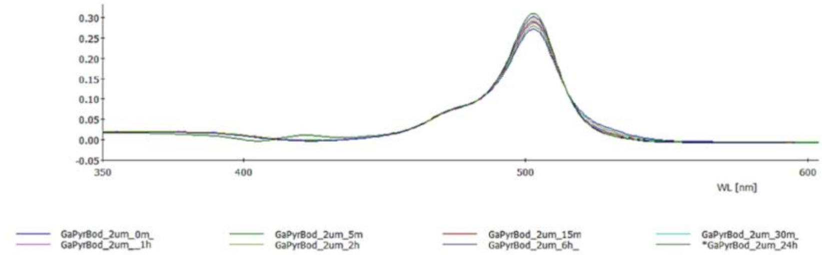

(g) Compound GaL3 in DMSO: RMPI 1:9

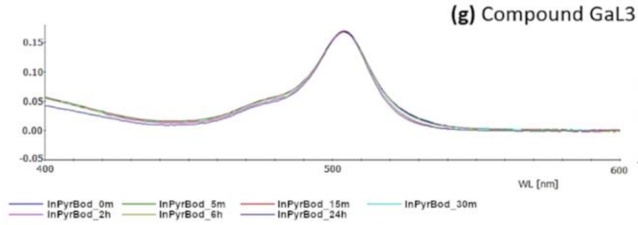

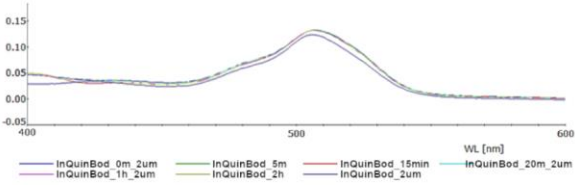

(h) Compound InL3 in DMSO: RMPI 1:9

(i) Compound InL4 in DMSO: RMPI 1:9

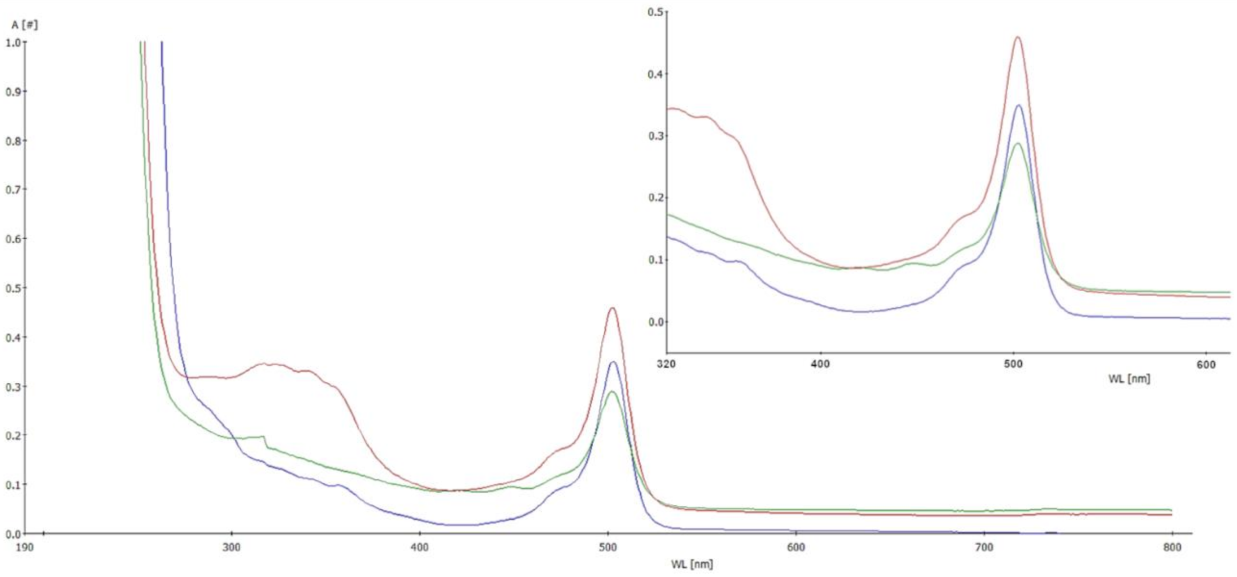

(j) UV-Vis in DMSO: PBS 30%:70%, 30 min, 1 µM conc.

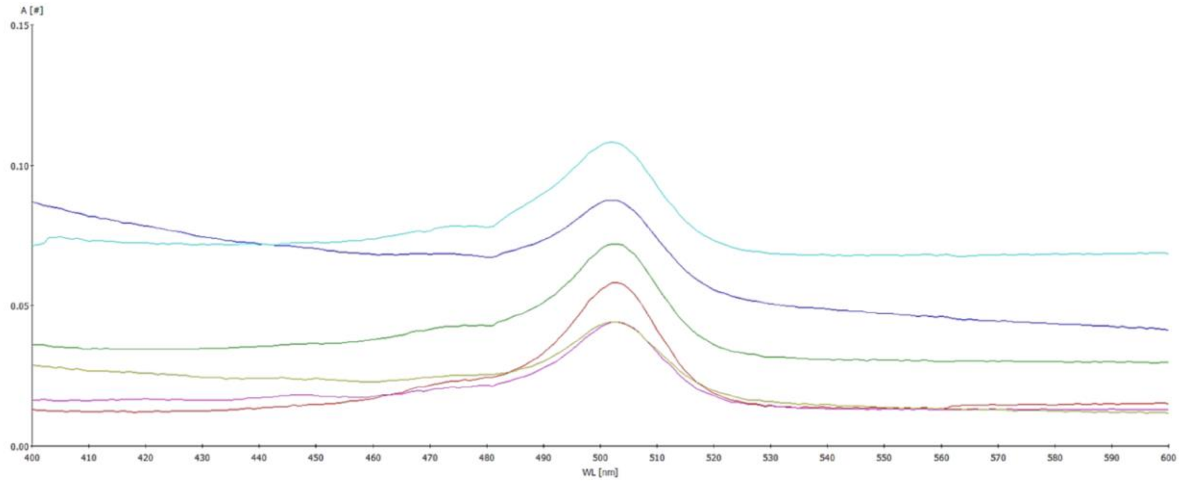

(k) UV-Vis in DMSO *vs.* DMSO: PBS 50%:50%, 500 nM, 30 min incubation, r.t.

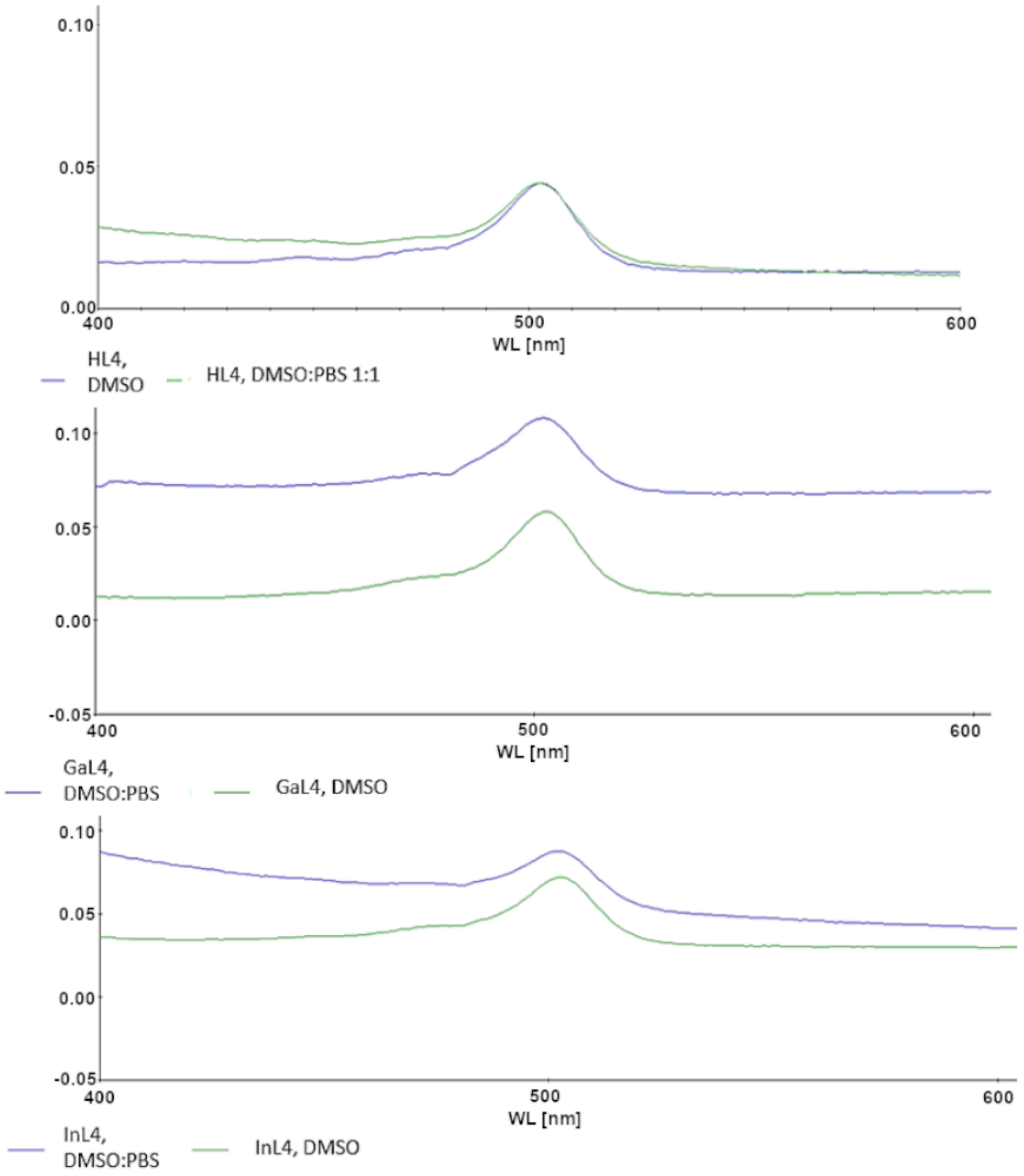

(l): Kinetic stability test 500 nM 1:1 DMSO PBS (pH=7.4)

Figure S.43. (a)-(l): Extended UV-Vis spectroscopy assays of **HL1-HL4** and corresponding Ga(III) and In(III) complexes. Measurements were recorded in solutions of 500 nM in DMSO:RMPI (1:99) or DMSO:PBS 1:1.

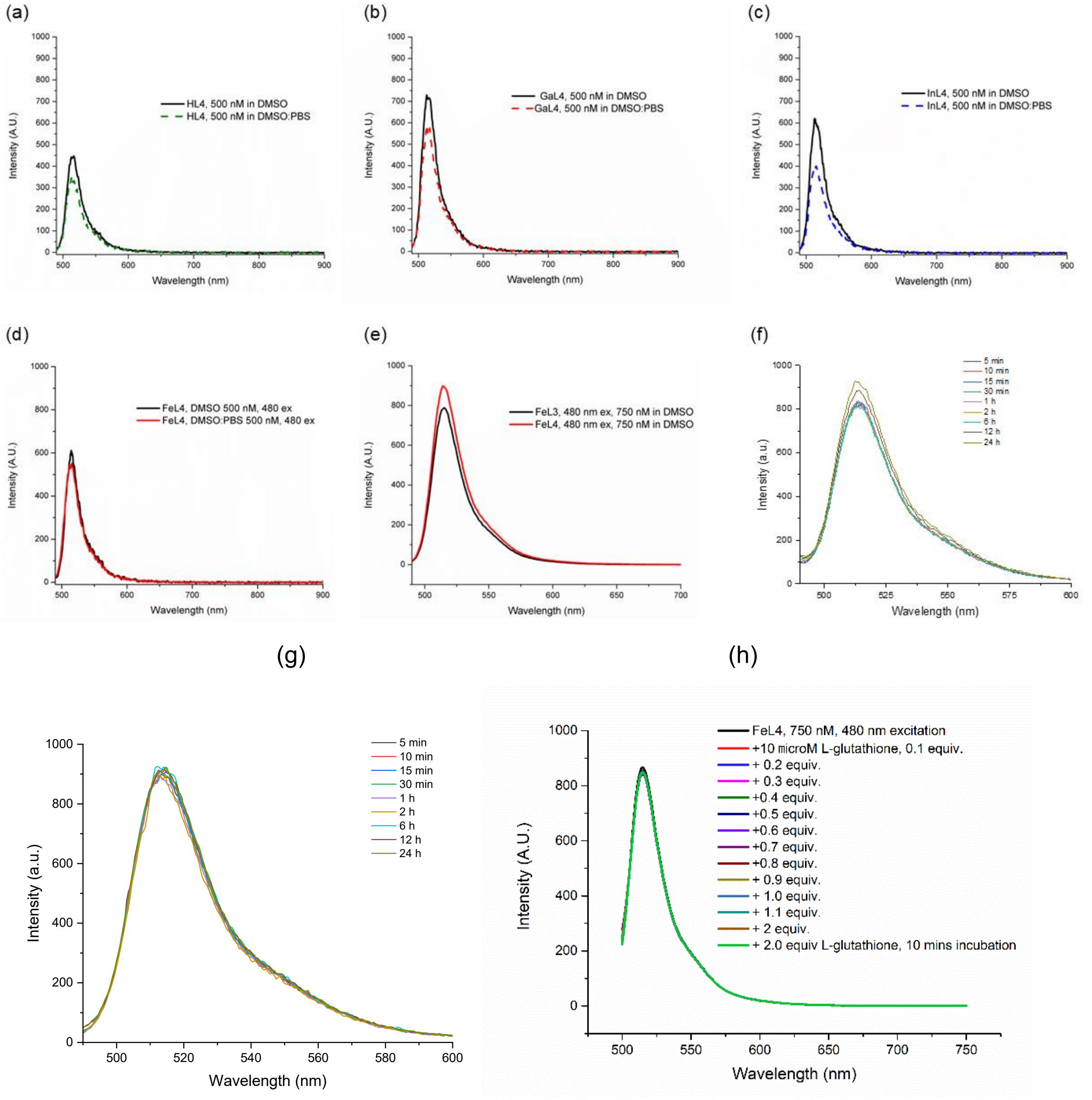

Figure S.44. Emisson spectra of **HL4 (a)**, **GaL4 (b)**, **InL4 (c)** and **FeL4 (d)** recorded at a concentration of 500 nM in DMSO and in DMSO:PBS (excitation 480 nm); **(e)** Emisson spectra of **FeL3** and **FeL4** at 750 nM in DMSO **(f)** Kinetic stability test for **GaL3**, 500 nM in 1:1 DMSO: RMPI Serum-free medium. **(g)** Kinetic stability test of **HL3**, 500 nM in 1:1 DMSO: RMPI Serum-free medium; **(h)** Kinetic stability test of **FeL4**, 750 nM in DMSO with the addition of 0-2 equiv. of L-glutathione.

.

Table S1. Photophysical data for selected **BODIPY**-based compounds discussed hereby. Concentration: 500 nM in MeOH. Relative quantum yields were recorded relative to a fluorescein standard in aq. 0.1 M conc. NaOH (Q.Y. 95% at λmax =495).

Compound	$\lambda_{max\text{-}abs}$ (nm)	$\lambda_{max\text{-}em}$ (nm)	Δλ (nm)	ϕ_F
**Bodipy-COOH**	503	510	7	0.45
**Bodipy-en**	503	512	9	0.34
**HL3**	503	515	12	0.36
**HL4**	503	514	11	0.37
**GaL3**	503	514	11	0.27
**InL3**	503	516	13	0.34
**FeL3**	503	513	10	0.12
**FeL4**	503	515	12	0.14

9. 2-Photon TCSPC Spectroscopy of Selected BODIPY conjugates

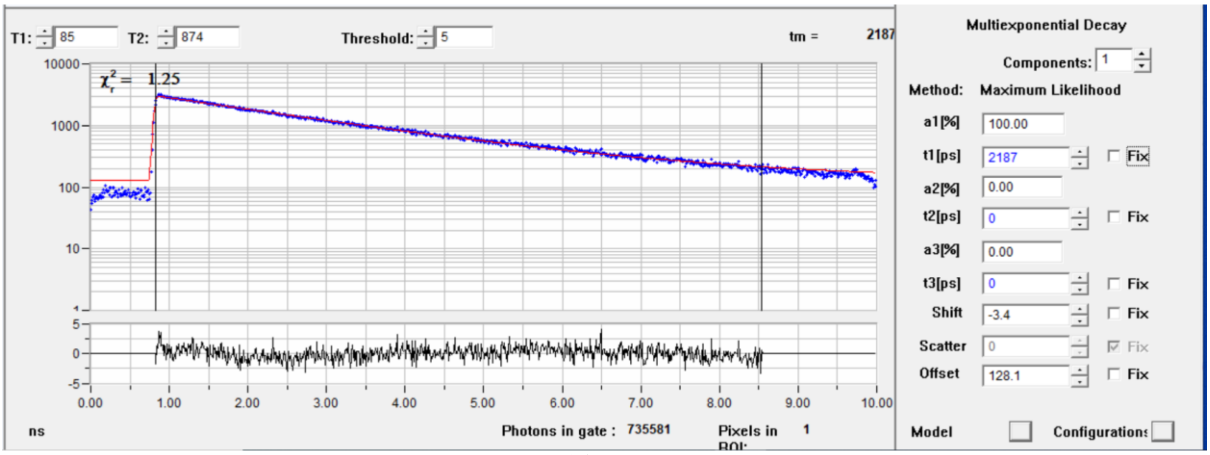

(a)

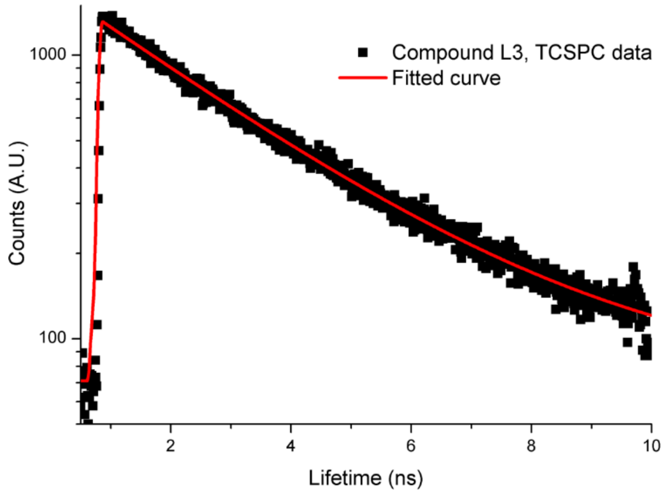

(b)

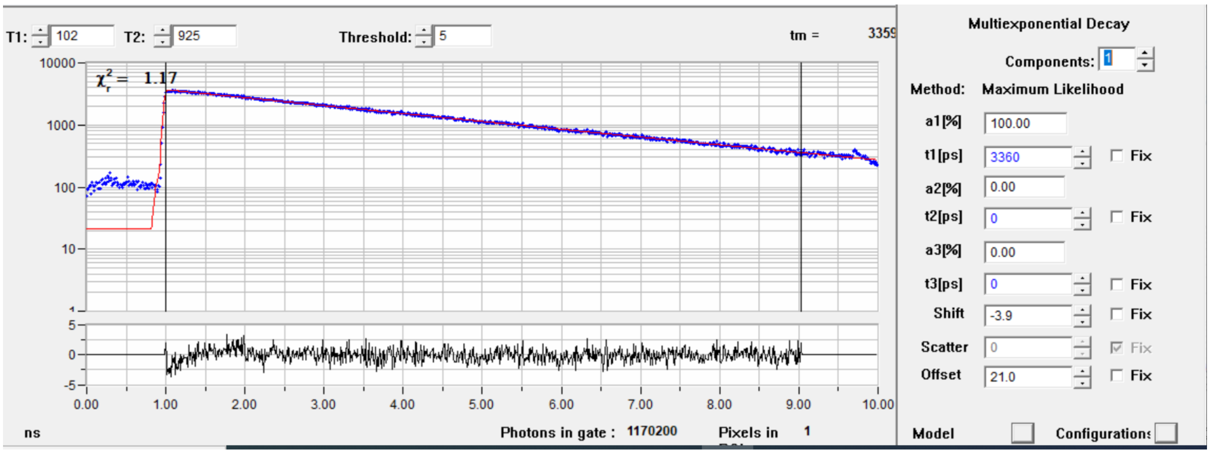
T1: 102
T2: 925
Threshold: 5
tm = 3359
χ^2_r = 1.17
Multiexponential Decay
Components: 1
Method: Maximum Likelihood
a1[%] 100.00
t1[ps] 3360 Fix
a2[%] 0.00
t2[ps] 0 Fix
a3[%] 0.00
t3[ps] 0 Fix
Shift -3.9 Fix
Scatter 0 Fix
Offset 21.0 Fix
ns
Photons in gate : 1170200
Pixels in 1
Model
Configurations

(c)

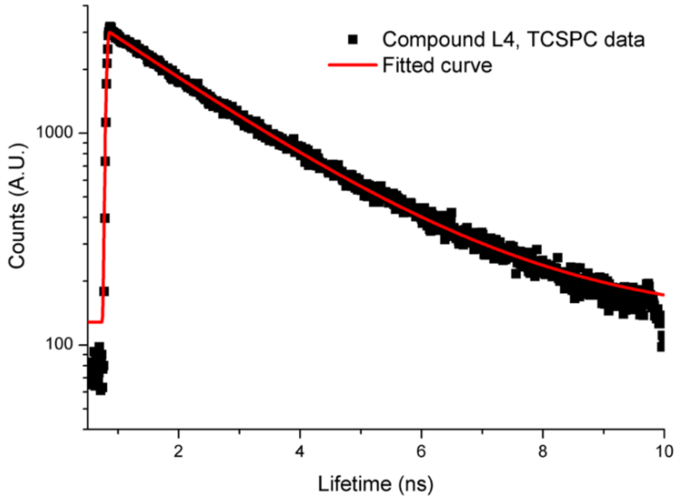
Compound L4, TCSPC data
Fitted curve
Counts (A.U.)
Lifetime (ns)
1000
100
2
4
6
8
10

(d)

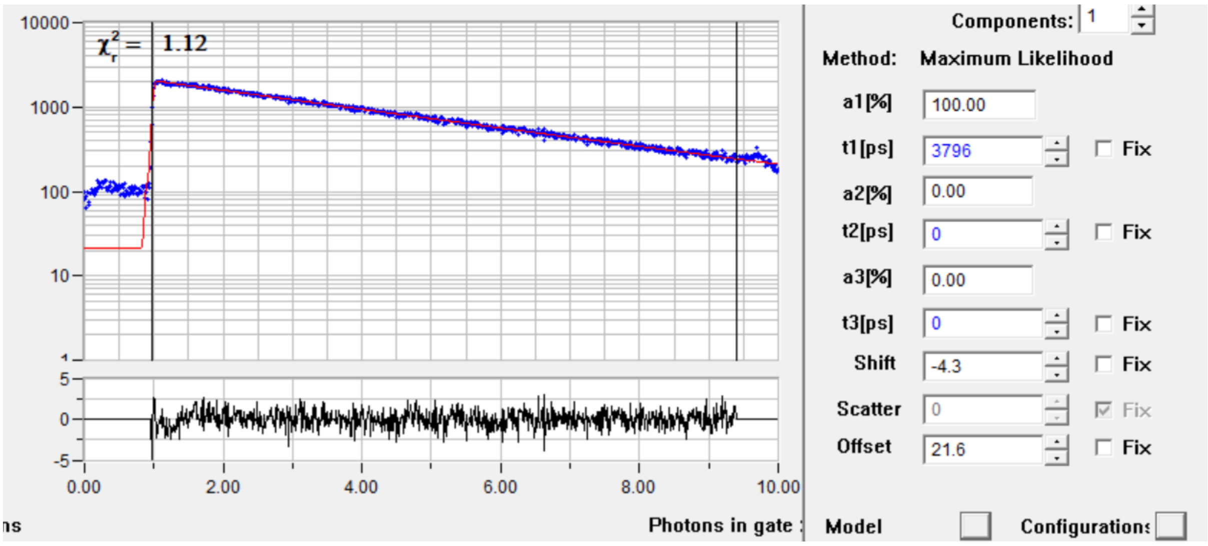

χ_r^2 = 1.12
Components: 1
Method: Maximum Likelihood
a1[%] 100.00
t1[ps] 3796 Fix
a2[%] 0.00
t2[ps] 0 Fix
a3[%] 0.00
t3[ps] 0 Fix
Shift -4.3 Fix
Scatter 0 Fix
Offset 21.6 Fix
Photons in gate
Model
Configurations

(e)

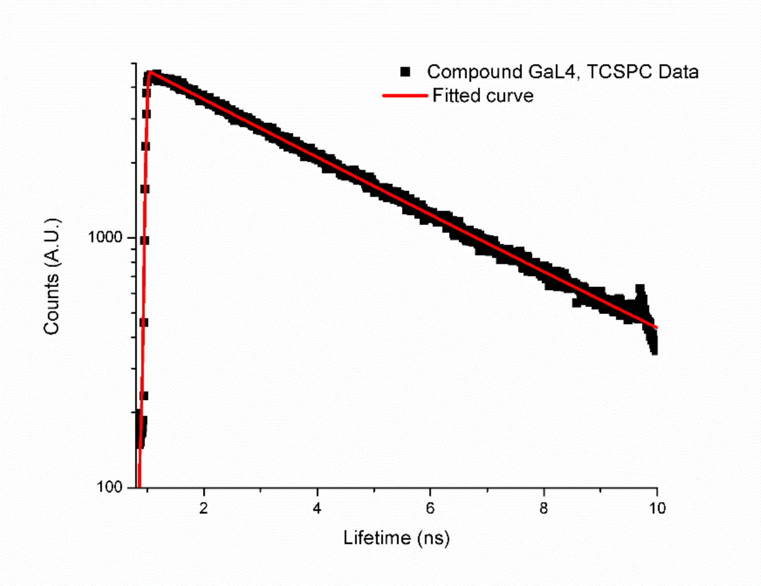

Compound GaL4, TCSPC Data
Fitted curve
Counts (A.U.)
Lifetime (ns)

(f)

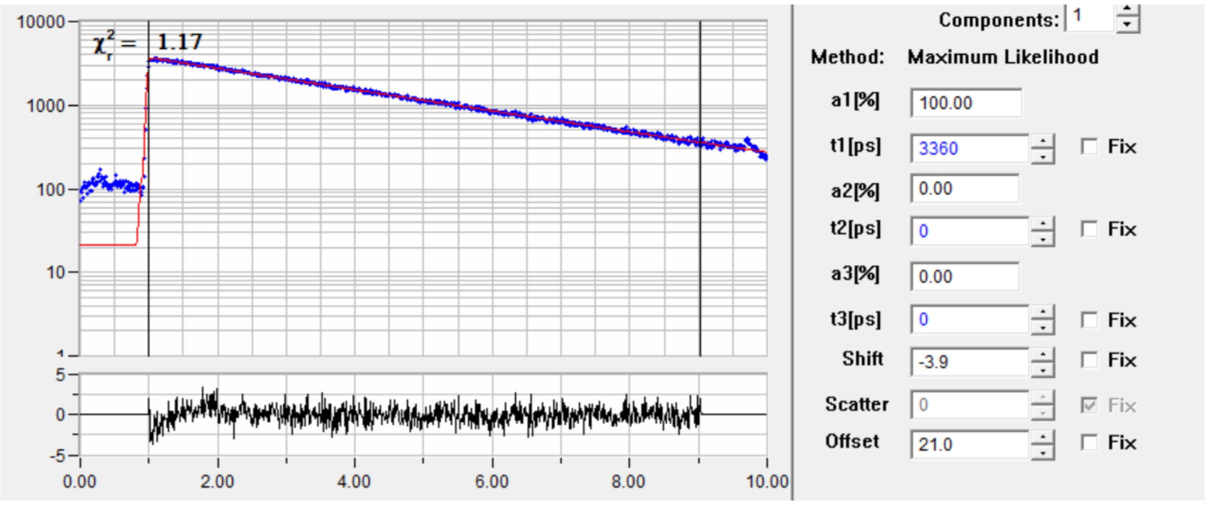

χ_r^2 = 1.17
Components: 1
Method: Maximum Likelihood
a1[%] 100.00
t1[ps] 3360 Fix
a2[%] 0.00
t2[ps] 0 Fix
a3[%] 0.00
t3[ps] 0 Fix
Shift -3.9 Fix
Scatter 0 Fix
Offset 21.0 Fix

(g)

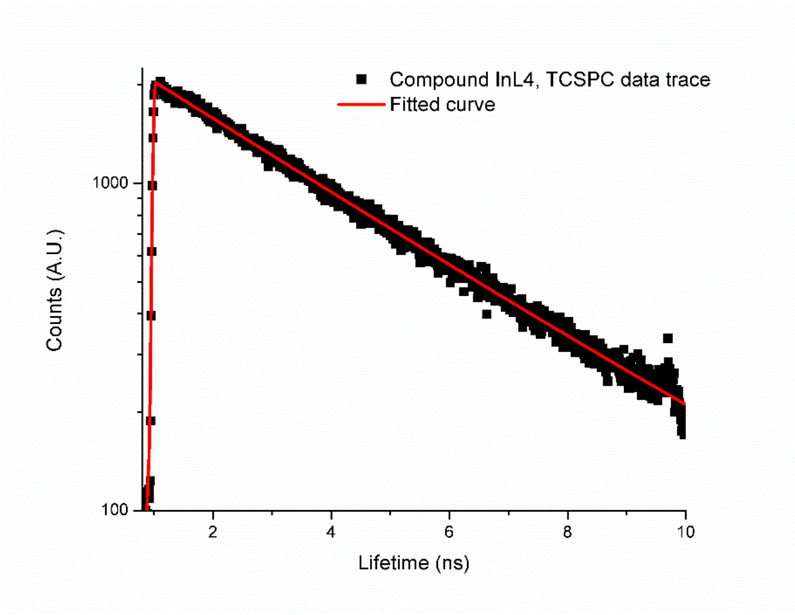

(h)

Figure S.45. 2 Photon TCSPC Spectroscopy of H**L3** (a and b) and **HL4** (c and d), 10 µM conc in DMSO. TCSPC parameters (a and c) and corresponding fitted data (b and d). 2 Photon TCSPC spectroscopy of **GaL4** (e and f) and **InL4** (g and h), 10 µM conc in DMSO. TCSPC parameters (e and g) and corresponding fitted data (f and h).

10. Cellular Viability Assays

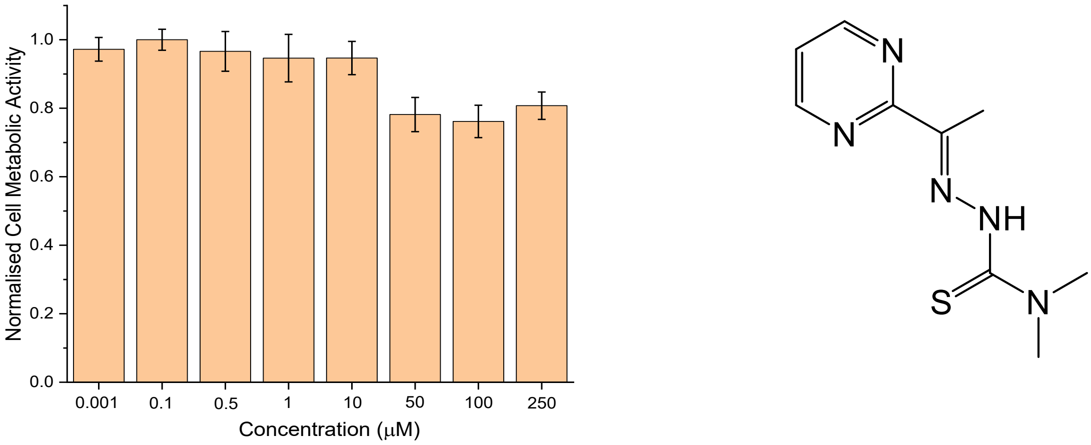

Figure S.46. Comparison of the 30 minutes MTT Assays of H**L1** in PC-3 cells, a timescale relevant to that used for imaging assays.

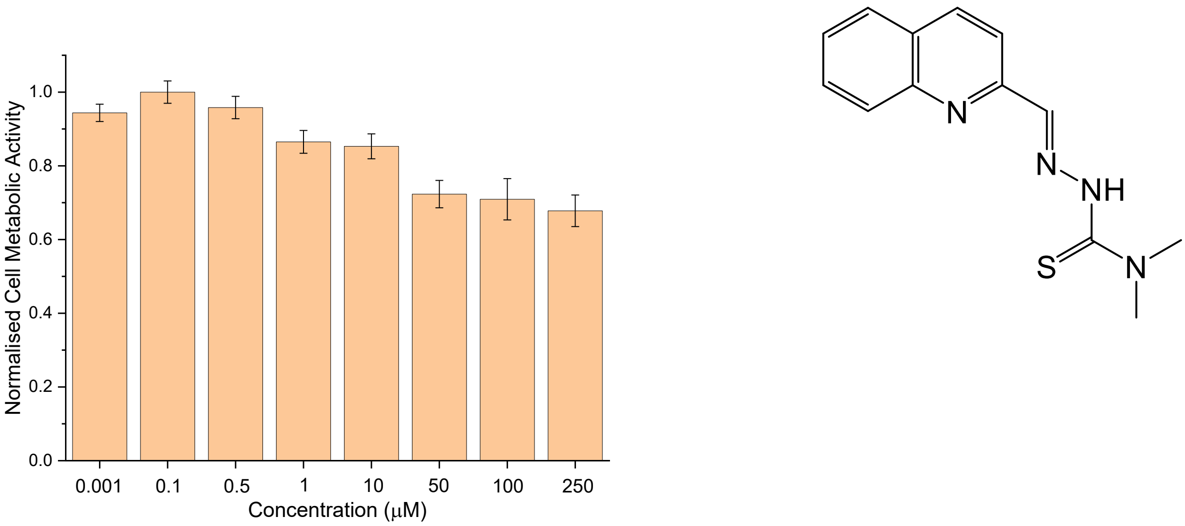

(b)

Figure S.47. Comparison of the 30 minutes MTT Assays of **HL2** compound in PC-3 cells, a timescale relevant to that used for imaging assays.

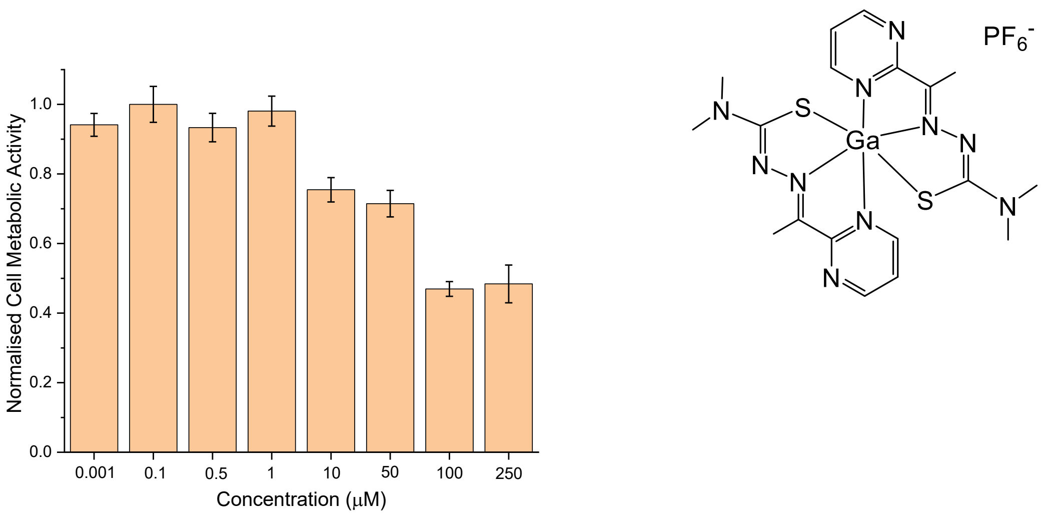

(a)

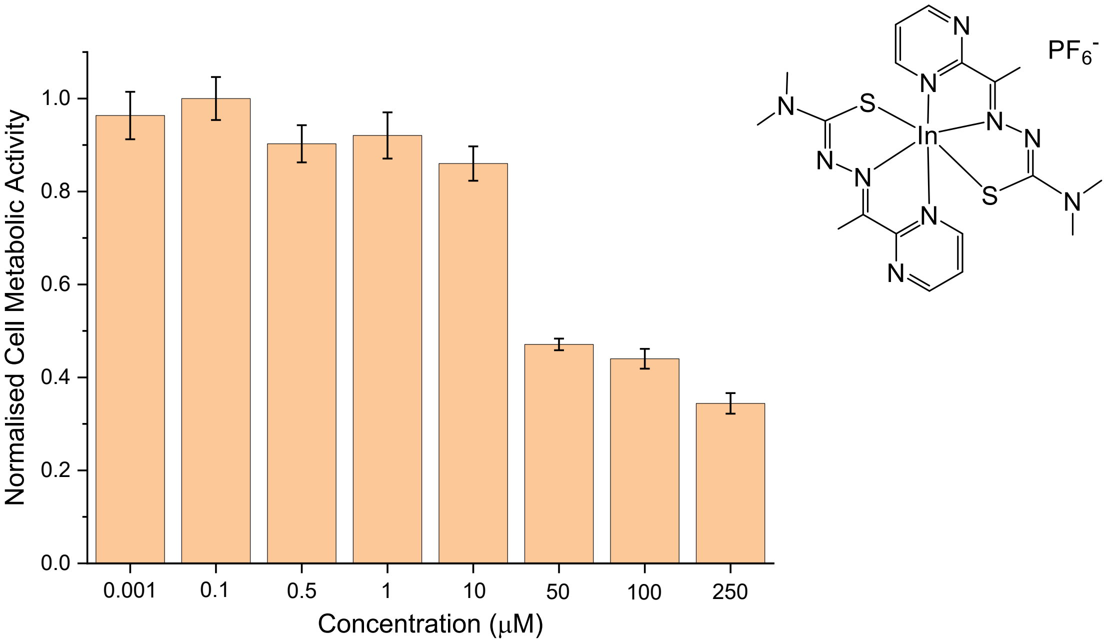

(b)

Figure S.48. Comparison of the 30 minutes MTT Assays of **GaL1** (a) and **InL1** (b) complexes in PC-3 cells, a timescale relevant to that used for imaging assays.

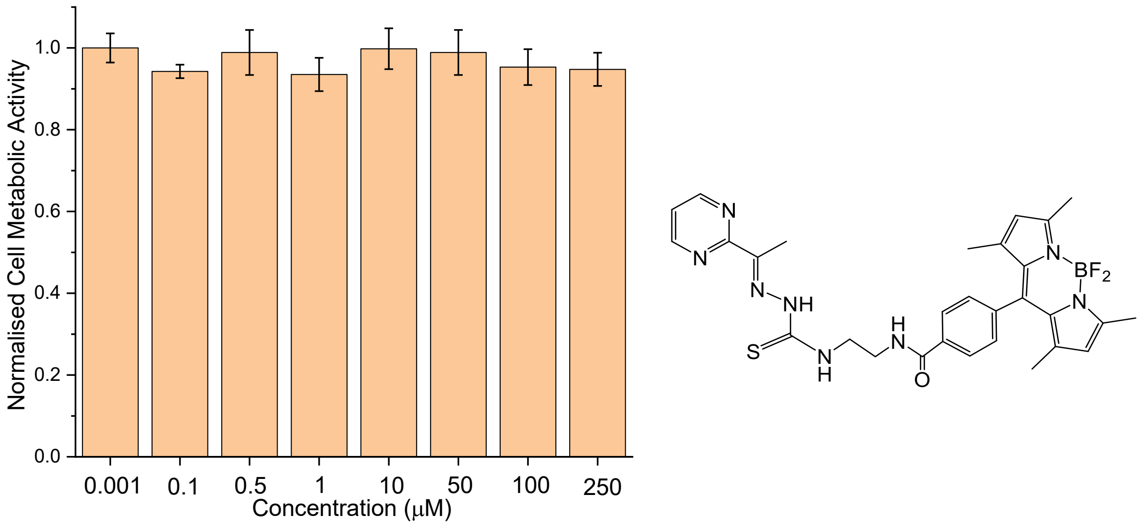

(a)

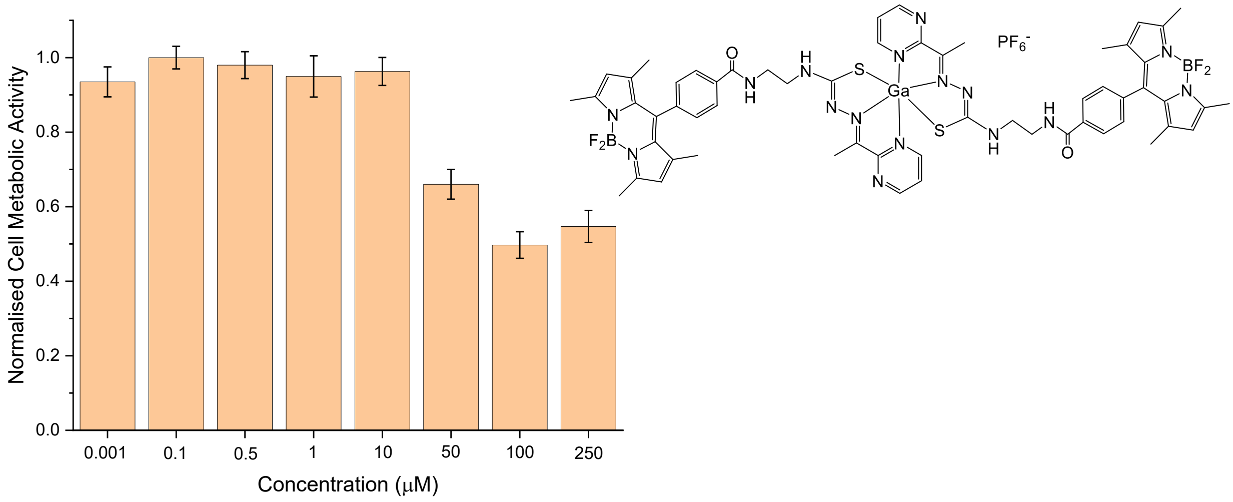

(b)

Figure S.49. Comparison of the 30 minutes MTT Assays of **HL3** (a) and **GaL3** (b) complexes in PC-3 cells, a timescale relevant to that used for imaging assays.

MTT Assays of Selected Compounds in PC-3 Cells over 48 h

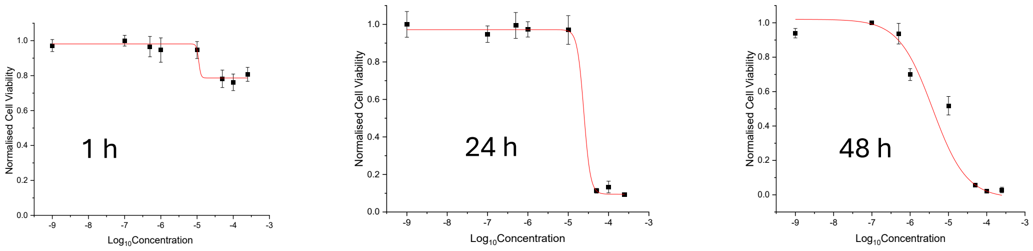

Figure S.50. Dose-response curves of PC-3 cells after 1 h, 24 h and 48 h (left to right) treatment with compound **HL1**. Data shown here were the average values from three independent experiments, error bars represent the standard deviation.

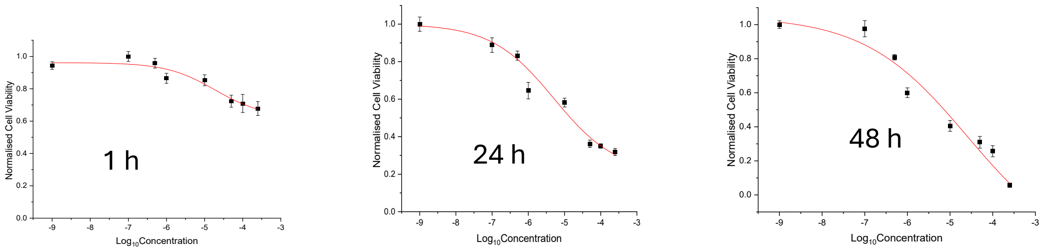

Figure S.51. Dose-response curves of PC-3 cells after 1 h, 24 h and 48 h (left to right) treatment with compound **HL2.** Data shown here were the average values from three independent experiments, error bars represent the standard deviation.

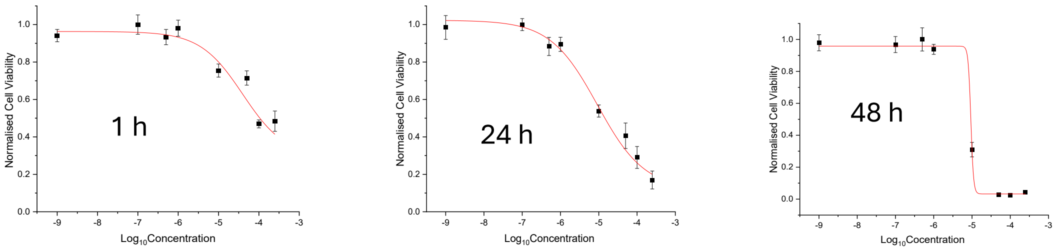

Figure S.52. Dose-response curves of PC-3 cells after 1 h, 24 h and 48 h (left to right) treatment with complex **GaL1**. Data shown here were the average values from three independent experiments, error bars represent the standard deviation.

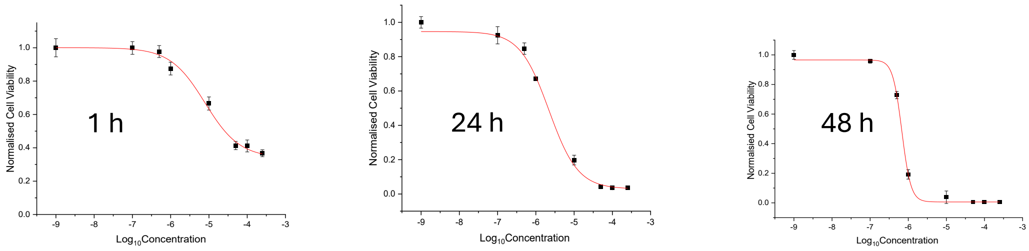

Figure S.53. Dose-response curves of PC-3 cells after 1 h, 24 h and 48 h (left to right) treatment with complex **InL1**. Data shown here were the average values from three independent experiments, error bars represent the standard deviation.

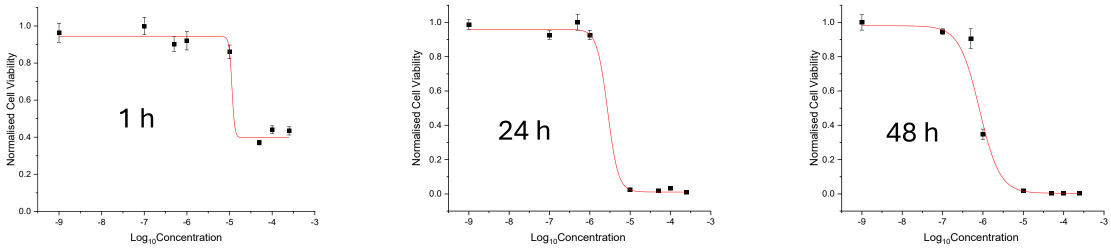

Figure S.54. Dose-response curves of PC-3 cells after 1 h, 24 h and 48 h (left to right) treatment with complex **GaL2**. Data shown here were the average values from three independent experiments, error bars represent the standard deviation.

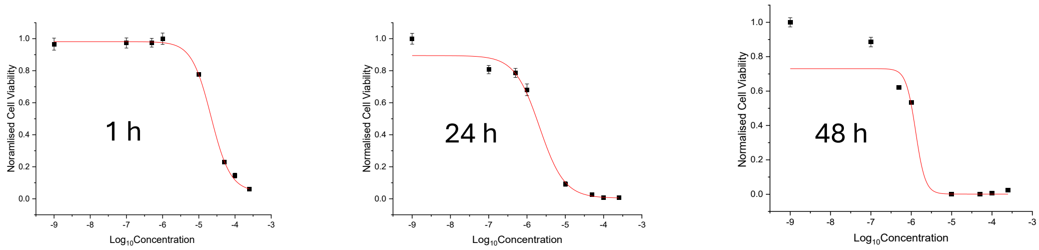

Figure S.55. Dose-response curves of PC-3 cells after 1 h, 24 h and 48 h (left to right) treatment with complex **InL2**. Data shown here were the average values from three independent experiments, error bars represent the standard deviation.

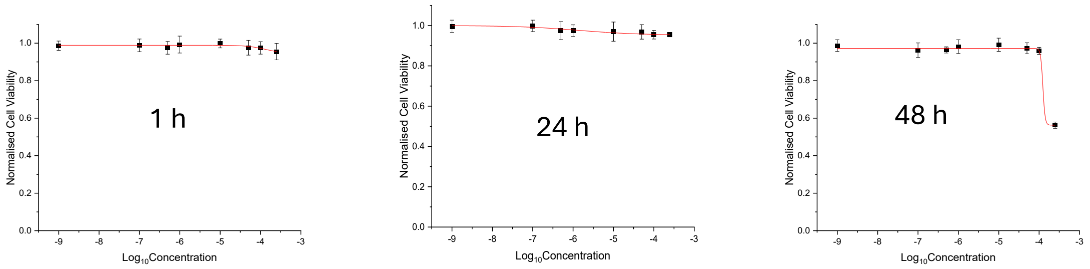

Figure S.56. Dose-response curves of PC-3 cells after 1 h, 24 h and 48 h (left to right) treatment with compound **HL3**. Data shown here were the average values from three independent experiments, error bars represent the standard deviation.

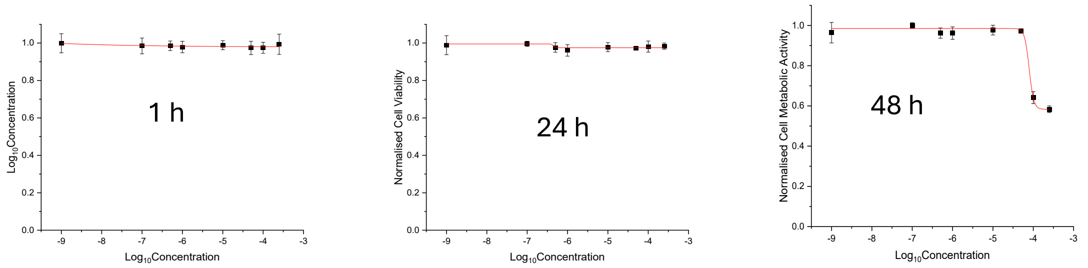

Figure S.57. Dose-response curves of PC-3 cells after 1 h, 24 h and 48 h (left to right) treatment with compound **HL4**. Data shown here were the average values from three independent experiments, error bars represent the standard deviation.

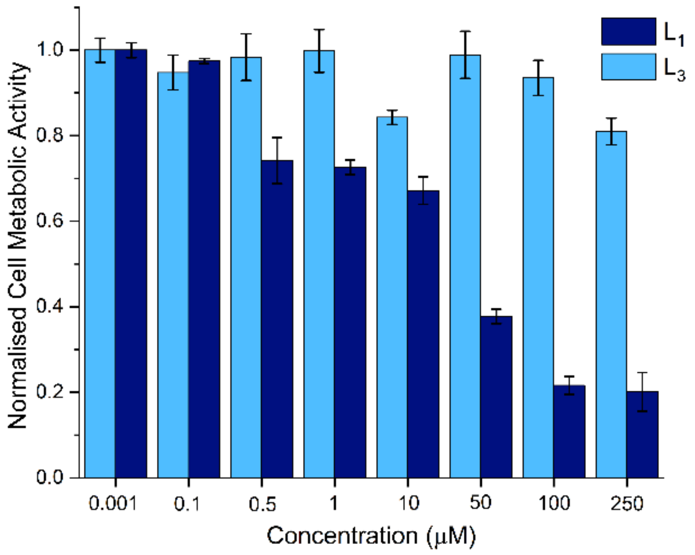

Figure S.58. A comparison of MTT assays data for **HL1** vs **HL3** in PC-3 over 24 h assays showing the significant reduction in cytotoxicity as a result of the BODIPY tag incorporation in the TSC structure.

HL1

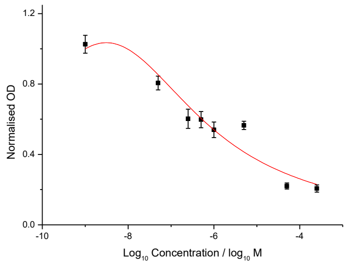

InL1

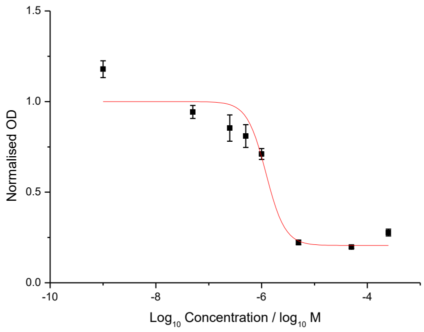

GaL1

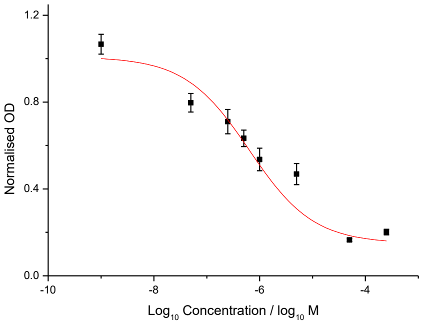

HL2

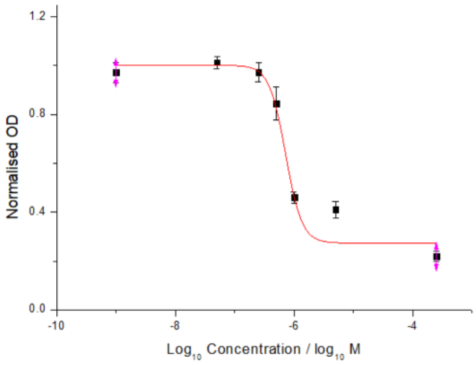

InL2

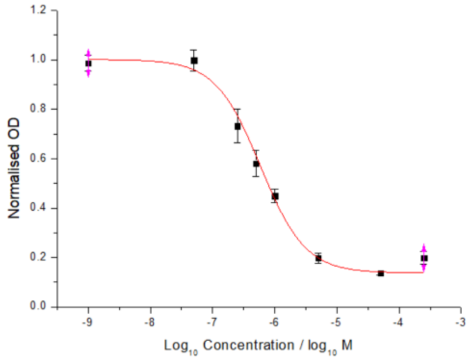

GaL2

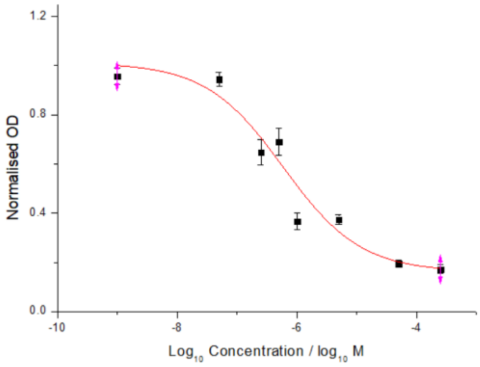

Figure S.59. Selected dose-response curves of HeLa cells after 24 h treatment with selected compounds. Data shown here were the average values from three independent experiments, error bars represent the standard deviation.

Table S2. Estimated IC_{50} values (μM) corresponding to Dose-response data for MTTs in HeLa cells after 24 h treatment with selected compounds

MTT (24 h)	HL1	InL1	GaL1	HL2	InL2	GaL2
IC50 (μM)	1.588	1.507	1.068	0.921	0.772	1.092

HL4

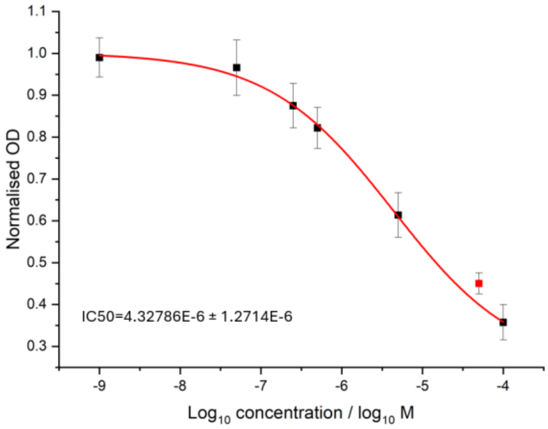

GaL4

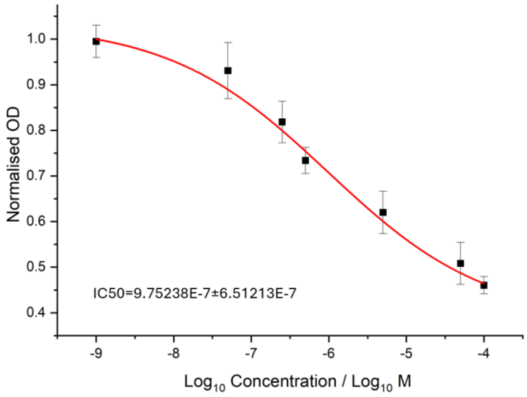

InL4

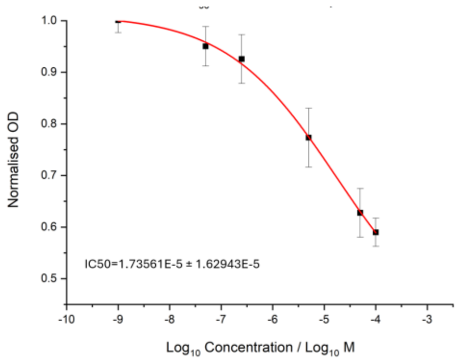

Figure S.60. Selected dose-response curves of HeLa cells after 24 h treatment with **HL4**, **GaL4** and **InL4** series. Data shown here were the average values from three independent

experiments, error bars represent the standard deviation. Estimated IC_{50} values (M) are summarised within graphs above

Table S3. IC50 values for PC-3 (human prostate cancer cells) following exposure to TSC ligands **HL1 and HL2** for 24 h and 48 h and compared to cisplatin tested under the same experimental conditions (1% DMSO).

Compound	24h IC_{50} (µM)	48 h IC_{50} (µM)
**Cis-Platin**	(100-250)[#]	30.65 ± 3.25
**HL1**	24.35 ± 1.33	3.36 ± 0.31
**HL2**	> 100[#]	25.64 ± 7.91

[a] N.B. Data presented as mean ± SD, n = 3; SD: standard deviation; ns: no significant cytotoxicity compared with the control group (p > 0.05). Data of some treatment groups could not be fitted with a good dose-response curve and the exact IC_{50} values could not be calculated; their IC_{50} values were expressed as concentration ranges and labelled with "#"

Table S.4. IC50 values for PC-3 (human prostate cancer cells) following exposure to selected TSC complexes **GaL1, GaL2, InL1 and InL2** for 24 h and 48 h.

Compound	24h IC_{50} (µM)	48h IC_{50} (µM)
**GaL1**	(10-50)[#]	9.34 ± 0.35
**InL1**	2.20 ± 0.98	0.68 ± 0.02
**GaL2**	2.76 ± 1.02	0.82 ± 0.14
**InL2**	2.10 ± 0.68	1.06 ± 0.28

[a] N.B. Data presented as mean ± SD, n = 3; SD: standard deviation; ns: no significant cytotoxicity compared with the control group (p > 0.05). Data of some treatment groups could not be fitted with a good dose-response curve and the exact IC_{50} values could not be calculated; their IC_{50} values were expressed as concentration ranges and labelled with "#".

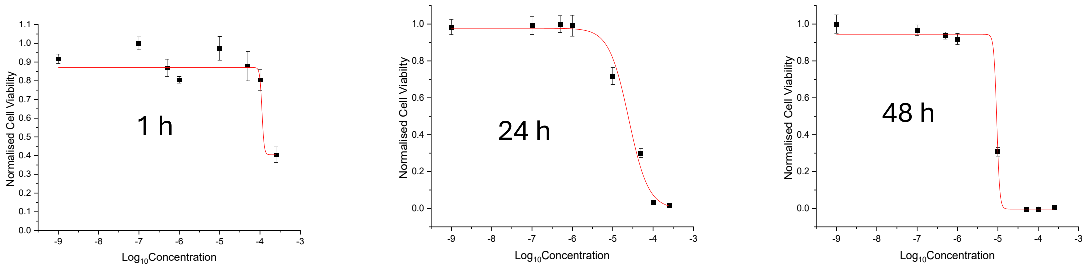

Figure S.61. Dose-response curves of PC-3 cells after 1 h, 24 h and 48 h (left to right) treatment with compound **GaL3**. Data shown here were the average values from three independent experiments, error bars represent the standard deviation.

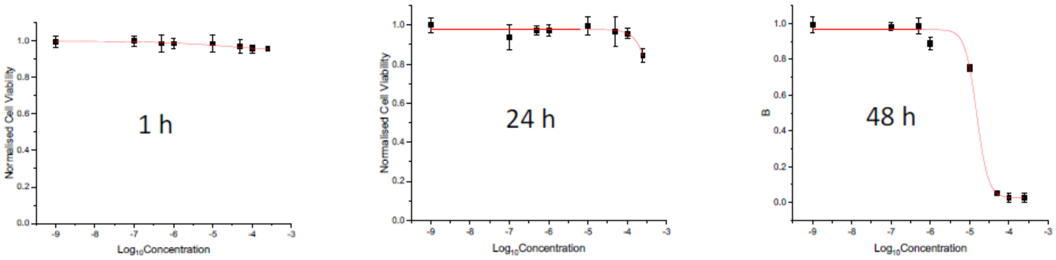

(a)

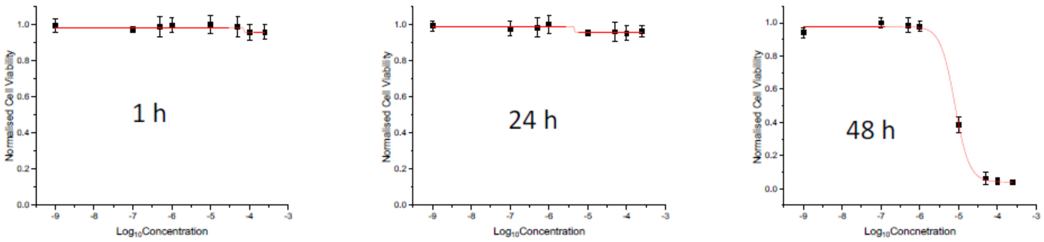

(b)

Figure S.62. Dose-response curves of PC-3 cells after 1 h, 24 h and 48 h (left to right) treatment with complex **FeL3** (a) and FeL4 (b) Data shown here were the average values from three independent experiments, error bars represent the standard deviation.

Table S.5. IC_{50} values for PC-3 (human prostate cancer cells) following exposure to selected compounds in the **HL3** series for 24 h and 48 h, performed within the same series of experiments. Based on dose response curves from MTT assays. [a]

Compound	24h IC_{50} (μM)	48 h IC_{50} (μM)
**HL3**	ns	> 250#
**GaL3**	(10-50)#	3.32 ± 0.84
**InL3**	25.62 ± 5.41	9.42 ± 0.05
**FeL3**	> 250#	36.64 ± 2.62
**FeL4**	> 250#	7.10 ± 1.72

[a] *N.B. Data presented as mean ± SD, n = 3; SD: standard deviation; ns: no significant cytotoxicity compared with the control group (p > 0.05). Data of some treatment groups could not be fitted with a good dose-response curve and the exact IC_{50} values could not be calculated; their IC_{50} values were expressed as concentration ranges and labelled with "#".*

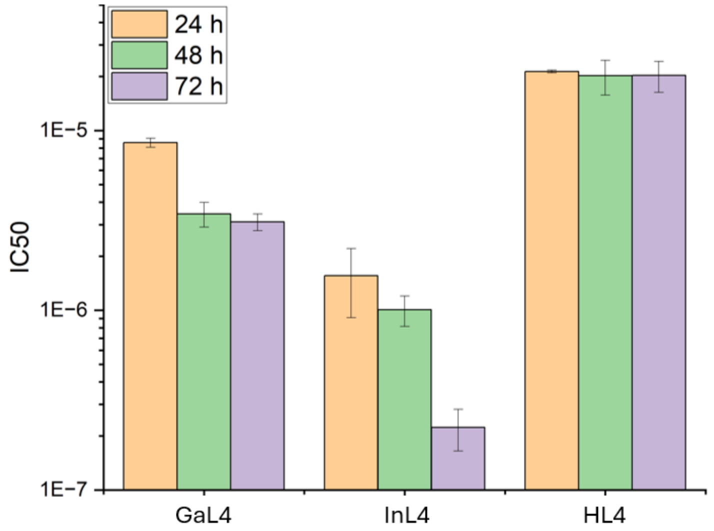

	IC50 GaL4 (M)	std dev.	IC50 InL4 (M)	std. dev.	IC50 HL4 (M)	std dev.
**24 h**	8.57E-06	4.96E-07	1.56E-06	6.49E-07	2.13E-05	3.85E-07
**48 h**	3.44E-06	5.37E-07	1.01E-06	1.94E-07	2.02E-05	4.45E-06
**72 h**	3.10E-06	3.33E-07	2.23E-07	5.83E-08	2.03E-05	4.01E-06

Figure S.63. IC_{50} values for PC-3 (human prostate cancer cells) following MTT assays of **HL4**, **GaL4** and **InL4** over 24 h, 48 and 72 h.

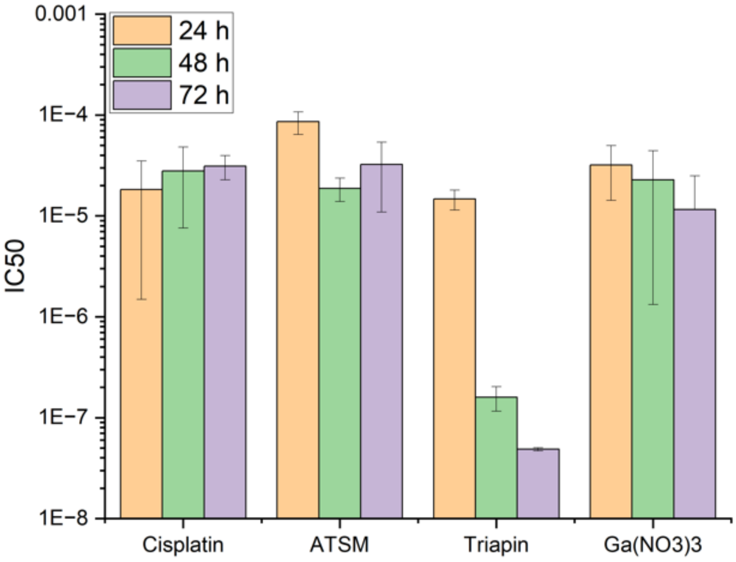

	IC50 24 h (M)	std. dev.	IC50 48 h (M)	std. dev.	IC5 72 h (M)	std dev
Cisplatin	1.82E-05	1.67E-05	2.78E-05	2.03E-05	3.12E-05	8.45E-06
ATSM	8.58E-05	2.15E-05	1.88E-05	4.89E-06	3.24E-05	2.15E-05
Triapin	1.47E-05	3.29E-06	1.59E-07	4.37E-08	4.87E-08	1.72E-09
Ga(NO3)3	3.20E-05	1.78E-05	2.28E-05	2.15E-05	1.16E-05	1.33E-05

Figure S.64. Control experiments: IC_{50} values for PC-3 (human prostate cancer cells) following exposure to clinically relevant commercial compounds under the same MTT assays conditions to those determined in the series over 24 h, 48 and 72 h. Cisplatin was dissolved in saline with 0.1% DMSO. Triapine^{TM}, ATSM and Ga(NO3) were dissolved in serum free medium with 1% DMSO

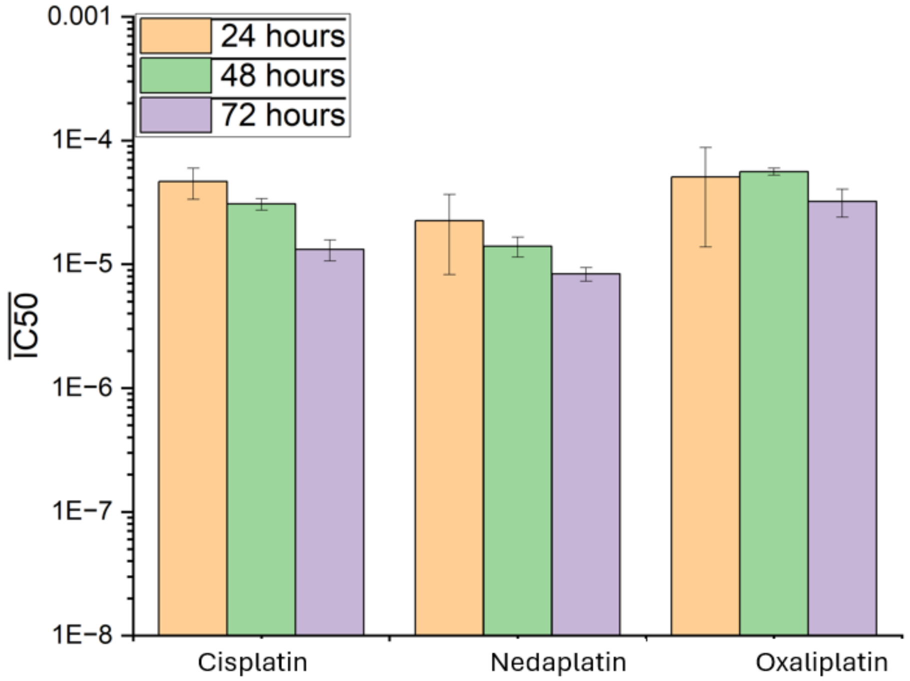

	24 hours IC50 (M)	std. dev	48 hours IC50 (M)	std. dev.	72 hours IC50 (M)	std. dev.
Cisplatin	4.65E-05	1.30E-05	3.06E-05	3.26E-06	1.31E-05	2.54E-06
Nedaplatin	2.25E-05	1.42E-05	1.40E-05	2.56E-06	8.35E-06	1.04E-06
Oxaliplatin	5.06E-05	3.68E-05	5.61E-05	3.55E-06	3.22E-05	8.20E-06

(**a**)

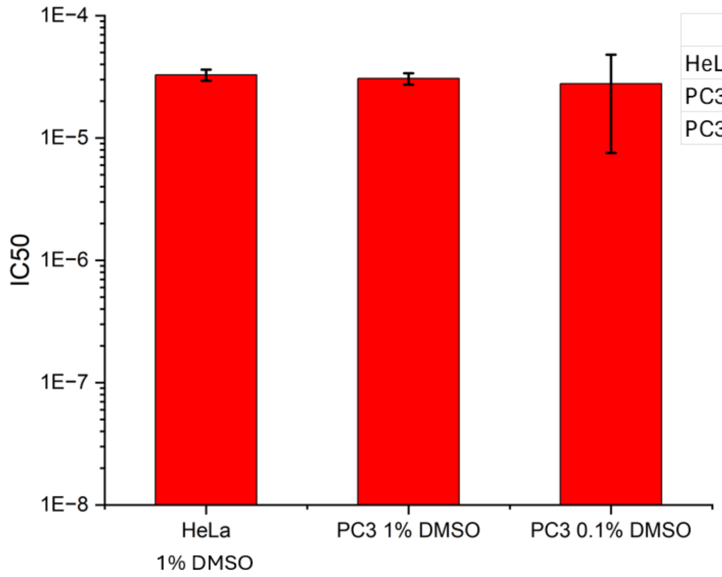

	IC50 48 h (M)	Std. dev.
HeLa	3.29E-05	3.41E-06
PC3 1% DMSO	3.06E-05	3.26E-06
PC3 0.1% DMSO	2.78E-05	2.03E-05

(b)

Figure S.65. Control experiments: IC_{50} values for PC-3 (human prostate cancer cells) following exposure to clinically relevant commercial compounds cisplatin, oxaliplatin and nedaplatin under the same MTT assays conditions to those determined in the series over 24 h,

48 and 72 h (1% DMSO). (b) Independent control experiment whereby cisplatin was dissolved in either 1% DMSO with serum free medium or in saline with 0.1% DMSO. Oxaliplatin and nedaplatin were dissolved in serum free medium with 1% DMSO.

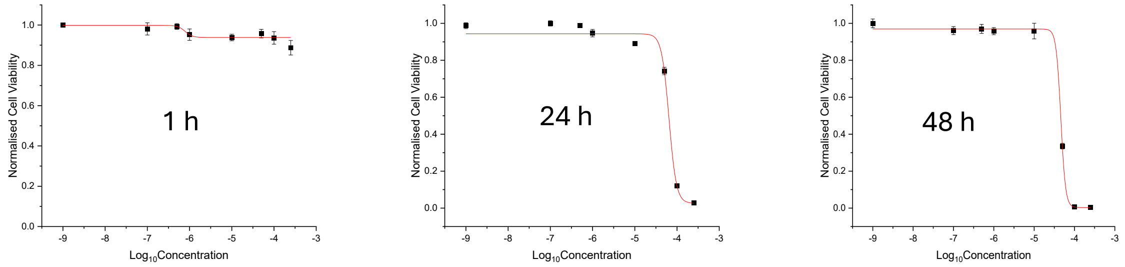

Figure S.66. Dose-response curves in the healthy cells line AG09429 after 1 h, 24 h and 48 h (left to right) treatment with compound **HL1**. Data shown here were the average values from three independent experiments, error bars represent the standard deviation.

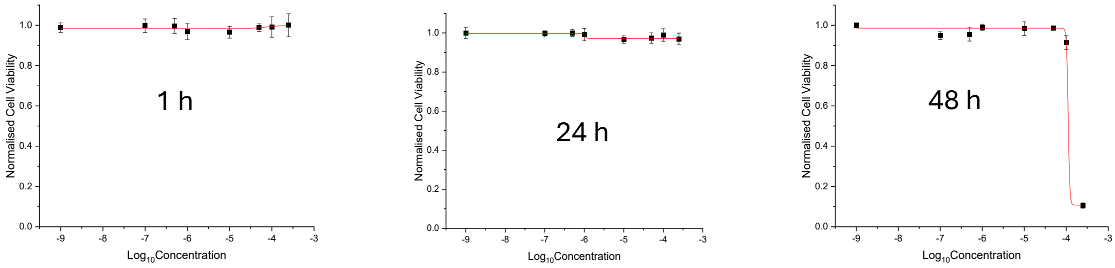

Figure S.67. Dose-response curves the healthy cells line AG09429 after 1 h, 24 h and 48 h (left to right) treatment with compound **HL2**. Data shown here were the average values from three independent experiments, error bars represent the standard deviation.

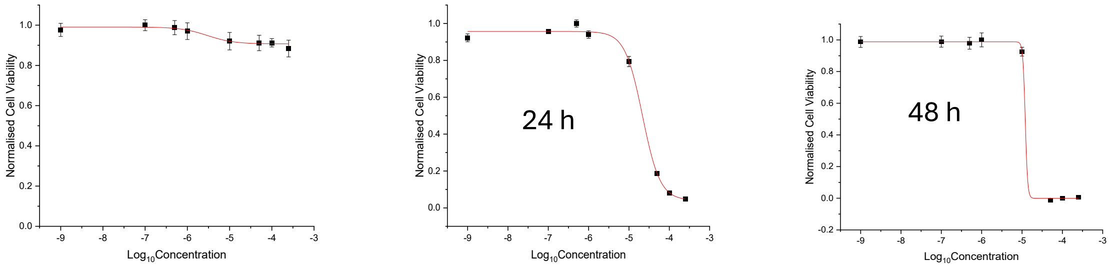

Figure S.68. Dose-response curves of AG09429 cells after 1 h, 24 h and 48 h (left to right) treatment with complex **GaL1**. Data shown here were the average values from three independent experiments, error bars represent the standard deviation.

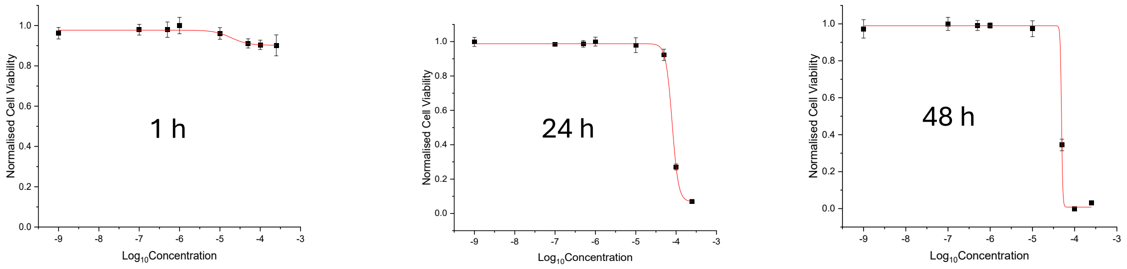

Figure S.69. Dose-response curves of AG09429 cells after 1 h, 24 h and 48 h (left to right) treatment with complex **InL1**. Data shown here were the average values from three independent experiments, error bars represent the standard deviation.

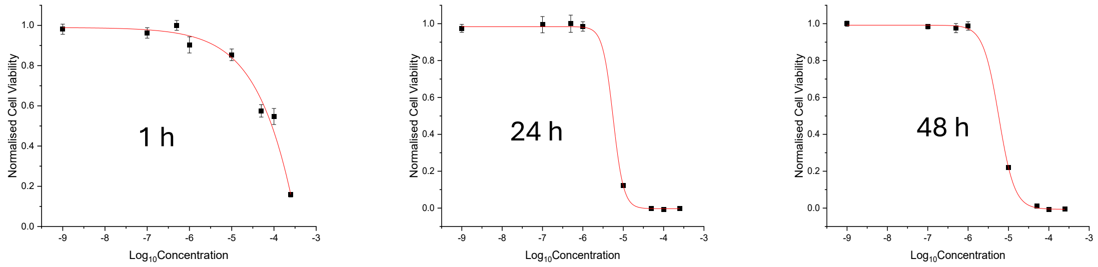

Figure S.70. Dose-response curves of AG09429 cells after 1 h, 24 h and 48 h (left to right) treatment with complex **GaL2**. Data shown here were the average values from three independent experiments, error bars represent the standard deviation.

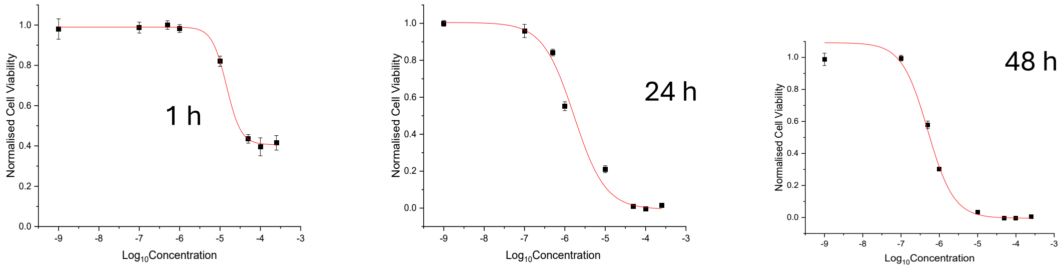

Figure S.71. Dose-response curves of AG09429 cells after 1 h, 24 h and 48 h (left to right) treatment with complex **InL2**. Data shown here were the average values from three independent experiments, error bars represent the standard deviation.

Table S.6. IC50 values for AG09429 (healthy controls, human gingival fibroblast) following exposure to selected compounds **1-6** for 24 h and 48 h.

Compound	24h IC_{50} (μM)	48 h IC_{50} (μM)
**Cis-Platin**	ns	ns
**HL1**	61.6 ± 2.13	46.88 ± 4.46
**HL2**	ns	109.70 ± 12.54
**GaL1**	22.12 ± 1.21	12.18 ± 1.15
**InL1**	79.62 ± 9.34	49.35 ± 2.41
**GaL2**	8.02 ± 0.01	5.88 ± 0.13
**InL2**	1.59 ± 0.59	0.53 ± 0.14

[a] *N.B. Data presented as mean ± SD, n = 3; SD: standard deviation; ns: no significant cytotoxicity compared with the control group (p > 0.05). Data of some treatment groups could not be fitted with a good dose-response curve and the exact IC_{50} values could not be calculated; their IC_{50} values were expressed as concentration ranges and labelled with "#".*

11. Selected Single Photon Confocal Fluorescence Microscopy in Living cells

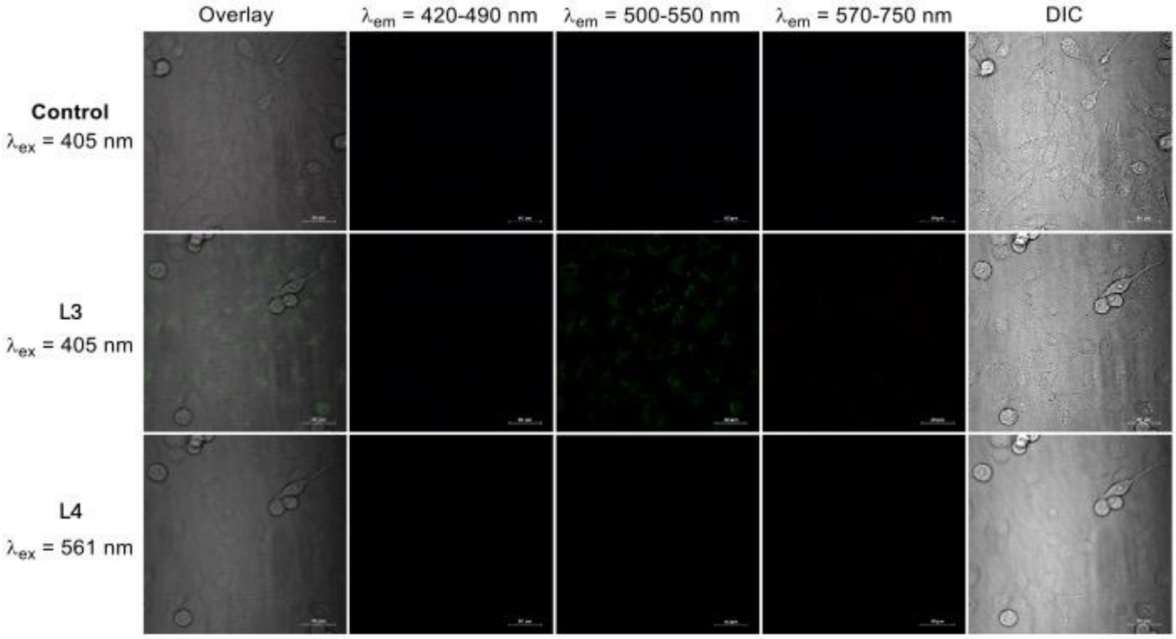

Figure S.72. Single photon confocal cell images in PC-3 cells of: Control group at 405 nm excitation (top row), compound **HL3** at 100 nM concentration and 405 nm excitation (middle row), and compound **HL4** at 100 nM concentration and 561 nm excitation (bottom row). Treatment time of 20 minutes at 37 °C. Scale bar: 50 µm.

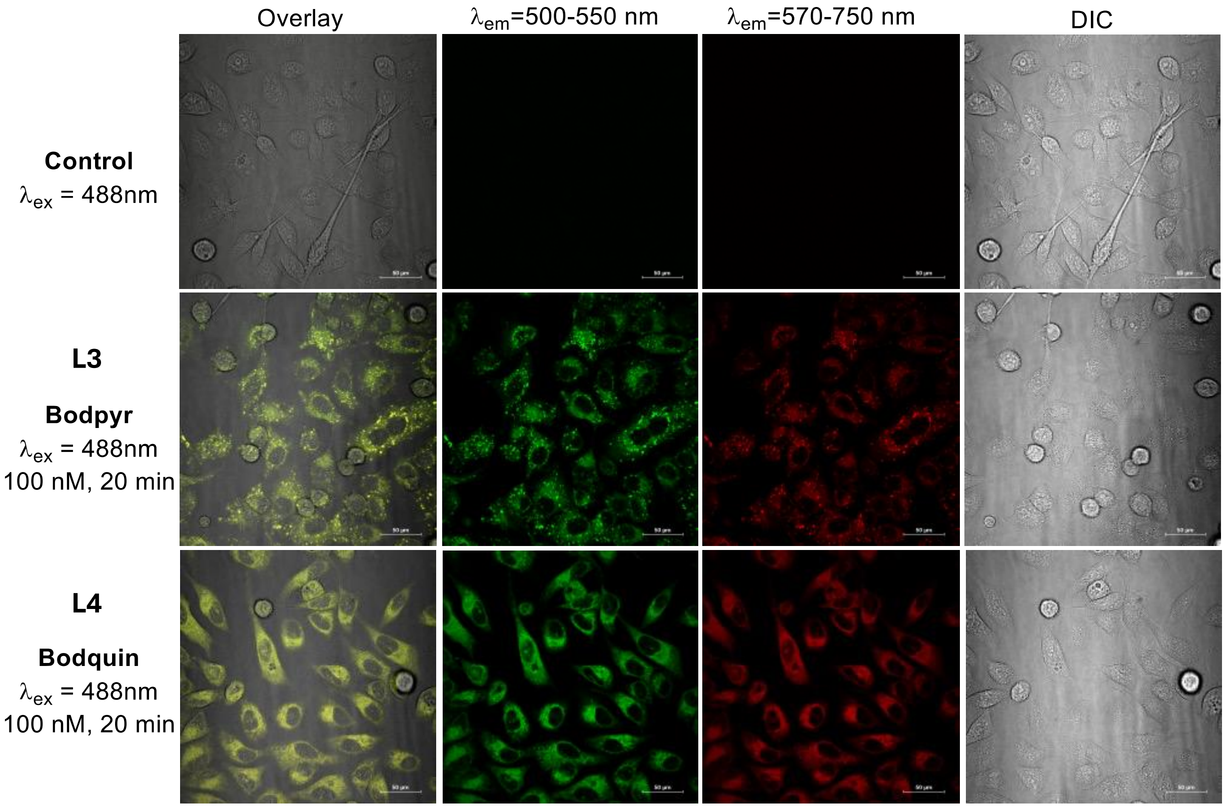

Figure S.73. Single photon confocal cell images in PC-3 cells of: Control group at 488 nm excitation (top row), compound **HL3** at 100 nM concentration and 488 nm excitation (middle row), and compound **HL4** at 100 nM concentration and 488 nm excitation (bottom row). Treatment time of 20 minutes at 37 °C. Scale bar: 50 μm.

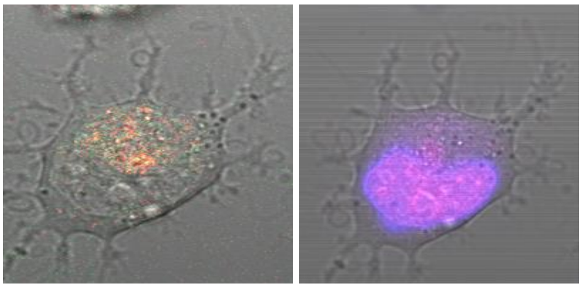

(a) **(b)**

Figure S.74. Laser confocal imaging in PC-3 cells incubated with the **InL2** and Hoechst dye Co-localisation (20 min incubation, 50 µM compound, 1% DMSO, 37 ºC) (a): Overlay of DIC, Red and green channels, ex 488 nm; (b) Overlay of DIC and Blue channel after staining of nucleus using Hoechst dye.

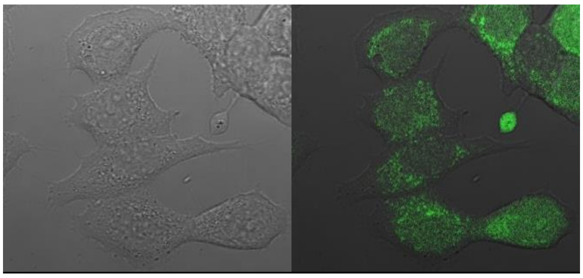

(a) **(b)**

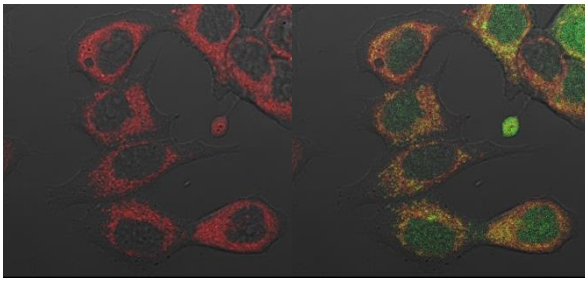

(c) **(d)**

Figure S.75. Confocal imaging and colocalisation in PC-3 cells using the **InL2** complex (20 min incubation, 50 µM compound, 1% DMSO, 37 ºC): (a) brightfield image of PC-3 cells (ex 405 nm); field of view 50 micrometer (b) complex **InL2**, green channel, ex 488 nm (c) ER tracker, ex 532 nm, red channel (d) overlay of complex and ER tracker emission channels.

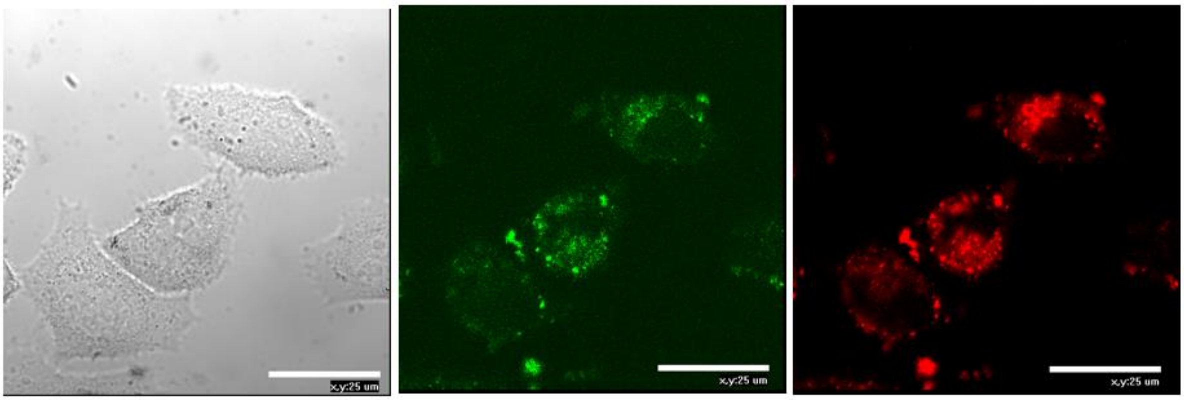

Figure S.76. Colocalisation confocal images of dimethyl-substituted Indium quinoline thiosemicarbazide complex **InL2,** (50 µM compound, 1% DMSO, 37 ºC). Left to right: brightfield image of PC3 cells, the InL2 complex 488 nm ex. emission in green channel and Lysotracker-Red co-staining (561 m ex, red emission channel),

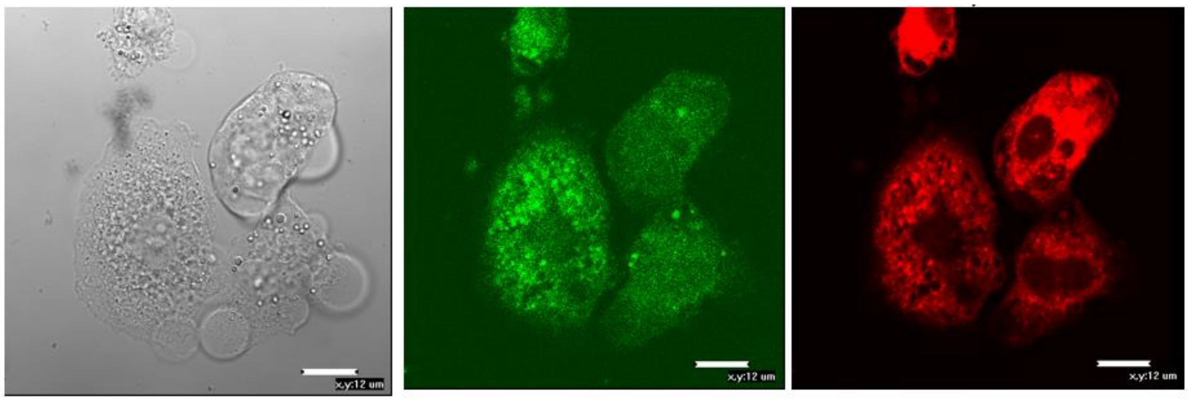

Figure S.77. Colocalisation confocal images of dimethyl-substituted Indium quinoline thiosemicarbazide complex **InL2** (50 µM compound, 1% DMSO, 37 ºC). Left to right: brightfield image of PC-3 cells, the Indium quinoline thiosemicarbazide complex emission in green channel (ex 488 nm), MitoTracker Red-costaining (ex 561 nm, red emission channel).

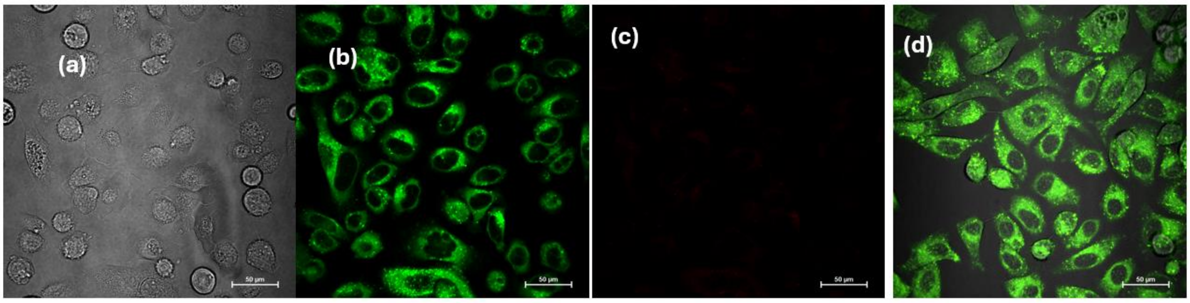

Figure S78. Confocal laser-scanning microscopy of PC-3 cells incubated at 37 °C for 20 min with **GaL3** (100 nM in 1:99 DMSO:serum-free medium). (a) bright field channel; (b) green channel (λ_{em}=500-550 nm); (c) red channel (λ_{em}=570-750 nm); (d) overlay of the DIC-green-red channels. λ_{ex}=488 nm, Scale bar: 50 µm.

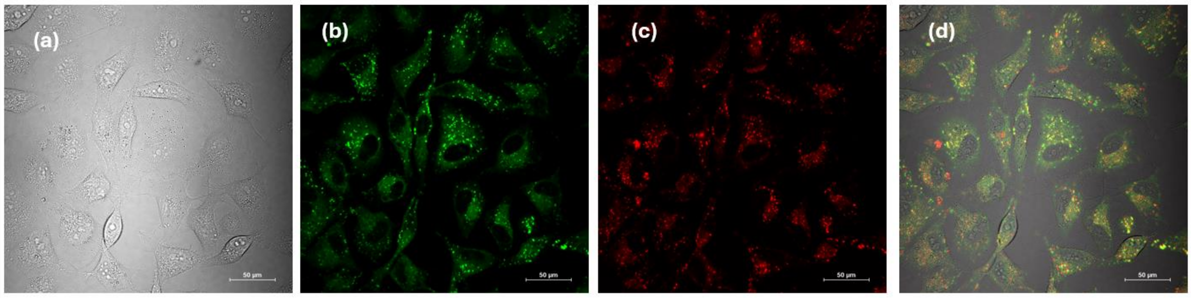

Figure S.79. Confocal laser-scanning microscopy of PC-3 cells incubated at 37 °C for 20 min with **GaL3** (100 nM in 1:99 DMSO:serum-free medium) and coincubated with Mitotracker-Red (1 µM in 1:99 DMSO:serum-free medium. (a) bright field channel; (b) green channel (λ_{em}=500-550 nm); (c) red channel (λ_{em}=570-750 nm); (d) overlay of the DIC-green-red channels. λ_{ex}=488 nm, Scale bar: 50 µm.

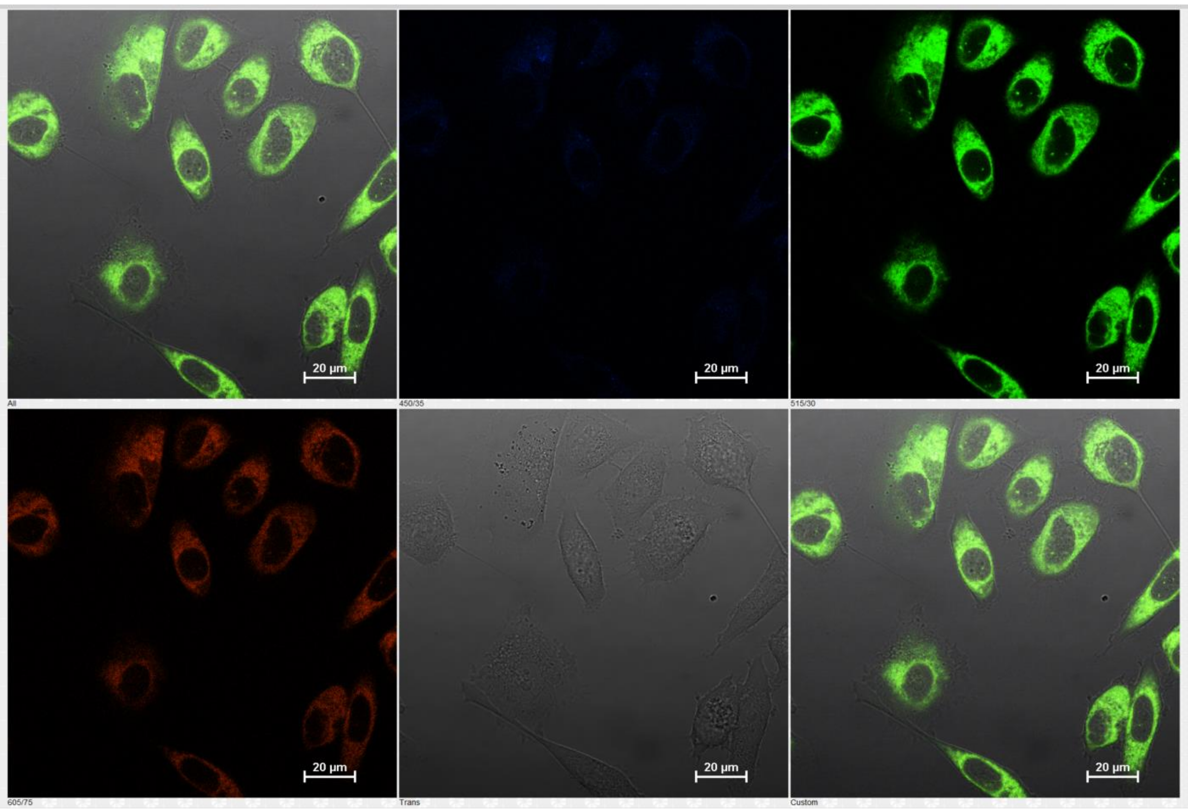

Figure S.80. Confocal laser-scanning microscopy of PC3 cells incubated at 37 °C for 15 min with **GaL4** (10 µM in 1:99 DMSO:serum-free medium). Clockwise: overlay of the DIC, blue-green-red channels, blue channel ($\lambda_{em\ max}$ =450 nm); green channel ($\lambda_{em\ max}$ =515 nm); red channel (λ_{em}= 605); DIC and custom overlay of green-red and DIC channels; λ_{ex}=405 nm. Scale bar: 20 µm.

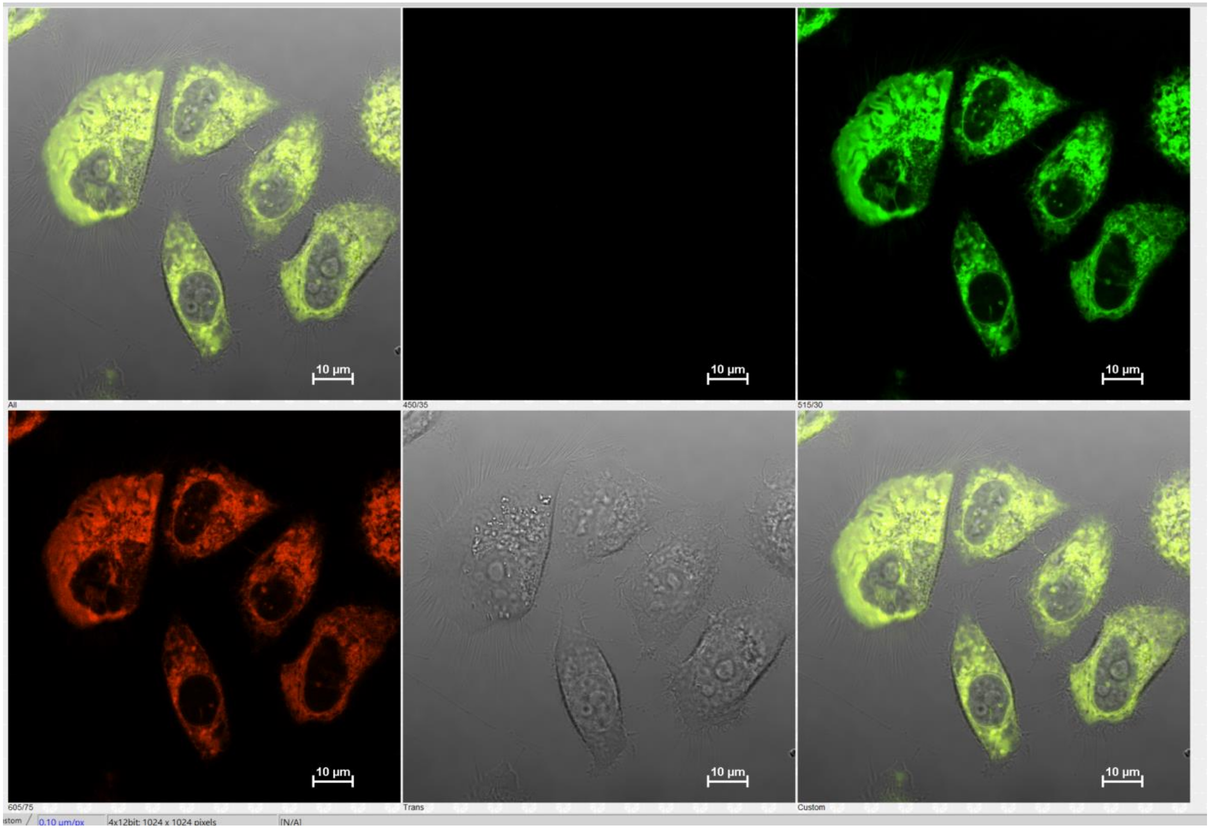

Figure S.81. Alternative field of view: Confocal laser-scanning microscopy of PC3 cells incubated at 37 °C for 15 min with **GaL4** (10 µM in 1:99 DMSO:serum-free medium). Clockwise: overlay of the DIC, blue-green-red channels, blue channel ($\lambda_{em\ max}$ =450 nm); green channel ($\lambda_{em\ max}$ =515 nm); red channel (λ_{em}= 605); DIC and custom overlay of green-red and DIC channels; λ_{ex}=488 nm. Scale bar: 20 µm.

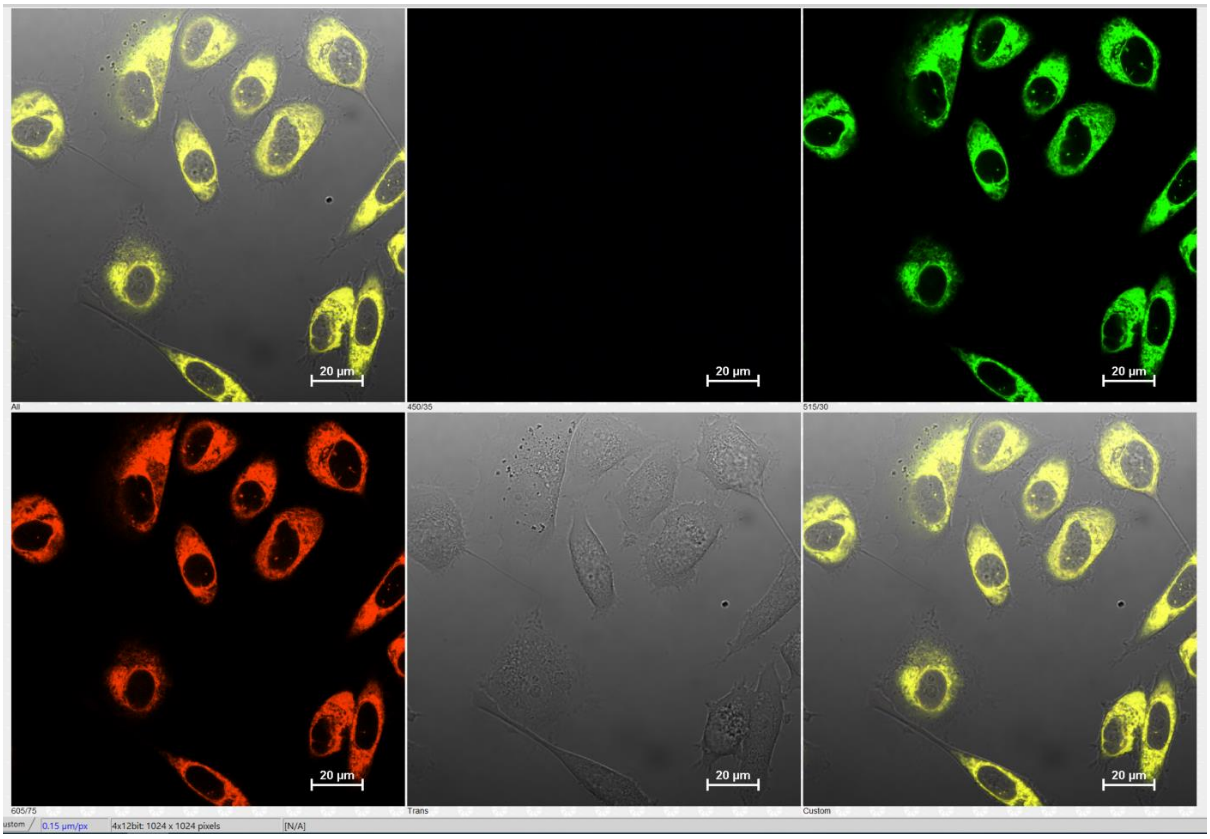

Figure S.82. Alternative field of view: Confocal laser-scanning microscopy of PC3 cells incubated at 37 °C for 15 min with **GaL4** (10 µM in 1:99 DMSO:serum-free medium). Clockwise: overlay of the DIC, blue-green-red channels, blue channel ($\lambda_{em\ max}$ =450 nm); green channel ($\lambda_{em\ max}$ =515 nm); red channel (λ_{em}= 605); DIC and custom overlay of green-red and DIC channels; λ_{ex}=488 nm. Scale bar: 20 µm.

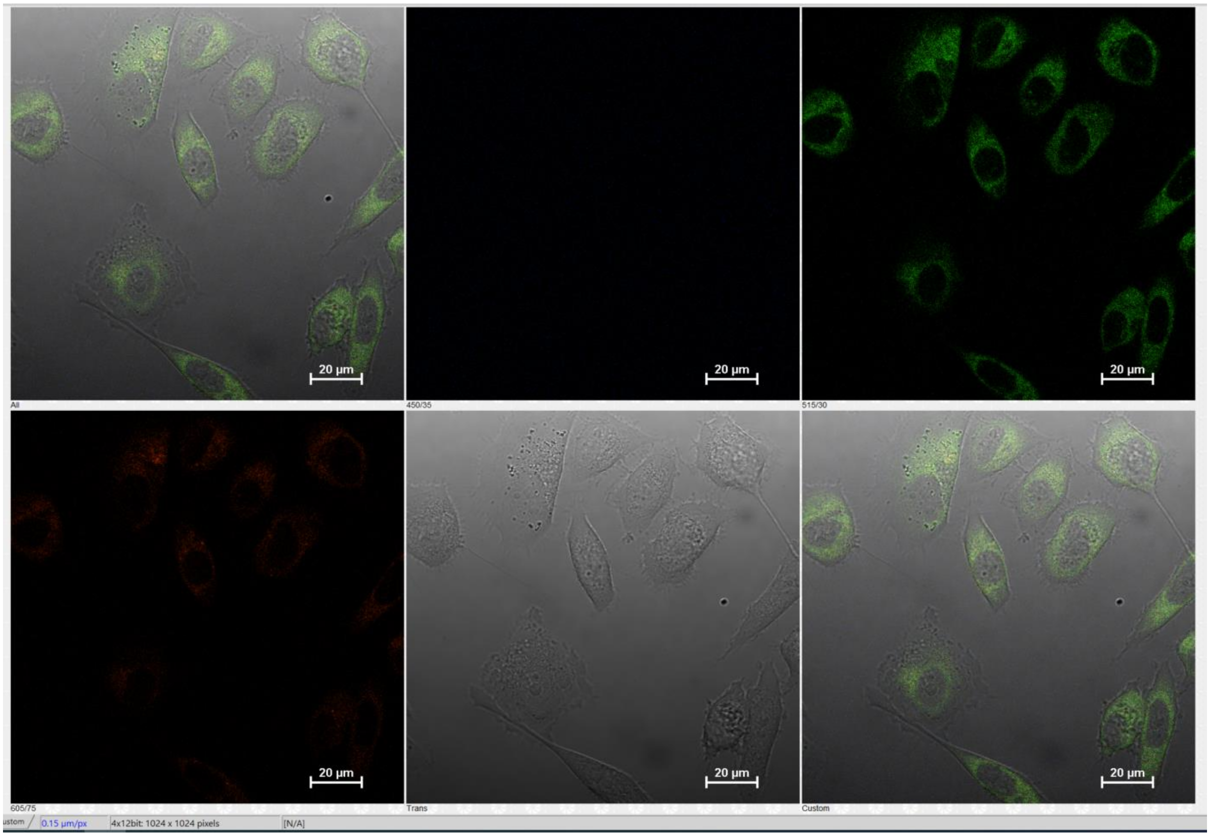

Figure S.83. Confocal laser-scanning microscopy of PC3 cells incubated at 37 °C for 15 min with **GaL4** (10 µM in 1:99 DMSO:serum-free medium). Clockwise: overlay of the DIC, blue-green-red channels, blue channel ($\lambda_{em\ max}$ =450 nm); green channel ($\lambda_{em\ max}$ =515 nm); red channel (λ_{em}= 605); DIC and custom overlay of green-red and DIC channels; λ_{ex}=543 nm. Scale bar: 20 µm.

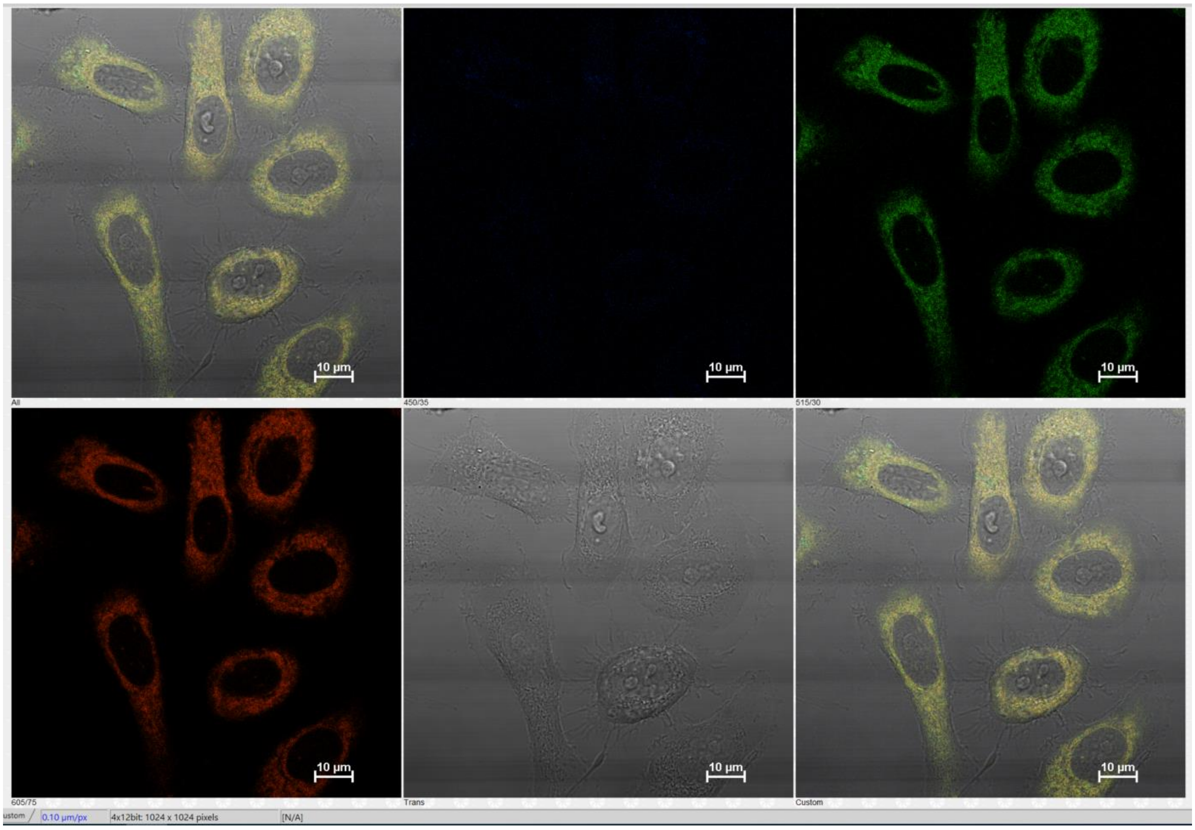

Figure S.84. Standard ER co-staining assays: Confocal laser-scanning microscopy of PC3 cells incubated at 37 °C for 15 min with **GaL4** (10 µM in 1:99 DMSO:serum-free medium). Clockwise: overlay of the DIC, blue-green-red channels, blue channel ($\lambda_{em\ max}$ =450 nm); green channel ($\lambda_{em\ max}$ =515 nm); red channel (λ_{em}= 605); DIC and custom overlay of green-red and DIC channels; λ_{ex}=405 nm. Scale bar: 10 µm.

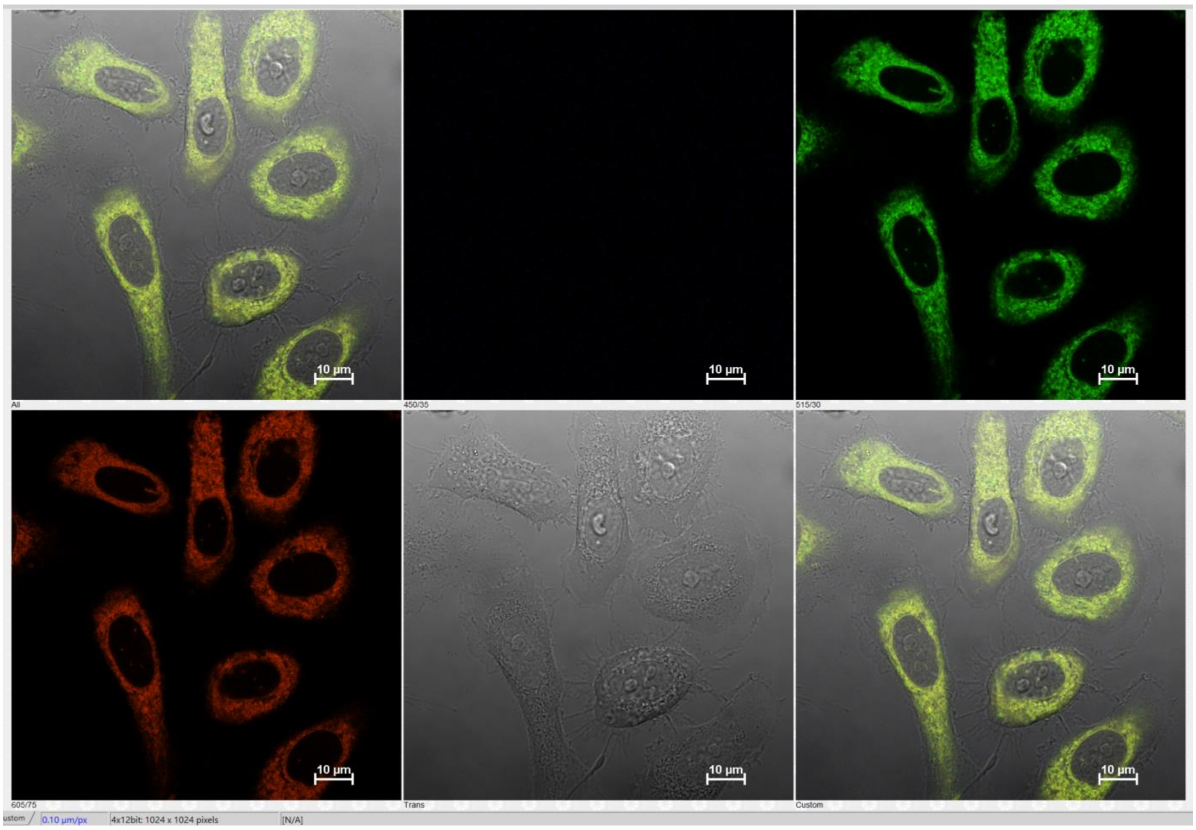

Figure S.85. Standard ER co-staining assays: Confocal laser-scanning microscopy of PC3 cells incubated at 37 °C for 15 min with **GaL4** (10 μM in 1:99 DMSO:serum-free medium). Clockwise: overlay of the DIC, blue-green-red channels, blue channel ($\lambda_{em\ max}$ =450 nm); green channel ($\lambda_{em\ max}$ =515 nm); red channel (λ_{em}= 605); DIC and custom overlay of green-red and DIC channels; λ_{ex}=488 nm. Scale bar: 10 μm.

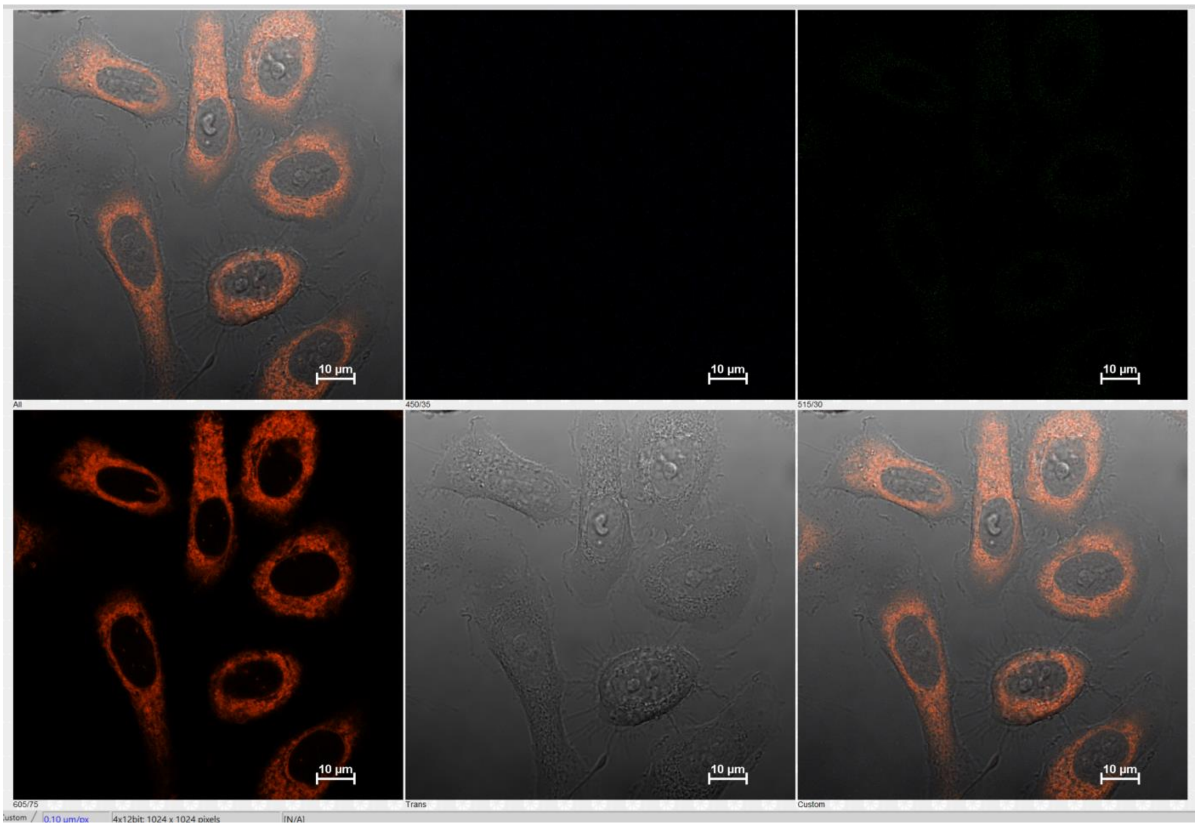

Figure S.86. Standard ER co-staining assays: Confocal laser-scanning microscopy of PC3 cells incubated at 37 °C for 15 min with **GaL4** (10 µM in 1:99 DMSO:serum-free medium). Clockwise: overlay of the DIC, blue-green-red channels, blue channel ($\lambda_{em\ max}$ =450 nm); green channel ($\lambda_{em\ max}$ =515 nm); red channel (λ_{em}= 605); DIC and custom overlay of green-red and DIC channels; λ_{ex}=543 nm. Scale bar: 10 µm.

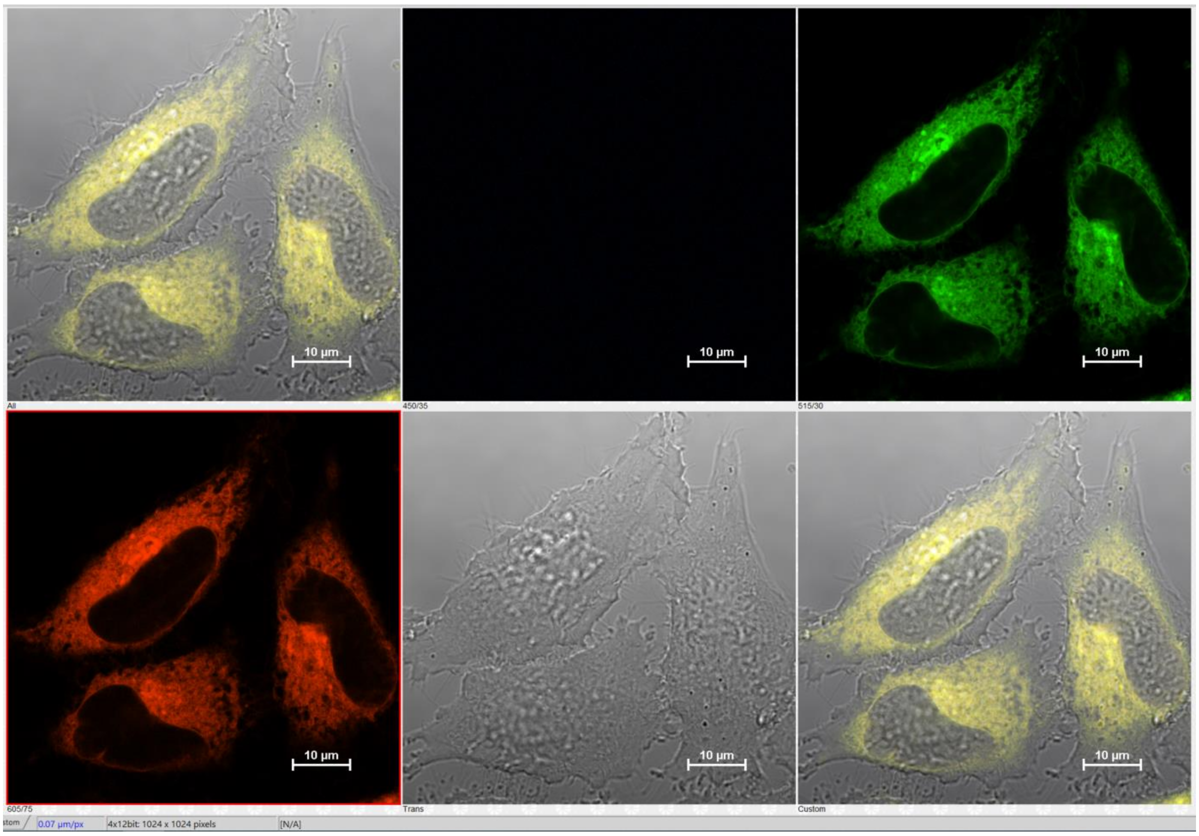

Figure S.87. Confocal laser-scanning microscopy of HeLa cells incubated at 37 °C for 15 min with **GaL4** (10 µM in 1:99 DMSO:serum-free medium). Clockwise: overlay of the DIC, blue-green-red channels, blue channel ($\lambda_{em\ max}$ =450 nm); green channel ($\lambda_{em\ max}$ =515 nm); red channel (λ_{em}= 605); DIC and custom overlay of green-red and DIC channels; λ_{ex}=543 nm. Scale bar: 10 µm.

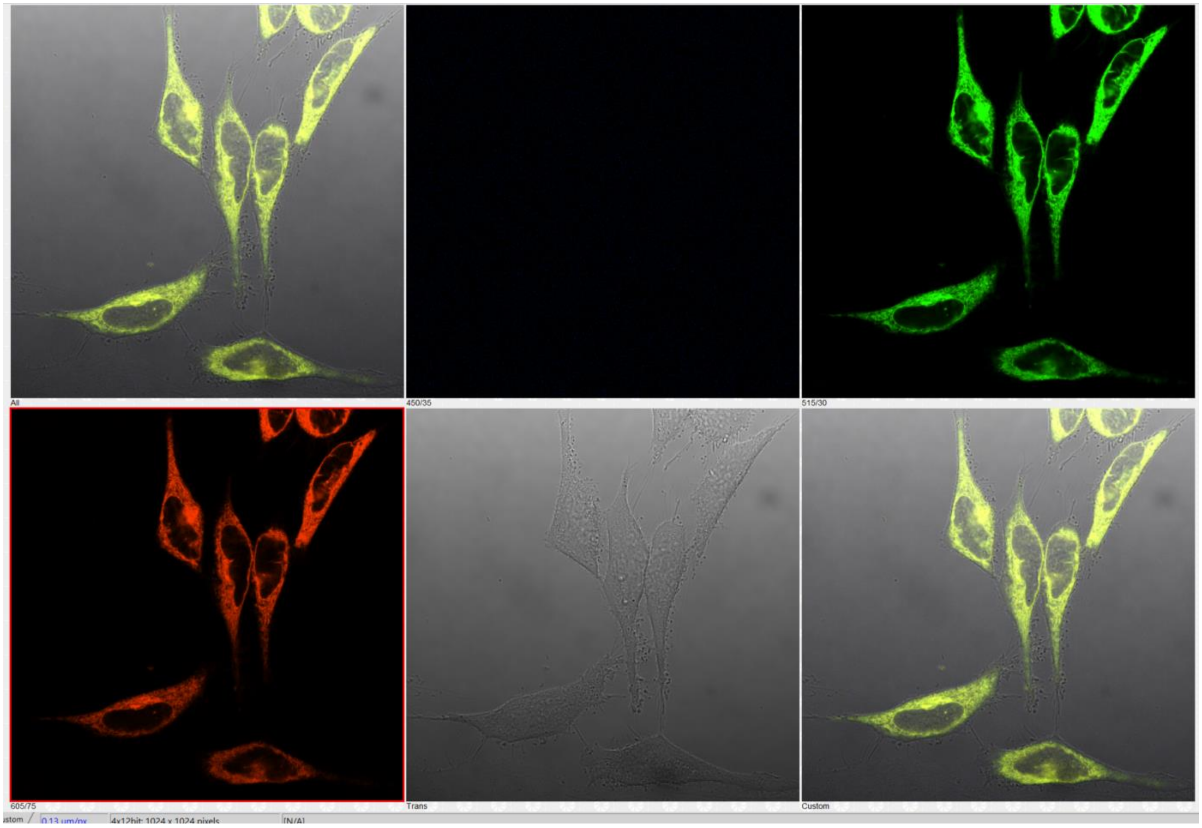

Figure S.88. Confocal laser-scanning microscopy of HeLa cells incubated at 37 °C for 15 min with **L4** (10 µM in 1:99 DMSO:serum-free medium). Clockwise: overlay of the DIC, blue-green-red channels, blue channel ($\lambda_{em\ max}$ =450 nm); green channel ($\lambda_{em\ max}$ =515 nm); red channel (λ_{em}= 605); DIC and custom overlay of green-red and DIC channels; λ_{ex}=488 nm. Field of view: 100 µm.

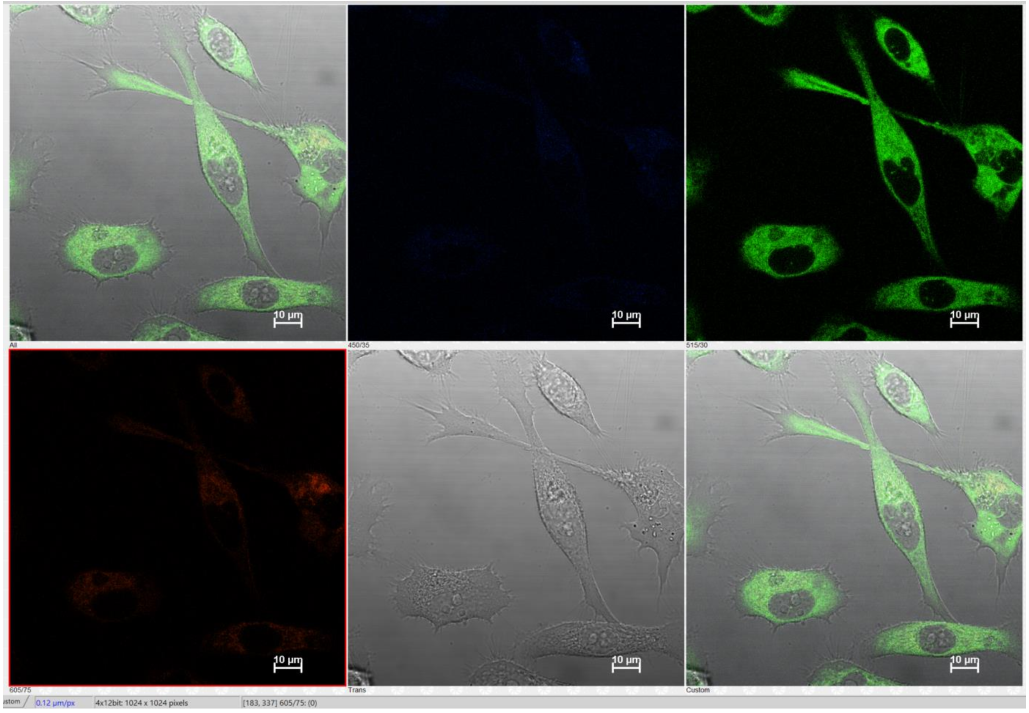

Figure S.89. Mitotracker co-staining assays: Confocal laser-scanning microscopy of PC3 cells incubated at 37 °C for 15 min with **GaL4** (10 µM in 1:99 DMSO:serum-free medium) and co-incubated with Mitotracker-Red (1 µM in 1:99 DMSO:serum-free medium). Clockwise: overlay of the DIC, blue-green-red channels, blue channel ($\lambda_{em\ max}$ =450 nm); green channel ($\lambda_{em\ max}$ =515 nm); red channel (λ_{em}= 605); DIC and custom overlay of green-red and DIC channels; λ_{ex}=405 nm. Scale bar: 10 µm.

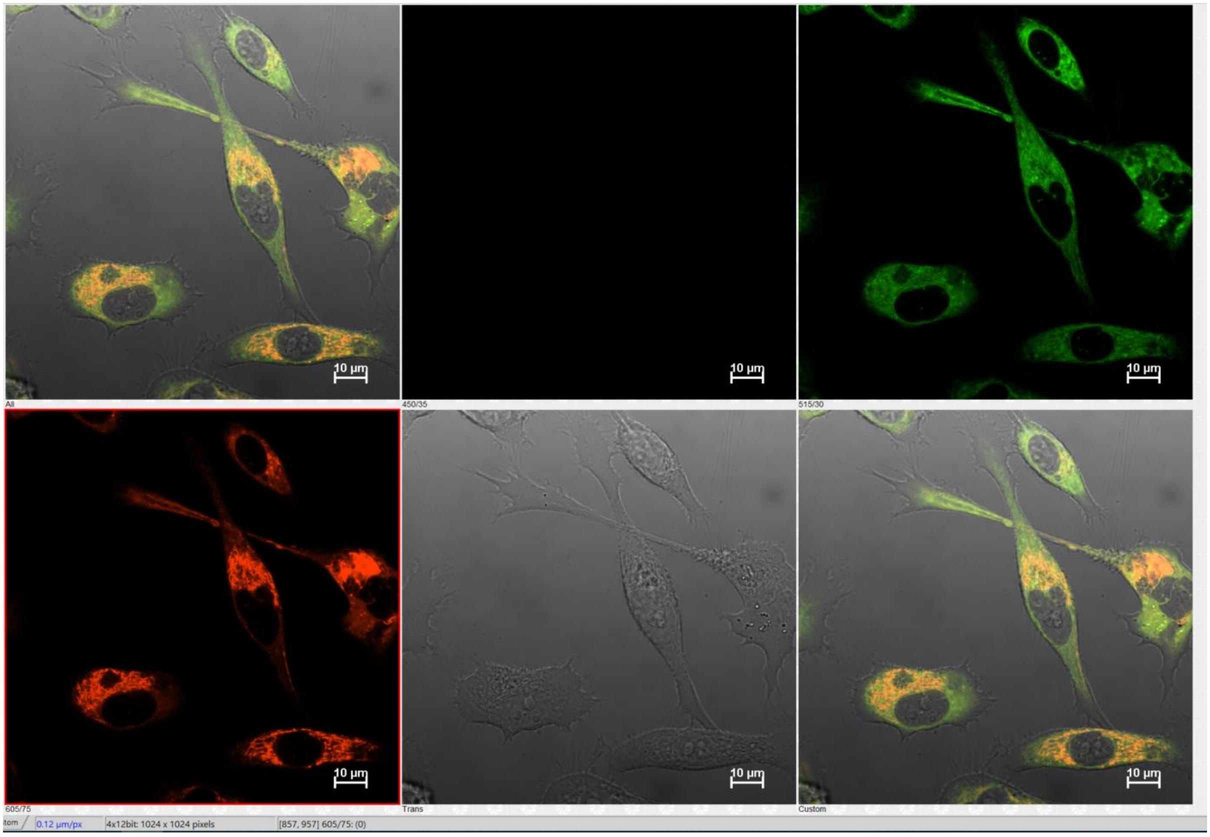

Figure S.90. Mitotracker co-staining assays: Confocal laser-scanning microscopy of PC3 cells incubated at 37 °C for 15 min with **GaL4** (10 µM in 1:99 DMSO:serum-free medium) and co-incubated with Mitotracker-Red (1 µM in 1:99 DMSO:serum-free medium). Clockwise: overlay of the DIC, blue-green-red channels, blue channel ($\lambda_{em\ max}$ =450 nm); green channel ($\lambda_{em\ max}$ =515 nm); red channel (λ_{em}= 605); DIC and custom overlay of green-red and DIC channels; λ_{ex}=488 nm. Scale bar: 10 µm.

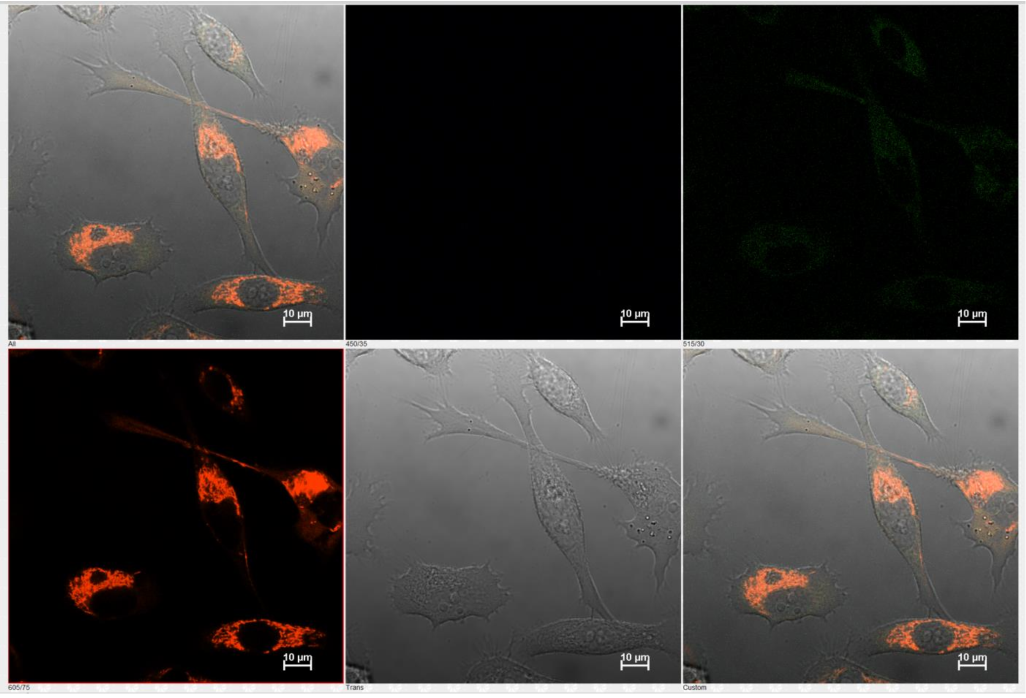

Figure S.91. Mitotracker co-staining assays: Confocal laser-scanning microscopy of PC3 cells incubated at 37 °C for 15 min with **GaL4** (10 µM in 1:99 DMSO:serum-free medium) and co-incubated with Mitotracker-Red (1 µM in 1:99 DMSO:serum-free medium). Clockwise: overlay of the DIC, blue-green-red channels, blue channel ($\lambda_{em\ max}$ =450 nm); green channel ($\lambda_{em\ max}$ =515 nm); red channel (λ_{em}= 605); DIC and custom overlay of green-red and DIC channels; λ_{ex}=548 nm. Scale bar: 10 µm.

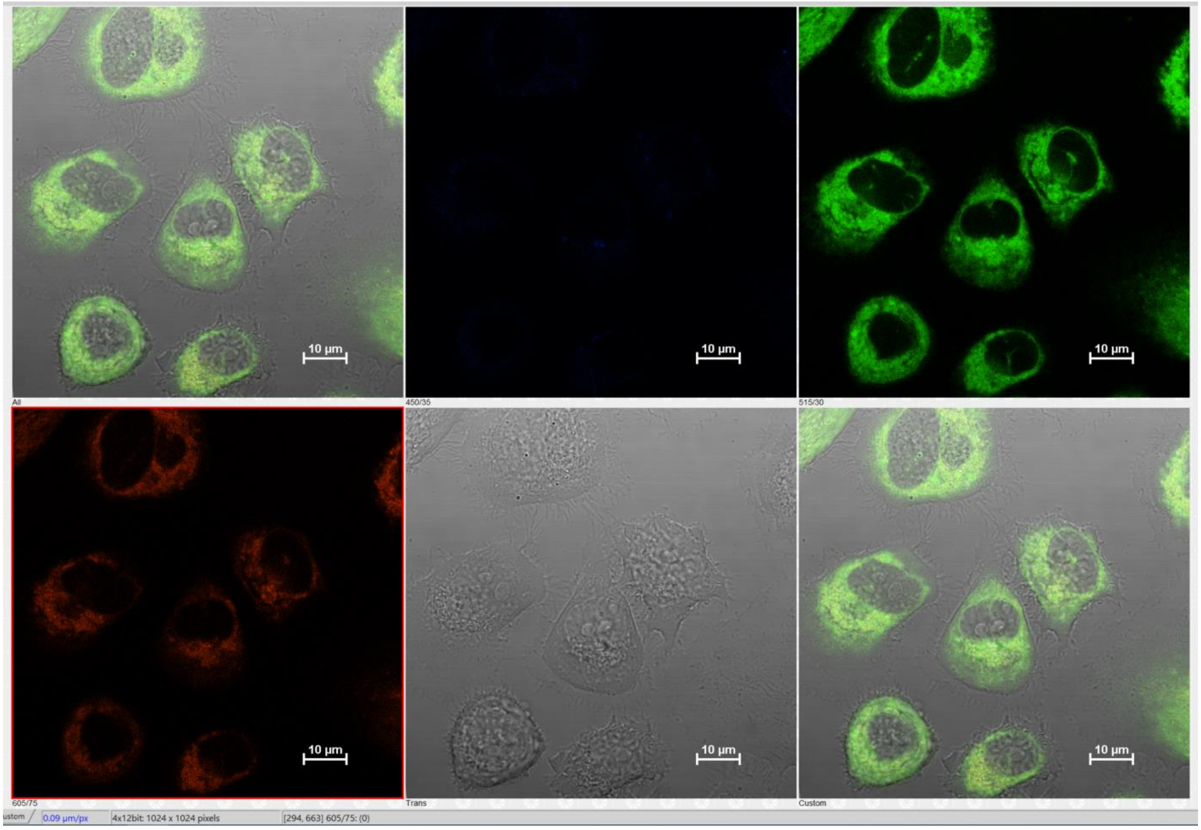

Figure S.92. Mitotracker co-staining assays: Confocal laser-scanning microscopy of PC3 cells incubated at 37 °C for 15 min with **InL4** (10 µM in 1:99 DMSO:serum-free medium) and co-incubated with Mitotracker-Red (1 µM in 1:99 DMSO:serum-free medium). Clockwise: overlay of the DIC, blue-green-red channels, blue channel ($\lambda_{em\ max}$ =450 nm); green channel ($\lambda_{em\ max}$ =515 nm); red channel (λ_{em}= 605); DIC and custom overlay of green-red and DIC channels; λ_{ex}=405 nm. Scale bar: 10 µm.

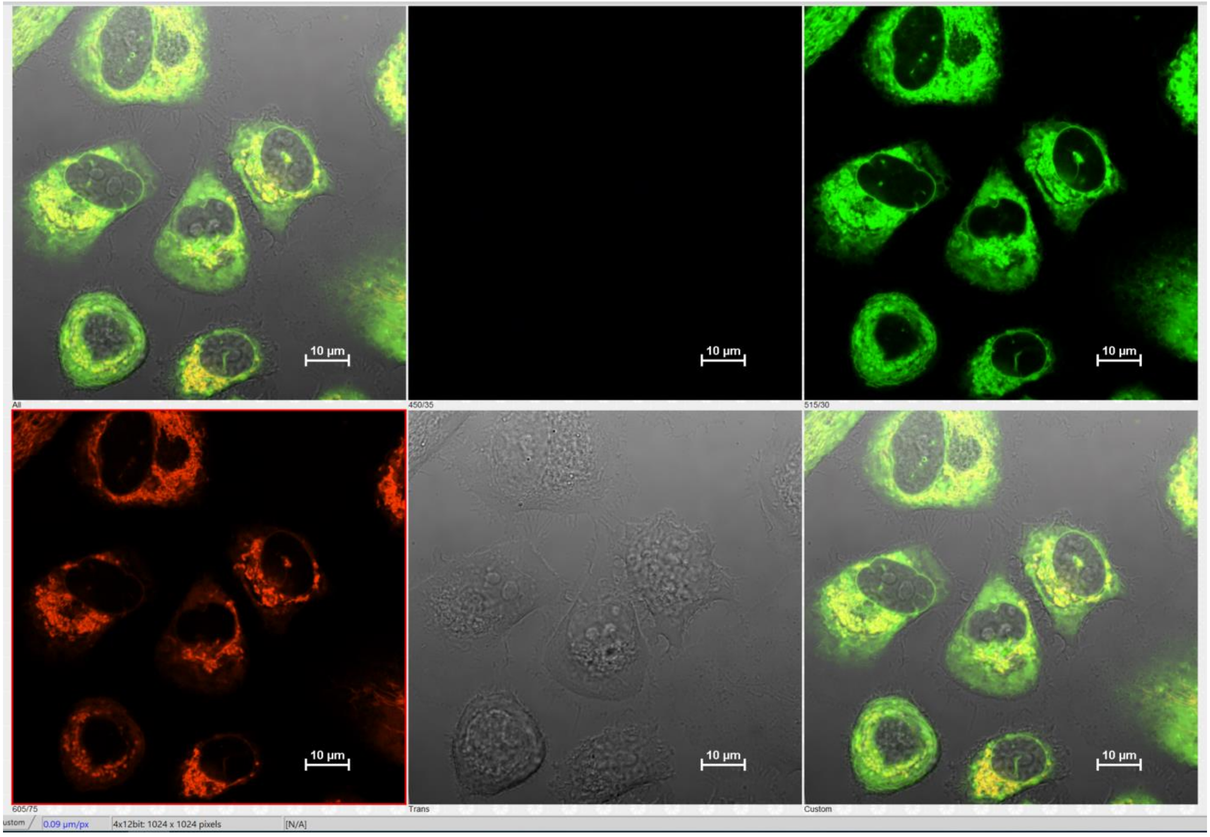

Figure S.93. Mitotracker co-staining assays: Confocal laser-scanning microscopy of PC3 cells incubated at 37 °C for 15 min with **GaL4** (10 µM in 1:99 DMSO:serum-free medium) and co-incubated with Mitotracker-Red (1 µM in 1:99 DMSO:serum-free medium). Clockwise: overlay of the DIC, blue-green-red channels, blue channel ($\lambda_{em\ max}$ =450 nm); green channel ($\lambda_{em\ max}$ =515 nm); red channel (λ_{em}= 605); DIC and custom overlay of green-red and DIC channels; λ_{ex}=488 nm. Scale bar: 10 µm.

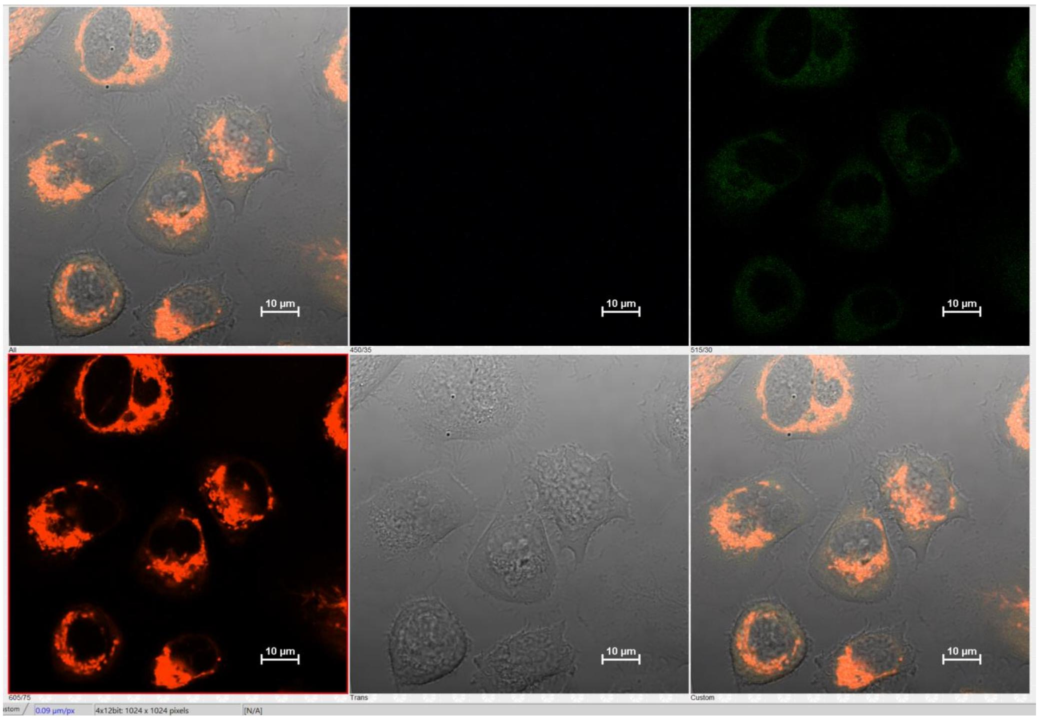

Figure S.94. Mitotracker co-staining assays: Confocal laser-scanning microscopy of PC3 cells incubated at 37 °C for 15 min with **GaL4** (10 µM in 1:99 DMSO:serum-free medium) and co-incubated with Mitotracker-Red (1 µM in 1:99 DMSO:serum-free medium). Clockwise: overlay of the DIC, blue-green-red channels, blue channel ($\lambda_{em\ max}$ =450 nm); green channel ($\lambda_{em\ max}$ =515 nm); red channel (λ_{em}= 605); DIC and custom overlay of green-red and DIC channels; λ_{ex}=548 nm. Scale bar: 10 µm.

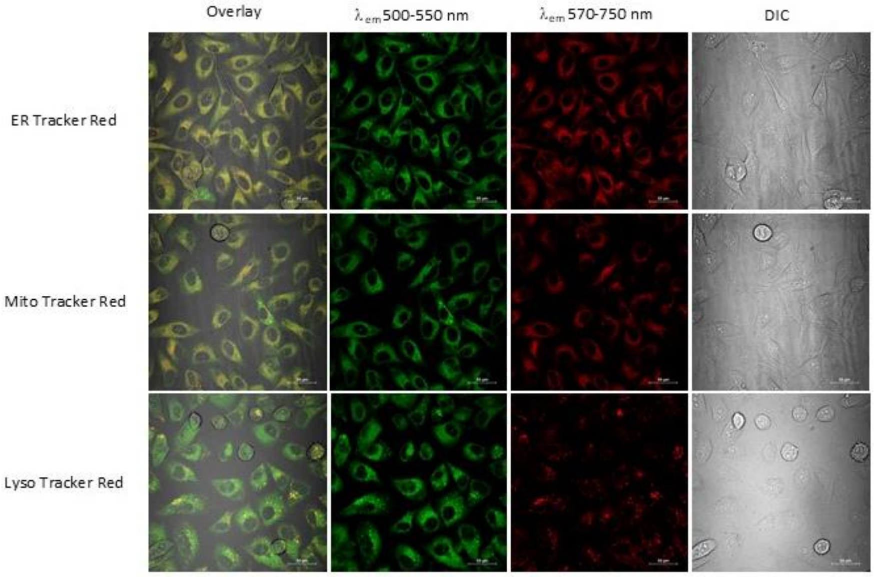

(a)

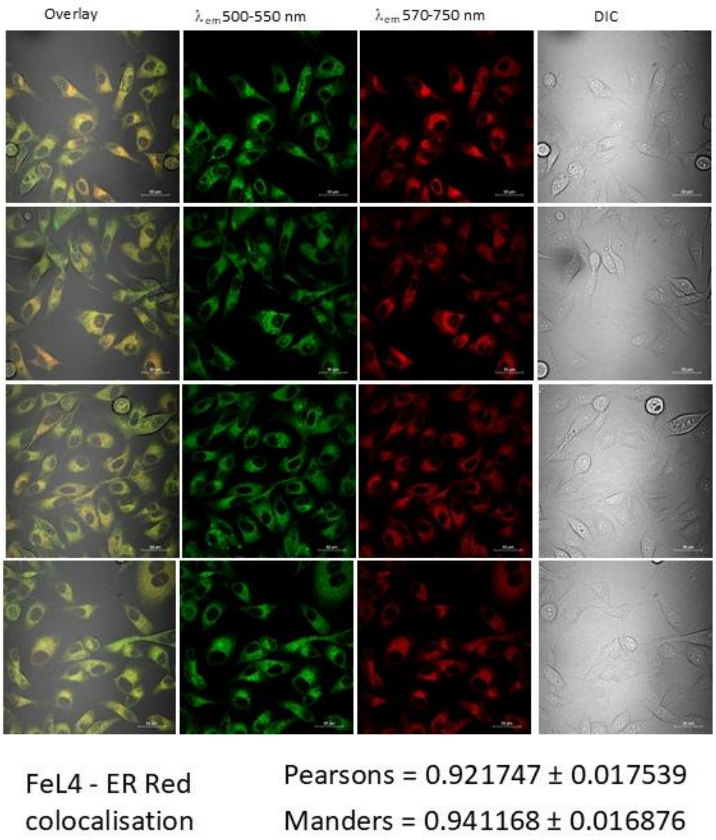
Overlay
λ_{em} 500-550 nm
λ_{em} 570-750 nm
DIC
FeL4 - ER Red colocalisation
Pearsons = 0.921747 ± 0.017539
Manders = 0.941168 ± 0.016876

(b)

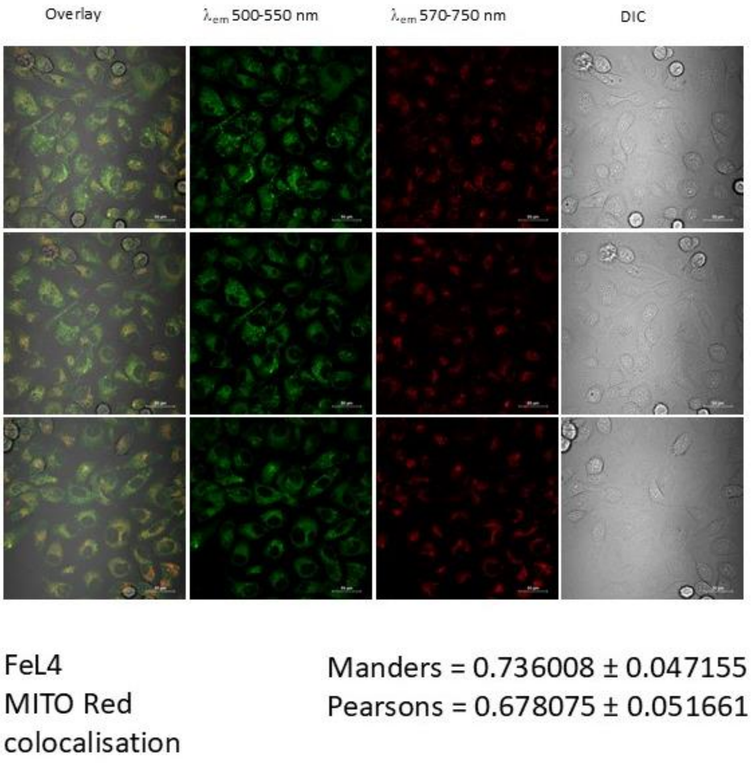
Overlay
λ_{em} 500-550 nm
λ_{em} 570-750 nm
DIC
FeL4
MITO Red
colocalisation
Manders = 0.736008 ± 0.047155
Pearsons = 0.678075 ± 0.051661

(c)

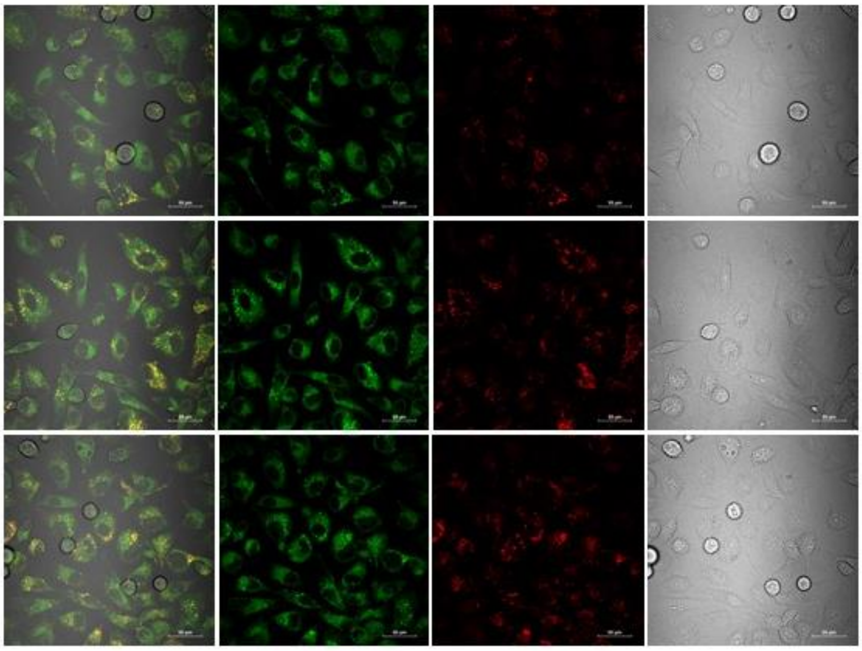

FeL4 - Lysotracker Red colocalisation

Pearsons = 0.556121 ± 0.048933

Manders = 0.741926 ± 0.036034

(d)

Figure S.95. Overview of selected co-staining assays (a) **HL4:** Confocal laser-scanning microscopy of PC3 cells incubated at 37 °C for 15 min (1 μM in 1:99 DMSO:serum-free medium) and co-incubated with ER-Red (1 μM in 1:99 DMSO:serum-free medium), top row, Mitotracker Red (middle row, 1 μM in 1:99 DMSO:serum-free medium) and Lyso tracker red (bottom row, 1 μM in 1:99 DMSO:serum-free medium). Images show, in each row, overlays of the DIC, blue-green-red channels, green channel; red channel; DIC channels; λ_{ex}=488 nm for emission 500-550 nm and 561 for emission 570-750 nm. Scale bar: 10 μm. (b)-(d) **FeL4:** Confocal laser-scanning microscopy of PC3 cells incubated at 37 °C for 15 min (1 μM in 1:99 DMSO:serum-free medium) and co-incubated with

(b) ER Red (1 μM in 1:99 DMSO:serum-free medium),

(c) Mitotracker Red (1 μM in 1:99 DMSO:serum-free medium)

(d) Lysotracker Red (1 μM in 1:99 DMSO:serum-free medium). Images show, in set, overlays of the DIC, blue-green-red channels, green channel; red channel; DIC channels; λ_{ex}=488 nm for emission in green channel (500-550 nm) and 561 n for emission in red channel (570-750 nm). Scale bar: 10 μm.

Table S.7. Overview of the Manders' correlation parameters of selected BODIPY conjugates extrapolated by scatterplot analysis using the software Nikon Elements-AR Analysis 4.30.02.
*Pearson's coefficient was also estimated indicating mitochondria colocalization for **GaL4** 0.815 ± 0.132 and **InL4** 0.82 ± 0.029; ER colocalization for **GaL4** 0.92 ± 0.008 and for **InL4** 0.92 ± 0.014.

**Compound**	**Endoplasmic Reticulum (ER)**	**Mitochondria**	**Lysosome**
HL3	0.489 ± 0.035	0.497 ± 0.047	0.629 ± 0.047
HL4	0.934 ± 0.021	0.852 ± 0.030	0.497 ± 0.050
GaL3	0.676 ± 0.045	0.589 ± 0.058	0.470 ± 0.057
InL3	0.539 ± 0.050	0.530 ± 0.043	0.596 ± 0.048
GaL4*	0.985 ± 0.010	0.970 ± 0.016	ns
InL4*	0.990 ± 0.010	0.990 ± 0.010	ns
FeL3	0.923 ± 0.024	0.773 ± 0.038	0.399 ± 0.050
FeL4	0.922 ± 0.018	0.678 ± 0.051	0.556 ± 0.049

12. Selected FLIM Imaging micrographs

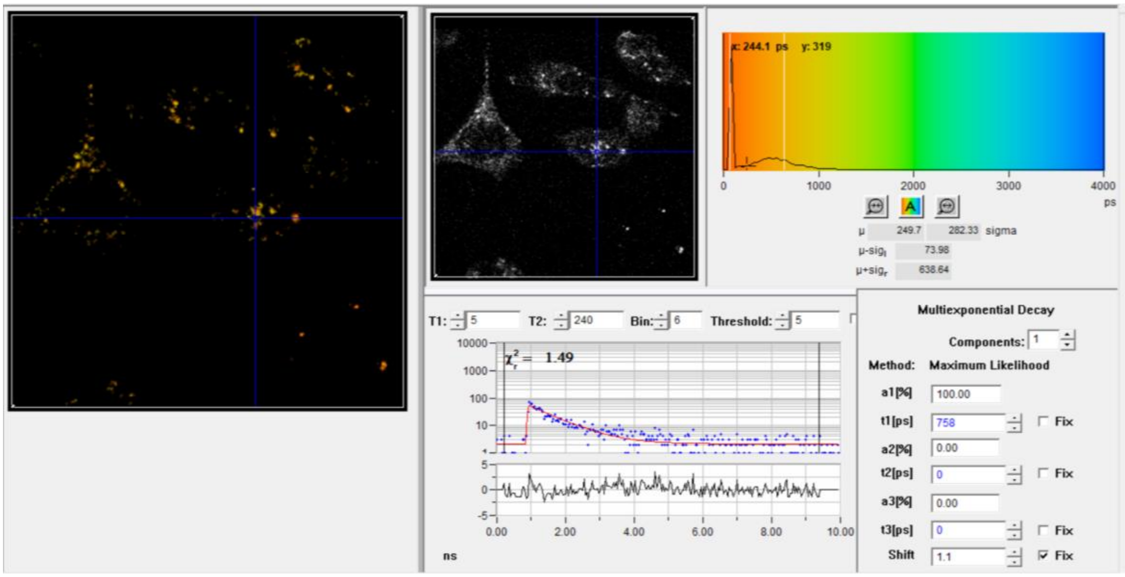

Figure S.96. 2-photon FLIM map and *in vitro* fluorescence lifetime distribution histograms of control group PC-3 treated with 1% DMSO for 20 min, with an excitation laser at 910 nm. Field of view (100 µm).

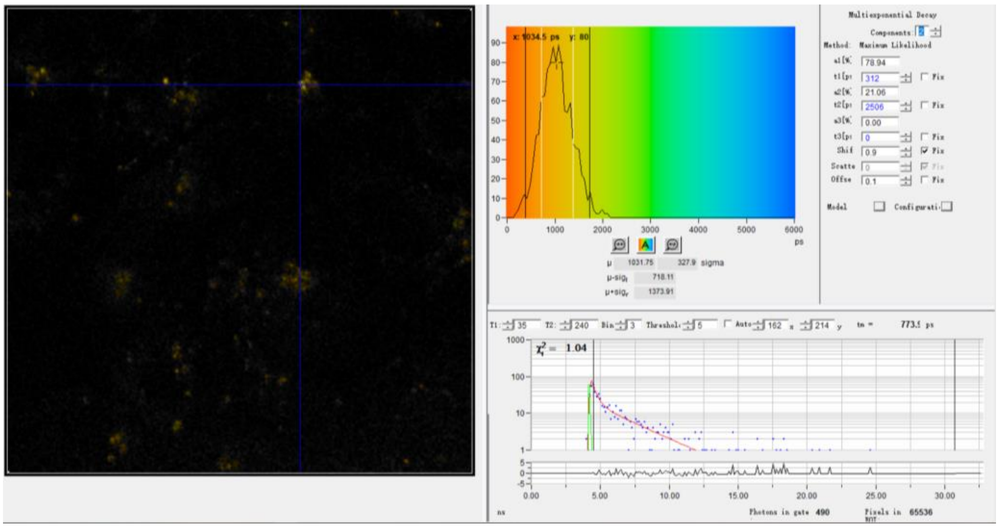

Figure S.97. Single-photon FLIM map and *in vitro* fluorescence lifetime distribution histograms of control group PC-3 treated with 1% DMSO for 20 min, with an excitation laser at 488 nm. Field of view (100 µm).

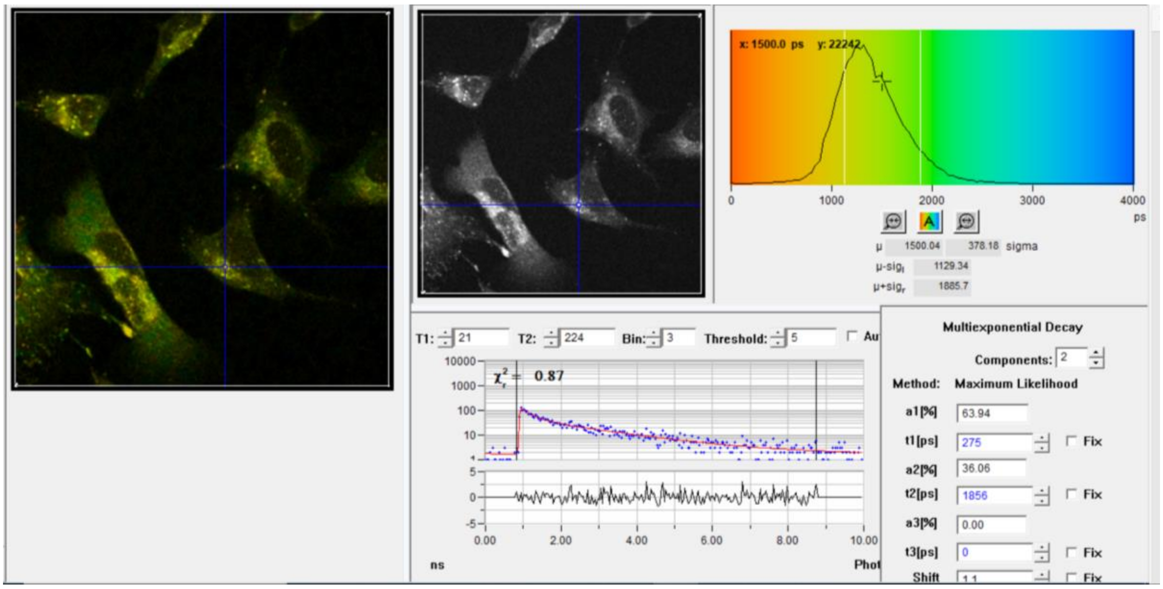

Figure S.98. 2-photon FLIM map and *in vitro* fluorescence lifetime distribution histograms of control group PC-3 treated with 1% PBS for 20 min, with an excitation laser at 810 nm (4.5 mW laser power). Images show how extensive autofluorescence may be caused in presence of PBS. Field of view (100 μm).

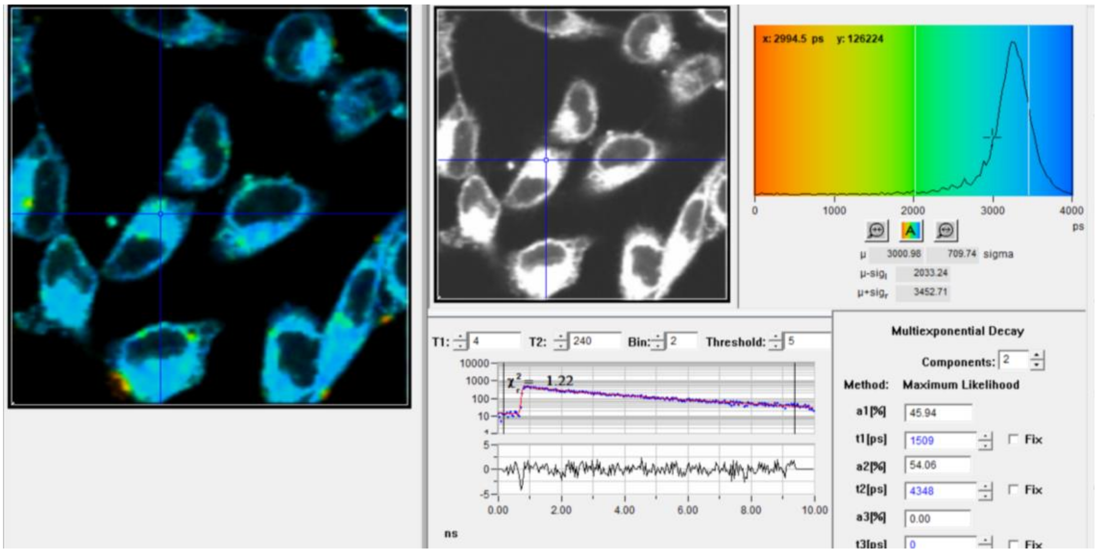

Figure S.99. Two-photon FLIM maps and *in vitro* fluorescence lifetime distribution histograms of HeLa cells treated 1 μM (contains 1% v/v DMSO) of BODIPY-COOH compound for 20 min, with an excitation laser at 910 nm. Field of view (100 μm).

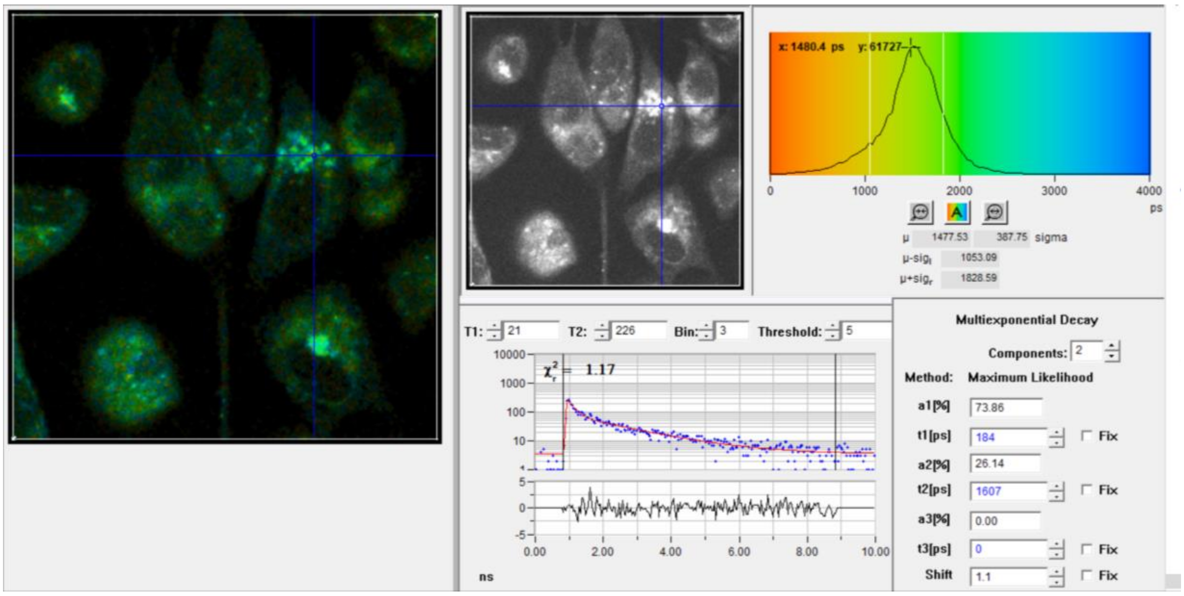

Figure S.100. Two-photon FLIM maps and *in vitro* fluorescence lifetime distribution histograms of PC-3 cells treated 1 μM (contains 1% v/v DMSO) of Mitotracker Red for 20 min, with an excitation laser at 910 nm, 5 mW laser power). Field of view (100 μm).

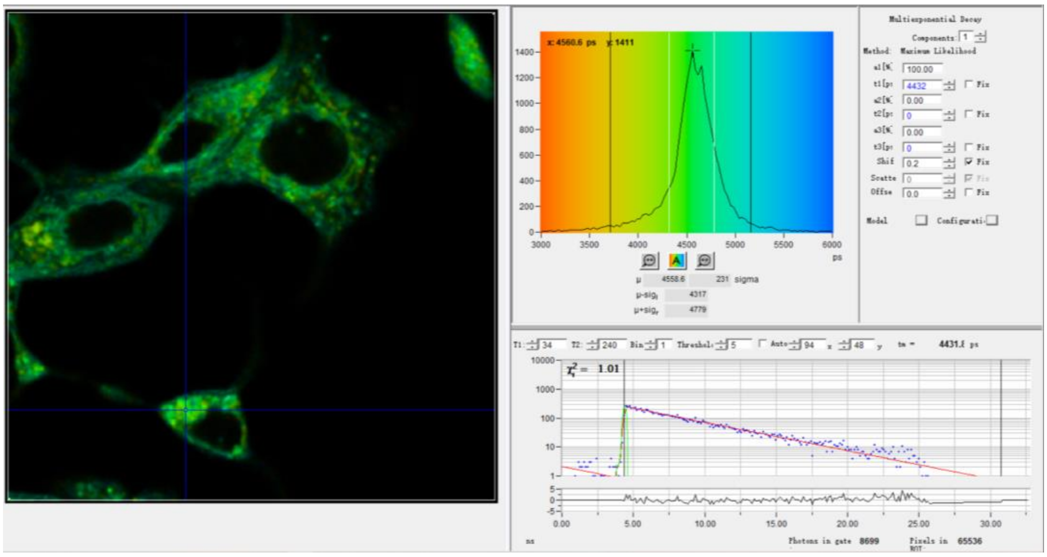

Figure S.101. Single-photon FLIM map and *in vitro* fluorescence lifetime distribution histograms of PC-3 treated 1 μM (contains 1% v/v DMSO) of compound **HL4** for 20 min, with an excitation laser at 488 nm. Field of view (100 μm).

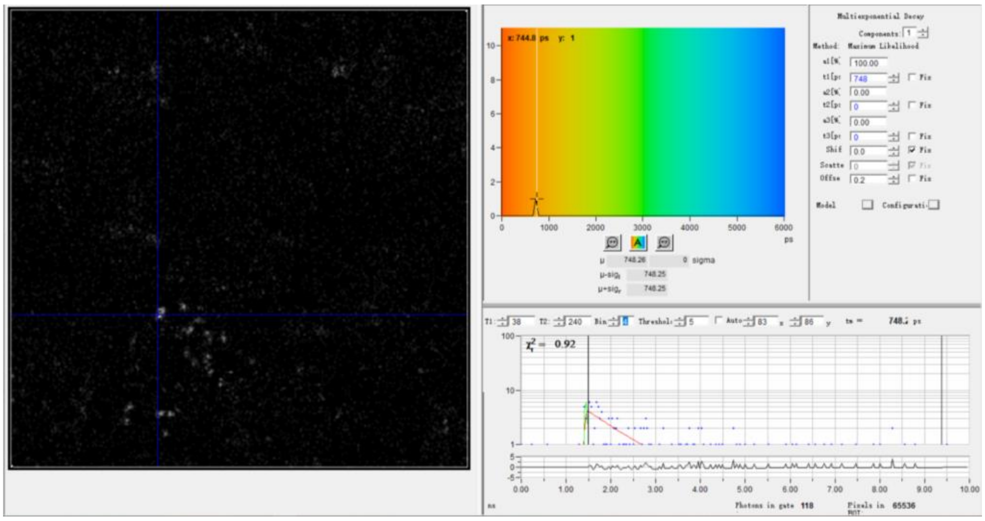

Figure S.102. Two-photon FLIM map and *in vitro* fluorescence lifetime distribution histograms of control group PC-3 treated with 1% DMSO for 20 min, with an excitation laser at 1010 nm. Field of view (100 μm).

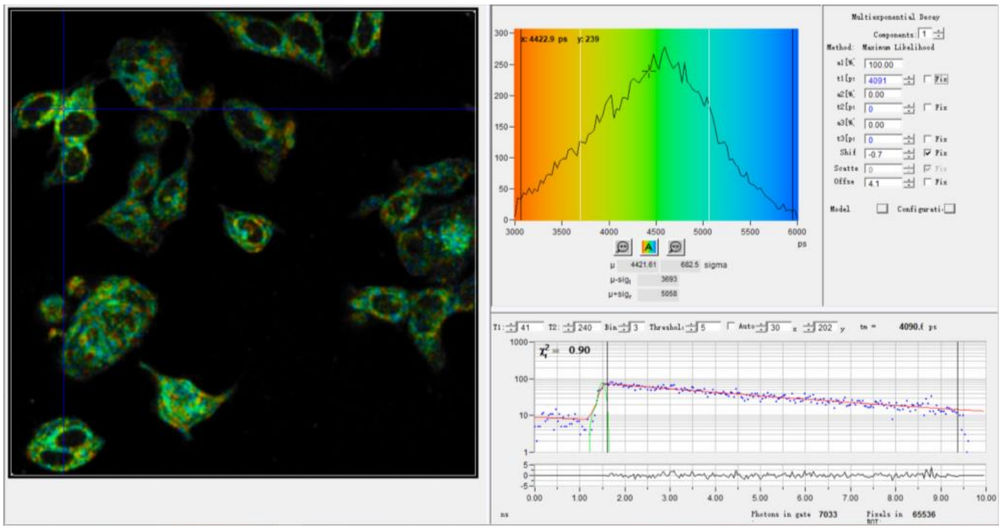

Figure S.103. Two-photon FLIM maps and *in vitro* fluorescence lifetime distribution histograms of PC-3 treated 1 μM (contains 1% v/v DMSO) of compound **HL4** for 20 min, with an excitation laser at 1010 nm. Field of view (100 μm).

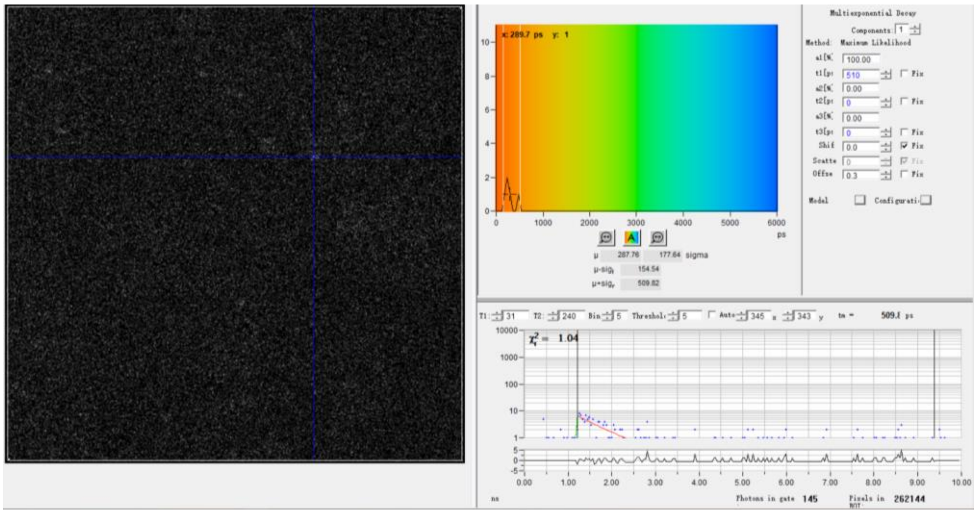

Figure S.104. Single-photon FLIM maps and *in vitro* fluorescence lifetime distribution histograms of control group PC-3 treated with 1% DMSO for 20 min, with an excitation laser at 640 nm. Field of view (100 μm).

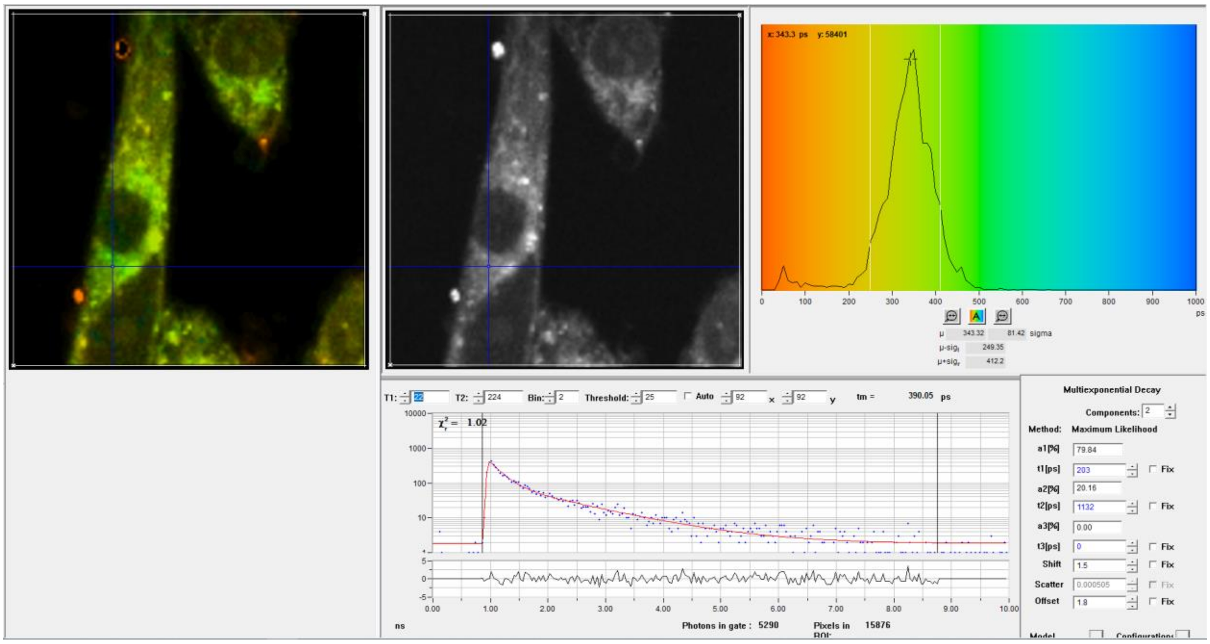

Figure S.105. (a) Two-photon FLIM map, intensity image and *in vitro* fluorescence lifetime distribution histograms of PC-3 treated 50 μM (containing 1% v/v DMSO) of compound **InL2** for 20 min, with an excitation laser at 910 nm. Field of view 100 μm. Bottom row: 2P TCSPC in random spot and corresponding parameters.

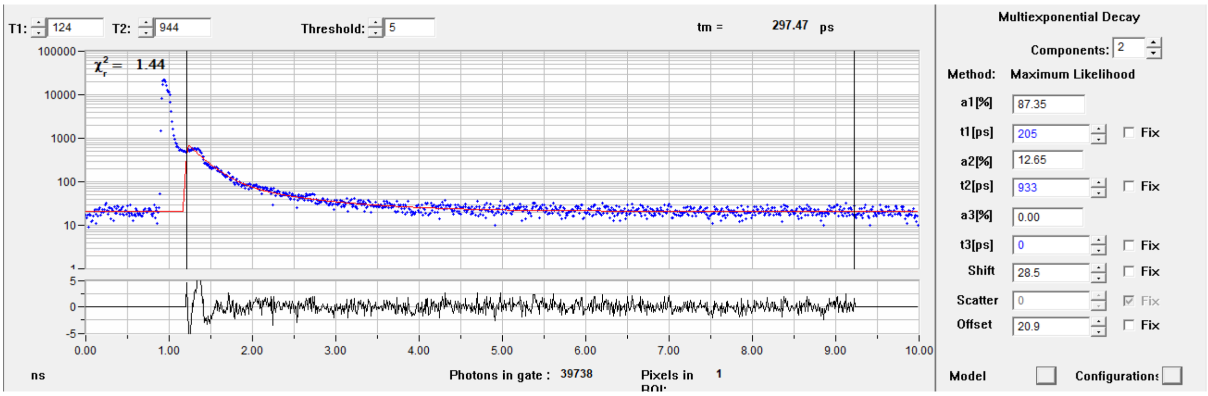

Figure S.106. 2P TCSPC in solution phase for **InL2** compound, 1 mM conc DMSO, and corresponded fitted parameters after subtraction of instrument response, 910 nm.

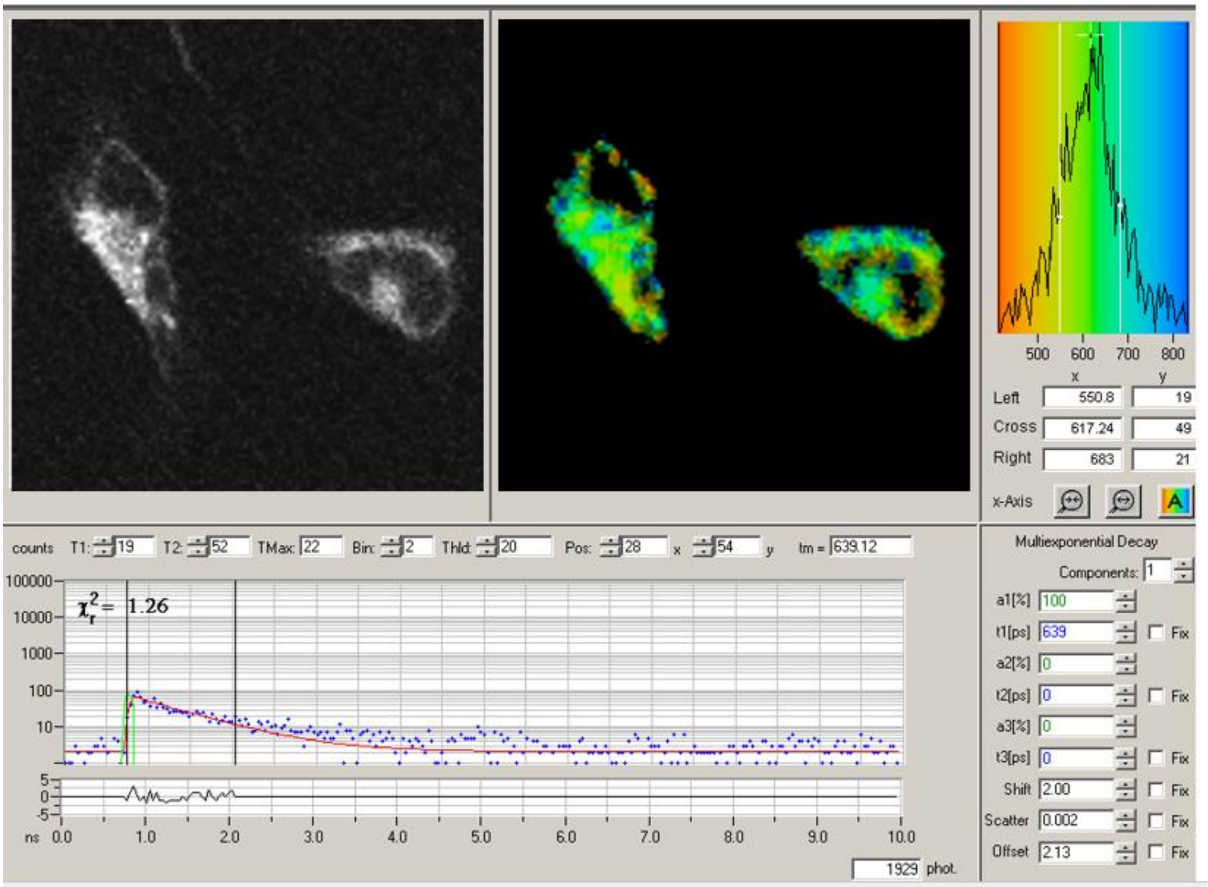

Figure S.107. (a) Two-photon FLIM map, intensity image and *in vitro* fluorescence lifetime distribution histograms of PC-3 treated 50 μM (containing 1% v/v DMSO) of compound **GaL2** for 20 min, with an excitation laser at 910 nm. Field of view 100 μm. Bottom row: 2P TCSPC in random spot and corresponding parameters.

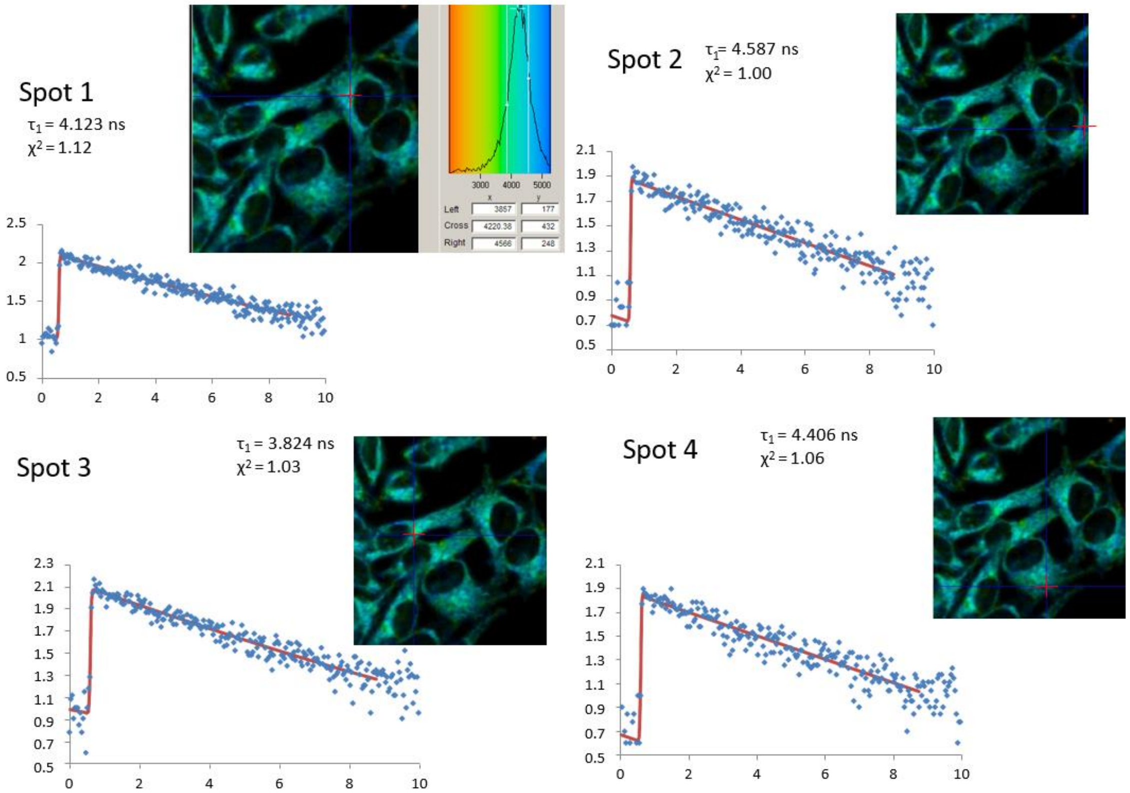

Figure S.108. Two-photon FLIM map, intensity image and *in vitro* fluorescence lifetime distribution histograms of HeLa cells treated 1 μM (containing 1% v/v DMSO) of compound **HL3** for 15 min, 37 ºC with an excitation laser at 910 nm. Field of view 100 μm.

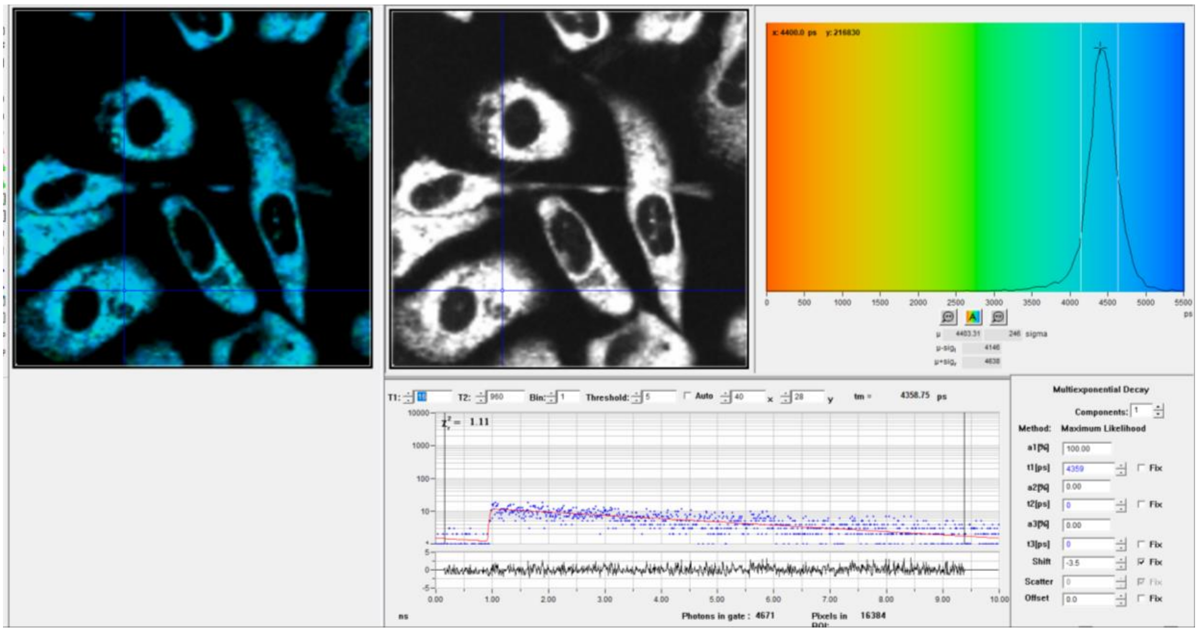

Figure S.109. Two-photon FLIM map, intensity image and *in vitro* fluorescence lifetime distribution histograms of PC-3 cells treated 1 µM (containing 1% v/v DMSO) of compound **HL4** for 15 min, 37 ºC with an excitation laser at 910 nm. Field of view 100 µm.

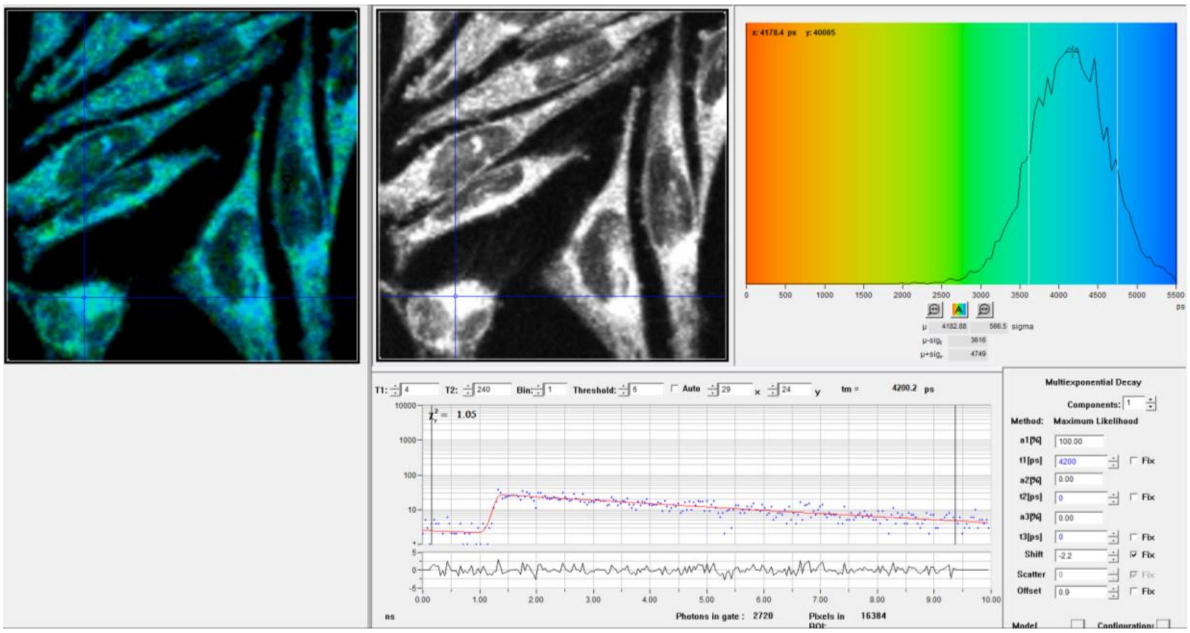

Figure S.110. Two-photon FLIM map, intensity image and *in vitro* fluorescence lifetime distribution histograms of HeLa cells treated 1 µM (containing 1% v/v DMSO) of compound **HL4** for 15 min, 37 ºC with an excitation laser at 910 nm. Field of view 100 µm.

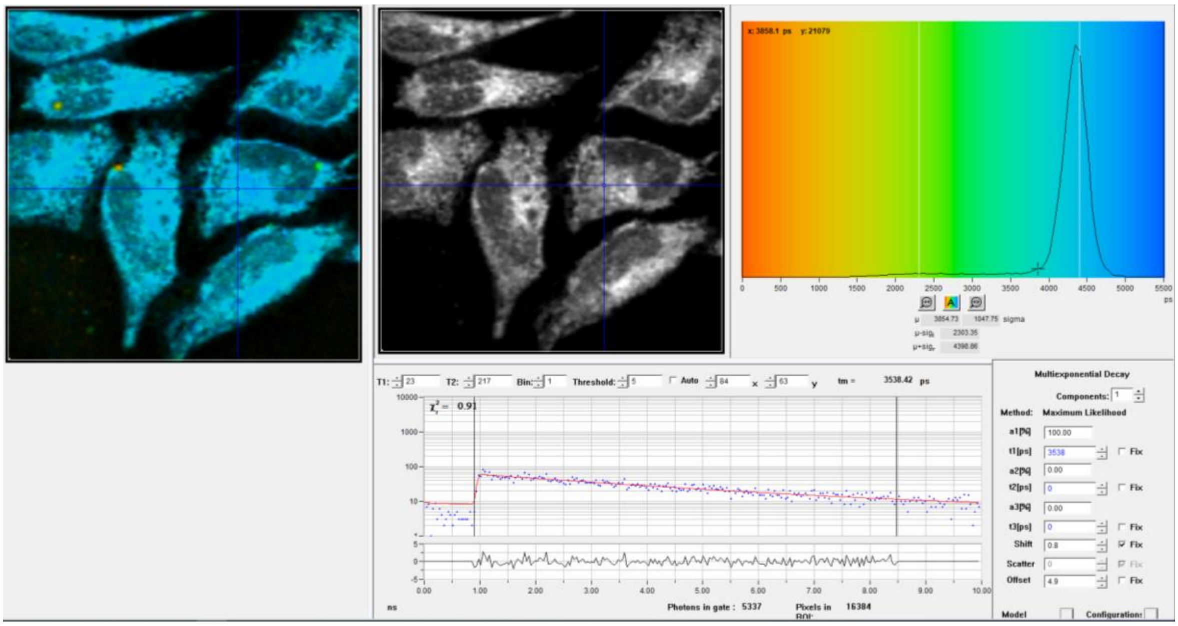

Figure S.111. Two-photon FLIM map, intensity image and *in vitro* fluorescence lifetime distribution histograms of living HeLa cells treated 1 μM (containing 1% v/v DMSO) of compound **GaL4** for 15 min, 37 °C with an excitation laser at 910 nm. Field of view 100 μm.

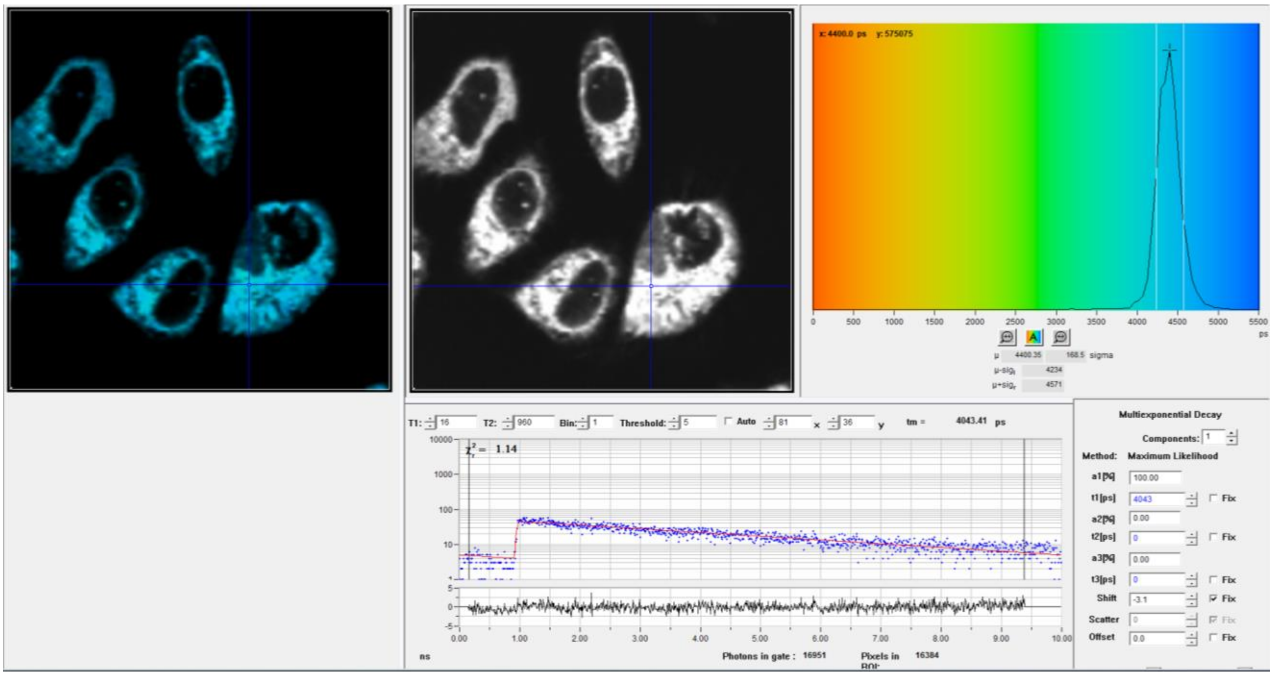

Figure S.112. Two-photon FLIM maps intensity image and *in vitro* fluorescence lifetime distribution histograms of living PC-3 cells treated 1 μM (containing 1% v/v DMSO) of compound **GaL4** for 15 min, 37 °C with an excitation laser at 910 nm. Field of view 100 μm.

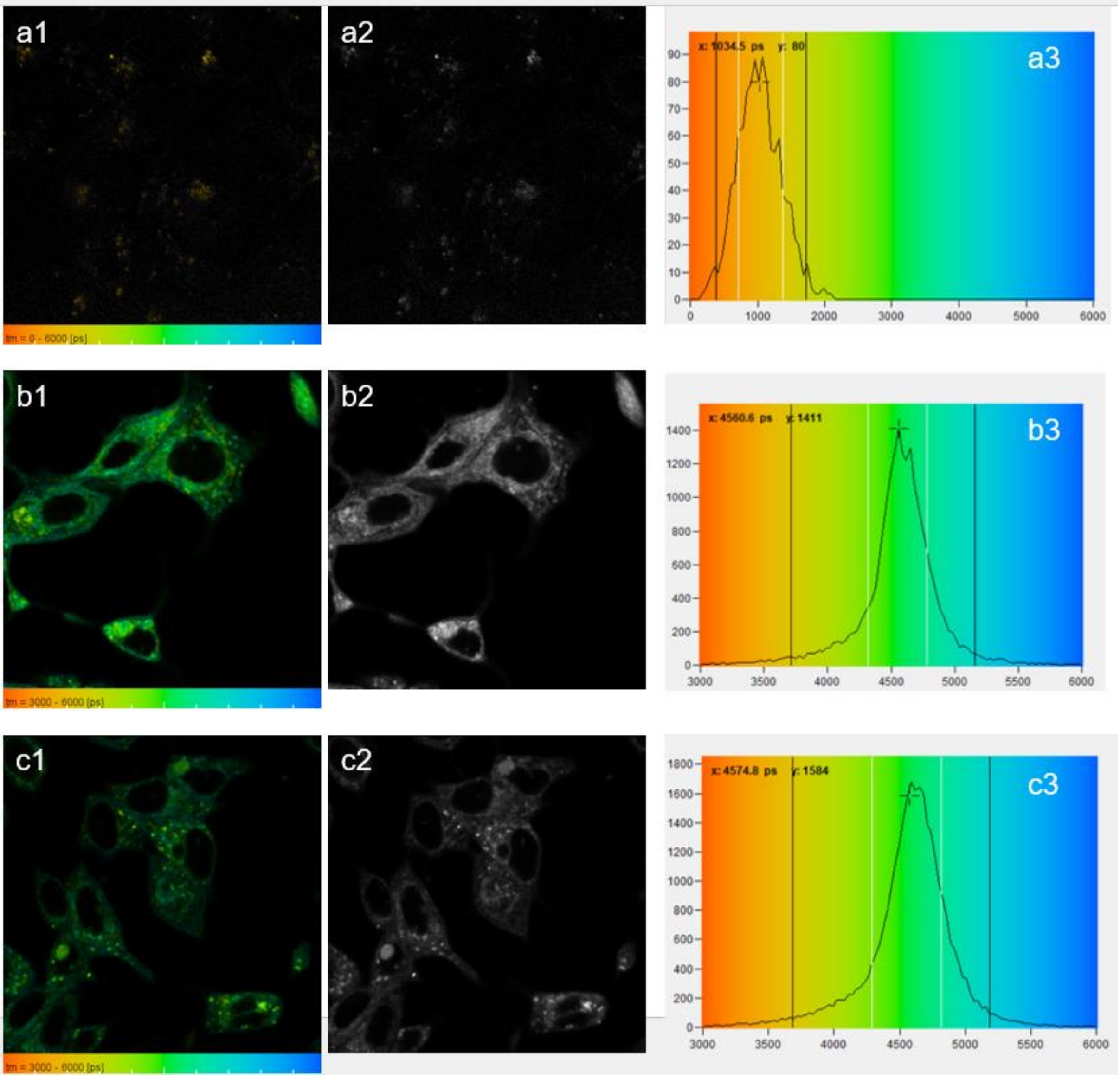

Figure S113. 1-photon FLIM maps, intensity images and *in vitro* fluorescence lifetime distribution histograms (left to right) of PC-3 cells treated with 1 μM of **HL4** (b2-b3) and corresponding **FeL4** (c1-c3) complex, where (a1-a3) represent the micrographs for the control group (1% DMSO). Incubation was 20 minutes, 37 ºC followed by with 2-photon excitation laser at λex = 488 nm. Field of view of 100 μm.

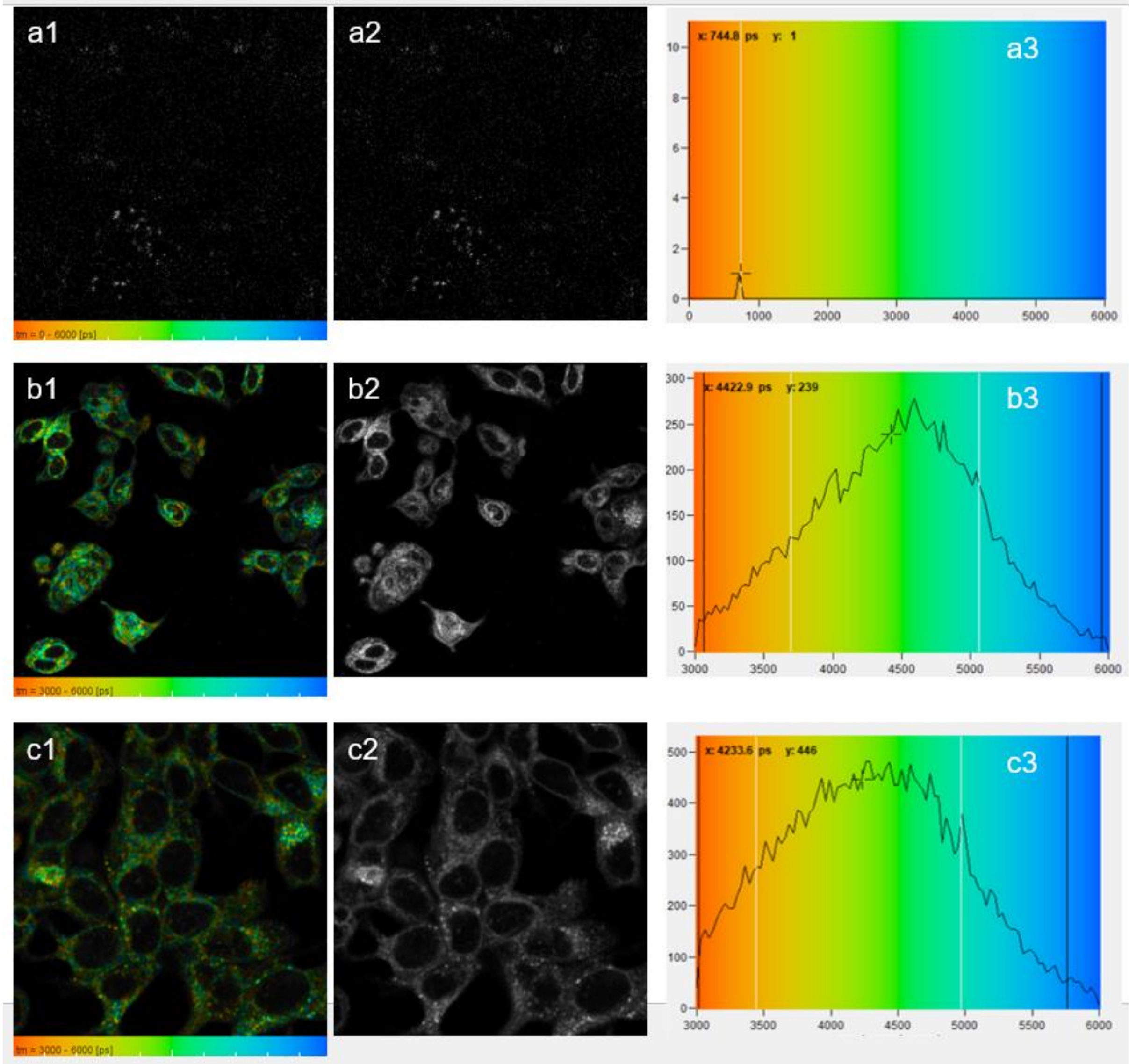

Figure S114. 2-photon FLIM maps, intensity images and *in vitro* fluorescence lifetime distribution histograms (left to right) of PC-3 cells treated with 1 μM of **HL4** (b2-b3) and corresponding **FeL4** (c1-c3) complex, where (a1-a3) represent the micrographs for the control group (1% DMSO). Incubation was 20 minutes, 37 ^{o}C followed by with 2-photon excitation laser at λex = 1010 nm. Field of view of 100 μm.

13. In vivo imaging assays

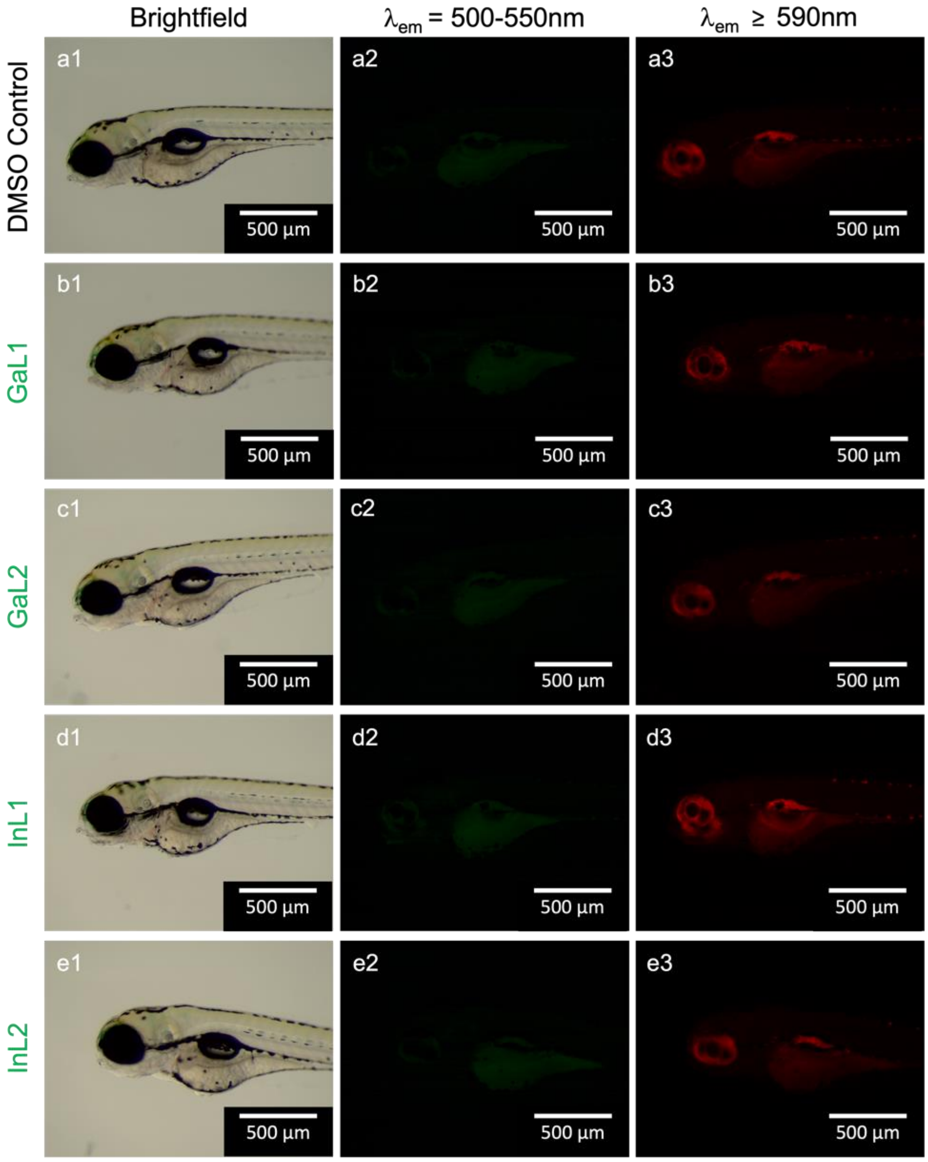

f

**DMSO Control, 120 minutes, 28.5°C**	8/8 viable (100% survival)
**GaL1, 200µM, 120 minutes, 28.5°C**	2/8 viable (25% survival)
**GaL2, 200µM, 120 minutes, 28.5°C**	5/8 viable (62.5% survival)
**InL1, 200µM, 120 minutes, 28.5°C**	1/8 viable (12.5% survival)
**InL2, 200µM, 120 minutes, 28.5°C**	3/8 viable (37.5% survival)

Figure S.115. Representative brightfield and fluorescence stereomicroscope images of 4 dpf zebrafish larvae incubated with compounds at 200 µM for 120 minutes at 28.5°C, where (a) 1% DMSO in embryo media, (b) GaL1, 1% DMSO, (c) GaL2, 1% DMSO, (d) InL1, 1% DMSO, (e) InL2, 1% DMSO. (f) survival following 120 minute incubation, calculated from eight treated larval fish per condition. a1-e1 is the brightfield channel; a2-e2 is the emission detected at λ = 500-550nm, compounds were excited at

450-490nm; a3-e3 is the emission detected at λ ≥ 590nm, compounds were excited at 540-580nm. Scale bar: 500µm.

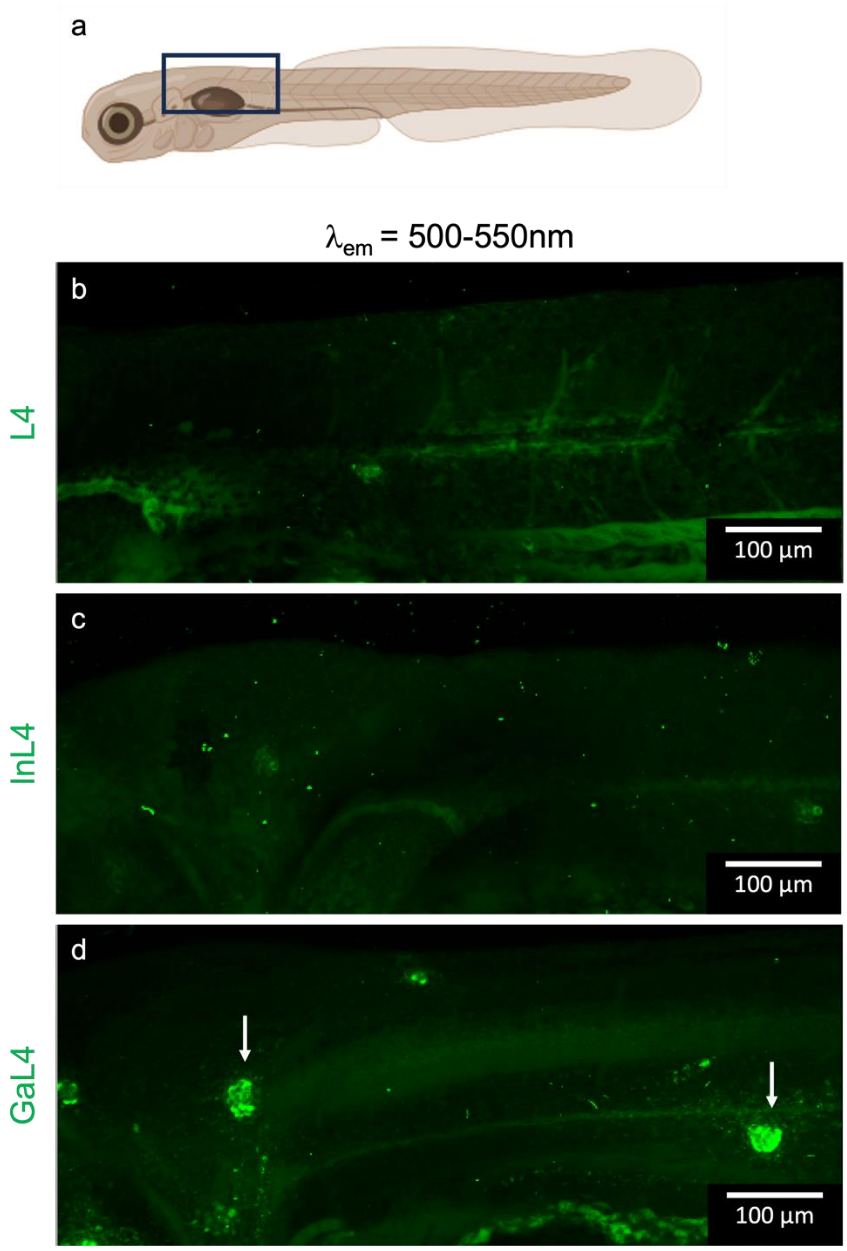

Figure S.116. (a) Schematic demonstrating area of imaging. (b-d) Representative line scanning confocal microscope images of 4 dpf zebrafish larvae incubated with compounds for 120 minutes at 28.5°C, arrows indicate clearly labelled neuromasts, where (b) HL4, 10µM, 1% DMSO, (c) InL4, 10µM, 1% DMSO, (d) GaL4, 10µM, 1% DMSO. b-d is the emission detected at λ = 500-550nm, compounds were excited at 488nm. Scale bar: 100µm.

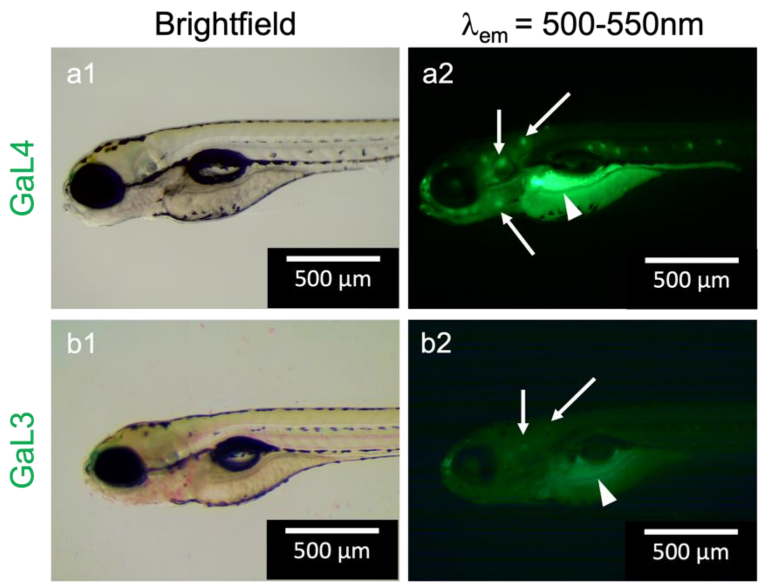

c

GaL4, 10µM, 120 minutes, 28.5°C	GaL3, 10µM, 120 minutes, 28.5°C
8/8 viable (100% survival)	1/8 viable (12.5% survival)

Figure S.117. Representative brightfield and fluorescence stereomicroscope images of 4 dpf zebrafish larvae incubated with compounds at 10µM for 120 minutes at 28.5°C, arrowheads indicate labelled digestive system, arrows indicate labelled neuromasts, where (a) GaL4, 1% DMSO, (b) GaL3, 1% DMSO. (c) indicates relative survival rate between 4dpf zebrafish treated with GaL4 and GaL3 in a side-by-side equimolar experiment. a1-b1 is the brightfield channel; a2-b2 is the emission detected at λ = 500-550nm, compounds were excited at 450-490nm and imaged at the same exposure ($100ms^{-1}$) and gain (2.40) settings. Scale bar: 500µm.

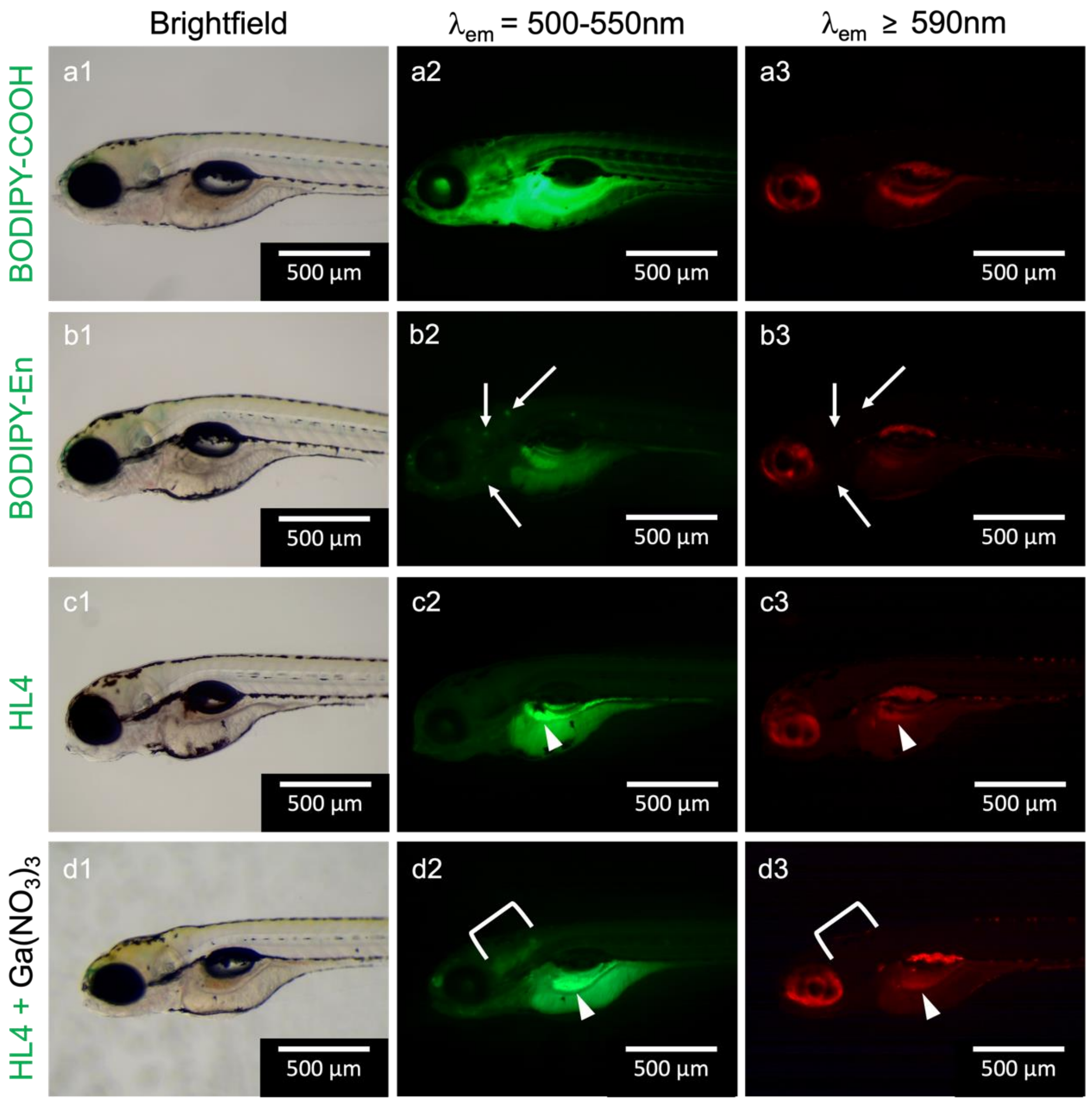

e

10mM $Ga(NO_3)_3$, 360 minutes, 28.5°C	1mM $Ga(NO_3)_3$, 360 minutes, 28.5°C	100µM $Ga(NO_3)_3$, 360 minutes, 28.5°C
0/8 viable (0% survival)	2/8 viable (25% survival)	8/8 viable (100% survival)

Figure S.118. Representative brightfield and fluorescence stereomicroscope images of 4 dpf zebrafish larvae incubated with compounds at 10 µM for 120 minutes at 28.5°C, arrowheads indicate labelled digestive system, arrows indicate labelled neuromasts, square brackets indicates neural tissue, where (a) BODIPY-COOH, 1% DMSO, (c) BODIPY-En, 1% DMSO, (d) HL4, 1% DMSO, (e) HL4, 1% DMSO, pre-incubated in 100 µM $Ga(NO_3)_3$ for 360 minutes. (f) survival following 360 minute incubation, calculated from eight treated larval fish per condition. a1-d1 is the brightfield channel; a2-d2 is the emission detected at λ = 500-550nm, compounds were excited at 450-490nm; a3-d3 is the emission detected at $\lambda \geq$ 590nm, compounds were excited at 540-580nm. Scale bar: 500µm.

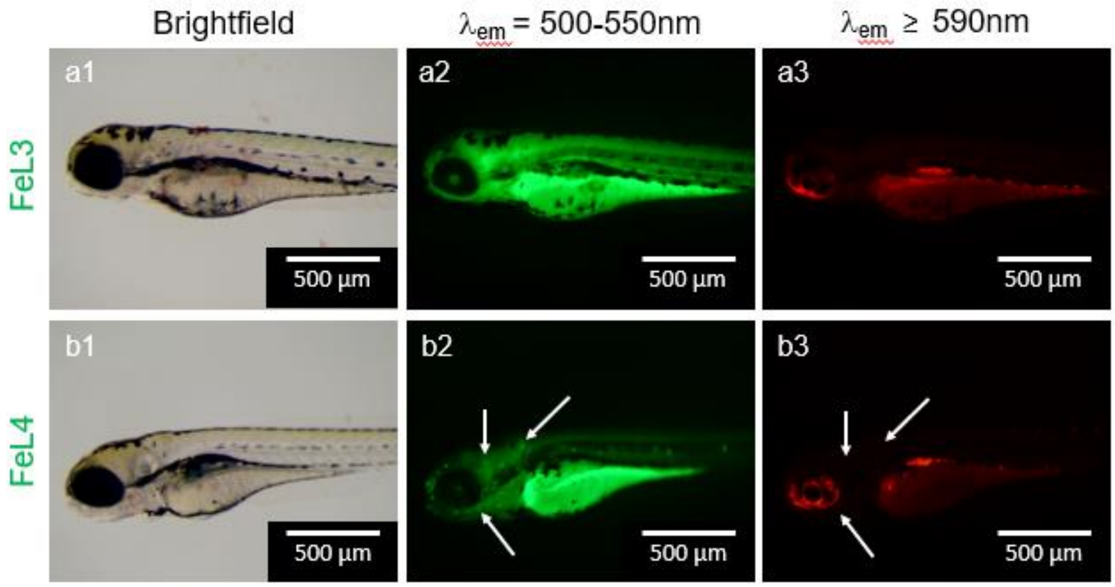

c

FeL3, 50µM, 120 minutes, 28.5°C	FeL4, 50µM, 120 minutes, 28.5°C
1/8 viable (12.5% survival)	8/8 viable (100% survival)

Figure S.119. Representative brightfield and fluorescence stereomicroscope images of 4 dpf zebrafish larvae incubated with compounds at 10µM for 120 minutes at 28.5°C, arrows indicate labelled neuromasts, where (a) FeL3, 1% DMSO, (b) FeL4, 1% DMSO. (c) indicates relative survival rate between 4dpf zebrafish treated with FeL3 and FeL4 in a side-by-side equimolar experiment. a1-b1 is the brightfield channel; a2-b2 is the emission detected at λ = 500-550nm, compounds were excited at 450-490nm and imaged at the same exposure (100ms-1) and gain (2.40) settings. Scale bar: 500µm.

14. X-ray Crystallography

Intensity data for HL1 and **BODIPY-NHS** were collected at 150(2) K on a Rigaku Xcalibur, EosS2 single crystal diffractometer using graphite monochromated Mo-Kα radiation (λ = 0.71073 Å) and for **HL2**, **GaL2**, and **HL4** on a Rigaku SuperNova Dual EosS2 single crystal diffractometer using monochromated Cu-Kα radiation (λ = 1.54184 Å). Unit cell determination, data collection data reduction and absorption correction were performed using the CrysAlisPro software[5]. The structures were solved with SHELXT[6] and refined by a full-matrix least-squares procedure based on F^2 (SHELXL-2018/3)[7]. All non-hydrogen atoms were refined anisotropically. Hydrogen atoms were placed onto calculated positions and refined using a riding model. Where possible hetero atom hydrogen atoms have been in the difference Fourier map and were refined freely or with bond length restraints. Additional programmes used for analysing data and their graphical manipulation included: SHELXle[8], ORTEP3 for windows [9] and Mercury[10.]

Crystal data and structure refinement for compound HL1.

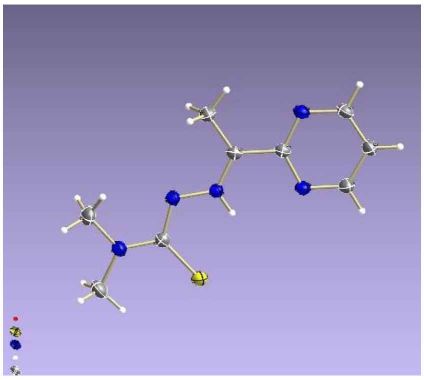

Figure S.120. Molecular structure of Compound **HL1**

Empirical formula	C9 H13 N5 S
Formula weight	223.30
Temperature	150.01(10) K
Wavelength	1.54184 Å
Crystal system	Monoclinic
Space group	$P2_1/n$

Unit cell dimensions	a = 8.06760(10) Å	a= 90°.
	b = 7.09860(10) Å	b= 100.4010(10)°.
	c = 19.2465(2) Å	g = 90°.
Volume	1084.11(2) $Å^3$	
Z	4	
Density (calculated)	1.368 Mg/m^3	
Absorption coefficient	2.455 mm^{-1}	
F(000)	472	
Crystal size	0.299 x 0.139 x 0.127 mm^3	
Theta range for data collection	4.672 to 73.060°.	
Index ranges	-10<=h<=10, -8<=k<=6, -23<=l<=23	
Reflections collected	17190	
Independent reflections	2165 [R(int) = 0.0267]	
Completeness to theta = 67.684°	100.0 %	
Absorption correction	Semi-empirical from equivalents	
Max. and min. transmission	1.00000 and 0.85842	
Refinement method	Full-matrix least-squares on F^2	
Data / restraints / parameters	2165 / 0 / 143	
Goodness-of-fit on F^2	1.037	
Final R indices [I>2sigma(I)]	R1 = 0.0329, wR2 = 0.0915	
R indices (all data)	R1 = 0.0334, wR2 = 0.0921	
Extinction coefficient	n/a	
Largest diff. peak and hole	0.241 and -0.364 e.$Å^{-3}$	

Crystal data and structure refinement for compound GaL2

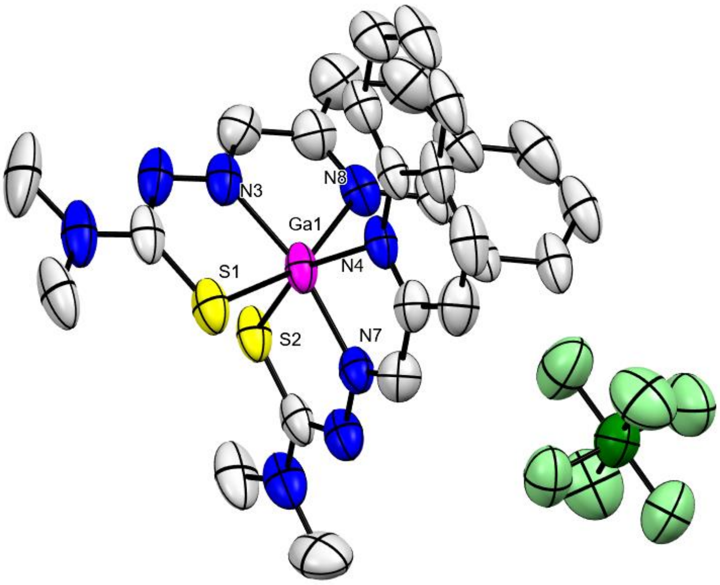

Figure S.121. Molecular structure of Compound **GaL2**

Empirical formula	C26 H26 F6 Ga N8 P S2	
Formula weight	729.36	
Temperature	150.00(10) K	
Wavelength	1.54184 Å	
Crystal system	Triclinic	
Space group	P-1	
Unit cell dimensions	a = 10.2428(6) Å	a= 90.957(3)°.
	b = 13.5493(7) Å	b= 102.821(4)°.
	c = 13.6565(5) Å	g = 94.743(5)°.
Volume	1840.51(16) $Å^3$	
Z	2	
Density (calculated)	1.316 Mg/m^3	
Absorption coefficient	3.014 mm^{-1}	
F(000)	740	
Crystal size	0.160 x 0.080 x 0.040 mm^3	
Theta range for data collection	4.582 to 68.238°.	
Index ranges	-11<=h<=12, -16<=k<=16, -16<=l<=10	
Reflections collected	11192	
Independent reflections	6531 [R(int) = 0.0308]	

Completeness to theta = 67.684°	96.7 %
Absorption correction	Semi-empirical from equivalents
Max. and min. transmission	1.00000 and 0.81462
Refinement method	Full-matrix least-squares on F^2
Data / restraints / parameters	6531 / 0 / 401
Goodness-of-fit on F^2	1.042
Final R indices [I>2sigma(I)]	R1 = 0.0625, wR2 = 0.1622
R indices (all data)	R1 = 0.0826, wR2 = 0.1779
Extinction coefficient	n/a
Largest diff. peak and hole	1.136 and -0.568 e.Å^{-3}

Crystal data and structure refinement for adduct HL2·HPF6

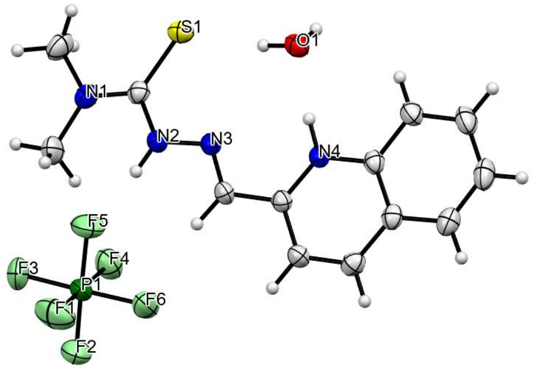

Figure S.122. Molecular structure of **HL2**, the quinoline-protonated adduct with **HPF6**

Identification code	s25sip6	
Empirical formula	C13 H17 F6 N4 O P S	
Formula weight	422.33	
Temperature	150.00(10) K	
Wavelength	1.54184 Å	
Crystal system	Triclinic	
Space group	P-1	
Unit cell dimensions	a = 7.6209(5) Å	α= 86.139(5)°.
	b = 8.5201(6) Å	β= 80.068(5)°.
	c = 13.9454(9) Å	γ = 75.255(6)°.
Volume	862.28(10) Å^3	
Z	2	

Density (calculated)	1.627 Mg/m^3
Absorption coefficient	3.250 mm^{-1}
F(000)	432
Crystal size	0.200 x 0.100 x 0.030 mm^3
Theta range for data collection	5.370 to 72.682°.
Index ranges	-9<=h<=6, -10<=k<=10, -17<=l<=15
Reflections collected	5502
Independent reflections	3344 [R(int) = 0.0244]
Completeness to theta = 67.684°	99.8 %
Absorption correction	Semi-empirical from equivalents
Max. and min. transmission	1.00000 and 0.83929
Refinement method	Full-matrix least-squares on F^2
Data / restraints / parameters	3344 / 0 / 253
Goodness-of-fit on F^2	1.033
Final R indices [I>2sigma(I)]	R1 = 0.0447, wR2 = 0.1153
R indices (all data)	R1 = 0.0543, wR2 = 0.1229
Extinction coefficient	n/a
Largest diff. peak and hole	0.412 and -0.318 e.$Å^{-3}$

Crystal data and structure refinement for compound BODIPY-NHS.

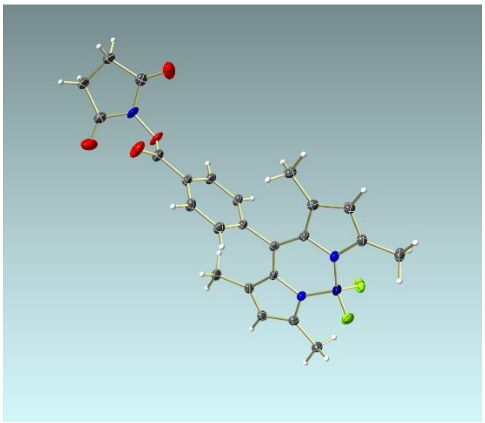

Figure S.123. Molecular structure of Compound **BODIPY-NHS**

Empirical formula	C24 H22 B F2 N3 O4	
Formula weight	465.25	
Temperature	150.00(13) K	
Wavelength	0.71073 Å	
Crystal system	Triclinic	
Space group	P-1	
Unit cell dimensions	a = 6.7299(2) Å	a= 98.752(3)°.
	b = 10.4151(3) Å	b= 96.426(3)°.
	c = 16.3381(6) Å	g = 104.530(3)°.
Volume	1082.08(6) Å^3	
Z	2	
Density (calculated)	1.428 Mg/m^3	
Absorption coefficient	0.109 mm^{-1}	
F(000)	484	
Crystal size	0.587 x 0.225 x 0.042 mm^3	
Theta range for data collection	2.964 to 27.103°.	
Index ranges	-8<=h<=8, -13<=k<=13, -20<=l<=20	
Reflections collected	28994	
Independent reflections	4769 [R(int) = 0.0282]	
Completeness to theta = 25.242°	99.9 %	

Absorption correction	Semi-empirical from equivalents
Max. and min. transmission	1.00000 and 0.94574
Refinement method	Full-matrix least-squares on F^2
Data / restraints / parameters	4769 / 0 / 311
Goodness-of-fit on F^2	1.034
Final R indices [I>2sigma(I)]	R1 = 0.0427, wR2 = 0.1001
R indices (all data)	R1 = 0.0521, wR2 = 0.1052
Extinction coefficient	n/a
Largest diff. peak and hole	0.294 and -0.280 e.Å^{-3}

Crystal data and structure refinement for Compound HL4

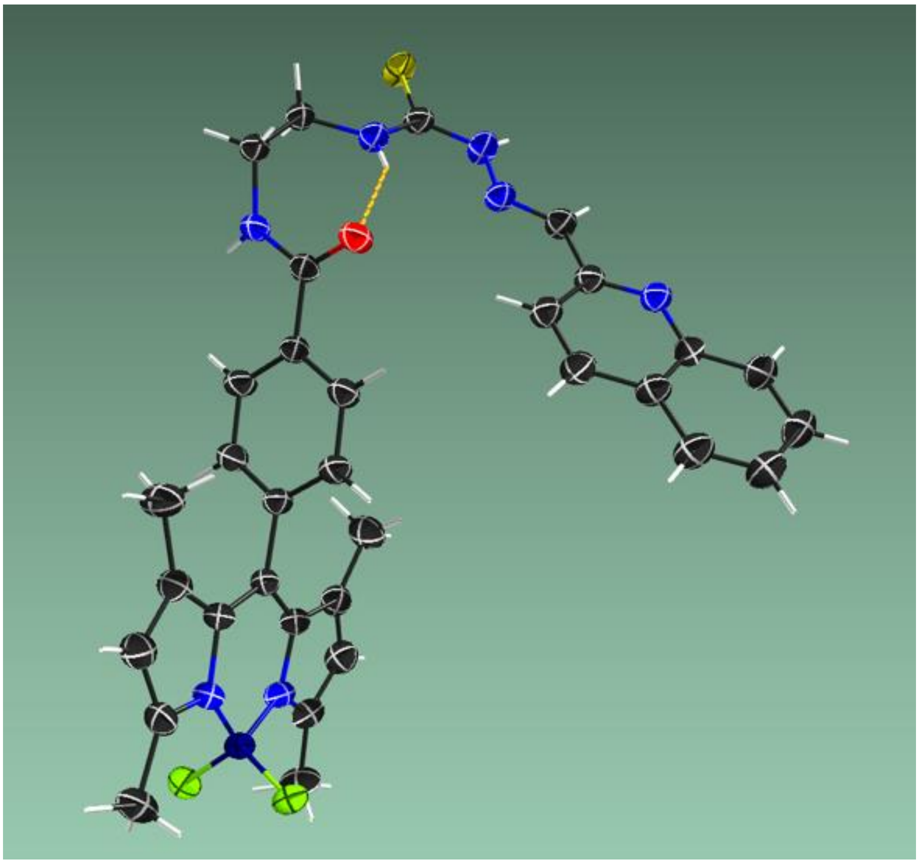

Figure S.124. Molecular structure of Compound **HL4**

Empirical formula	C33 H32 B F2 N7 O S	
Formula weight	623.52	
Temperature	150.00(13) K	
Wavelength	1.54184 Å	
Crystal system	Triclinic	
Space group	P-1	
Unit cell dimensions	a = 10.3033(2) Å	〈= 110.156(2)°.
	b = 10.7787(2) Å	®= 93.819(2)°.
	c = 14.7436(4) Å	© = 95.137(2)°.
Volume	1522.58(6) $Å^3$	
Z	2	
Density (calculated)	1.360 Mg/m^3	
Absorption coefficient	1.381 mm^{-1}	
F(000)	652	
Crystal size	0.150 x 0.100 x 0.020 mm^3	
Theta range for data collection	3.211 to 79.102°.	

Index ranges	-12<=h<=13, -13<=k<=11, -18<=l<=18
Reflections collected	20979
Independent reflections	6174 [R(int) = 0.0584]
Completeness to theta = 67.684°	99.1 %
Absorption correction	Semi-empirical from equivalents
Max. and min. transmission	1.00000 and 0.79789
Refinement method	Full-matrix least-squares on F^2
Data / restraints / parameters	6174 / 0 / 422
Goodness-of-fit on F^2	1.040
Final R indices [I>2sigma(I)]	R1 = 0.0497, wR2 = 0.1296
R indices (all data)	R1 = 0.0630, wR2 = 0.1382
Extinction coefficient	n/a
Largest diff. peak and hole	0.313 and -0.334 e.Å^{-3}

Crystal data and structure refinement for Compound GaL1

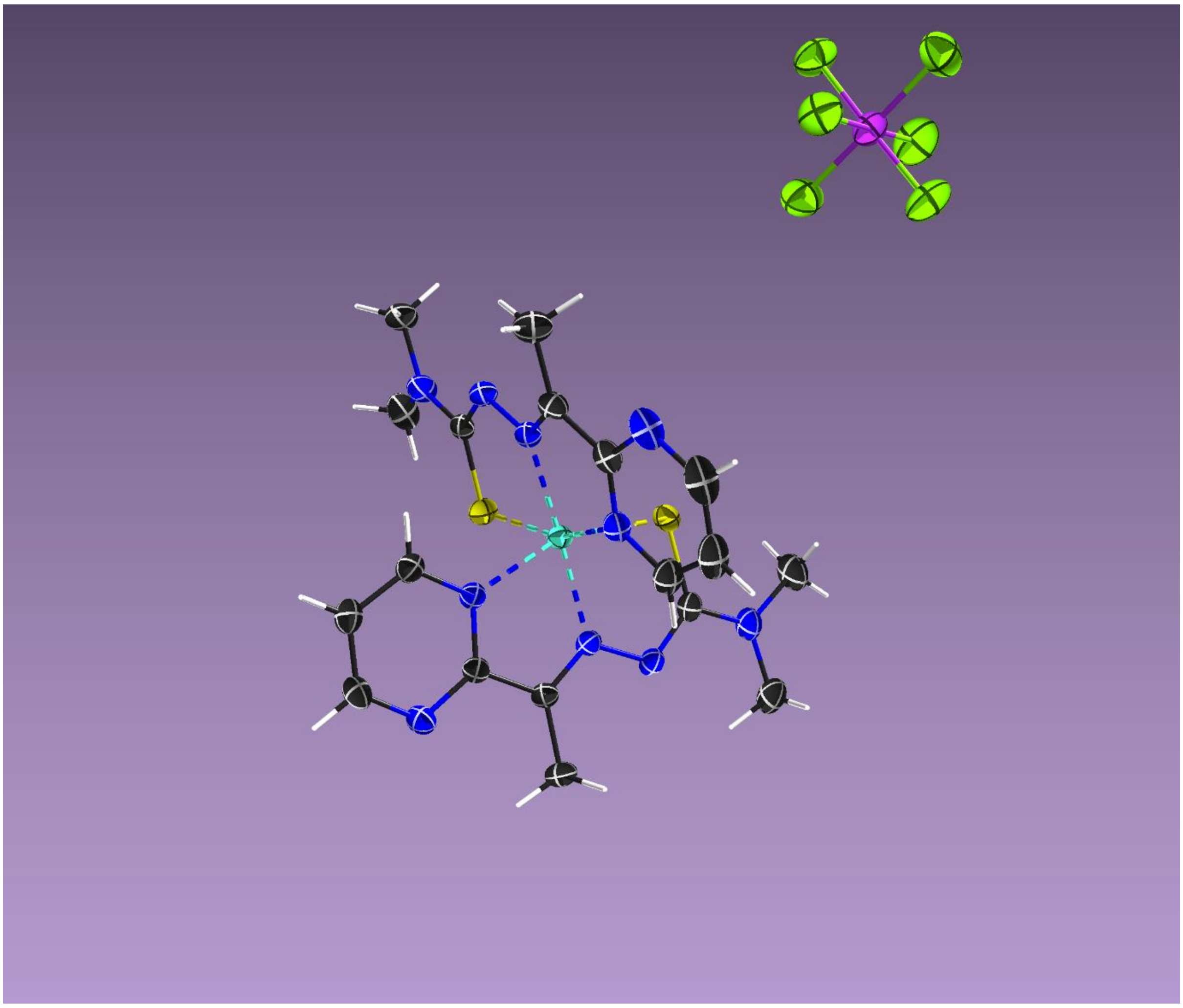

Figure S.125. Molecular structure of Compound **GaL1**

Empirical formula	C18 H24 F6 Ga N10 P S2	
Formula weight	659.28	
Temperature	150.00(10) K	
Wavelength	1.54184 Å	
Crystal system	Triclinic	
Space group	P-1	
Unit cell dimensions	a = 9.06271(17) Å	α= 78.2162(15)°.
	b = 11.26613(19) Å	β= 86.0905(15)°.
	c = 13.6776(2) Å	γ = 74.9223(16)°.
Volume	1319.84(4) $Å^3$	
Z	2	
Density (calculated)	1.659 Mg/m^3	
Absorption coefficient	4.146 mm^{-1}	
F(000)	668	
Crystal size	0.280 x 0.180 x 0.060 mm^3	
Theta range for data collection	3.301 to 78.346°.	
Index ranges	-10<=h<=11, -14<=k<=14, -17<=l<=17	
Reflections collected	45320	
Independent reflections	5590 [R(int) = 0.0400]	
Completeness to theta = 67.684°	100.0 %	
Absorption correction	Semi-empirical from equivalents	
Max. and min. transmission	1.00000 and 0.42739	
Refinement method	Full-matrix least-squares on F^2	
Data / restraints / parameters	5590 / 0 / 349	
Goodness-of-fit on F^2	1.083	
Final R indices [I>2sigma(I)]	R1 = 0.0278, wR2 = 0.0765	
R indices (all data)	R1 = 0.0294, wR2 = 0.0776	
Extinction coefficient	n/a	
Largest diff. peak and hole	0.356 and -0.430 e.$Å^{-3}$	

Crystal data and structure refinement for Compound InL2

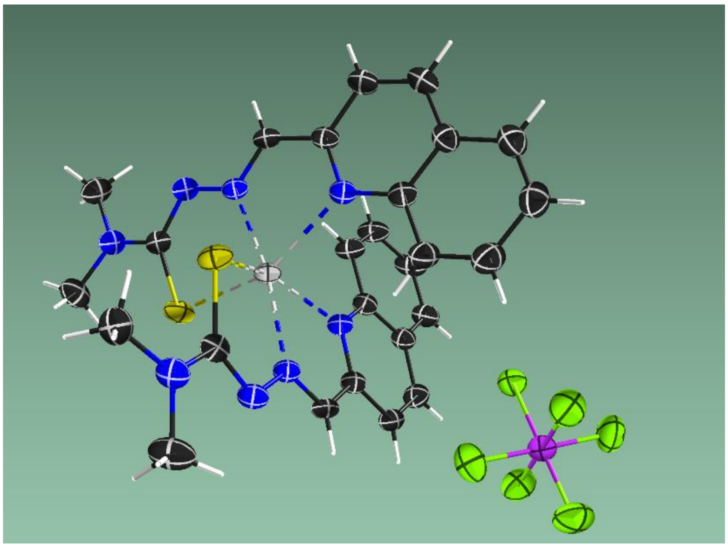

Figure S.126. Molecular structure of Compound **InL2**

Bond precision: C-C = 0.0049 A Wavelength=1.54184

Cell:	a=14.5176(9)	b=13.06691(8)	c=16.48862(11)
	alpha=90	beta=102.6572(6)	gamma=90
Temperature:	150 K		

	Calculated	Reported
Volume	3051.88(19)	3051.88(19)
Space group	P 21/n	P 21/n
Hall group	-P 2yn	-P 2yn
Moiety formula	C26 H26 In N8 S2, F6 P	C26 H26 In N8 S2, F6 P
Sum formula	C26 H26 F6 In N8 P S2	C26 H26 F6 In N8 P S2
Mr	774.46	774.46
Dx,g cm-3	1.686	1.686
Z	4	4
Mu (mm-1)	8.591	8.591
F000	1552.0	1552.0
F000'	1560.77	
h,k,lmax	18,16,20	18,16,20
Nref	6595	6511
Tmin,Tmax	0.266,0.651	0.333,1.000
Tmin'	0.144	

Correction method= # Reported T Limits: Tmin=0.333 Tmax=1.000
AbsCorr = MULTI-SCAN

Data completeness= 0.987 Theta(max)= 79.001

R(reflections)= 0.0381(6091) wR2(reflections)= 0.0947(6511)

S = 1.052 Npar= 401

15. References

1. American Type Culture Collection (ATCC), https://www.atcc.org/products/crl-1435 (accessed July 2024).
2. American Type Culture Collection (ATCC), HeLa, https://www.atcc.org/products/crm-ccl-2 (accessed July 2024).
3. Coriell Institute for Medical Research, https://www.coriell.org/0/Sections/Search/Sample_Detail.aspx?Ref=AG09429&Product=CC (accessed July 2024).
4. C. Dardonville, C. Fernandez-Fernandez, S.-L. Gibbons, G. J. Ryan, N. Jagerovic, A. M. Gabilondo, J. J. Meana, L. F. Callado, *Bioorg. Med. Chem.* **2006**, *14*, 6570–6580.
5. *CrysAlisPro*, version 1.171.39.46, Rigaku Oxford Diffraction, 2018.
6. G. M. Sheldrick, *Acta Crystallogr. C* **2015**, *71*, 3–8.
7. C. B. Hübschle, G. M. Sheldrick, B. Dittrich, *J. Appl. Crystallogr.* **2011**, *44*, 1281–1284.
8. L. J. Farrugia, *J. Appl. Crystallogr.* **1997**, *30*, 565.
9. C. F. Macrae, P. R. Edgington, P. McCabe, E. Pidcock, G. P. Shields, R. Taylor, M. Towler, J. van de Streek, *J. Appl. Crystallogr.* **2006**, *39*, 453–457.
10. S. Sarpaki, F. Cortezon-Tamarit, S. R. M. M. de Aguiar, R. M. Exner, D. Divall, R. L. Arrowsmith, H. Ge, F. J. Palomares, L. Carroll, D. G. Calatayud, S. J. Paisey, E. O. Aboagye, S. I. Pascu, *Nanoscale* **2020**, *12*, 6603–6608. https://doi.org/10.1039/c9nr10145d
11. J. W. Akitt, N. N. Greenwood, A. Stor, *J. Chem. Soc.* **1965**, 4410–4416.
12. J. D. Glickson, T. P. Pitner, J. Webb, R. A. Gams, *J. Am. Chem. Soc.* **1975**, *97*, 1679–1683.
13. M. J. Taylor, *Polyhedron* **1990**, *9*, 207–214.
14. Y. Sun, C. J. Anderson, T. S. Pajeau, D. E. Reichert, R. D. Hancock, R. J. Motekaitis, A. E. Martell, M. J. Welch, *J. Med. Chem.* **1996**, *39*, 458–470.
15. P. Bénézeth, I. I. Diakonov, G. S. Pokrovski, J.-L. Dandurand, J. Schott, I. L. Khodakovsky, *Geochim. Cosmochim. Acta* **1997**, *61*, 1345–1357.
16. Y. Toporivska, A. Mular, K. Piasta, M. Ostrowska, D. Illuminati, A. Baldi, V. Albanese, S. Pacifico, I. O. Fritsky, M. Remelli, R. Guerrini, E. Gumienna-Kontecka, *Inorg. Chem.* **2021**, *60*, 13332–13347.
17. D. E. Runacres, V. K. Greenacre, J. M. Dyke, J. Grigg, G. Herbert, W. Levason, G. McRobbie, G. Reid, *Inorg. Chem.* **2023**, *62*, 20844–20857.
18. V. Lebruška, T. Dobrovolná, T. Gemperle, V. Kubíček, S. Kossatz, P. Hermann, *Dalton Trans.* **2024**, *53*, 17554–17564.
19. I. Ryza, C. Granata, N. Ribeiro, E. Nalewajko-Sieliwoniuk, A. Kießling, M. Hryniewicka, W. Plass, B. Godlewska-Żyłkiewicz, *J. Inorg. Biochem.* **2024**, *260*, 112670.
20. V. V. Gaensicke, S. Bachmann, L. Craciunescu, A. W. Prentice, M. J. Paterson, D. Iuga, P. J. Sadler, R. C. Marchi, *Dalton Trans.* **2025**, *54*, 5446–5457.
21. Y. Zhu, Z. Cheng, J. Zhang, L. Feng, A. Mishra, R. T. M. de Rosales, R. K. Brown, N. Long, G.-L. Law, *JACS Au* **2025**, https://doi.org/10.1021/jacsau.5c00946.
